# Hyperinflammatory reprogramming marks the transition from MASH to HCC

**DOI:** 10.1016/j.jhepr.2026.102055

**Published:** 2026-10-07

**Authors:** Shawn Lu Wen Tan, Erez Israeli, Jillian Wee, Hui Min Tan, Ravisankar Rajarethinam, Immanuel Kwok, Shu Zhen Chong, Weiping Han

**Affiliations:** 1 Institute of Molecular and Cell Biology (IMCB), Agency for Science, Technology and Research (A^∗^STAR), 61 Biopolis Drive, Proteos, 138673, Singapore; 2 Singapore Immunology Network, Agency for Science, Technology and Research (A^∗^STAR), 8A Biomedical Grove, Immunos, 138648, Singapore

**Keywords:** liver cancer, tumor microenvironment, lipid-associated macrophage, granulopoiesis, endothelial activation

## Abstract

**Background & Aims:**

Up to 40% of metabolic dysfunction-associated steatohepatitis (MASH)-related hepatocellular carcinoma (HCC) occurs in the absence of cirrhosis. Yet the biological features underlying non-cirrhotic MASH-to-HCC progression remain poorly understood. We sought to define the cellular and molecular programs associated with non-cirrhotic MASH-to-HCC progression.

**Methods:**

We integrated histopathological and biochemical assessments, bulk and single-cell RNA sequencing, serum cytokine profiling, and multiplex immunohistochemistry in Gubra-Amylin-NASH diet-fed mice that remained HCC-naïve or developed non-cirrhotic HCC. Key findings were evaluated in an independent human cohort with non-cirrhotic MASH and MASH-HCC.

**Results:**

Body composition metrics, fibrosis severity, histopathology, and liver injury markers poorly distinguished HCC progressors from non-progressors. In contrast, bulk transcriptomics identified prominent immune dysregulation in cancer-primed adjacent non-tumor liver. Single-cell RNA sequencing revealed coordinated hyperinflammatory reprogramming characterized by loss of *Trem2*^+^ macrophages, expansion of *Trem2*^low^ pro-inflammatory macrophages, accumulation of immature neutrophils, T helper 17 skewing and heightened activation of dendritic and endothelial cells. Cell-cell communication analysis identified IL-1 as the most altered signaling pathway in HCC progressors (pathway distance = 7.95, top-ranked), with myeloid-derived IL-1β signaling emerging as a dominant axis targeting endothelial and immune populations. Elevated circulating IL-6 (154.3 *vs.* 30.1 pg/ml, *p* = 0.065) and TNF (25.2 *vs.* 16.0 pg/ml, *p* = 0.062) in HCC progressors were consistent with a systemic hyperinflammatory phenotype. Human non-cirrhotic MASH-HCC adjacent non-tumor tissues similarly exhibited increased *IL1B* expression (log_2_ expression = 4.21 *vs.* 4.03, *p* <0.01) and enrichment of IL-1β-responsive inflammatory programs.

**Conclusions:**

Non-cirrhotic MASH-to-HCC progression is associated with coordinated hyperinflammatory reprogramming across immune and stromal compartments. Myeloid-derived IL-1β is a central signaling axis within the cancer-permissive MASH microenvironment, providing a potential basis for HCC risk stratification and preventive intervention.

**Impact and implications:**

The biological factors that distinguish patients with metabolic dysfunction-associated steatohepatitis (MASH) who progress to hepatocellular carcinoma (HCC) in the absence of cirrhosis remain poorly understood, limiting current approaches to HCC risk stratification and surveillance. Here, we identify coordinated hyperinflammatory reprogramming across immune and stromal compartments, centered on a myeloid-driven IL-1β signaling network, as a defining feature associated with non-cirrhotic MASH-HCC progression. These findings are particularly relevant to patients with non-cirrhotic MASH who may fall outside current fibrosis-based HCC surveillance strategies, and raise the possibility that inflammatory signatures could complement existing clinical parameters to identify individuals at elevated HCC risk. Prospective studies and functional perturbation will be required to establish whether this hyperinflammatory state precedes and contributes causally to HCC development, and to determine its utility for patient stratification and preventive intervention.

## Highlights

• Non-cirrhotic MASH-HCC progression is marked by coordinated hyperinflammatory reprogramming.
• *Trem2*^low^ macrophages and immature neutrophils accumulate in cancer-primed MASH liver.
• Dendritic and endothelial cells acquire activated, pro-inflammatory states during HCC progression.
• Myeloid-derived IL-1β emerges as a dominant signaling axis linking immune and stromal compartments.
• Human non-cirrhotic MASH-HCC recapitulates IL-1β-associated inflammatory programs.

## Graphical abstract

**Figure:**
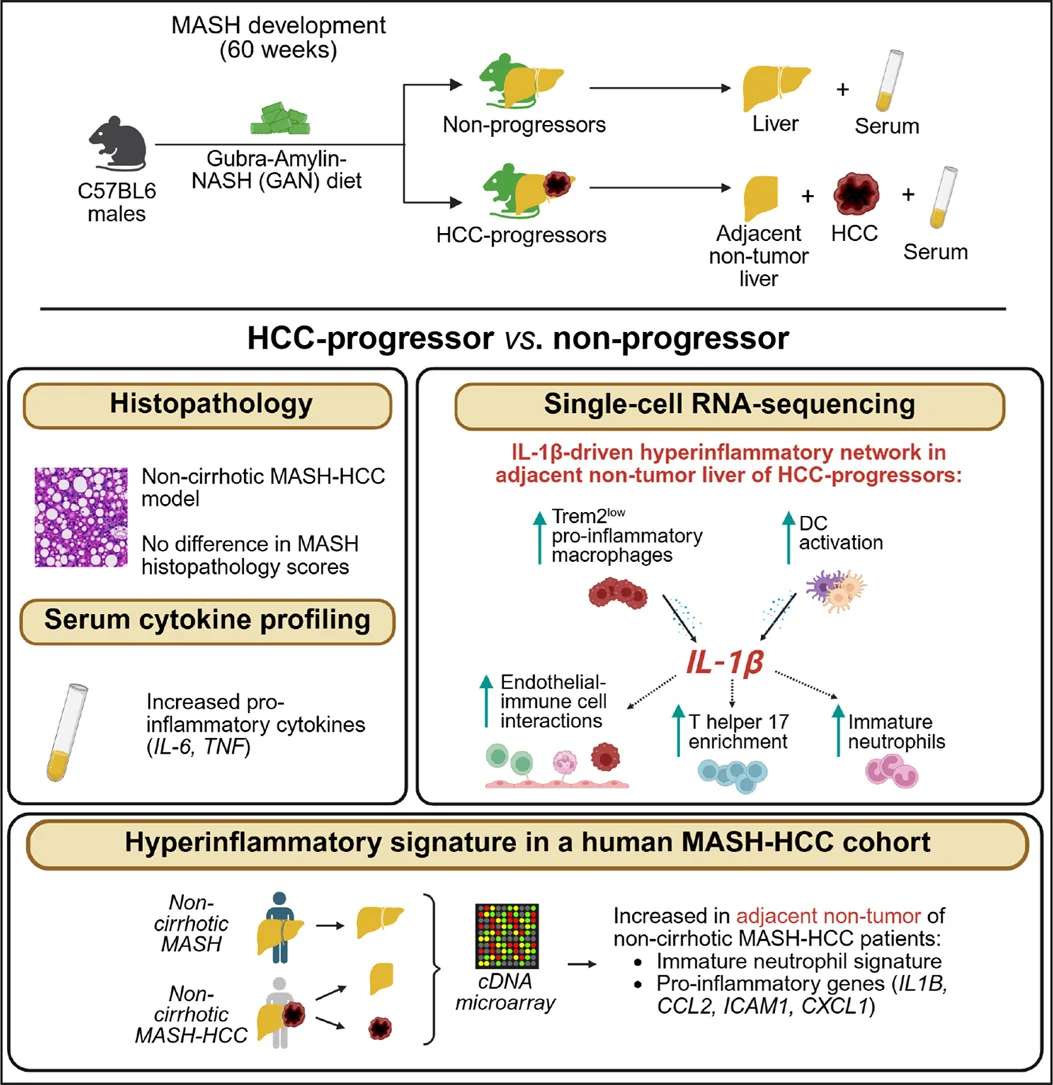

## Introduction

Up to 30-50% of metabolic dysfunction-associated steatohepatitis (MASH)-related hepatocellular carcinoma (HCC) cases develop in the absence of cirrhosis, placing many patients outside current HCC surveillance strategies.^1^ This is a challenge compounded by the fast-growing global burden of MASH.^2^ Therapeutic options for advanced MASH-related HCC remain relatively limited, with evidence suggesting that patients with MASH-HCC are relatively resistant to immune checkpoint inhibitor combination therapies compared to HCCs derived from other etiologies (*e.g*. viral hepatitis).^3^ Collectively, these factors underscore the need to define the mechanisms driving HCC development in non-cirrhotic MASH to improve cancer prevention and patient risk stratification.

MASH is driven by a confluence of factors, including lipotoxicity, endoplasmic reticulum stress, and chronic inflammation, resulting in sustained liver injury and fibrosis.[2], [4] MASH studies have implicated specific immune subsets in HCC development, including neutrophils,^5^ immunoglobulin A-producing B cell subsets,^6^ and ‘auto-aggressive’ cytotoxic CD8^+^ T cell populations,[3], [7] which emerge in pre-malignant MASH livers and promote hepatocarcinogenesis. These findings highlight the pivotal role of liver immunity in MASH-associated HCC. However, an unbiased, integrative view of how diverse immune populations evolve in concert across the MASH-to-HCC transition remains lacking, limiting our understanding of its immunological underpinnings.

Here, we employ a diet-induced murine model of MASH that progresses to non-cirrhotic HCC with partial penetrance, enabling dissection of features distinguishing HCC progressors from non-progressors. Bulk transcriptomics of adjacent non-tumor liver and serum cytokine profiling revealed a hyperinflammatory state in HCC progressors compared with non-progressors. Single-cell transcriptomics further generated a single-cell atlas spanning HCC-naïve MASH liver, adjacent non-tumor tissue and HCC, providing a cross-sectional view of the MASH-to-HCC transition. Together, our findings establish a foundation for further mechanistic dissection of HCC-promoting immune alterations in MASH that may inform future strategies for HCC prevention and early detection.

## Materials and methods

### *In vivo* mouse studies

Six-week-old male C57BL6/J mice (Invivos) were acclimatized until 8 weeks of age, then fed either GAN diet (Research Diets, D09100310i) or low-fat diet (LFD) (D09100304i) for 16, 32 or 55–60 weeks. Body weight was measured weekly and body composition monthly using EchoMRI. At harvest, blood was collected by cardiac puncture for serum preparation, and livers were weighed, photographed and processed for downstream analyses. Liver tissues were either snap-frozen, preserved in RNAlater or formalin-fixed and paraffin-embedded. Serum and frozen tissues were stored at −80°C. Mice were maintained at the A∗STAR Biological Resource Centre under Institutional Animal Care and Use Committee approval (IACUC No. 231792).

### Serum proteomics

Mouse serum samples were analyzed using the Olink Target 48 Mouse Cytokine Panel (Olink Proteomics, Uppsala, Sweden) based on proximity extension assay (PEA) technology.

### Pathology evaluation of H&E liver sections

H&E- and Sirius Red-stained liver sections were evaluated by a certified veterinary pathologist blinded to treatment groups. Steatosis was scored as 0 (<5% hepatocytes affected), 1 (5–33%), 2 (>33–66%) or 3 (>66%); lobular inflammation as 0 (no foci), 1 (<2 foci), 2 (2–4 foci) or 3 (>4 foci per 200 × field); and hepatocyte ballooning as 0 (none), 1 (few ballooned hepatocytes) or 2 (many/prominent ballooned hepatocytes). MASLD activity score was calculated as the sum of these three scores. Fibrosis was staged as 0 (none), 1A (mild zone 3 perisinusoidal), 1B (moderate zone 3 perisinusoidal), 1C (portal/periportal), 2 (perisinusoidal and portal/periportal), 3 (bridging) or 4 (cirrhosis). Large lipogranulomas were scored as absent (0) or present (1).

### Single-cell RNA sequencing

Liver single-cell suspensions were labeled with TotalSeq-B anti-mouse hashtag antibodies (BioLegend), washed and resuspended in PBS with 1% bovine serum albumin. Two pools of five samples were generated using equal cell numbers and non-overlapping hashtags. Each pool was loaded onto the Chromium iX (10x Genomics) targeting 21,500 recovered cells. Gene expression and Feature Barcode libraries were prepared using the 10x Genomics Next GEM Single Cell 3′ v3.1 and 3′ Feature Barcode kits according to the manufacturer’s protocol. Libraries were sequenced on an Illumina NovaSeq 6000 (2 × 151 bp), targeting 50,000 reads/cell for gene expression and 5,000 reads/cell for Feature Barcodes.

### Pre-processing of scRNA-sequencing data, quality control and clustering

Raw reads were processed using Cell Ranger v7.1.0 and analyzed using Seurat v5.0.0. Quality control was performed on individual samples using mitochondrial read percentage, total counts, detected genes and count-to-gene relationships, as detailed in Supplementary Methods. Data were normalized using *SCTransform*, integrated using *IntegrateData*, and subjected to clustering and uniform manifold approximation and projection (UMAP) visualization. Clusters and subclusters were annotated based on differential marker expression.

### Cell-cell communication analysis

Cell–cell communication was inferred using CellChat v2.1.0^39^ after excluding hepatocytes and cholangiocytes. GAN and GAN-Adj.NT cells were analyzed separately, with communication probabilities inferred between subclusters using *computeCommunProb* (type = "truncatedMean"). *LiftCellChat* was used to harmonize subcluster identities across groups, and subclusters with <10 cells in either group were excluded from comparative analyses.

### mRNA *in situ* hybridisation

*Camp* mRNA was detected in 5-μm mouse FFPE liver sections using a murine *Camp* RNAscope probe (ACDbio, 446231) and RNAscope 2.5 HD Reagent Kit–Red (ACDbio, 322373) according to the manufacturer’s instructions.

### Processing of human MASH-HCC microarray dataset

Human MASH and MASH-HCC liver microarray data and metadata were obtained from GEO (GSE164760). Probes lacking unique gene annotations or with ambiguous RefSeq assignments were excluded, and multiple probes mapping to the same gene were averaged. Gene-level expression values were log_2_-transformed and quantile-normalized using *preprocessCore*. PCA outliers (|z-score| >2.5 for PC1 or PC2) were excluded. Single-sample gene set enrichment analysis was performed using GSVA v1.44.5 (method = "ssgsea").

### Multiplex immunohistochemistry

Multiplex immunohistochemistry of liver FFPE sections was performed using the MACSima platform as previously described.^50^ Following deparaffinization, antigen retrieval, permeabilization and blocking, sections underwent iterative staining with FITC-, PE- and APC-conjugated antibodies and imaging on the MACSima instrument. Images were processed using MACS iQ View software. Antibodies used were anti-TREM2 (Abcam, ab305103), anti-F4/80 (Miltenyi Biotec, 130-116-499), anti-CD31 (130-111-710), anti-CD146 (130-118-407), anti-albumin (Proteintech, 16475-1-AP) and anti-α-SMA (Miltenyi Biotec, 130-123-363).

### Quantification and statistical analyses

Statistical analyses were performed in R. Logistic regression was performed using *glm(family* = *“binomial”)*. Multiple-testing correction used the Benjamini–Hochberg method unless otherwise stated. Significance was denoted as: *p* >0.1, n.s.; *p* <0.1, #; *p* <0.05, ∗; *p* <0.01, ∗∗; *p* <0.001, ∗∗∗.

## Results

### A murine model of non-cirrhotic MASH-to-HCC progression with partial HCC penetrance

To model MASH-HCC development, we challenged C57BL6 male mice *ad libitum* with the Gubra-Amylin-NASH (GAN) diet,^8^ which is composed of 40% kcal fat, 20% kcal fructose and 2% kcal cholesterol for up to 60 weeks. A calorie-matched diet containing 10% kcal fat was used as a control diet (LFD). A total of 89 and 52 mice were placed on GAN and LFD diets, respectively, and sampled at 16, 32 and 55-60 weeks (hereafter 60 weeks) to characterize MASH-to-HCC progression (Fig. 1A). As expected, GAN diet exposure increased body weight, fat mass and liver weight and impaired glucose homeostasis (Fig. S1A–C). By 16 weeks, >90% of GAN-fed mice exhibited significant hepatic steatosis (score ≥2), preceding hepatocyte proliferation (Ki67) and fibrosis (Sirius Red), which emerged at 32 and 60 weeks, consistent with progression from steatosis to MASH (Fig. S1D and E). Bridging fibrosis (stage 3, 42%), increased lobular inflammation (score ≥2, 53%), high MASLD activity scores (score ≥5, 37.5%) and lipogranulomas (87.5%) emerged only in the 60-week GAN cohort, indicating advanced MASH (Fig. S1D and E). Hepatocyte ballooning was infrequent (3/64 mice; Fig. S1E), consistent with its limited prevalence in murine MASH models.^9^

**Fig. 1:**
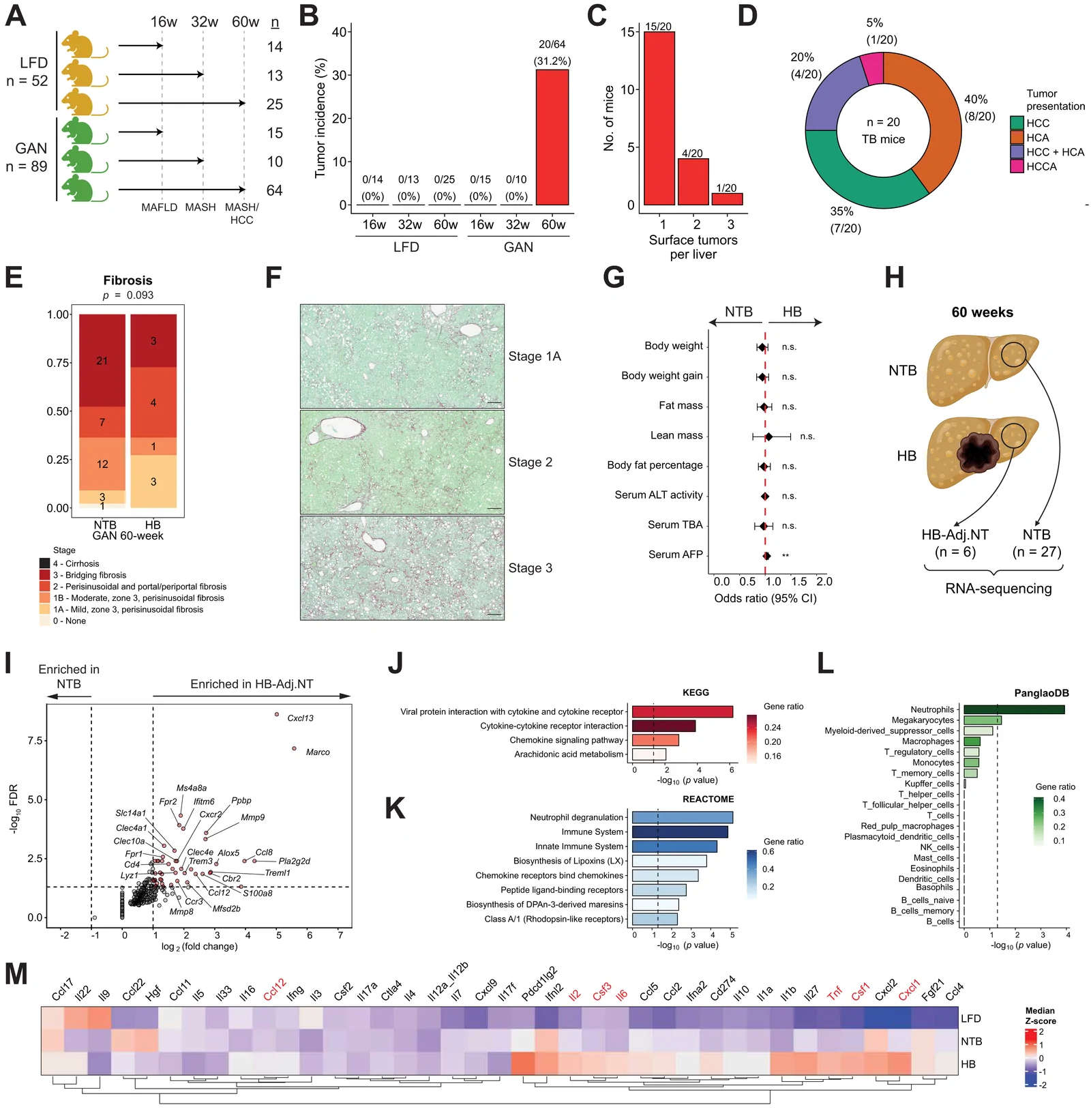
A dietary model of non-cirrhotic MASH-to-HCC progression with partial HCC penetrance highlights an augmented local and systemic inflammatory response in HCC progressors. (A) Experimental set-up for the GAN murine model of MASH-to-HCC progression. (B) Liver tumor incidence across MASH-to-HCC progression in the GAN model. (C) Number of surface tumor nodules more than 2 mm^3^ in each tumor-bearing liver. (D) Tumor presentation of tumor-bearing mice. (E) Histopathology scoring of NTB and HB 60-week GAN liver sections for fibrosis stage. Fisher’s exact test. (F) Representative images of non-tumor regions of 60-week HB liver sections stained with picrosirius red and counterstained with Fast Green. Liver sections from three independent HB mice displaying the indicated fibrosis stages are shown. Scale bar represents 200 μm. (G) Logistic regression of metabolic and serum parameters of 60-week GAN-treated NTB and HB mice. (H) Schematic of bulk RNA-sequencing of 27 NTB liver tissues and 6 HB-Adj.NT tissues from HB mice. (I) Differential expression analysis of bulk transcriptomes of 60-week GAN-treated Adj.NT liver samples of HB mice and NTB livers. BH-adjusted *p* values. (J) KEGG pathway enrichment of 48 upregulated DEGs in HB-Adj.NT tissues. *p* values calculated using the g:SCS algorithm of gProfiler2. (K) Reactome pathway enrichment of 48 upregulated DEGs in HB-Adj.NT tissues. *p* values calculated using the g:SCS algorithm of gProfiler2. (L) Immune signature enrichment using signatures from PanglaoDB of 48 upregulated DEGs in HB-Adj.NT tissues. *p* values calculated using the g:SCS algorithm of gProfiler2. (M) Relative abundance of cytokines in sera of 60-week mice measured using the Olink Target 48 mouse cytokine panel. Highlighted in red are cytokines that show a significant difference (*p* <0.1) between NTB and HB mice. *Post hoc* Dunn’s test following Kruskal-Wallis test, Benjamini-Hochberg adjusted *p* values. Adj.NT, adjacent non-tumor; BH, Benjamini-Hochberg; DEG, differentially expressed gene; GAN, Gubra-Amylin-NASH; HB, HCC-bearing; HCA, hepatocellular adenoma; HCC, hepatocellular carcinoma; HCCA, hepatocholangiocellular adenoma; MASH, metabolic dysfunction-associated steatohepatitis; NTB, non-tumor-bearing.

At 60 weeks, 20/64 GAN mice (31.2%) developed visible liver tumors, with none observed at earlier timepoints (Fig. 1B). Tumor burden was generally low, with 19/20 tumor-bearing (TB) mice harboring only 1–2 visible nodules (Figs 1C and S1F). Histopathological evaluation of H&E-stained liver sections from TB mice identified HCC in 7/20 mice, hepatocellular adenoma (HCA) in 8/20, both HCC and HCA in 4/20, and hepatocholangiocellular adenoma (HCCA) in 1/20 (Fig. 1D). HCCs exhibited diverse histological subtypes resembling those observed in human HCC, including trabecular HCCs with varying steatosis (Fig. S1G–I), steatohepatitic HCCs (Fig. S1J), clear cell-like variants (Fig. S1K,L), and mixed solid-trabecular patterns (Fig. S1M,N). One HCC displayed signs of vascular invasion, indicative of advanced disease (Fig. S1O). Thus, this model recapitulates MASH-to-HCC progression with partial HCC penetrance and histological features of human HCC.

To focus on MASH-to-HCC progression, we excluded 60-week GAN mice with only HCAs or HCCAs and stratified the remaining 60-week GAN cohort as non-tumor-bearing (NTB, n = 44) or HCC-bearing (HB, n = 11). Notably, 8/11 HB mice developed HCC on a background of stage ≤2 fibrosis, and none had cirrhosis (stage 4) (Fig. 1E,F). Fibrosis severity also trended lower in HB than NTB livers, together indicating that advanced fibrosis was not required for HCC development in this model. HB livers also showed a modest trend towards lower steatosis, potentially reflecting altered lipid metabolism, while other histopathological features were comparable between groups (Fig. S2A).

We next compared body composition, serum alanine aminotransferase (ALT) and total bile acids as markers of hepatocellular injury and hepatobiliary dysfunction, respectively, as well as the HCC biomarker alpha-fetoprotein (AFP), between HB and NTB mice and assessed their association with HCC progression by logistic regression. Serum AFP was the only marker significantly elevated in HB mice (Fig. S2B) but only modestly predictive of HCC onset (odds ratio = 1.02, *p* = 0.007) (Fig. 1G). Together, these findings indicate that HCC can emerge in the GAN model without advanced fibrosis or overt systemic and biochemical features that distinguish HCC progressors from non-progressors.

### Bulk liver transcriptomics reveals immune dysregulation in adjacent non-tumor liver of HCC progressors

Non-tumor tissues adjacent to tumors are increasingly recognized as cancer-primed fields harboring molecular and microenvironmental alterations that promote or permit malignant transformation.^10^ To identify mechanisms underlying HCC development in MASH, we sequenced the bulk transcriptomes of NTB livers (n = 27) and adjacent non-tumor samples from HB livers (HB-Adj.NT, n = 6) (Fig. 1H). Pairwise comparison identified 48 differentially expressed genes (DEGs), all of which were upregulated (fold change >2, adjusted *p* <0.05) in HB-Adj.NT tissues (Fig. 1I, Table S1). These DEGs were enriched for immune-related transcripts, including the B-cell chemoattractant, *Cxcl13*, the macrophage scavenger receptor, *Marco,* myeloid activation markers *Ms4a8a*, *Fpr1, Fpr2*, and *S100a8* and myeloid chemoattractants *Ppbp*, *Ccl8*, and *Ccl12* (Fig. 1I). Despite the modest number of DEGs, KEGG and Reactome gene set overrepresentation analyses robustly identified enrichment of immune pathways, including “Cytokine-cytokine receptor interaction” and “Neutrophil degranulation” (Fig. 1J,K). Neutrophils represented the most enriched immune cell signature, suggesting their accumulation in adjacent non-tumor liver of HB mice (Fig. 1L, Table S2). These findings suggest heightened immune activation in the cancer-primed liver of HCC progressors.

To validate the heightened inflammatory state in HB mice, we next profiled 39 serum cytokines in LFD (n = 6), NTB (n = 14) and HB (n = 7) mice. Several pro-inflammatory cytokines, including IL-6, TNF, CSF1, CSF3, CXCL1, IL-2 and CCL12, were elevated in HB mice (Figs 1M and S2C). Notably, IL-6 and CSF3 promote neutrophil production and expansion,^11^ consistent with the neutrophil enrichment identified by bulk transcriptomics. CCL12, a CCR2 ligand associated with monocyte recruitment, was also among the upregulated DEGs in HB-Adj.NT liver. Together, these findings support a heightened systemic and hepatic inflammatory state in HCC progressors.

### A cross-sectional single-cell atlas of the MASH-to-HCC transition

We next performed single-cell RNA (scRNA)-sequencing of ten 60-week liver samples comprising two LFD controls, two NTB GAN mice, and three paired adjacent non-tumor (GAN-Adj.NT) and tumor (GAN-Tumor) samples from three HB mice (Fig. S3A). Histopathological evaluation of matched liver sections confirmed all tumor samples as HCC (Fig. S3B). Steatosis, lobular inflammation, MASLD activity and fibrosis scores were generally comparable between HB and NTB GAN mice (hereafter GAN) with none of the HB livers exceeding stage 2 fibrosis (Fig. S3A).

A total of 19,178 cells passed quality control, comprising 3,280 LFD, 4,153 GAN, 5,978 GAN-Adj.NT and 5,767 GAN-Tumor cells, with all samples well represented (Fig. S3C). Louvain clustering and annotation using established markers identified 14 major cell types visualized by UMAP (Fig. 2A,B). Myeloid populations included monocytes (*Ccr2*), macrophages (*Adgre1, Trem2*), Kupffer cells (*Clec4f*), neutrophils (*Cxcr2*), conventional type 1 dendritic cell (cDC1) (*Xcr1*), cDC2 (*Cd209a*), migratory DC3 (*Ccr7, Batf3*) and plasmacytoid dendritic cells (pDCs) (*Siglech*) (Figs 2B and S3D). Lymphoid populations comprised T cells (*Cd3e*), B cells (*Pax5*) and ILC1s (*Ncr1, Klrb1b*), while non-immune populations included endothelial cells (*Clec4g*), cholangiocytes (*Spp1, Krt19*) and hepatocytes (*Alb, Apoa2*). Despite their abundance in the liver, hepatocytes were poorly captured, likely due to limited viability during tissue dissociation, while hepatic stellate cells were not detected. Nevertheless, the dataset captured diverse immune and stromal populations across the MASH-to-HCC transition.

**Fig. 2:**
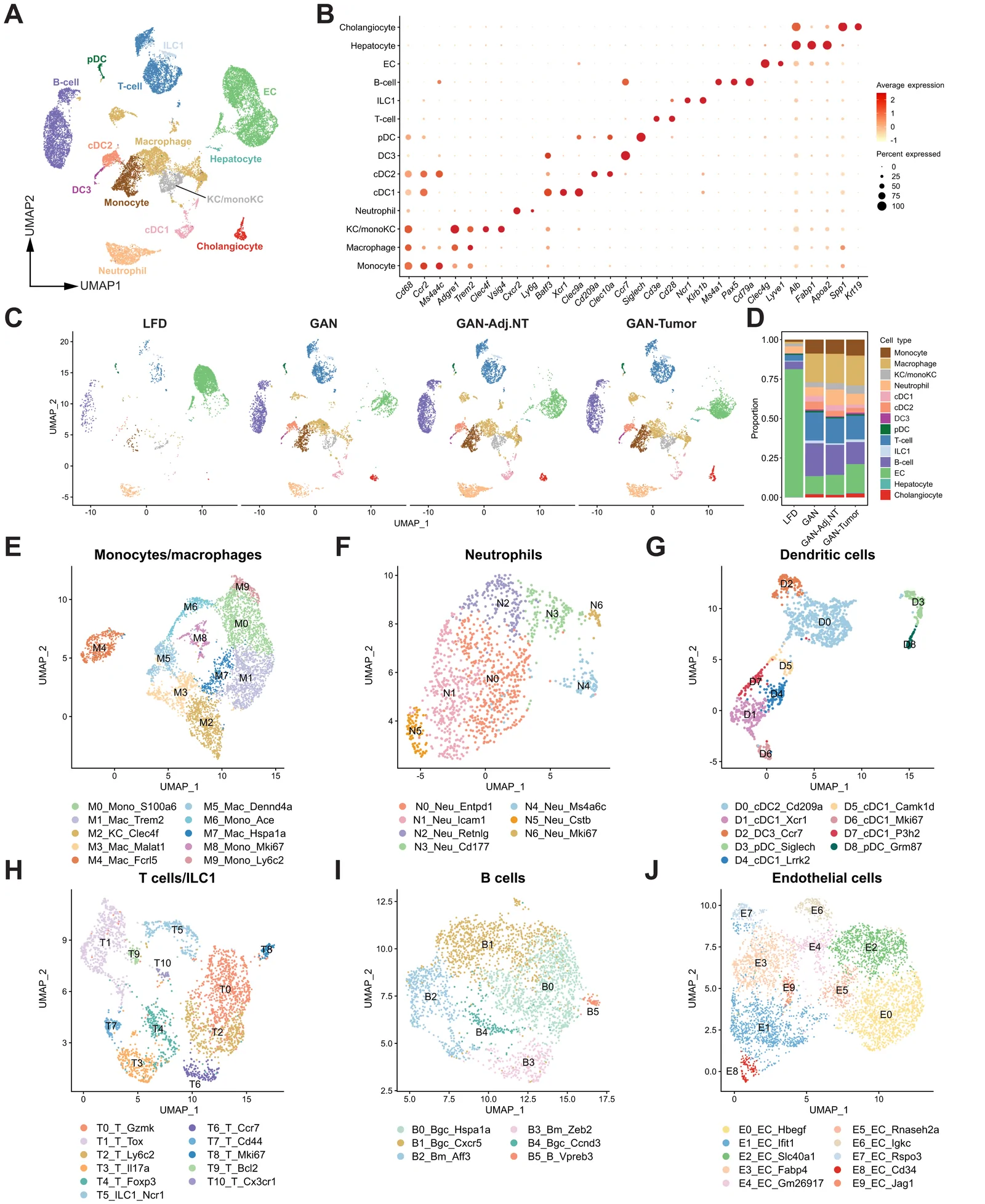
A cross-sectional single-cell atlas of the MASH-to-HCC transition. (A) UMAP plot of 19,178 cells showing broad cell types captured. (B) Dot plot of marker genes for each cell type. (C) UMAP plot separated by sample group. (D) Proportions of each cell type across sample groups. (E) UMAP plot of monocytes and macrophages after re-clustering into 10 subclusters. (F) UMAP plot of neutrophils after re-clustering into 7 subclusters. (G) UMAP plot of dendritic cells after re-clustering into 9 subclusters. (H) UMAP plot of T cells and ILC1s after re-clustering into 11 subclusters. (I) UMAP plot of B cells after re-clustering into 6 subclusters. (J) UMAP plot of ECs after re-clustering into 10 subclusters. cDC, conventional dendritic cell; pDC, plasmacytoid dendritic cell; EC, endothelial cell; GAN, Gubra-Amylin-NASH; HCC, hepatocellular carcinoma; ILC1, innate lymphoid cell type 1; KC, Kupffer cell; MASH, metabolic dysfunction-associated steatohepatitis; UMAP, uniform manifold approximation and projection.

LFD samples were dominated by ECs, which comprised >75% of LFD cells and approximately half of all ECs captured. LFD- and GAN-associated ECs occupied distinct UMAP regions, indicating substantial endothelial transcriptomic reprogramming in MASH (Figs 2C,D and S3D). In contrast, GAN-associated samples (GAN, GAN-Adj.NT and GAN-Tumor) were predominantly composed of immune cells, consistent with heightened inflammation in MASH (Figs 2D and S3D-F). Broad cell-type composition was largely similar across GAN-associated samples, except for neutrophils, which were nearly doubled in GAN-Adj.NT compared with GAN livers (9.9% *vs*. 5.6%), consistent with their enrichment in bulk transcriptomics (Figs 1L and S3G).

To resolve more subtle cellular changes across the MASH-to-HCC transition, we separately re-clustered six compartments: monocytes/macrophages, neutrophils, dendritic cells, T cells/ILC1s, B cells and ECs. Cholangiocytes and hepatocytes were excluded due to low cell numbers. This higher-resolution analysis identified 53 subclusters, which are characterized below (Fig. 2E–J).

### Depletion of *Trem2*^+^ macrophages and expansion of *Trem2*^low^ pro-inflammatory macrophages in HCC progressors

Re-clustering of monocytes and macrophages identified 10 subclusters (M0–M9; Fig. 3A) with differential enrichment across sample groups quantified by chi-square analysis (Fig. 3B,C, see supplementary methods). Four monocyte populations were identified: three classical monocyte clusters (M0, M8, M9; *Ccr2*^+^*Ly6c2*^+^) and one *Ace*^+^ patrolling monocyte cluster (M6). M8 monocytes additionally expressed proliferation-associated genes, including *Mki67*, consistent with a differentiating monocyte population. Notably, *Ly6c2*^*+*^ M9 infiltrating monocytes and M8 differentiating monocytes were strongly enriched in GAN-Adj.NT compared with GAN livers, indicating enhanced monocyte recruitment and differentiation in the cancer-primed microenvironment (Fig. 3B–D).

**Fig. 3:**
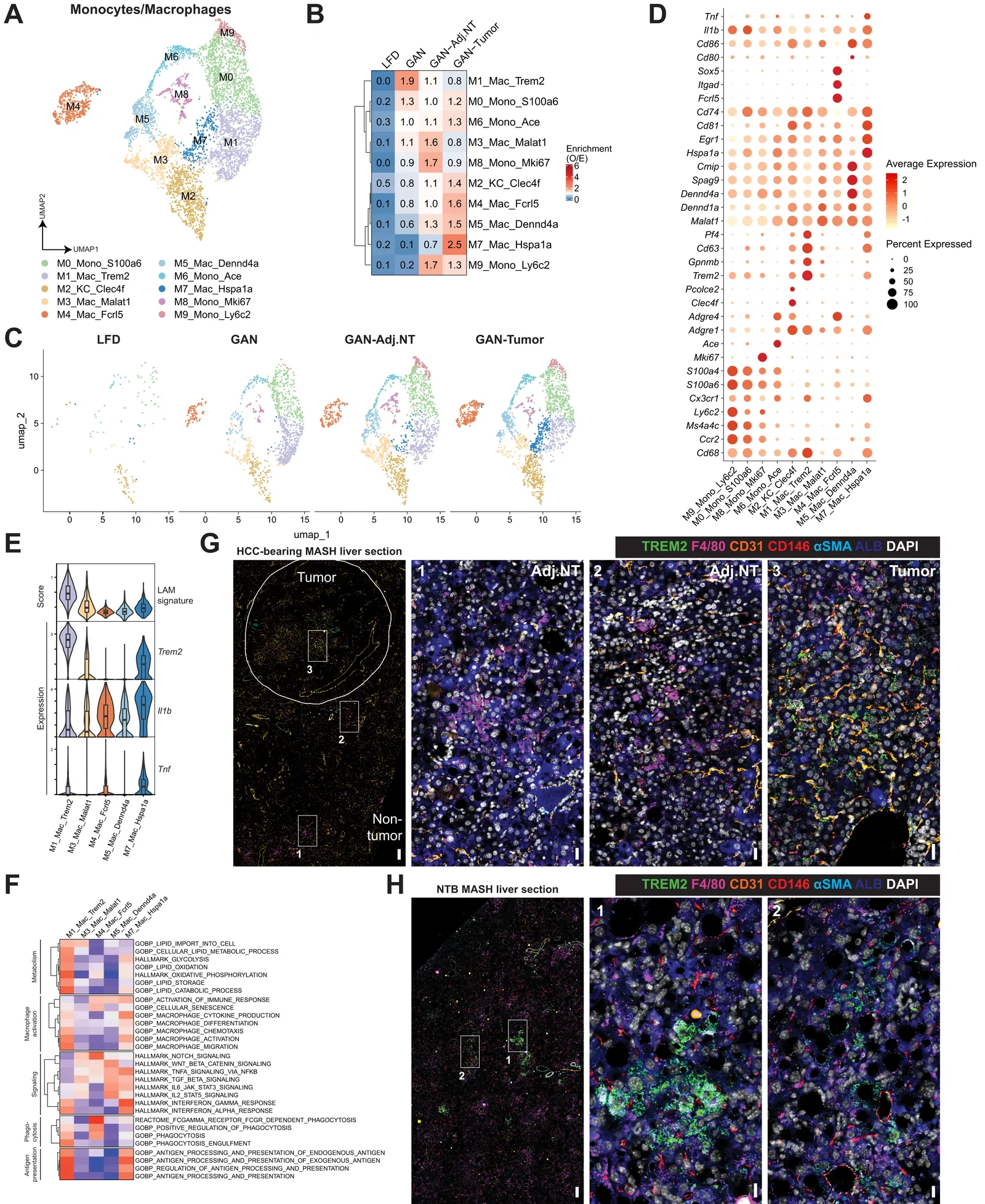
Depletion of *Trem2*^+^ macrophages and accumulation of *Trem2*^*low*^ pro-inflammatory macrophages in HCC progressors. (A) UMAP of monocytes and macrophages showing 10 subclusters. (B) Enrichment scores of monocytes and macrophages. (C) UMAP of monocytes and macrophages separated by sample group. (D) Dot plot of marker genes for monocytes and macrophages. (E) Violin plot showing the enrichment score for the LAM signature (top row) and expression levels of *Trem2, Il1b* and *Tnf* across macrophage subclusters. (F) Single-cell gene set enrichment Z-scores averaged across macrophage subclusters. (G) Multiplex immunohistochemistry of a liver section from an HCC-bearing mouse fed the GAN diet for 60 weeks. Leftmost panel: the tumor region demarcated in white; scale bar represents 200 μm. Albumin and DAPI signals were removed from the leftmost panel for enhanced clarity. Enlarged regions of interest 1, 2 and 3 are shown in the panels to the right; scale bars represent 25 μm. (H) Multiplex immunohistochemistry of a liver section from a non-tumor-bearing mouse fed the GAN diet for 60 weeks. Leftmost panel’s scale bar represents 200 μm. Albumin and DAPI signals were removed from the left-most panel for enhanced clarity. Enlarged regions of interest 1 and 2 are shown in the panels to the right; scale bars represent 25 μm. Adj.NT, adjacent non-tumor; DEG, differentially expressed gene; GAN, Gubra-Amylin-NASH; HCC, hepatocellular carcinoma; LAM, lipid-associated macrophage; UMAP, uniform manifold approximation and projection; NTB, non-tumor-bearing.

Macrophages were segregated into six subclusters. The largest, M1, expressed lipid-associated macrophage (LAM) markers (*Trem2, Gpnmb, Cd63*), identifying them as *Trem2*^*+*^ LAMs, which have reported protective functions in MASH.[12], [13] Notably, *Trem2*^+^ LAMs were enriched in GAN livers but diminished in GAN-Adj.NT and GAN-Tumor tissue, suggesting progressive depletion during HCC transition (Fig. 3B–D). M2 expressed *Clec4f* and *Pcolce2*, consistent with Kupffer cells (KCs) (Fig. 3D). Compared with LFD KCs, GAN-associated KCs showed marked loss of *Timd4* and *Cd163*, consistent with a predominantly monocyte-derived origin^14^ (Fig. S4A). *Trem2* expression was also increased in KCs from GAN and GAN-Adj.NT compared to LFD KCs, suggesting that monocyte-derived KCs may acquire LAM-like tissue-reparative functions.

The remaining macrophage subclusters (M3, M4, M5, M7) were enriched in HCC-transitioned livers (Fig. 3B). M3-M5 macrophages showed negligible *Trem2* expression and low enrichment for a 22-gene LAM signature^12^ (Table S3) and lipid metabolism pathways, leading us to annotate them as *Trem2*^*low*^ macrophages (Fig. 3E,F). Compared with M1 LAMs, M3-M5 macrophages were more differentiated and exhibited reduced metabolic, macrophage activation and antigen presentation programs, consistent with chronically stimulated macrophage states with diminished function (Figs 3F and S4B,C). Notably, M4 and M5 showed increased *Il1b* expression and enrichment of immune activation pathways, indicating a pro-inflammatory phenotype (Fig. 3E,F). Thus, HCC transition was associated with a shift from *Trem2*^+^ LAMs towards *Trem2*^low^ pro-inflammatory macrophage states.

M7 macrophages were highly enriched in tumors (Fig. 3B) and expressed *Trem2*, albeit at reduced levels compared to M1 LAMs, alongside stress response genes (*Hspa1a, Egr1*) and the pro-inflammatory cytokines *Il1b* and *Tnf* (Fig. 3D,E). They were also enriched for inflammatory Hallmark pathways, including TNFα/NFκB, IL6/JAK/STAT3 and type I/II interferon signaling (Fig. 3F), together identifying M7 as a stress-responsive, pro-inflammatory tumor-associated macrophage population.

To investigate the origins of M7 macrophages, we mapped the five macrophage populations (M1, M3, M4, M5 and M7) by diffusion analysis.^15^ M1, M7, M3 and M5 formed a transcriptional continuum, while M4 remained distinct (Fig. S4D). RNA velocity revealed strong directional vectors from M1 towards M7, suggesting a transition from *Trem2*^+^ LAMs to the M7 state (Fig. S4D). Consistent with this trajectory, *Trem2* showed negative velocity residuals in M1 and M7 macrophages, indicating progressive *Trem2* downregulation (Fig. S4E).

Compared with M1 LAMs, M7 macrophages were enriched for genes previously linked to cancer cell proliferation (*Pde4c, Cd81*)[16], [17] and immunosuppression (*Hes1, Kdm6b, Stk17b*)[18], [19], [20] in the tumor microenvironment (Fig. S4F). Thus, our analyses suggest that *Trem2*^+^ macrophages undergo a transcriptional transition within tumors, marked by loss of the LAM program and acquisition of a stress-activated phenotype with pro-inflammatory, tumor-promoting and immunosuppressive features.

Given the progressive loss of *Trem2*^+^ LAMs and accumulation of *Trem2*^low^ macrophage states identified by scRNA-sequencing, we next examined their spatial organization by multiplex immunohistochemistry. Murine liver tissues were stained for macrophage/LAM markers (TREM2, F4/80), endothelial markers (CD31, CD146), hepatocytes (ALB) and activated myofibroblast/smooth muscle cell marker (α-SMA). TREM2 antibody specificity was validated in CRISPR/Cas9-engineered *Trem2*-knockout RAW264.7 macrophages (Fig. S4G). In HCC-transitioned MASH liver, F4/80^+^TREM2^low^ macrophages predominated in the adjacent non-tumor regions (Fig. 3G, regions 1 and 2). In contrast, F4/80^+^ TREM2^+^ macrophages were enriched within the tumor core and closely associated with endothelial and myofibroblast stromal networks (Fig. 3G, region 3), suggesting potential crosstalk with endothelial cells and fibroblasts within the tumor microenvironment. Whether the F4/80^+^TREM2^+^ macrophages observed within tumors correspond to M1 LAMs, *Trem2*^+^ KCs (M2), or M7 macrophages remains unclear. In contrast, distinct clusters of F4/80^+^ TREM2^+^ macrophages were abundant throughout the liver parenchyma of the NTB GAN liver, though F4/80^+^ TREM2^low^ macrophages were also present and uniformly distributed (Fig. 3H, regions 1 and 2). Some F4/80^+^ TREM2^+^ macrophages formed crown-like structures around lipid-laden hepatocytes or residual lipid droplets, consistent with macrophage-mediated clearance of damaged hepatocytes. Thus, these spatial findings corroborate our scRNA-sequencing data, supporting a shift from reparative *Trem2*^+^ LAMs towards *Trem2*^low^ macrophage states within the cancer-primed Adj.NT microenvironment.

### Enrichment of immature neutrophils in adjacent non-tumor regions of HCC-transitioned livers

Subclustering of neutrophils identified seven populations (N0–N6; Fig. 4A), of which N2, N3, N4 and N6 were preferentially enriched in GAN-Adj.NT compared with GAN livers, with N3 showing the strongest enrichment (Fig. 4B,C). N3 and N6 highly expressed *Cd177, Ngp, Camp* and *Ltf*, markers of immature bone marrow neutrophils^21^ (Fig. 4D). N6 additionally expressed the proliferation marker *Mki67*, consistent with a cycling population at an earlier stage of neutrophil differentiation than N3.

**Fig. 4:**
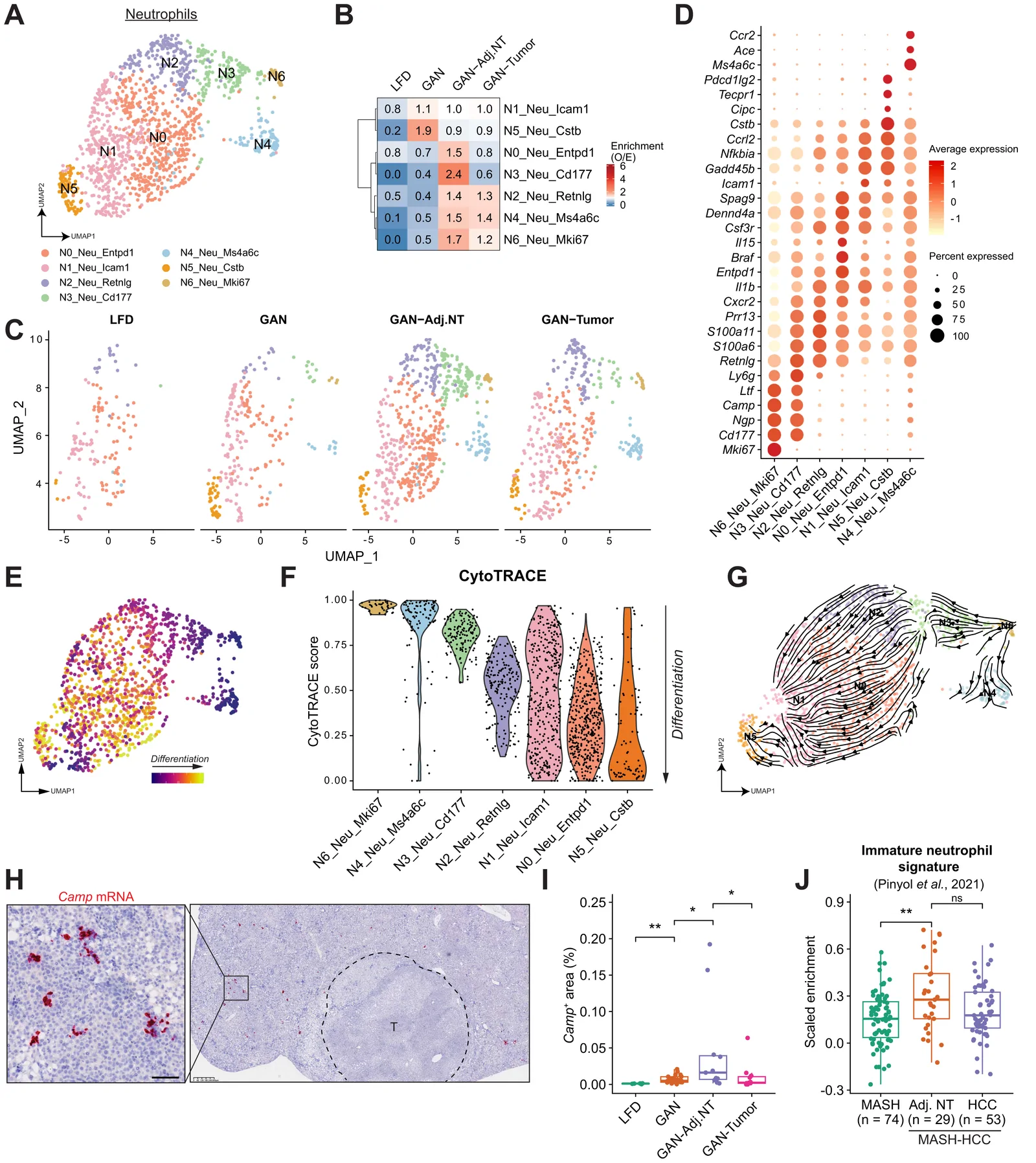
Enrichment of immature neutrophils in adjacent non-tumor regions of HCC-transitioned livers. (A) UMAP of neutrophils showing 7 subclusters. (B) Enrichment scores of neutrophil subclusters. (C) UMAP of neutrophils separated by sample group. (D) Dot plot of marker genes for neutrophils. (E) UMAP showing CytoTRACE scores of neutrophils used to predict degree of differentiation. (F) Violin plot of CytoTRACE scores across neutrophil subclusters. (G) CellRank2 trajectory analysis integrating RNA velocity and gene similarity of neutrophils. (H) Representative image of an HCC-bearing liver section stained for *Camp* mRNA by RNAscope. Scale bars of left and right panels represent 100 μm and 300 μm, respectively. (I) Quantification of percentage of *Camp*^+^ area in liver sections. Wilcoxon signed-rank test. (J) Gene set enrichment scores for the 5-gene immature neutrophil signature in a human MASH-HCC cohort. Wilcoxon signed-rank test. HCC, hepatocellular carcinoma; MASH, metabolic dysfunction-associated steatohepatitis; UMAP, uniform manifold approximation and projection.

To validate the immature states of N6 and N3 neutrophils, we mapped our cells onto a published single-cell dataset spanning neutrophil development across murine bone marrow, peripheral blood, spleen and pancreatic tumors.^22^ N6 overlapped predominantly with bone marrow-derived preNeu cells, whereas N3 mapped to the immature IMM-1, IMM-2 and MAT1 populations (Fig. S5A–C). CytoTRACE and RNA velocity-based CellRank2 trajectory inference further supported their immature states, with N6 predicted as an initial state transitioning towards N3 (Figs 4E-G and S5D,E). Notably, a subset of N3 cells showed minimal RNA velocity and was predicted by CellRank2 as a terminal state (Figs 4G and S5F), suggesting that N3 represents a stable immature neutrophil state that accumulates in GAN-Adj.NT liver.

We further validated this finding by *in situ* hybridisation of *Camp* mRNA, a marker of immature neutrophils, which revealed significant enrichment of *Camp*^+^ clusters in adjacent non-tumor regions of HB livers (Fig. 4H,I). To assess whether immature neutrophils similarly accumulate in human MASH-HCC, we derived a stringent eight-gene signature specific to N3/N6 neutrophils, of which five genes have human orthologues (*CAMP, CD177, GPR27, ITGB2, LTF*; Fig. S5G). In a human MASH/MASH-HCC transcriptomic cohort,^23^ this signature was markedly enriched in adjacent non-tumor tissues from MASH-HCC compared with HCC-naïve MASH liver (Fig. 4J), supporting conservation in human disease. As immature neutrophils are normally restricted to the bone marrow but can enter circulation during inflammation-driven emergency granulopoiesis,^11^ their accumulation in GAN-Adj.NT liver further supports a hyperinflammatory microenvironment associated with HCC progression.

N4 neutrophils, also enriched in HCC-transitioned livers (Fig. 4B), co-expressed neutrophil (*Cxcr2, Csf3r*) and monocyte (*Ms4a6c, Ace, Ccr2*) markers (Fig. 4D), resembling previously described neutrophil-like monocytes.^24^ These cells emerge during severe inflammatory conditions, including tissue injury and lipopolysaccharide exposure, through emergency monopoiesis. Their enrichment in HCC-transitioned livers therefore provides further evidence of a heightened inflammatory state.

Given the marked enrichment of immature neutrophils in GAN-Adj.NT liver, we tested whether immature neutrophils directly promote HCC development by treating LFD and GAN mice from 48 weeks with six fortnightly doses of G-CSF/anti-G-CSF complexes (G-CSFcx) to induce immature neutrophil expansion and mobilization^25^ (Fig. S5H). Despite significant accumulation of immature neutrophils (Cd101^-^Ly6G^+^Cd11b^+^) in peripheral blood and liver (Fig. S5I and J), G-CSFcx treatment did not alter tumor incidence or burden (Fig. S5L,M), indicating that immature neutrophil accumulation alone is insufficient to promote HCC. Nevertheless, the enrichment of immature neutrophils and neutrophil-like monocytes in HCC-transitioned liver strongly reflects heightened emergency granulopoiesis and monopoiesis associated with the hyperinflammatory state of HCC progressors.

### Enhanced stress-induced activation of cDC1 and cDC2 in HCC progressors

Subclustering identified nine dendritic cell populations, comprising five cDC1 (D1, D4–D7; *Xcr1, Batf3, Irf8*), one cDC2 (D0; *Cd209a, Clec10a*), one migratory DC3 (D2; *Ccr7, Mreg, Fscn1*) and two pDC (*Siglech*; D3, D8) subclusters (Fig. 5A,D). Notably, cDC1 composition shifted from D7 enrichment in GAN liver to D4 enrichment in GAN-Adj.NT liver (Fig. 5B,C). Compared with D7, D4 cDC1s exhibited 195 upregulated DEGs, including immediate-early (*Fosb, Junb, Egr1*) and heat-shock (*Hsp90ab1, Hspa1a*) genes, alongside reduced *Xcr1* expression (Fig. 5D,E; Table S4). Pathway analysis further revealed enrichment of stress-response and immune-activation programs, including IL-17 signaling (Fig. 5F), consistent with a more activated, stress-responsive cDC1 state in GAN-Adj.NT liver.

**Fig. 5:**
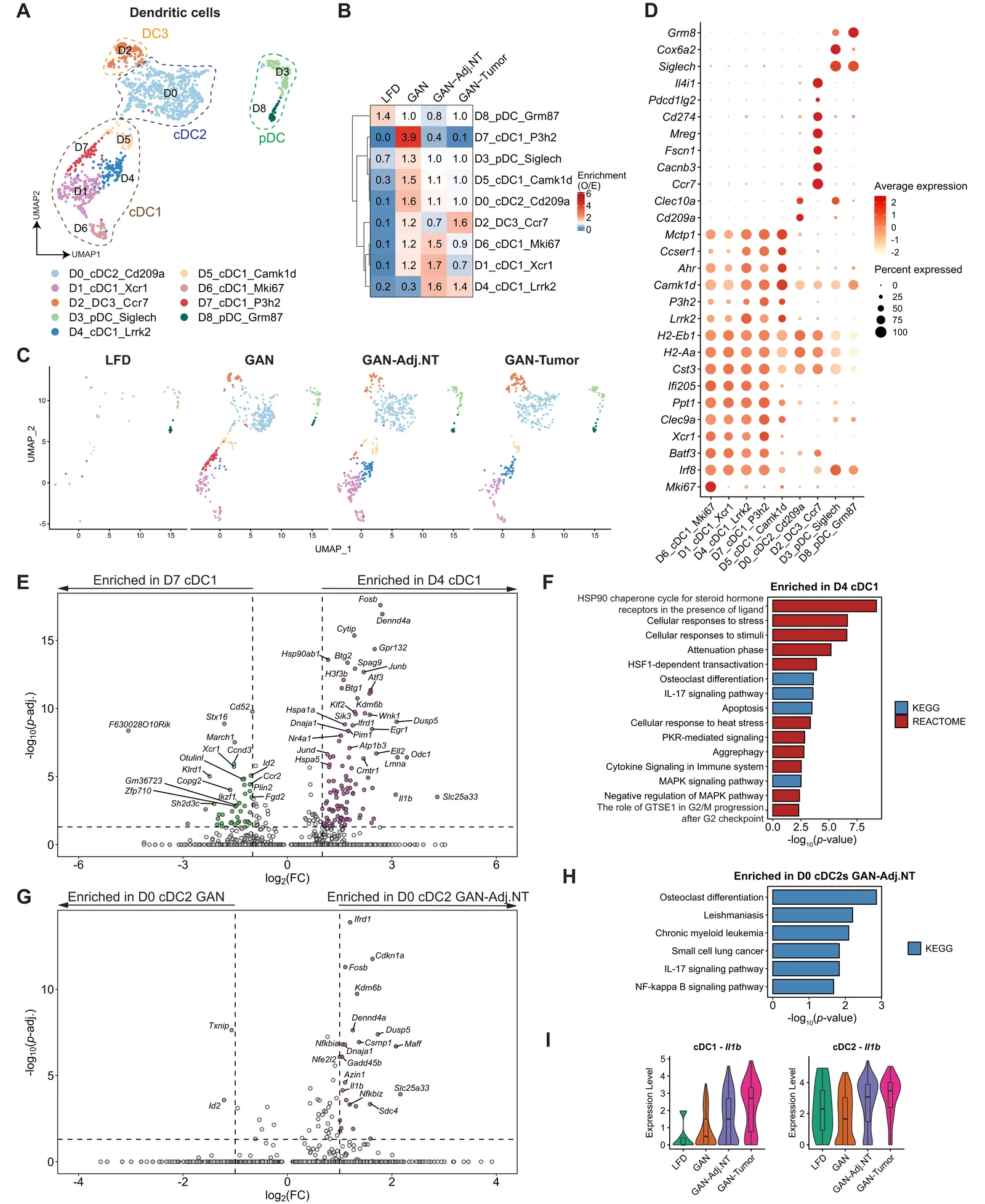
Enhanced stress-induced activation of cDC1 and cDC2 in HCC progressors. (A) UMAP of dendritic cells showing 9 subclusters, comprising cDC1, cDC2, DC3 and pDC subsets. (B) Enrichment scores of dendritic cell subclusters. (C) UMAP of dendritic cells separated by sample group. (D) Dot plot of marker genes for dendritic cells. (E) Differential expression analysis of D4 cDC1s (enriched in GAN-Adj.NT tissues) *vs*. D7 cDC1s (enriched in GAN tissues). BH-adjusted *p* values. (F) KEGG and Reactome gene set enrichment of DEGs enriched in D4 cDC1s. Top 15 gene sets are shown. *p* values calculated using the g:SCS algorithm of gProfiler2. (G) Differential expression analysis of D0 cDC2 cells in GAN-Adj.NT tissues *vs*. GAN counterparts. BH-adjusted *p* values. (H) KEGG and Reactome gene set enrichment of DEGs enriched in GAN-Adj.NT D0 cDC2s. Only KEGG gene sets showed significant enrichment. *p* values calculated using the g:SCS algorithm of gProfiler2. (I) Expression of *Il1b* in cDC1 (D1, D4, D5, D6, D7) and cDC2 (D0) cells across sample groups. Adj.NT, adjacent non-tumor; cDC1, conventional dendritic cell type 1; cDC2, conventional dendritic cell type 2; DC3, dendritic cell 3; DEG, differentially expressed gene; GAN, Gubra-Amylin-NASH; HCC, hepatocellular carcinoma; pDC, plasmacytoid dendritic cell; UMAP, uniform manifold approximation and projection; BH, Benjamini-Hochberg.

Trajectory analysis indicated that D4 and D7 cDC1s arise from D1 and converge on D5 (Fig. S6A). D5 displayed greater differentiation by CytoTRACE and reduced *Xcr1*, *Batf3* and MHC class II expression, consistent with a terminally differentiated, post-stimulated state (Figs 5D and S6B,C). Conversely, D1 was metabolically active and enriched for antigen processing and presentation pathways, consistent with a pre-stimulated state (Fig. S6D). Thus, GAN-Adj.NT cDC1s follow a distinct trajectory through an activated, pro-inflammatory D4 intermediate before converging on the shared D5 terminal state.

Differential expression analysis of cDC2s (D0) similarly revealed increased activation and stress responses in GAN-Adj.NT compared with GAN cells, including upregulation of immediate-early (*Fosb, Gadd45b, Dnaja1*), NFκB-associated (*Il1b, Nfkbia*) and oxidative stress-response (*Nfe2l2, Maff*) genes (Fig. 5G,H, Table S5). Notably, *Il1b* expression was elevated in both cDC1 and cDC2 populations from GAN-Adj.NT and GAN-Tumor tissues compared with GAN liver (Fig. 5I). Together, these findings reveal activated, pro-inflammatory dendritic cell states associated with HCC progression.

### Increased Th17/Treg ratio and dysfunctional T cells in livers of HCC progressors

Subclustering of T cells and ILC1s identified 10 T-cell populations (T0–T4, T6–T10) and one ILC1 cluster (T5; *Ncr1, Klrb1b, Eomes*^low^) (Fig. 6A,D). T-cell populations included four CD8+ cytotoxic T lymphocyte (CTL) clusters (T0, T2, T8, T10; *Cd8b1, Ccl5, Nkg7*), T regulatory cells (Treg) (T4; *Foxp3*), T helper 17 (Th17) cells (T3; *Il17a, Rorc*) and naïve T cells (T6; *Tcf7, Lef1, Sell*) (Fig. 6A,D).

**Fig. 6:**
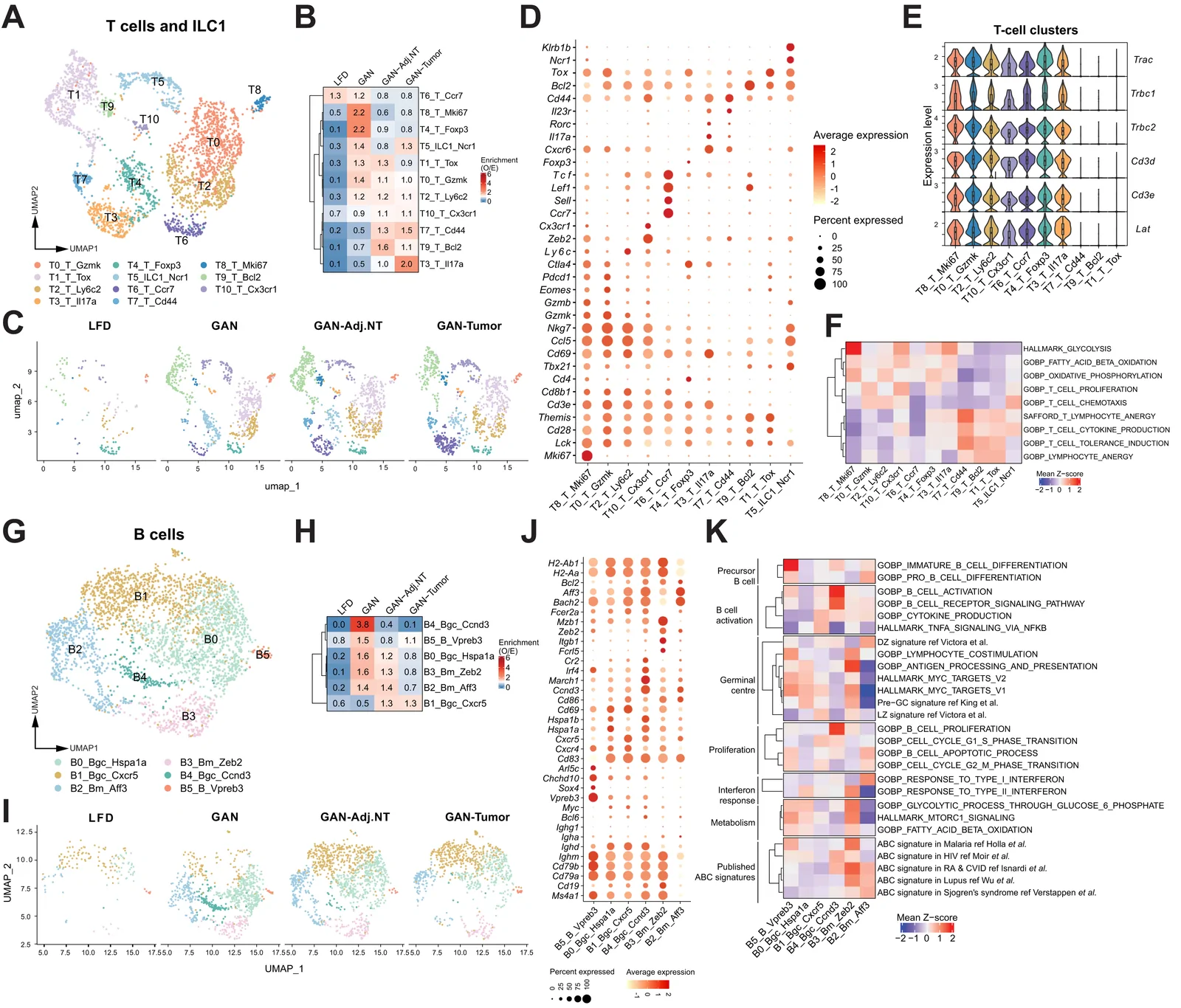
Increased Th17/Treg ratio and loss of dark zone germinal center B cells in livers of HCC progressors. (A) UMAP of T cells and ILC1 revealing 11 subclusters comprising 10 T-cell and 1 ILC1 subclusters. (B) Enrichment scores of T-cell and ILC1 subclusters. (C) UMAP of T cells and ILC1 separated by sample group. (D) Dot plot of marker genes for T cells and ILC1. (E) Expression of genes involved in T-cell receptor signaling across T-cell subclusters. (F) Single-cell gene set enrichment Z-scores of gene sets across T-cell and ILC1 subclusters. (G) UMAP of B cells revealing 6 subclusters. (H) Enrichment scores of B-cell subclusters. (I) UMAP of B cells separated by sample group. (J) Dot plot of marker genes for B cells. (K) Single-cell gene set enrichment Z-scores of gene sets across B cell subclusters. HCC, hepatocellular carcinoma; ILC1, innate lymphoid cell type 1; Th17, T helper 17; Treg, T regulatory cell; UMAP, uniform manifold approximation and projection.

Tregs were enriched in HCC-naïve GAN livers but markedly reduced in GAN-Adj.NT and GAN-Tumor tissues, accompanied by progressive Th17 enrichment (Fig. 6B,C). This shift towards an increased Th17/Treg balance is characteristic of pro-inflammatory immune states and further supports a hyperinflammatory microenvironment associated with HCC progression.

Three T-cell subclusters (T1, T7, and T9) displayed reduced expression of T-cell receptor signaling genes (*Trac, Trbc1, Trbc2, Cd3d, Cd3e, Lat*) (Fig. 6E) and enrichment of anergy-associated signatures, consistent with hyporesponsive states (Fig. 6E,F). T7 and T9 were enriched in GAN-Adj.NT compared to GAN livers, while T1, which expressed the exhaustion-associated gene *Tox*,^26^ was similarly abundant across both groups (Fig. 6B). CytoTRACE and CellRank2 further indicated that these were highly differentiated states, with T7 and T9 arising from Th17 (T3) and CTL (T10) populations, respectively (Fig. S7A–C). Together, these findings suggest accumulation of differentiated, dysfunctional T-cell states associated with chronic stimulation,^27^ further supporting a hyperinflammatory yet functionally dysregulated immune landscape in HCC progressors.

### Loss of dark-zone germinal center B cells in HCC progressors and atypical B cells in MASH

B cells were re-clustered into six subclusters comprising three germinal center B-cell (Bgc) populations (B0, B1, B4; *Bcl6, Myc*), two atypical memory B-cell populations (B2, B3) and one immature B-cell population (B5; *Vpreb3, Sox4, Cd83*^*low*^) (Fig. 6G,J). B4 Bgc were enriched in GAN livers, whereas B1 Bgc predominated in GAN-Adj.NT and GAN-Tumor tissues, indicating divergent Bgc phenotypes between HCC-naïve and HCC-progressed liver (Fig. 6H,I). Gene signature analysis further resolved distinct Bgc states (Fig. 6K, Table S6). B0 resembled antigen-experienced pre-germinal center B cells, whereas B1 exhibited light-zone germinal center features, including *Cxcr5*, *Cd69* and *Cd86* expression and enrichment of TNFα/NFκB and cytokine production pathways (Fig. 6J–K).[28], [29]

In contrast, B4 showed dark-zone germinal center characteristics, with enriched B-cell activation, B-cell receptor signaling and proliferation programs, high *Ccnd3* and *Irf4* expression, and reduced MYC target activity, consistent with late-stage dark-zone Bgc transitioning towards plasma cell differentiation.[30], [31] The depletion of B4 dark-zone Bgc in GAN-Adj.NT therefore suggests impaired germinal center function and a compromised humoral response during HCC progression.

Interestingly, two atypical B-cell subclusters (B2 and B3) were similarly enriched in GAN and GAN-Adj.NT livers, suggesting their emergence during MASH progression (Fig. 6H–J). B3 expressed *Zeb2, Cd19, Cd86, Fcrl5* and *Itgb1*, while lacking *Ighd, Fcer2a, Cr2* and *Bach2*, resembling age-associated B cells (ABCs) described in autoimmune and infectious diseases.[32], [33], [34] Consistently, B3 was enriched for multiple ABC signatures (Fig. 6K) and expressed *Mzb1* (Fig. 6J). B3 also showed enrichment of mTORC1 signaling, fatty acid oxidation, antigen processing/presentation and type II interferon-response programs (Fig. 6K). As ABCs are autoreactive memory B cells with the capacity to generate autoantibody-producing cells,^35^ their identification here suggests a previously unrecognized B-cell state in MASH.

In contrast, B2 displayed memory B-cell features, including increased *Bcl2* and reduced MHC class II genes (*H2-Aa, H2-Ab1*), but lacked key B-cell receptor signaling components (*Cd19, Cd79a, Cd79b*), consistent with an anergic phenotype (Fig. 6J).^36^ B2 cells were also terminally differentiated (Fig. S7D–F), metabolically less active and enriched for type I interferon-response programs, resembling atypical memory B cells described in chronic viral infection.^37^ Thus, distinct atypical memory B-cell states associated with autoimmune and chronic infectious diseases also emerge during MASH progression.

### Augmented endothelial cell activation in livers of HCC progressors and fetal-like EC reprogramming in MASH-HCC tumors

Re-clustering of ECs identified ten subclusters with distinct tissue distributions (Fig. 7A–C). E0, E2 and E5 expressed canonical LSEC markers (*Clec4g, Hpgd, Stab1*) and were almost exclusively derived from LFD liver, whereas E3, characterized by lipid metabolism genes (*Fabp4, Fabp5, Cd36*), was restricted to GAN-associated tissues (Fig. 7B–D). Accordingly, GAN-associated ECs showed increased lipid metabolism, particularly lipid import and storage (Fig. 7E,F), together with enrichment of extracellular matrix organization and cell adhesion programs compared with LFD ECs (Fig. S8A and B, Table S7). Importantly, ECs from GAN-Adj.NT and GAN-Tumor tissues were enriched for pro-inflammatory programs, including IL-6, type I/II interferon and TNFα signaling (Fig. 7F). Consistently, GAN-Adj.NT ECs upregulated the alarmin genes *S100a8/S100a9* and interferon-response genes *Isg15/Ifit1* compared with GAN ECs (Figs 7E and S8C, Table S8), indicating heightened endothelial inflammatory activation in the cancer-primed microenvironment.

**Fig. 7:**
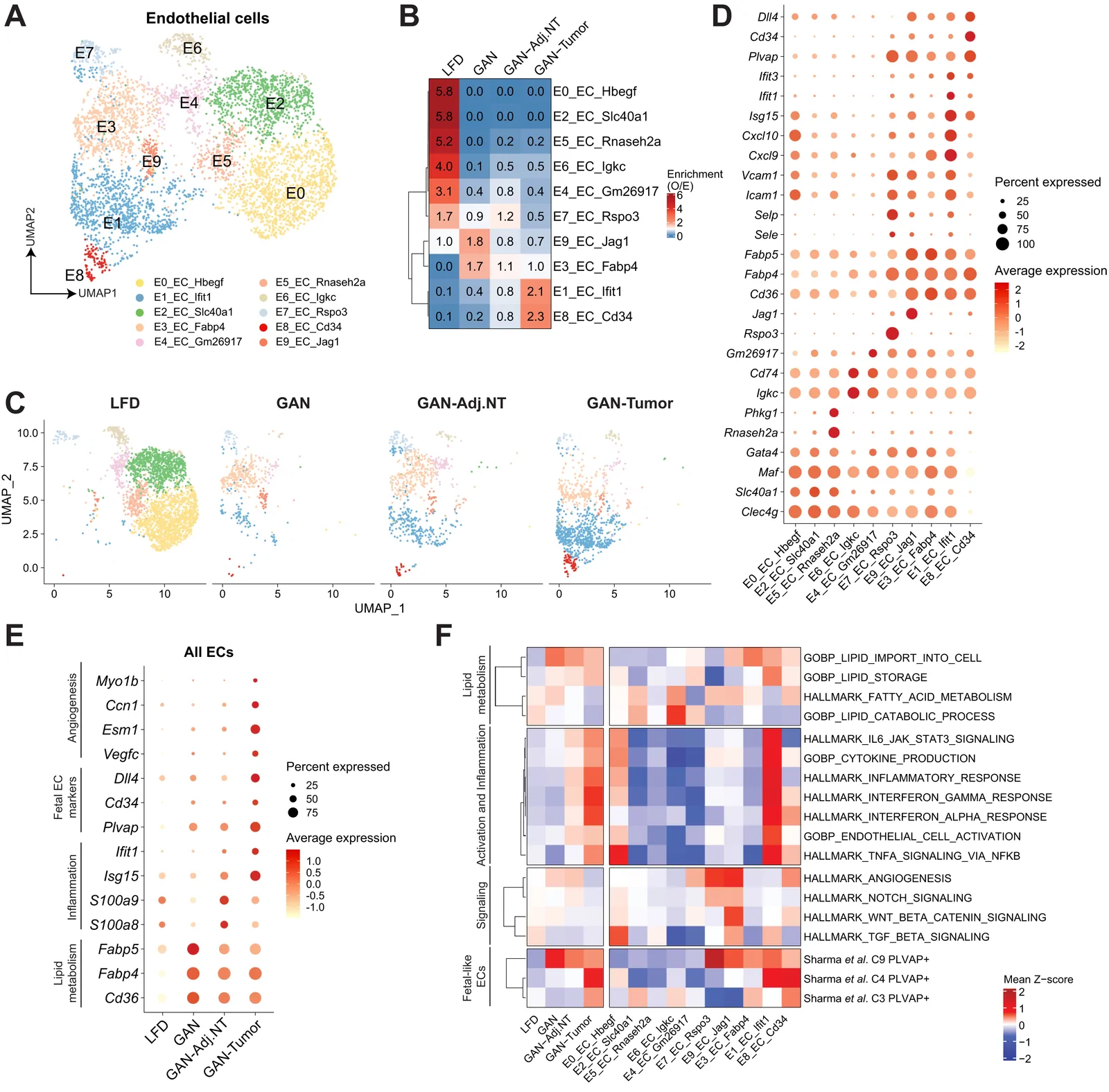
Augmented endothelial cell activation in livers of HCC progressors and emergence of fetal-like EC subsets in HCCs. (A) UMAP of ECs revealing 10 subclusters. (B) Enrichment scores of EC subclusters. (C) UMAP of ECs separated by sample group. (D) Dot plot of marker genes for ECs. (E) Expression of chosen genes across all ECs within each sample group. (F) Single-cell gene set enrichment Z-scores of gene sets across all ECs separated by sample group (heatmap, left) and across EC subclusters (heatmap, right). EC, endothelial cell; HCC, hepatocellular carcinoma; UMAP, uniform manifold approximation and projection.

E1 and E8 ECs were strongly enriched in GAN-Tumor tissue and present at lower abundance in GAN-Adj.NT liver (Fig. 7B,C). E1 exhibited an activated, pro-inflammatory phenotype, with enrichment of interferon-response programs and expression of *Cxcl9, Cxcl10, Isg15, Ifit1* and *Ifit3* (Fig. 7D,F). E8 expressed the fetal LSEC-associated gene *Plvap* and *Cd34*, while showing reduced LSEC identity markers (*Clec4g, Maf, Gata4*), consistent with a fetal-like phenotype. Notably, PLVAP^+^ “oncofetal” ECs have been described in human HCC and implicated in DLL4/NOTCH2-mediated immunosuppression.^38^ Indeed, E1 and E8 were enriched for published PLVAP^+^ oncofetal EC signatures,^38^ supporting their oncofetal identities (Fig. 7F; Table S9). GAN-Tumor ECs also upregulated pro-angiogenic factors (*Vegfc, Esm1, Ccn1, Myo1b*) compared with GAN-Adj.NT ECs (Fig. 7E), several of which were enriched in E1/E8 (Fig. S8D). Altogether, ECs undergo metabolic and extracellular matrix remodeling in MASH, acquire heightened inflammatory states in HCC progressors, and develop tumor-enriched oncofetal phenotypes resembling those observed in human HCC.

### Endothelial-immune cell-cell communication and a myeloid-driven IL-1β signaling network underlie the hyperinflammatory phenotype in HCC progressors

Our analyses revealed 53 distinct immune and non-parenchymal subclusters with differential enrichment across MASH-to-HCC progression (Fig. S9A). To identify signaling pathways underlying the hyperinflammatory phenotype of HCC progressors, we used CellChat^39^ to infer ligand–receptor (LR) communication between these subclusters, focusing on GAN *vs*. GAN-Adj.NT tissues.

After excluding subclusters with <10 cells in either group, GAN-Adj.NT tissue showed a marked increase in predicted LR interactions, particularly from EC subclusters (E1, E3, E4, E7, E9) towards neutrophils (N0–N5), dendritic cells (D0, D2, D8) and T cells (T2, T3, T7) (Fig. 8A). These senders included pro-inflammatory E1, peri-central E7 (*Rspo3*) and peri-portal E9 (*Jag1*) ECs, suggesting widespread endothelial–immune crosstalk across vascular niches in GAN-Adj.NT tissue.

**Fig. 8:**
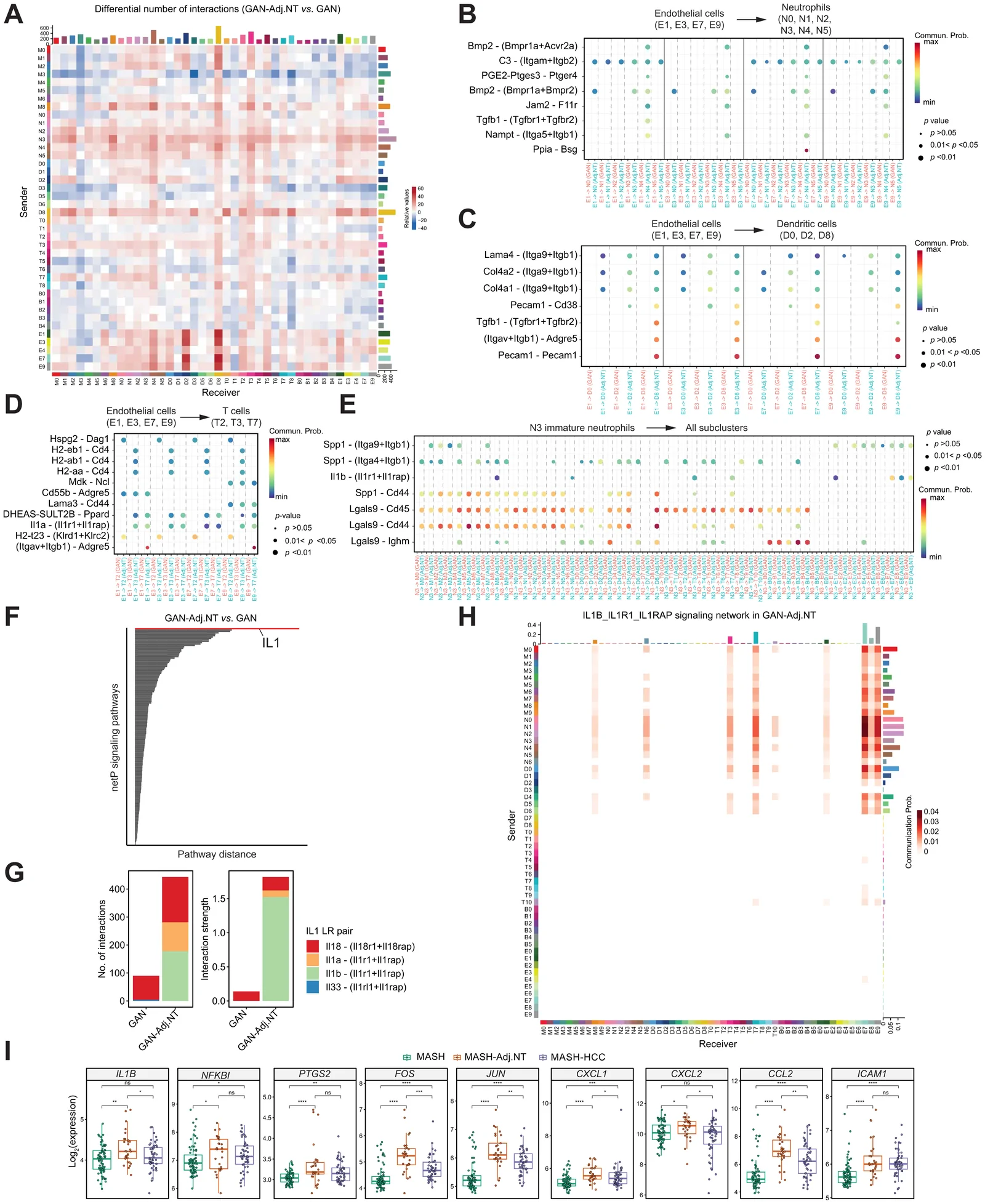
Endothelial-immune cell-cell communication and a myeloid-driven IL-1β signaling network underlie the hyperinflammatory phenotype in HCC progressors. (A) Differential number of interactions between subclusters in the GAN-Adj.NT and GAN cell-cell communication networks. Red indicates an increase in the number of interactions in GAN-Adj.NT, while blue indicates an increase in the GAN network. (B) Top ligand–receptor interactions between endothelial cells and neutrophils uniquely identified in GAN-Adj.NT but not in the overall GAN network. (C) Top ligand–receptor interactions between endothelial cells and dendritic cells uniquely identified in GAN-Adj.NT but not in the overall GAN network. (D) Top ligand–receptor interactions between endothelial cells and T cells uniquely identified in GAN-Adj.NT but not in the overall GAN network. (E) Top ligand–receptor interactions between N3 immature neutrophils and all subclusters uniquely identified in GAN-Adj.NT but not in the overall GAN network. (F) Joint manifold learning and classification analysis of the inferred communication networks based on functional similarity by CellChat comparing signaling networks between GAN-Adj.NT and GAN subclusters. (G) Number of interactions (left) and interaction strengths (right) of IL-1 LR pairs among GAN-Adj.NT and GAN subclusters. (H) Communication probabilities of Il1b-(Il1r1+Il1rap) signaling between senders (vertical axis) and receivers (horizontal axis) in the GAN-Adj.NT network, as predicted by CellChat. (I) Log_2_-normalised expression of indicated genes in liver tissues from a human cohort of non-cirrhotic MASH and MASH-HCC patients. Wilcoxon signed-rank test. Adj.NT, adjacent non-tumor; GAN, Gubra-Amylin-NASH; HCC, hepatocellular carcinoma; LR, ligand-receptor; MASH, metabolic dysfunction-associated steatohepatitis.

Among EC–neutrophil interactions uniquely enriched in GAN-Adj.NT tissue, CellChat identified LR pairs involved in leukocyte adhesion and migration, including *C3–(Itgam+Itgb2)* and *Jam2–F11r*, consistent with enhanced neutrophil recruitment (Fig. 8B). Increased *Bmp2* signaling was also predicted from ECs to mature (N0), immature (N3) and monocyte-like (N4) neutrophils.

EC communication with cDC2s (D0), DC3s (D2) and pDCs (D8) was similarly increased in GAN-Adj.NT tissue (Fig. 8C), predominantly through ECM–integrin interactions, including collagen IV and laminin (*Col4a1, Col4a2, Lama4*) signaling to α9β1 integrins (*Itga9, Itgb1*). These interactions suggest enhanced endothelial support for dendritic cell recruitment and retention, potentially amplifying local immune responses.

EC communication with *Ly6c2*^+^ CTLs (T2), Th17 cells (T3) and dysfunctional Th17-derived T cells (T7) was also increased in GAN-Adj.NT tissue (Fig. 8D). Predicted MHC class II–*Cd4* and *Il1a–(Il1r1+Il1rap)* interactions with Th17 cells suggest enhanced antigen presentation and IL-1-driven Th17 activation,^40^ while ECM interactions (*Lama3, Hspg2*) with Ly6c2^+^ CTLs may promote their recruitment and retention. These findings support enhanced endothelial–T-cell crosstalk within the inflammatory GAN-Adj.NT niche.

N3 immature neutrophils, which were strongly enriched in GAN-Adj.NT livers, also showed increased predicted communication with multiple immune populations. *Lgals9* and *Spp1* emerged as dominant N3-derived signals, particularly through *Lgals9–Cd44* and *Spp1–Cd44/integrin* interactions associated with immune-cell recruitment, adhesion and retention (Fig. 8E). These findings suggest that N3 neutrophils may contribute to an inflammatory, pro-tumorigenic niche despite being insufficient to independently promote HCC. Collectively, CellChat analysis revealed extensive rewiring of endothelial–immune and neutrophil–immune communication in GAN-Adj.NT livers, consistent with enhanced inflammatory cell recruitment, retention and intercellular crosstalk.

To unbiasedly identify signaling pathways most altered in GAN-Adj.NT tissue, we performed joint manifold learning of CellChat communication networks based on functional similarity across conditions. IL-1 signaling emerged as the most prominently altered pathway between GAN and GAN-Adj.NT tissues (Fig. 8F). Within this pathway, *Il1b–(Il1r1+Il1rap)* showed the greatest increase in both interaction number and strength in GAN-Adj.NT livers, while no significant interactions were detected in GAN livers (Fig. 8G). Consistently, pro-IL-1β protein was increased in Adj.NT liver from HB mice compared with NTB GAN mice, supporting increased IL-1β production (Fig. S9B).

Analysis of the *Il1b–(Il1r1+Il1rap)* network identified myeloid cells, including monocytes/macrophages, cDC1/2s and neutrophils, as the major IL-1β senders in GAN-Adj.NT livers (Fig. 8H). Dominant receivers included pro-inflammatory (E1), fetal-like (E8), peri-central (E7) and peri-portal (E9) ECs, as well as Th17 cells (T3), proliferating monocytes (M8) and immature proliferating neutrophils (N6) (Fig. 8H). These findings identify myeloid-derived IL-1β as a prominent signaling axis in HCC-transitioned MASH liver, potentially reinforcing endothelial activation, leukocyte recruitment and Th17 inflammation in a feed-forward hyperinflammatory microenvironment.

### IL-1β-associated hyperinflammatory reprogramming is recapitulated in human MASH-HCC

To assess the relevance of our findings to human disease, we analyzed liver transcriptomic data from non-cirrhotic patients with MASH and MASH-HCC.^23^ Indeed, *IL1B* expression was significantly elevated in MASH-HCC adjacent non-tumor tissue, together with canonical IL-1β-responsive genes (*NFKBIA, PTGS2, FOS, JUN, CXCL1, CXCL2, CCL2, ICAM1*), consistent with activation of NFκB and immediate-early inflammatory programs (Fig. 8I). Single-sample GSEA further revealed enrichment of “IL-1β production”, “Response to IL-1”, “Inflammatory Response”, “TNFα signaling via NFκB” and “IL-6/JAK/STAT3 signaling” pathways in adjacent non-tumor compared with MASH-only liver (Fig. S9C). Together, these findings support the presence of a heightened IL-1β-associated inflammatory program in human non-cirrhotic MASH-HCC adjacent non-tumor liver, mirroring our observations in mice.

## Discussion

In this study, we present a high-resolution, cross-sectional single-cell atlas of immune and stromal remodeling across HCC-naïve and HCC-progressed MASH liver. Our analyses reveal coordinated changes across multiple cellular compartments that converge on a hyperinflammatory microenvironment in the adjacent non-tumor liver of HCC progressors. These included depletion of *Trem2*^+^ LAMs with expansion of *Trem2*^*low*^ pro-inflammatory macrophages, accumulation of immature neutrophils, stress-responsive activation of cDC1 and cDC2 populations, increased Th17/Treg balance, loss of dark-zone germinal center B cells, and inflammatory endothelial activation. Cell–cell communication analysis further identified myeloid-derived IL-1β signaling as a prominent axis potentially linking these cellular changes. Elevated circulating pro-inflammatory cytokines further indicated that this hyperinflammatory phenotype extends beyond the liver.

Importantly, human non-cirrhotic MASH-HCC adjacent non-tumor tissues similarly exhibited an immature neutrophil signature and heightened *IL1B*-associated inflammatory programs, supporting the clinical relevance of this inflammatory state. Collectively, our findings suggest that non-cirrhotic HCC progression in MASH is associated with coordinated hyperinflammatory reprogramming.

A highlight of this reprogramming was the shift in macrophage states from *Trem2*^+^ LAMs towards *Trem2*^low^ pro-inflammatory populations. *TREM2*^+^ macrophages are increasingly recognized for their reparative functions in MASH, including clearance of dying, lipid-laden hepatocytes.[12], [13] Their depletion could therefore compromise tissue repair and permit inflammatory signals to persist. Consistent with this possibility, *Trem2* has been shown to suppress tumorigenesis in chemical models of HCC,^41^ while *Trem2* deficiency enhances macrophage *Il1b* expression and inflammatory signaling in pancreatic cancer.^42^ Our trajectory analyses further suggest that *Trem2*^+^ LAMs may transition towards a stress-responsive, IL-1β-expressing macrophage state within tumors. Together, these observations raise the possibility that disruption of TREM2-associated reparative macrophage programs contributes to escalating myeloid inflammation during MASH-to-HCC progression.

Myeloid-derived IL-1β may provide an organizing signal connecting this macrophage reprogramming to the broader cellular changes observed in HCC progressors. IL-1β cooperates with IL-6 and IL-23 to promote Th17 differentiation,^40^ and with G-CSF, IL-6 and IL-17A to drive emergency granulopoiesis and mobilization of immature neutrophils.^11^ It also potently activates endothelial cells and promotes leukocyte recruitment.^43^ Accordingly, the increased Th17/Treg balance, accumulation of immature neutrophils and endothelial activation observed in adjacent non-tumor liver could represent interconnected manifestations of an IL-1β-associated inflammatory circuit rather than independent phenomena.

IL-17A itself can promote HCC development in experimental MASH,^44^ while IL-1β and IL-6/STAT3 signaling have established pro-tumorigenic functions,[45], [46] providing potential mechanisms through which sustained inflammation could establish a tumor-permissive environment.

Endothelial cells may be particularly important amplifiers of this circuit. MASH was associated with loss of canonical LSEC states and acquisition of lipid metabolic and extracellular matrix remodeling programs, followed by inflammatory endothelial activation in HCC progressors. CellChat predicted extensive communication between ECs and neutrophils, dendritic cells and T cells, with myeloid-derived IL-1β representing a major input to multiple endothelial populations. Activated ECs could therefore translate myeloid inflammatory signals into enhanced leukocyte recruitment and retention, potentially establishing a feed-forward circuit that sustains inflammation within the cancer-primed liver. The emergence of oncofetal EC states within tumors further indicates progressive endothelial reprogramming as MASH transitions to established HCC.

Our lymphocyte analyses provide additional evidence of chronic immune dysregulation. Alongside Th17 skewing, we identified dysfunctional, anergic-like T-cell and atypical B-cell states resembling populations described during chronic infection and autoimmunity.^32^ Previous studies have implicated autoreactive T cells in MASH-associated hepatocarcinogenesis,[3], [7] suggesting that persistent antigenic stimulation may contribute to this immune landscape. Whether the dysfunctional lymphocyte populations identified here actively promote HCC progression or instead reflect chronic inflammatory exposure remains unclear.

These findings lead us to propose a model in which loss or reprogramming of reparative Trem2^+^ macrophages permits increased myeloid IL-1β signaling, which in turn reinforces endothelial activation, emergency granulopoiesis and Th17 inflammation. These interacting processes could establish a self-sustaining hyperinflammatory field permissive for malignant transformation. Importantly, however, the cross-sectional design of our study cannot establish the temporal sequence or causality of these events, and the inflammatory state could partly arise as a consequence of established tumorigenesis. Prospective studies tracking these cellular states before tumor development, together with targeted perturbation of TREM2 and IL-1β signaling, will be required to test this model.

The hyperinflammatory and autoimmune-like features of HCC-progressed MASH raise the possibility that immunosuppressive therapies could prevent HCC development. However, glucocorticoids induce hyperglycemia, hyperinsulinemia and insulin resistance, potentially worsening metabolic dysfunction,^47^ while IL-1β blockade failed to ameliorate disease in a preclinical MASH model.^48^ Similarly, CCR2/CCR5 inhibition targeting inflammatory monocyte/macrophage recruitment failed in phase III MASH trials.^49^ These outcomes highlight the complexity and redundancy of inflammatory networks in MASH and caution against targeting individual inflammatory pathways in isolation.

## Abbreviations

ABC, age-associated B cell; Adj.NT, adjacent non-tumor; AFP, alpha-fetoprotein; ALT, alanine transaminase; Bgc, germinal center B cell; CTL, cytotoxic T lymphocyte; DC3, dendritic cell 3; DEG, differentially expressed gene; EC, endothelial cell; FFPE, formalin-fixed paraffin-embedded; GAN, Gubra-Amylin-NASH; HB, HCC-bearing; HCA, hepatocellular adenoma; HCCA, hepatocholangiocellular adenoma; HCC, hepatocellular carcinoma; ILC1, innate lymphoid cell type 1; KC, Kupffer cell; LAM, lipid-associated macrophage; LFD, low-fat diet; LR, ligand-receptor; LSEC, liver sinusoidal endothelial cell; MASH, metabolic dysfunction-associated steatohepatitis; MASLD, metabolic dysfunction-associated steatotic liver disease; MHC, major histocompatibility complex; NTB, non-tumor-bearing; TBA, total bile acids; TCR, T-cell receptor; Th17, T helper 17; Treg, T regulatory cell; UMAP, uniform manifold approximation and projection.

## Authors’ contributions

Shawn Lu Wen Tan: Conceptualization, Data Curation, Formal analysis, Investigation, Methodology, Visualization, Writing – original draft, Writing – review & editing. Erez Israeli: Formal analysis, Writing – review & editing. Jillian Wee: Investigation. Hui Min Tan: Investigation. Ravisankar Rajarethinam: Formal analysis. Immanuel Kwok: Methodology, Writing – review & editing. Shu Zhen Chong: Methodology, Writing – review & editing. Weiping Han: Supervision, Funding acquisition, Conceptualization, Writing – review & editing.

## Data availability

All bulk RNA-sequencing and single-cell transcriptomic data have been deposited in the GEO Database under accession numbers GSE308029 and GSE308046, respectively. Additional data supporting the findings of this study are available from the corresponding author upon reasonable request.

## Declaration of generative AI and AI-assisted technologies in the writing process

During the preparation of this work the authors used ChatGPT (OpenAI) in order to improve language and readability. After using this tool/service, the authors reviewed and edited the content as needed and take full responsibility for the content of the manuscript.

## Financial support

This work was funded by Competitive Research Program grant (NRF-CRP23-2019-0004) from the 10.13039/501100001381National Research Foundation, Singapore.

## Conflict of interest

The authors declare that they have no conflict of interest.

Please refer to the accompanying ICMJE disclosure forms for further details.
