# Supplementary material for: Hyperinflammatory reprogramming marks the transition from MASH to HCC
Source: JHEP Rep. 2026 Oct 7;8(12):102055. doi: 10.1016/j.jhepr.2026.102055 (PMC13756514; doi:10.1016/j.jhepr.2026.102055)
Supplement: Supplementary file 1 [file mmc1.pdf]

Hyperinflammatory reprogramming marks the transition from MASH to HCC

Shawn Lu Wen Tan, Erez Israeli, Jillian Wee, Hui Min Tan, Ravisankar Rajarethinam, Immanuel Kwok, Chong Shu Zhen, Weiping Han

Table of contents

Supplementary methods……………………………………………………………….………..2
Fig. S1………………………………………………………………………………...….....9
Fig. S2……………………………………………………………………………………11
Fig. S3……………………………………………………………………………………12
Fig. S4……………………………………………………………………………………14
Fig. S5……………………………………………………………………………………16
Fig. S6……………………………………………………………………………………18
Fig. S7……………………………………………………………………………………19
Fig. S8……………………………………………………………………………………20
Fig. S9……………………………………………………………………………………21
Table S1………………………………………………………………………………....22
Table S2………………………………………………………………………………....25
Table S3………………………………………………………………………………....30
Table S4………………………………………………………………………………....31
Table S5………………………………………………………………………………....34
Table S6………………………………………………………………………………....35
Table S7………………………………………………………………………………....37
Table S8………………………………………………………………………………....49
Table S9………………………………………………………………………………....53
Supplementary references………………………………………………….……….....55

Supplementary Methods

Serum measurements of ALT activity, TBA and AFP

Serum ALT activity, TBA and AFP were measured using the ALT Activity Assay Kit (Abcam, ab105134; 2 µL serum in duplicate), TBA Kit (Cell Biolabs, STA-631; 10 µL) and Mouse AFP ELISA Kit (R&D Systems, MAFP00; 2.5 µL), respectively, according to the manufacturers' instructions.

Immunohistochemistry of liver sections

FFPE liver sections (5 µm) were stained with H&E or Fast Green/Sirius Red (0.1% Direct Red 80, 0.1% Fast Green in 1.3% picric acid; Sigma-Aldrich). For Ki67 immunohistochemistry, sections were deparaffinized, rehydrated and subjected to antigen retrieval in citrate buffer (pH 6.0) at 90 °C for 20 min. Following peroxidase blocking (Bloxall, Vector Laboratories, SP-6000) and blocking with 10% goat serum, sections were incubated with anti-Ki67 (Abcam, ab15580; 1:1000) overnight at 4 °C, followed by biotinylated goat secondary antibody (1:200) for 30 min. Signal was developed using VECTASTAIN Elite ABC HRP and ImmPACT DAB substrate (Vector Laboratories, SK-4105). Sections were counterstained with hematoxylin, dehydrated and mounted in Permount (Fisher Scientific, SP15-500).

Preparation for bulk RNA-sequencing

RNAlater-preserved mouse liver (10–20 mg) was homogenized in RLT buffer containing β-mercaptoethanol using a TissueLyser II (20 Hz, 2 × 2 min). RNA was extracted using the RNeasy Plus Kit (Qiagen) according to the manufacturer's instructions, except that 50% instead of 70% ethanol was used for RNA precipitation.

RNA quantity and quality were assessed using a NanoDrop (Thermo Fisher Scientific) and Agilent 2100/2200 Bioanalyzer (Agilent Technologies). One microgram of total RNA was used for library preparation.

Library preparation for bulk RNA-sequencing

Sequencing libraries were prepared according to the manufacturer's protocol. Poly(A) mRNA was isolated using the Poly(A) mRNA Magnetic Isolation Module. RNA was fragmented and primed, followed by first- and second-strand cDNA synthesis using ProtoScript II Reverse Transcriptase and Second Strand Synthesis Enzyme Mix, respectively. Purified double-stranded cDNA was end-repaired, dA-tailed, adaptor-ligated and size-selected to recover ~400-bp fragments (~300-bp inserts). Libraries were PCR-amplified with indexed P5/P7 primers, bead-purified, validated using a Qsep100 (Bioptic), and quantified using a Qubit 3.0 Fluorometer (Invitrogen). Indexed libraries were multiplexed and sequenced on an Illumina NovaSeq using 2 × 150-bp paired-end reads.

Pre-processing of bulk RNA-sequencing

Fastq files were trimmed (15 bp) and checked for quality control metrics using Fastp [1] and aligned to mm10 mouse reference genome using STAR (2.7.11a) and further filtered to remove non-primary alignments, reads that fail platform quality checks, optical duplicates, or supplementary alignments using Samtools (1.6). Raw counts and normalized counts were extracted using HTSeq (2.0.3) and Cufflinks (2.2.1). Batch correction was performed using the *ComBat-seq* function from the sva R package (3.44.0). ComBat-seq-corrected counts were used for differential expression analyses

using DESeq2. Gene set enrichment analyses were performed using gprofiler2 R package.

Sample preparation for scRNA-sequencing

Mouse liver tissues were stored overnight in MACS Tissue Storage Solution (Miltenyi Biotec, 130-100-008), with tumor-bearing livers separated into tumor and adjacent non-tumor (Adj.NT) regions. Up to 1 g of tissue was minced and dissociated using the Tumor Dissociation Kit and gentleMACS Octo Dissociator (Miltenyi Biotec, 130-096-730; 37C_m_TDK_2 program) according to the manufacturer’s instructions. Dissociated cells were filtered through a 70-μm strainer, subjected to red blood cell lysis (Miltenyi Biotec, 130-094-183), and washed with cold PBS before downstream processing.

Pre-processing of scRNA-sequencing data, quality control and clustering

Raw reads were processed using Cell Ranger v7.1.0 (*cellranger multi*) for hashtag-based sample deconvolution, alignment to the mm10 reference genome, and generation of gene expression and Feature Barcode matrices. Data were analyzed using Seurat v5.0.0. Cells were excluded if mitochondrial read percentage deviated by >2.5 median absolute deviations (MADs), or total counts or detected genes by >3 MADs from the sample median. Cells with abnormally high counts relative to detected genes were additionally excluded using a log-log linear regression model (intercept offset, 0.16). Data were normalized and scaled using *SCTransform* and integrated using *IntegrateData*. Clustering, UMAP visualization and differential expression analysis (*FindAllMarkers*, Wilcoxon test) were performed using Seurat. Clusters were

annotated based on marker gene expression. Major cell types were re-clustered and iteratively refined to remove suspected doublets and annotate subclusters.

Calculation of enrichment scores between sample groups for subclusters of the single-cell RNA-sequencing dataset.

To calculate enrichment of cell clusters across sample groups, we derived expected cell numbers for each cluster in each sample group using the Chi-square formula. Enrichment scores were quantified by dividing the observed number of cells by the expected number of cells for each cluster in each sample group.

Diffusion mapping and trajectory analyses

Diffusion maps were plotted using the destiny (3.23) R package [2]. The *Velocyto* python implementation [3] was used to generate loom files from BAM files of previous CellRanger output. Expression count matrices, gene names, PCA and UMAP embeddings were extracted from seurat objects and used to construct an anndata object using the anndata python package [4]. Utilising anndata objects, scVelo [5] and Scanpy [6] python packages were used to pre-process and compute RNA velocities using the dynamical model in *scVelo*. CellRank2 [7] was used to integrate RNA velocity kernels with gene similarity kernels in a 4:1 ratio to infer cellular trajectories. Initial and terminal macrostates were predicted by CellRank2 using the Generalized Perron Cluster Cluster Analysis (GPCCA) algorithm. Cellular differentiation scores were estimated using the CytoTRACE R package (0.3.3) based on the observation that the number of expressed genes decreases during differentiation [8].

Single-cell gene set enrichment

The *AddModuleScore* function in Seurat (5.0.0) was used to quantify enrichment scores for selected gene sets in single-cells using default parameters. The function calculates the average expression level of each gene set on the single-cell level and subtracts the aggregated expression of a control gene set randomly selected from each bin of genes binned based on averaged expression. Z-scores were calculated based on the enrichment scores and the average Z-score within each cluster/group was plotted in heatmaps using ComplexHeatmap R package. Hallmark, KEGG, Reactome and Gene Ontology Gene sets were derived from MSigDB database.

Curation of immature neutrophil signature and single sample gene set enrichment of human dataset.

Positive DEGs of subcluster N3 versus all other subclusters except for N6 subcluster (also an immature neutrophil subcluster) were extracted using *FindMarkers* function utilizing the Wilcoxon statistical test. A stringent filtering criteria of $\log_2$ fold change > 7 and adjusted *p*-value < 1e-20 was applied which resulted in eight top genes remaining (*Camp, Ngp, Cd177, Gpr27, Itgb2l, Ltf, F730016J06Rik, 4930438A08Rik*). Human orthologs for these genes are derived using the babelgene (22.9) R package, for which 5 human orthologs were available (*CAMP, CD177, GPR27, ITGB2, LTF*). These five genes constituted the human immature neutrophil signature.

G-CSFcx mouse experiment, immune cell isolation and flow cytometry

For each dose, 1.5 µg G-CSF (Neupogen, Amgen) was incubated with 7.5 µg anti-G-CSF monoclonal antibody (Southern Biotech, 10128-01) at 37 °C for 20 min to form G-CSF/anti-G-CSF complexes (G-CSFcx), then diluted to 150 µL with sterile saline. Starting at 48 weeks, GAN- and LFD-fed mice received six retro-orbital doses of G-

CSFcx or saline at 2-week intervals over 12 weeks. At sacrifice, 10 µL tail blood was mixed with 10 µL 0.5 M EDTA. Following red blood cell lysis, cells were stained with primary antibodies (1:1000 each) for 20 min at 4 °C, washed, and resuspended in PBS containing 1 µM DAPI and CountBright beads (1:100; Thermo Fisher Scientific, C36950) for flow cytometry. Livers were separated into tumor and adjacent non-tumor regions where applicable. Up to 1 g of tissue was minced and digested for 30 min at 37 °C in RPMI containing DNase I (0.25 mg/mL; Sigma-Aldrich, 10104159001), collagenase IV (385 U/mL; Sigma-Aldrich, C51380) and 10% FBS, using 2 mL digestion buffer per gram of tissue. Samples were processed on a gentleMACS Octo Dissociator using the m_impTumour_03 and m_impTumour01 programs twice each, filtered through 70-µm MACS SmartStrainers (Miltenyi Biotec, 130-098-462), and enriched by 33% Percoll centrifugation (700 × g, 12 min, room temperature). Following red blood cell lysis, cells were stained with anti-Ly6G (BioLegend, 127606), anti-CD11b (BD Biosciences, 563402), anti-Siglec-F (BD Biosciences, 562757) and anti-CD101 (Thermo Fisher Scientific, 12-1011-82) at 1:200 for 20 min at 4°C. Cells were washed and resuspended in PBS containing 3 µM DAPI and CountBright beads (1:10) before analysis on a NovoCyte Penteon flow cytometer (Agilent Technologies). Data were analyzed using FlowJo v10.9.0.

Generation and cell culture of RAW264.7 Trem2 KO using CRISPR/Cas9.

Single-guide RNAs (sgRNAs) targeting murine *Trem2* were cloned into PX458 plasmid (Addgene, 48138) and transfected into RAW264.7 macrophages using HiPerFect Transfection Reagent (Qiagen, 301704). Single GFP-positive cells were sorted into individual wells of a 96-well plate. Clones were picked and screened for successful Trem2 KO using anti-Trem2 antibody (Cell Signaling, 61788) via western blot.

Successful Trem2 KO clones were verified using Sanger sequencing. RAW264.7 macrophages were cultured in DMEM containing 10% dialyzed fetal bovine serum (ThermoFisher, 26400044) and 1% penicillin/streptomycin in humidified 37°C incubators with 5% CO2.

Immunofluorescence of RAW264.7 cells

RAW264.7 cells were seeded on glass coverslips and fixed the next day with 4% paraformaldehyde for 10 min at room temperature. Following PBS washes, cells were permeabilized with 1X PBS/0.1% Triton-X for 10 min and blocked with 2% bovine serum albumin for 30 min. Cells were incubated with primary antibodies (anti-Trem2, Abcam, ab305103, 1:250) for 2h at room temperature. Cells were washed 3x with 1X PBS. Anti-rabbit Alexa Fluor 647 antibodies were applied for 30 min at room temperature. Cells were washed 3x with 1X PBS, treated with 1 µg/ml DAPI for 5 min and mounted on glass slides using FluorSave Reagent (Sigma Aldrich, 345789). Images were acquired using a Nikon Ni-E Upright microscope and analyzed using ImageJ.

Figure S1, related to Figure 1

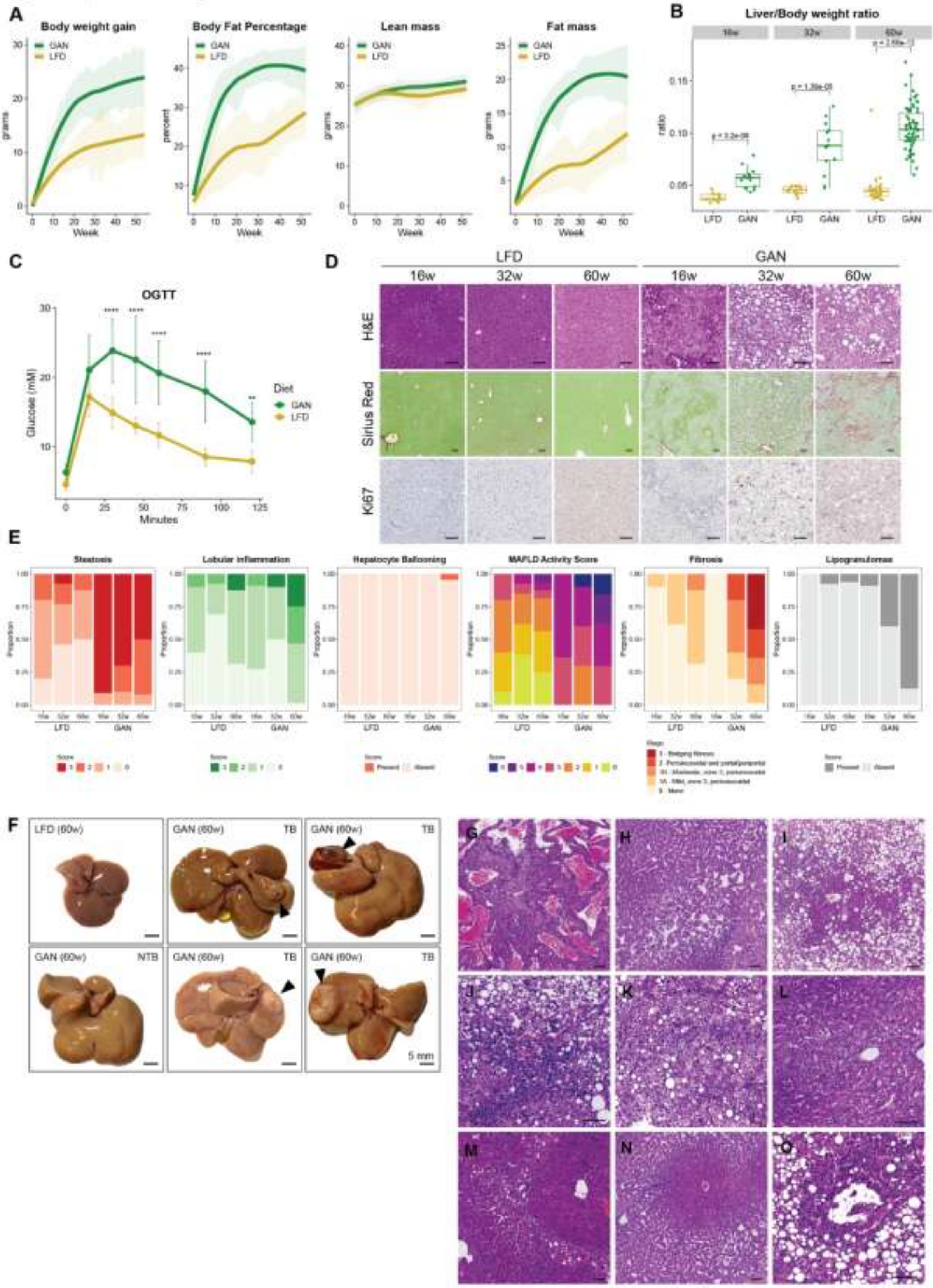

Figure S1, related to Figure 1

A) Longitudinal measurements of body weight and composition parameters of mice challenged with LFD or GAN diets across the study duration. Darker solid lines indicate the smoothed mean values, lighter shaded areas indicate the standard deviation.
B) Liver-to-body weight ratios of mice at the various timepoints. WIlcoxon rank-sum test.
C) Oral glucose intolerance test of LFD (n=5) and GAN (n=5) mice following 16 weeks of diet exposure. Mean ± SD shown, two-tailed Student's t-test.
D) Representative images of liver sections stained with hematoxylin and eosin, Sirius red, and Ki67. Scale bars represent 50 μm.
E) Histopathology scoring of liver sections across the entire cohort. See Methods for details on scoring criteria.
F) Representative pictures of whole livers. TB – tumor-bearing; NTB – non-tumor-bearing. Black arrows indicate where tumors are observed.
G) H&E image of a HCC with trabecular pattern featuring blood lakes.
H) H&E image of a HCC with trabecular pattern.
I) H&E image of a HCC with trabecular pattern with macrovesicular steatosis.
J) H&E image of a steatohepatitis HCC with macrovesicular steatosis and immune infiltration.
K) H&E image of a HCC with clear-cell pattern.
L) H&E image of a HCC with clear-cell pattern.
M) H&E image of a HCC with mixed trabecular and solid patterns.
N) H&E image of a HCC with mixed trabecular and solid patterns.
O) H&E image of vascular invasion of cancer cells into a neighbouring vessel. Scale bars represent 50 μm.

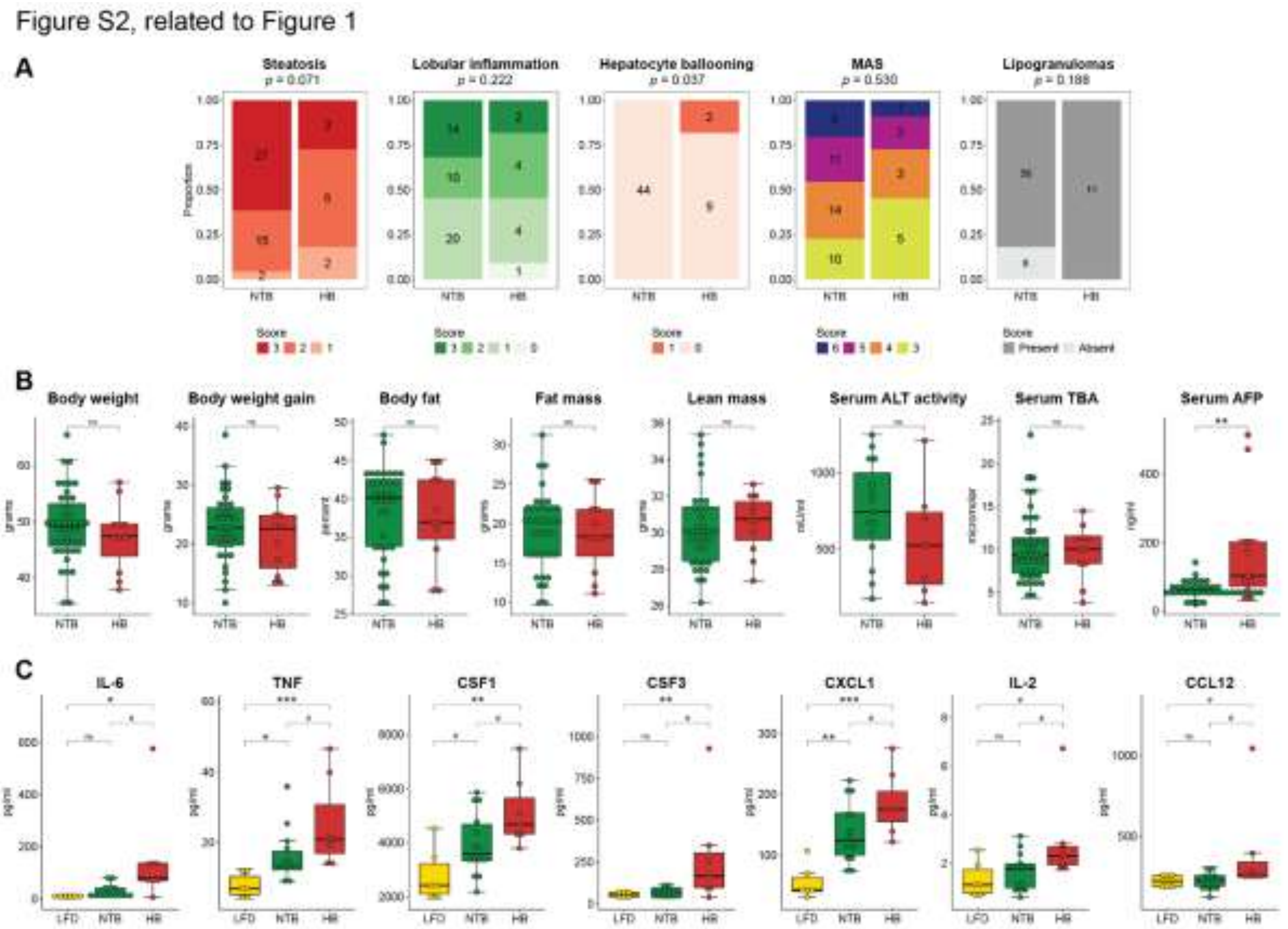

Figure S2, related to Figure 1

A) Pathology evaluation scores of NTB and HB 60-week GAN liver sections for steatosis, lobular inflammation, hepatocyte ballooning, MAFLD activity score (MAS) and presence of lipogranulomas. Fisher’s exact test.

B) Body weight, body composition parameters and abundance of serum markers in NTB and HB 60-week GAN mice. WIlcoxon rank-sum test.

C) Absolute abundance of significantly different cytokines highlighted in red in Figure 1L. Post-hoc Dunn’s test following Kruskal-Wallis test, Benjamini-Hochberg adjusted *p*-values. *p* >0.1, ns; *p* <0.1, #; *p* <0.05, *; *p* <0.01, **; *p* <0.001, ***.

Figure S3, related to Figure 2

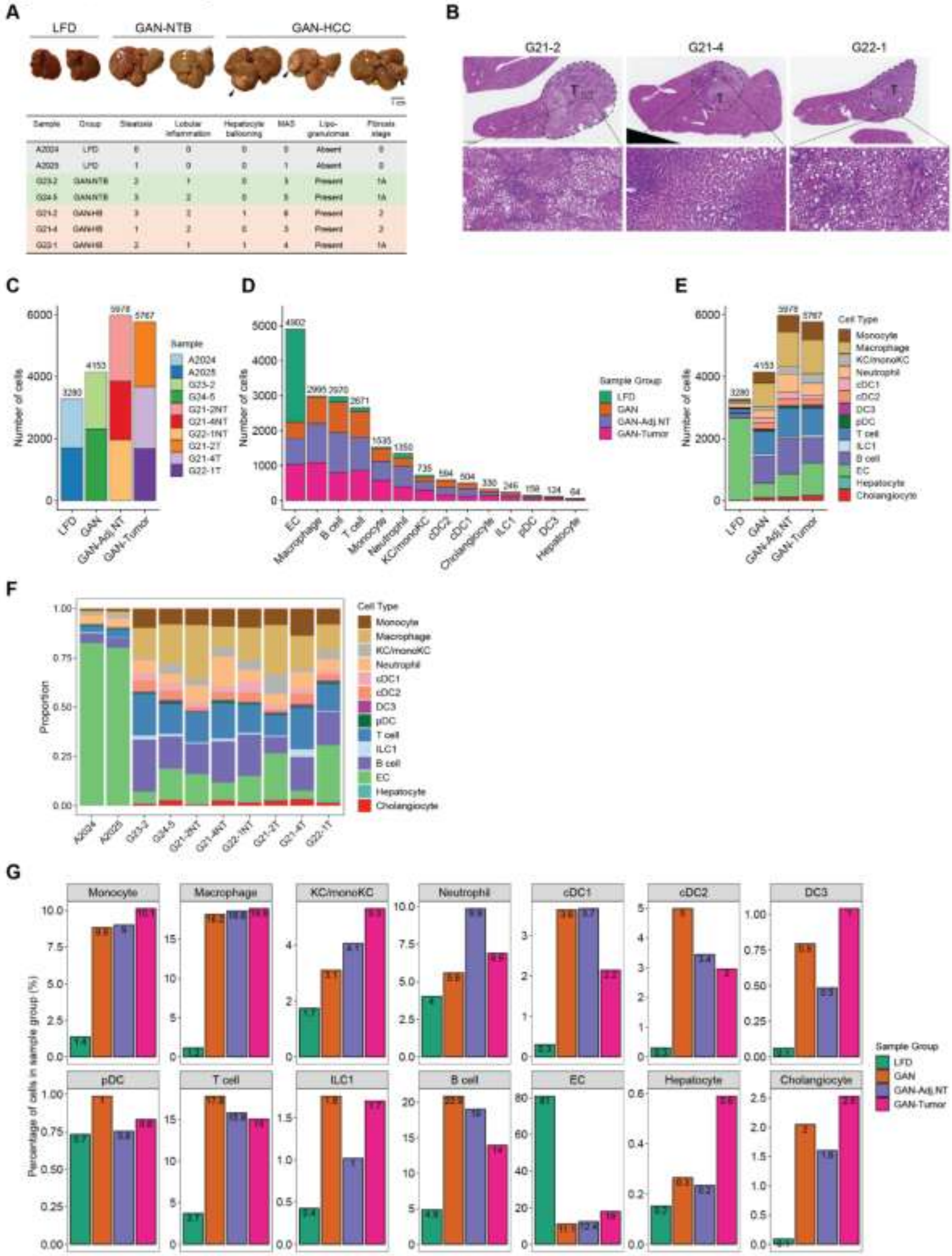

Figure S3, related to Figure 2.

A) Images of livers used for single-cell transcriptomics. Bottom table shows histopathology scores of liver sections taken from each liver.
B) H&E images of GAN-HCC livers used for single-cell RNA-sequencing.
C) Number of cells in each sample group contributed by individual samples.
D) Number of cells in each cell type contributed by each sample group.
E) Number of cells in each sample group contributed by each cell type.
F) Proportion of cell types in each sample.
G) Percentage of each cell type in each sample group.

Figure S4, related to Figure 3

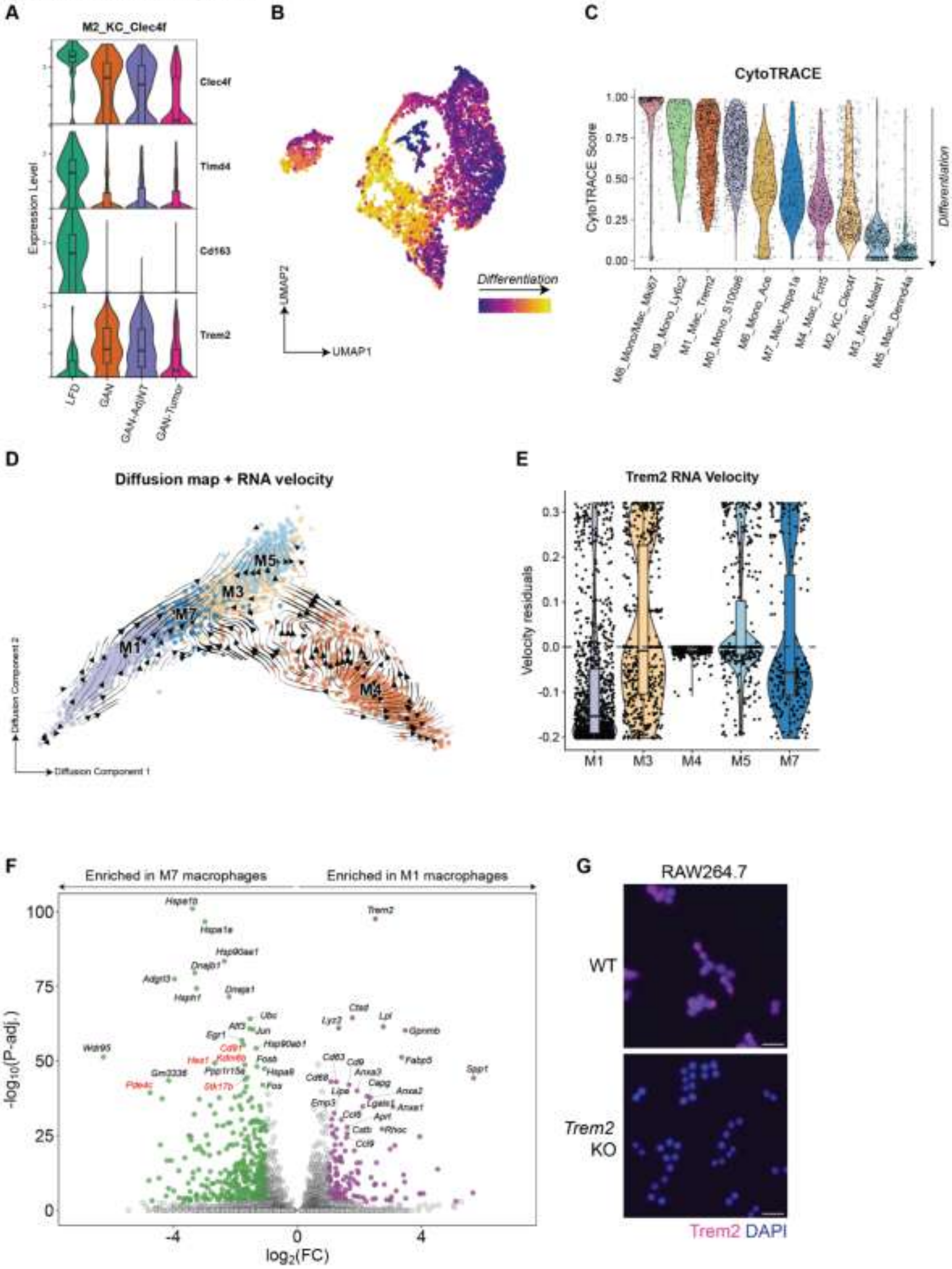

Figure S4, related to Figure 3.

A) Expression of genes in M2_KC_Clec4f cells across sample groups.
B) UMAP showing CytoTRACE scores of monocytes and macrophages to predict degree of differentiation.
C) CytoTRACE scores across monocyte and macrophage subclusters.
D) Diffusion map and RNA velocity vectors of macrophage subclusters.
E) RNA velocity residuals of Trem2 in macrophage subclusters.
F) Differential expression analysis of M1 and M7 macrophages.
G) Immunofluorescence staining of RAW264.7 macrophages for Trem2 (magenta) and DAPI (dark blue). Scale bars represent 20 μm. WT – wild type, KO – knockout.

Figure S5, related to Figure 4

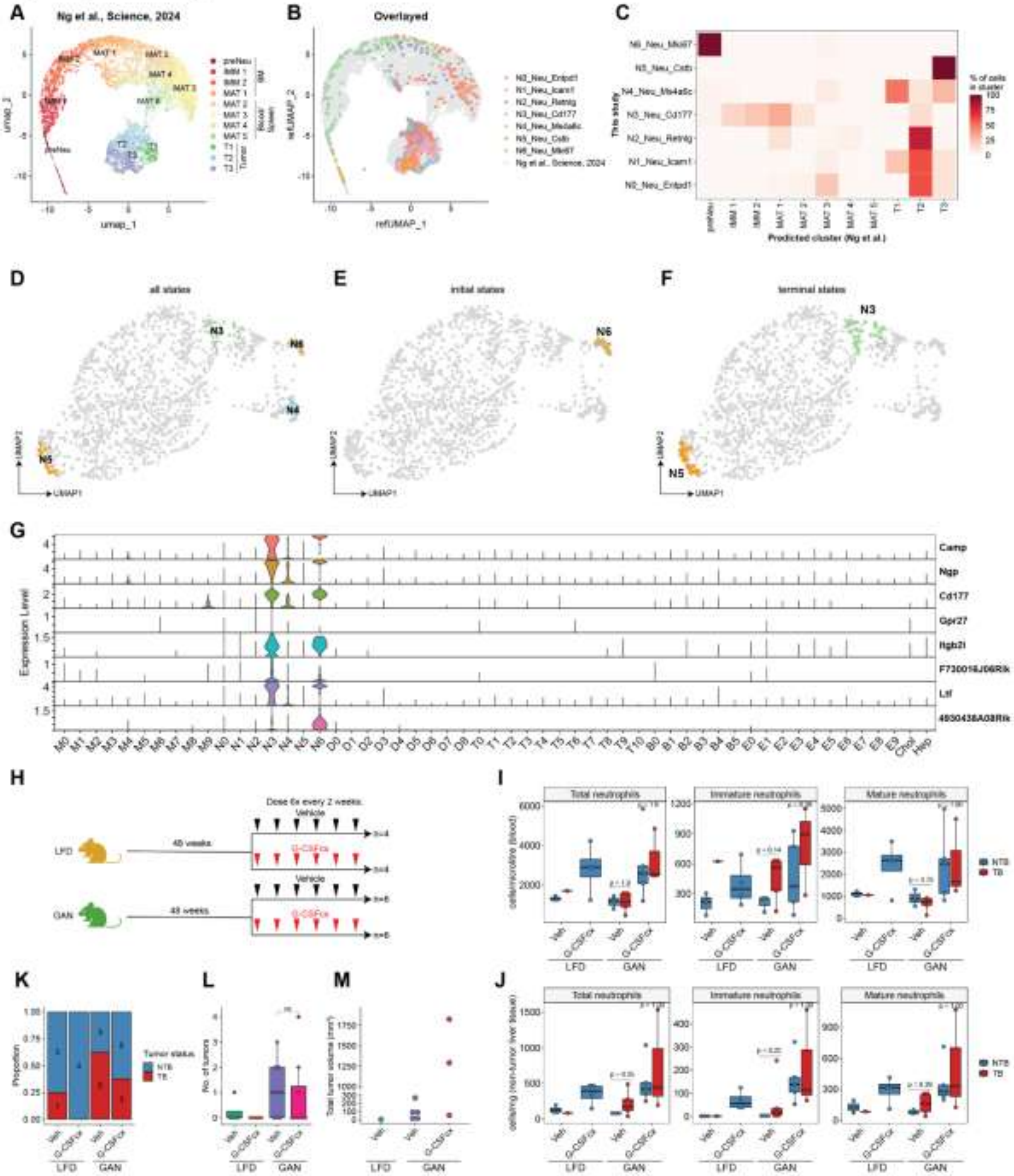

Figure S5, related to Figure 4.

A) UMAP of neutrophils from[9] to be used as reference for S5B.

B) Overlaying of neutrophils from this study onto the reference UMAP of S5A.

C) Percentage of each neutrophil subcluster in this study predicted to overlap with respective clusters from [9].

D) Prediction of macrostates by CellRank2.

E) Prediction of initial macrostates by CellRank2.

F) Prediction of terminal macrostates by CellRank2.

G) Expression of the top eight genes enriched in N3 and N6 compared with all other subclusters in the dataset.

H) Experimental set-up for G-CSFcx *in vivo* experiment.

I) Absolute number of neutrophils ($Ly6G^+Cd11b^+$), immature neutrophils ($Cd101^-Ly6G^+Cd11b^+$) and mature neutrophils ($Cd101^+Ly6G^+Cd11b^+$) in peripheral blood as measured by flow cytometry. WIlcoxon rank-sum test.

J) Absolute number of neutrophils ($Ly6G^+Cd11b^+$), immature neutrophils ($Cd101^-Ly6G^+Cd11b^+$) and mature neutrophils ($Cd101^+Ly6G^+Cd11b^+$) in liver tissues as measured by flow cytometry. WIlcoxon rank-sum test.

K) Proportion of non-tumor-bearing (NTB) and tumor-bearing (TB) mice in the G-CSFcx experiment.

L) Number of tumors (> 2 mm^3) in each liver in the G-CSFcx experiment. WIlcoxon rank-sum test.

M) Total tumor volume per liver in the G-CSFcx experiment.

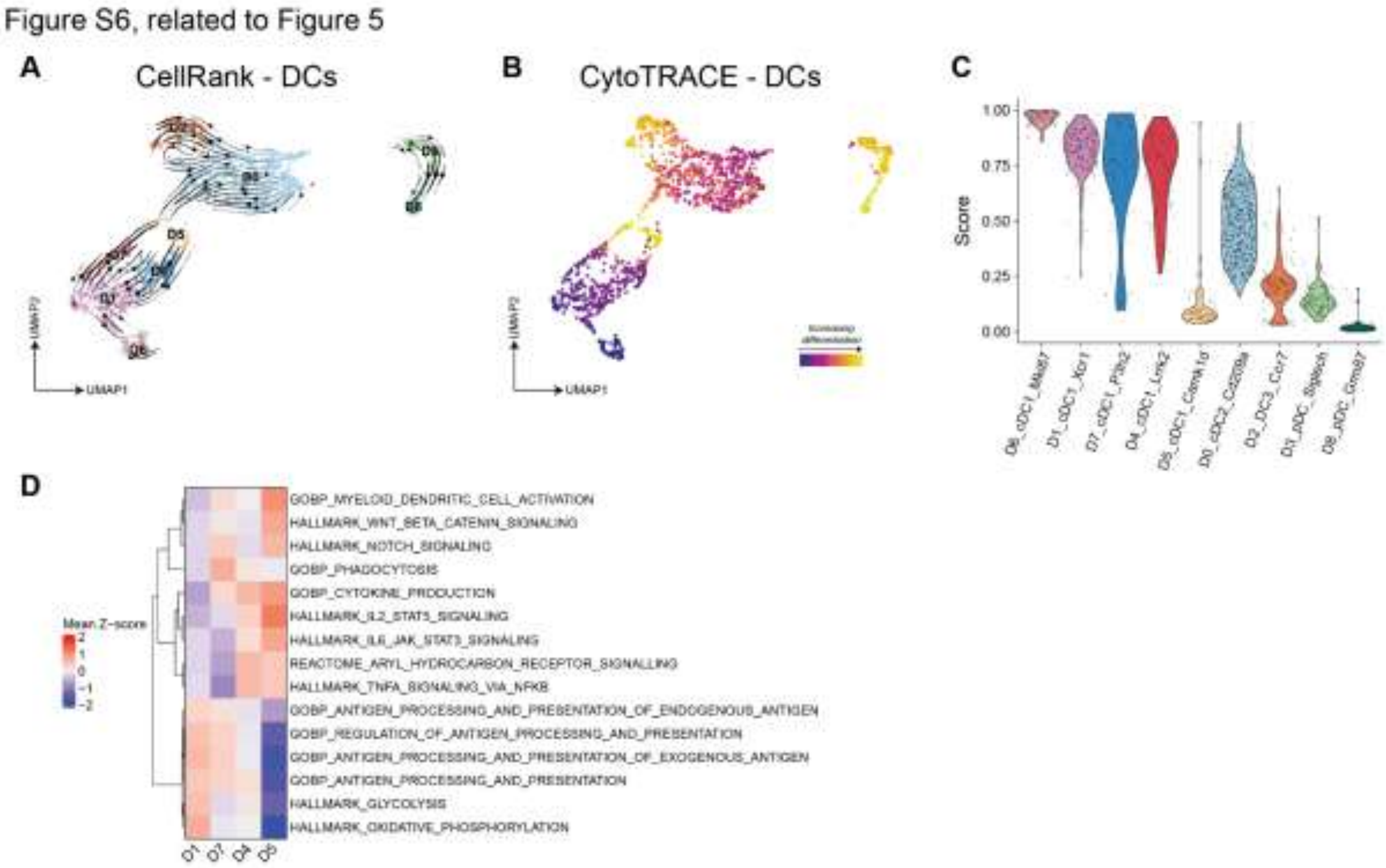

Figure S6, related to Figure 5

A) Trajectory analysis of dendritic cells by CellRank2 based on RNA velocity and gene similarity.
B) UMAP showing CytoTRACE scores of dendritic cells.
C) CytoTRACE scores of dendritic cells across subclusters.
D) Single-cell gene set enrichment Z-scores of gene sets across cDC1 clusters.

Figure S7, related to Figure 6

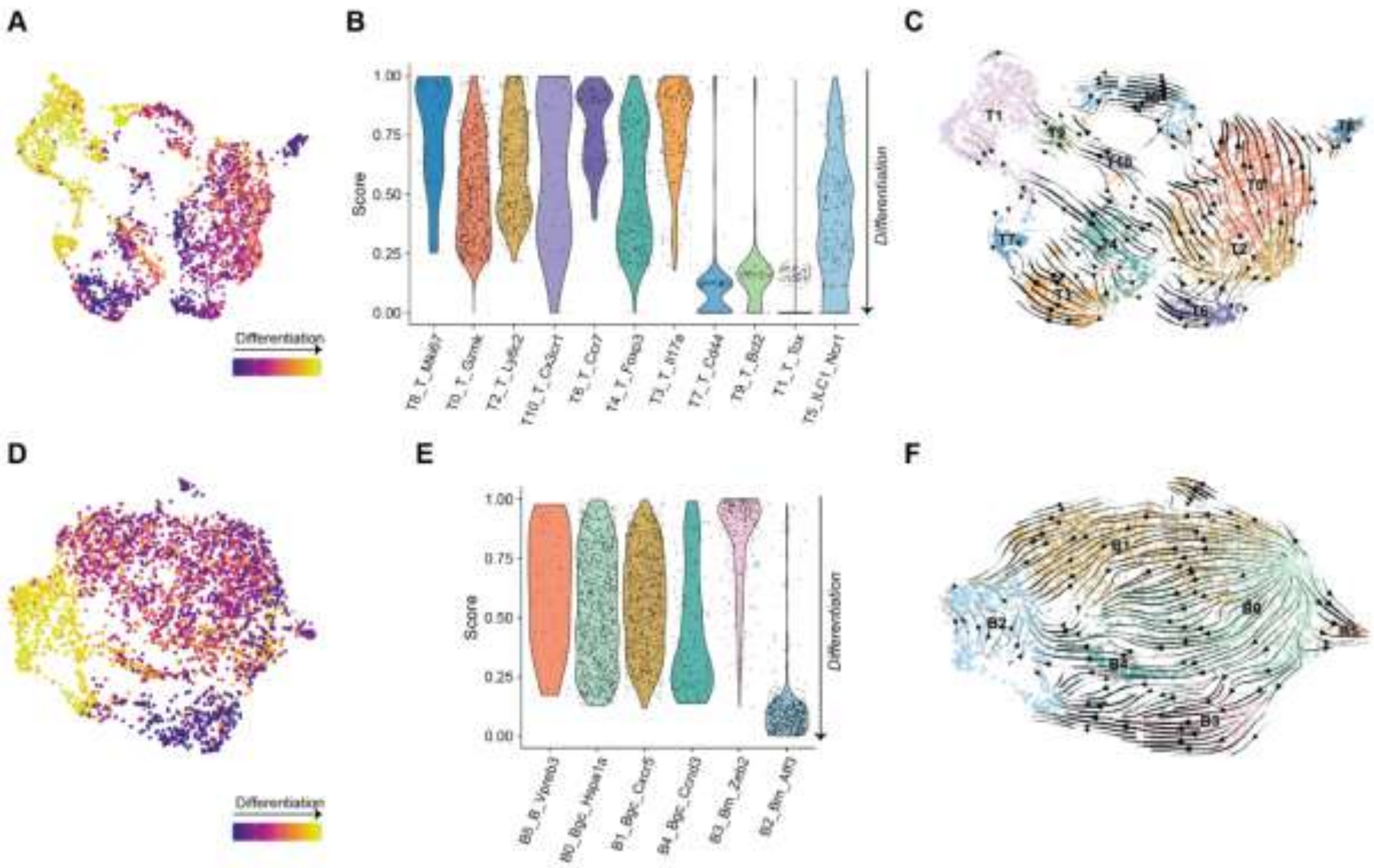

Figure S7, related to Figure 6.

A) UMAP showing CytoTRACE scores of T cells and ILC1.

B) CytoTRACE scores of T cells and ILC1 across subclusters.

C) Trajectory analysis of T cells and ILC1 by CellRank2 based on RNA velocity and gene similarity.

D) UMAP showing CytoTRACE scores of B cells.

E) CytoTRACE scores of B cells across subclusters.

F) Trajectory analysis of B cells by CellRank2 based on RNA velocity and gene similarity.

Figure S8, related to Figure 7

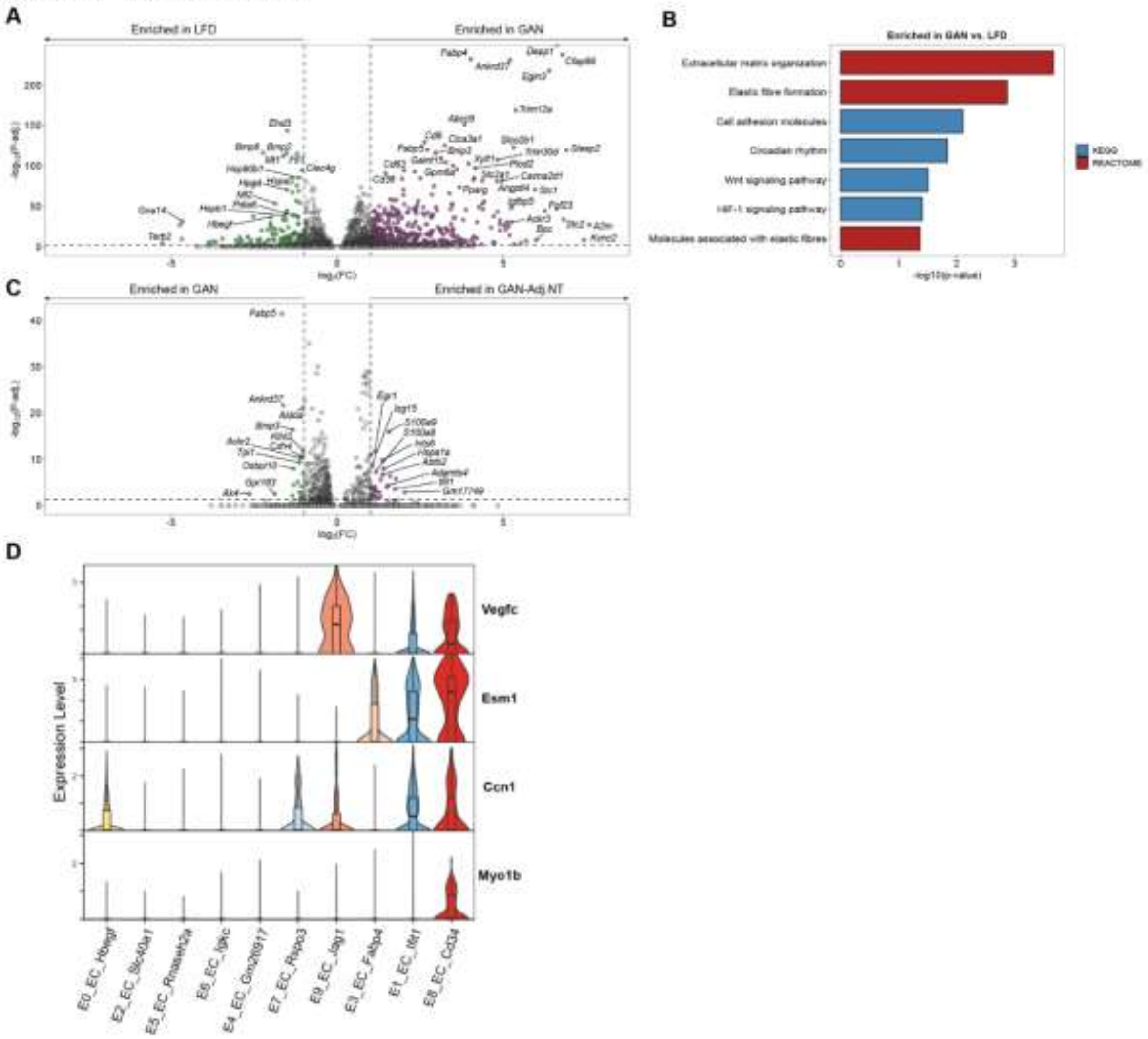

Figure S8, related to Figure 7.

A) Volcano plot showing differential gene expression between GAN and LFD ECs.

B) KEGG and Reactome pathway enrichment analysis of genes upregulated in GAN versus LFD ECs. P-values calculated using the g:SCS algorithm of gProfiler2.

C) Volcano plot showing differential gene expression between GAN-Adj.NT vs GAN ECs.

D) Expression of pro-angiogenic factors across EC subclusters.

Figure S9, related to Figure 8

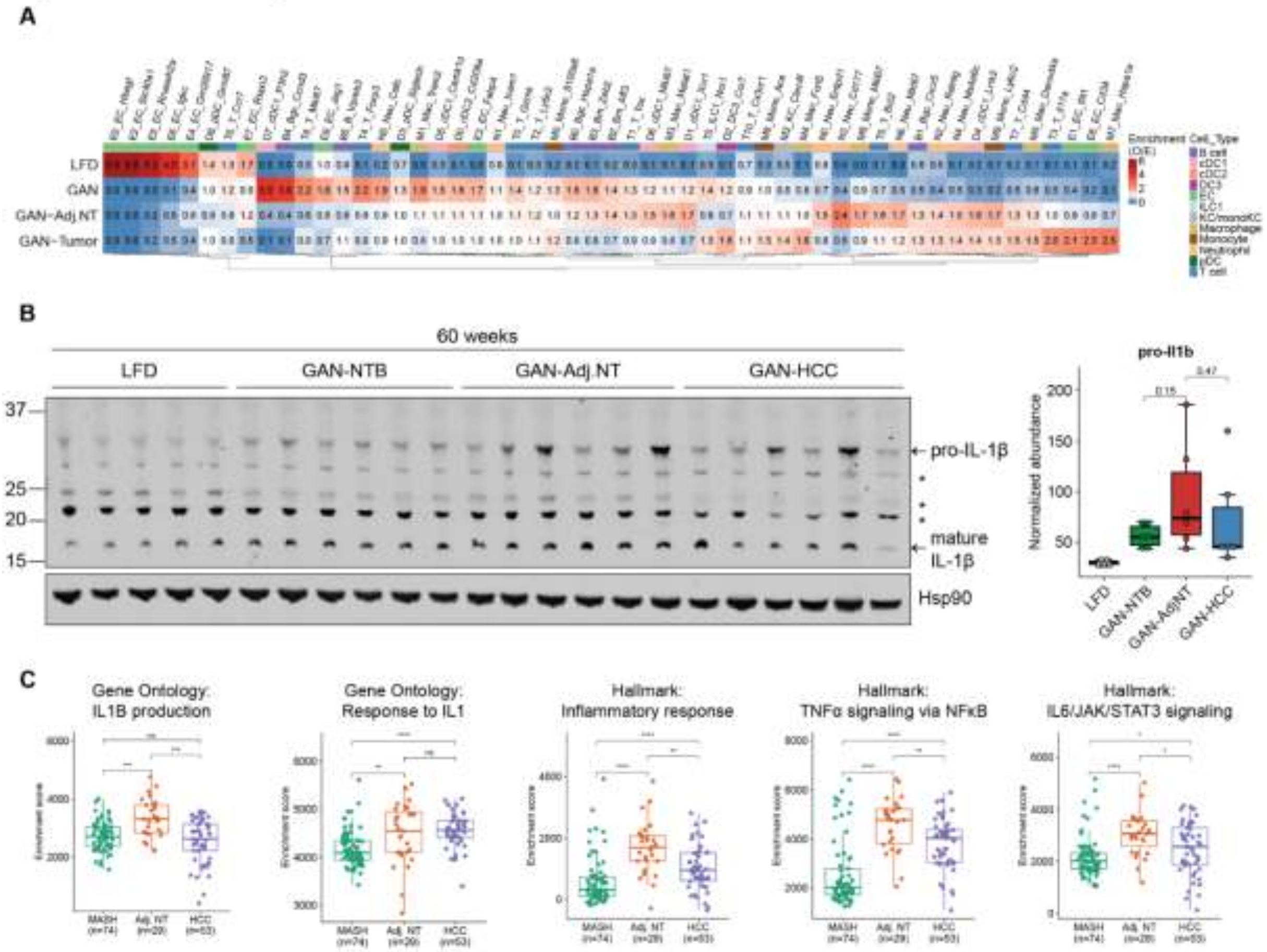

Figure S9, related to Figure 8

A) Hierarchical clustering of enrichment scores for all 53 subclusters across sample groups.

B) Western blot of IL-1β in liver tissues from mice fed the LFD or GAN diets for 60 weeks. Boxplot on the right shows the densitometric measurements of pro-IL1b normalised to Hsp90. WIlcoxon rank-sum test.

C) ssGSEA enrichment scores for the indicated gene signatures in liver tissues from a human cohort of non-cirrhotic MASH and MASH-HCC patients. WIlcoxon rank-sum test.

Supplementary Table 1. Pairwise transcriptomic comparison between HB-Adj.NT and NTB liver tissues. Significant DEGs (adjusted p-value < 0.05) shown.

Gene	baseMean	log2FoldChange	lfcSE	pvalue	padj
Pla2g2d	32.656	4.912	0.993	2.81E-07	4.98E-04
Ccl8	75.854	4.088	0.893	1.26E-06	1.35E-03
Cbr2	9.751	2.799	0.776	4.09E-05	1.45E-02
Ltf	129.477	2.685	0.897	3.04E-07	4.98E-04
Olfm4	6.270	2.660	0.784	8.22E-05	2.00E-02
Cd177	35.935	2.645	0.732	4.01E-05	1.45E-02
Mmp9	114.983	2.623	0.562	4.31E-07	4.98E-04
Alox5	31.890	2.534	0.708	4.23E-05	1.46E-02
Ppbp	111.669	2.523	0.579	1.64E-06	1.52E-03
S100a8	202.046	2.516	0.700	4.07E-05	1.45E-02
Ngp	230.503	2.267	0.941	1.78E-06	1.54E-03
Ccl12	10.320	2.263	0.719	0.00016	3.13E-02
Pglyrp1	29.339	2.135	0.562	1.50E-05	7.40E-03
Tubb1	76.243	2.110	0.623	6.87E-05	1.79E-02
Clec10a	41.364	1.864	0.404	4.02E-07	4.98E-04
Ifitm6	34.359	1.801	0.392	3.98E-07	4.98E-04
Clec4e	44.175	1.792	0.531	6.33E-05	1.72E-02
Trem3	9.437	1.774	0.568	0.00014	2.82E-02
Xlr	31.952	1.764	0.488	2.67E-05	1.19E-02
Ccr3	12.915	1.763	0.570	0.00016	3.13E-02
Lyz1	73.502	1.749	0.499	4.02E-05	1.45E-02
Slc1a3	26.471	1.730	0.422	3.82E-06	2.64E-03
Slc14a1	17.165	1.687	0.405	2.56E-06	2.08E-03
Clec4a1	342.499	1.458	0.299	8.92E-08	2.47E-04
Cd4	78.757	1.402	0.422	6.53E-05	1.74E-02
Fpr1	174.160	1.387	0.304	3.88E-07	4.98E-04
Mcemp1	17.378	1.359	0.457	0.00018	3.39E-02
Clec4n	381.365	1.348	0.283	1.44E-07	3.33E-04
Itgam	446.432	1.346	0.388	3.93E-05	1.45E-02
Pf4	154.457	1.324	0.477	0.00036	4.92E-02
G530011O06Rik	70.967	1.304	0.393	6.23E-05	1.72E-02
Ly6c2	80.186	1.282	0.458	0.00033	4.87E-02
Slc28a2	70.377	1.272	0.299	1.61E-06	1.52E-03
F630028O10Rik	12.342	1.238	0.402	0.00012	2.62E-02
Wfdc17	752.983	1.227	0.307	4.55E-06	3.00E-03
Alox5ap	217.141	1.194	0.314	9.93E-06	5.73E-03
A530064D06Rik	21.969	1.169	0.325	2.07E-05	9.86E-03
Cd300e	93.689	1.100	0.394	0.00030	4.63E-02
Rab37	12.556	1.098	0.362	0.00012	2.48E-02
Il2rg	450.681	1.088	0.341	8.69E-05	2.01E-02
Aoah	267.281	1.083	0.327	5.57E-05	1.64E-02

Ebi3	49.745	1.075	0.314	3.75E-05	1.45E-02
Ltb4r1	20.841	1.050	0.323	6.04E-05	1.72E-02
Pira2	47.565	1.037	0.325	8.23E-05	2.00E-02
Siglece	276.246	1.004	0.268	1.15E-05	6.24E-03
Clec4a2	230.006	0.963	0.304	8.61E-05	2.01E-02
Adgre4	323.501	0.958	0.252	8.15E-06	4.91E-03
Prune2	62.098	0.945	0.347	0.00036	4.92E-02
Pirb	499.728	0.945	0.255	1.17E-05	6.24E-03
1600010M07Rik	11.786	0.944	0.355	0.00033	4.87E-02
Emilin2	221.185	0.919	0.327	0.00027	4.41E-02
Sell	121.831	0.918	0.310	0.00017	3.25E-02
Klra2	180.291	0.915	0.247	1.29E-05	6.59E-03
Cfp	1153.663	0.901	0.234	6.96E-06	4.38E-03
Vsig4	2155.248	0.872	0.316	0.00030	4.64E-02
Clec4a3	371.114	0.860	0.257	4.38E-05	1.48E-02
Ncf1	494.359	0.851	0.248	3.35E-05	1.45E-02
Rac2	675.392	0.842	0.266	7.65E-05	1.93E-02
Csf3r	183.341	0.817	0.294	0.00028	4.51E-02
Cd300lf	73.501	0.803	0.288	0.00026	4.28E-02
P2rx7	301.100	0.795	0.241	4.79E-05	1.58E-02
Cybb	3443.118	0.790	0.250	8.44E-05	2.01E-02
Dock11	183.378	0.788	0.262	0.00014	2.85E-02
Chp2	94.727	0.783	0.193	2.98E-06	2.30E-03
Lrrc25	199.423	0.778	0.236	5.21E-05	1.61E-02
Adgre1	2086.430	0.774	0.235	5.24E-05	1.61E-02
Pilrb2	67.532	0.755	0.246	0.00011	2.33E-02
Fcer1g	750.294	0.749	0.280	0.00035	4.92E-02
Pilrb1	78.800	0.725	0.258	0.00025	4.21E-02
Fgd2	112.764	0.724	0.223	6.19E-05	1.72E-02
Ctsc	4704.155	0.720	0.183	3.28E-06	2.39E-03
Fcgr4	646.624	0.714	0.253	0.00021	3.90E-02
Naaa	242.579	0.697	0.223	9.34E-05	2.09E-02
Prkcb	387.397	0.678	0.246	0.00027	4.41E-02
Hck	454.440	0.668	0.239	0.00024	4.20E-02
Pld4	649.666	0.658	0.233	0.00023	4.03E-02
Nlrp3	72.413	0.652	0.238	0.00028	4.47E-02
P2ry12	169.497	0.628	0.223	0.00022	3.99E-02
P2ry13	247.545	0.612	0.184	4.08E-05	1.45E-02
Fam49b	658.257	0.610	0.192	7.15E-05	1.83E-02
Nfam1	407.598	0.599	0.217	0.00025	4.20E-02
Msr1	1205.189	0.596	0.220	0.00029	4.51E-02
Trpm2	253.465	0.594	0.222	0.00032	4.74E-02
Csf1r	3938.611	0.582	0.209	0.00025	4.20E-02
Gmfg	285.282	0.527	0.205	0.00036	4.92E-02
Zxdb	75.643	0.496	0.172	0.00016	3.12E-02
Lbr	368.007	0.454	0.140	4.90E-05	1.58E-02

Mxd1	229.890	0.436	0.146	0.00011	2.34E-02
Atp13a2	651.094	0.435	0.148	0.00014	2.88E-02
Tmem164	487.919	0.429	0.148	0.00016	3.13E-02
Ogfrl1	725.001	0.421	0.130	5.56E-05	1.64E-02
Slc9a9	254.112	0.381	0.143	0.00034	4.91E-02
Lyn	1869.741	0.358	0.140	0.00031	4.65E-02
Hps3	274.856	0.355	0.104	2.55E-05	1.18E-02
Zdhhc20	600.596	0.311	0.114	0.00031	4.70E-02
Fbxl14	355.235	0.292	0.095	8.87E-05	2.01E-02
Tbc1d14	798.027	0.220	0.083	0.00036	4.92E-02
Grb2	2105.647	0.181	0.071	0.00035	4.92E-02
Pmm2	1362.557	-0.192	0.074	0.00037	4.94E-02

Supplementary Table 2. Immune cell gene signatures from PanglaoDB.

Cell Type	Gene signature (PangLaoDB)
B_cells	*Cd2, Cd5, Ms4a1, Cr2, Cd22, Cd40, Cd69, Cd70, Cd79a, Cd79b, Cd80, Cd86, Tnfrsf9, Sdc1, Tnfsf4, Tnfrsf13b, Tnfrsf13c, Pdcd1, Ighm, Rasgrp3, Ltb, Fli1, Cd14, Sema6d, Lair1, Ifit3, Igll1, Dntt, Mme, Spn, Cd19, Cd27, B3gat1, Cd72, Pax5, Jchain, Mzb1, Ly6d, Fcmr, Bank1, Edem1, Vpreb3, Pou2af1, Creld2, Derl3, Ralgps2, Fchsd2, Pold4, Tnfrsf17, Hvcn1, Fcrla, Edem2, Blnk, Txndc11, Btla, Smap2, Fkbp11, Sec61a1, Spcs3, Spib, Eaf2, Cxcr4, Birc3, Iglc1, Il21r, Igkc, Vpreb1, Klhl6, Slamf6, Bst1, Msh5, Dok3, Bach2, Pxk, Ighg1, Cd38, Ptprc, Ebf1, Bcl11a, Ccr7, Cd55, Cd74, Cd52, Tlr9, Swap70, Hmga1*
B_cells_memory	*Cd38, Cd80, Cd84, Cd86, Nt5e, Pax5, Ptprc, Spib, Adam28, Aim2, Alox5, Bach2, Bank1, Blk, Ccr6, Cd180, Cd19, Cd22, Cd27, Cd37, Cd69, Cd72, Cd79a, Cd79b, Cr2, Cxcr5, Dennd5b, Fcgr2b, Frk, Gng7, Hhex, Ighm, Igkc, Il7, Irf8, Ltb, Ly86, Mbl2, Ms4a1, Nmbr, P2rx5, Pnoc, Ptprcap, Ralgps2, Rasgrp2, Sik1, Sit1, Slc12a1, Sp140, Stap1, Tmem156, Tnfrsf13b, Tnfrsf17, Traf4, Vpreb3, Zbtb32*
B_cells_naive	*Cd19, Cd2, Cd72, Il2ra, Mme, Abcb4, Adam28, Bach2, Bank1, Bcl7a, Bend5, Blk, Braf, Cd180, Cd22, Cd37, Cd69, Cd79a, Cd79b, Cr2, Cxcr5, Eaf2, Fcgr2b, Frk, Hhex, Ighm, Igkc, Irf8, Ltb, Ly86, Mep1a, Mical3, Ms4a1, Nmbr, P2rx5, F630111L10Rik, Pnoc, Ptprcap, Ralgps2, Rasgrp2, Sell, Sik1, Slc12a1, Spib, Stap1, Ugt1a10, Vpreb3, Tnfsf8, Foxp1, Mitf, Cd52, Tnfrsf13c, Stag3, Birc3, Cd74*
Basophils	*Cd69, Il3ra, Ccr3, Enpp3, Fcer1a, Tlr4, Ltc4s, Cpa3, Itgal, Itgam, Itgax, Itgb7, Il4, Ms4a2, Ms4a3, Hdc, Gata2, Ccl3, Cd33, Cd44, Cdh1, Htr1b, Igfbp7, Dnase2b, Fads2, Gpc4, Grm6, Acss2, Adora2b, Aqp9, Bace1, Bmp4, Cables1, Ccl4, Cebpa, E2f8, Fads1, Far2, Fbxo9, Gabbr1, Gpr183, Hgf, Ifitm1, Inf2, Jazf1, L1cam, Lama5, Mboat1, Nfil3, Npl, Osbpl5, Otub2, P2ry1, Pdk3, Pdzd4, Perp, Plekhg5, Plg, Rnf39, Slc26a3, Slc26a8, Sncaip, St6galnac5, Tulp3, Tec, Unc93a, Wrn, Id1*
Cholangiocytes	*Sstr2, Slc4a2, Sctr, Krt7, Kcnn2, Itgb4, Ggt6, Cftr, Cckbr, Aqp4, Aqp1, Pigr, Ggt1, Jag1, Gpbar1, Ggt7, Krt19, Onecut2, Alpl, Hnf1b, Alb, Agr2, Tff3, Tff1, Sox9, Epcam, Cldn4, Mmp7, Tff2, Scgb3a1, Fxyd2, Defb1, Lcn2, Cxcl1, Lgals2, Tacstd2, Elf3, Spp1, Muc5b, Lgals4*
Dendritic_cells	*Il6, Cd86, Cd83, Cr2, Tlr9, Lamp3, Trem2, Fabp4, S100a9, Arg1, Clec10a, Dnase1l3, Clec9a, Etv6, Cd163, Cxcr4, Vsig4, Nr4a3, Ccr7, Traf1, Relb, Batf3, Ccl22, Slamf7, Xcr1, Cxcl16, Scimp, Fcgr2b, Fgd2, Rab7b, Naaa, Hck, Cd180, Hfe, Ccr2, Ryr1, Itgae, Sema4a, Dpp4, Slamf8, Cxcr3, Btla, Flt3, Tlr3, Itgax, Gpr132, Adam19, Ap1s3, Ass1, Adgrg5, Gpr68, Kit, Kmo, P2ry10, Rab30, Zbtb46, S100a4, Clec7a, Aif1, Lst1, Ctss, Irf8, Adgre1, Ccl17, Cd14, Cd207, Cd8a, Cx3cr1, Itgam, Ly75, Pdcd1lg2, Ptprc, Sirpa, Axl, Ppp1r14a, Cd22, Dab2, S100a8, Vcan, Anxa1, Fcer1a, Cadm1, Camk2d, Lgals3, Napsa, Plbd1,*

	Rnase6, Plac8, Slc11a1, Pdpn, S100b, Cd28, Ppl, Slurp1, Serpinb9
Eosinophils	*C3ar1, Fut4, C5ar1, Cxcr3, Ccr1, Ccr3, Cd244a, Ptgdr2, S100a9, Pglyrp1, Ltc4s, Epx, Cd69, Il5, Prg2, Prg3, Cpa3, Ikzf2, Ccl11, Ccl5, Csf2, Il5ra*
Gamma_delta_T_cells	*Ptgds, Gzmb, S100b, Nkg7, Prf1, Hopx, Cst7, Klrd1, Ctsw, Spon2, Ifitm1, Gzma, Cd247, Clic3, Cd7, Adgrg1, Ccl5, Stmn1, Hmgb2, Tyms, Mki67, Ube2c, Tuba1b, Aspm, Cenpa, Top2a, Pcna, Aurkb, Birc5, Nusap1, Troap, Cenpf, Ccnb1, Trgv7, Trdv5*
Hepatic_stellate_cells	*Adamts13, Cygb, Pparg, Pdgfra, Rgs5, Ppara, Foxf1, Alb, Nr1h4, Sema7a, Pnpla3, Wt1, Fgf10, Gfap, Ngfr, Vegfa, Acta2, Myb, Des, Fap, Slc8a1, Reln, Syp, Vcl, Timp1, Col1a1, Tagln, Col1a2, Col3a1, Sparc, Rbp1, Dcn, Myl9, Tpm2, Bgn, Igfbp7, Igfbp3, Olfml3, Igfbp6, Ccl2, Colec11, Hgf*
Hepatoblasts	*Afp, Alb, Cebpa, Krt14, Dlk1, Cdh1, Foxm1, Ggt1, Hnf4a, Prox1, Map2k4, Smad5, Krt18, Krt8, Hnf1b, Hhex, Met*
Hepatocytes	*Fosl1, Eppk1, Ucp2, Gck, Lrp5, Slc10a1, Nos2, Atp7b, Hnf4a, Gjb2, Fgfr4, Prox1, Crp, Slc2a2, Tfr2, Kif13b, Lipc, Vdr, Asgr1, Arg1, Afp, Alb, Otc, Ugt1a1, Zhx2, Hhex, Foxa3, Foxa2, Foxa1, Cyp7a1, Cyp2e1, Cyp1a2, Cebpa, Cdh1, Gpc3, Ahr, Cyp1a1, Nr1i3, Cps1, Gls2, Pck1, Tat, Wt1, Prrg4, Sult1a1, Apoh, Ctnnb1, Abcc3, Fgb, Aqp3, Plscr1, Fga, Apob, Ang, Anxa13, Sat2, Sfrp5, A1cf, Apoa1, Bnip3, Fgl1, Pah, Serpina6, Apoa2, Azgp1, Fgg, Apoc3, Defb1, Tm4sf4, Gc, Ambp, Orm1, Ttr, Hal, Ass1, Hmgcs1, Acss2, Tm7sf2, Sec16b, Slbp, Rnd3, Bche, Ghr, Aldh6a1, Masp2, Hamp, Glul, Acly, Asl, Tmem97, Cp, Slpi, Acat3, Tm4sf5, Msmo1, Lepr, Rcan1, Ar, Plin1, Rpp25l, Hsd11b1, Apom, Tkfc, G0s2, Pon3, Ttc36, Golt1a, Fst, Mcc, Aqp9, Gsta1, Nnt, Saa4, Mrps18c, Ociad1, Apoa5, Entpd5, C4b, Eid2, Atic, Serpinh1, Samd5, Grb14, Abcd3, Rhob, Epb41l4b, Gpat4, Sptbn1, Sdc2, Phlda1, Wtap, Acadm*
Kupffer_cells	*Tnf, Mpo, Vsig4, Prok2, Stard5, Msr1, Tlr9, Ppara, Trem1, Slc11a1, Tlr4, Mmp13, Cd14, Vdr, Chit1, Osm, Ppard, Clec4f, Adgre1, Il1b, Slc40a1, Timd4, Ifi204, Slc15a3, Dnase1l3, Marco, Hfe, Cd38, Cd163, Ccr5, Clec1b, Clec4g, Gpihbp1, Folr2, Pltp, Irf7, Spic, Csf1r, C1qc, C1qa, C1qb, Clec4e*
Macrophages	*Itgal, Itgam, Cd14, Fut4, Cd33, Cd80, Cd86, Cd163, Ccr5, Tlr2, Tlr4, Adgre1, Gpr34, Trem2, Fabp4, S100a8, Cpm, Chit1, F13a1, Cx3cr1, Cxcl16, Tgfbr1, Slamf9, Scimp, Lilra5, Cd83, C3ar1, Stab1, Mrc1, Parp14, Fgd2, Rab7b, Rbpj, Slco2b1, Naaa, Egln3, Fgl2, Gpnmb, Clec4d, Adam8, Arl11, Mmp12, Lyve1, Pltp, Vsig4, Ms4a4a, Fpr1, Cd180, Gdf15, Rab20, Hfe, Tnf, Ccr2, Snx20, Fmnl1, Gpr132, Slamf7, Nceh1, Ccl24, C5ar1, Cd300a, Ccl7, Ccl2, Il1b, Irf5, Ahr, Myo1g, Dusp5, Gpr171, Ccr7, Dnase1l3, Cxcl1, Samsn1, Nr4a3, Ccl22, S100a4, Mmp9, Hilpda, Nrp2, Slc37a2, Ctsk, Cd36, Lpcat2, Hpgds, Ifnar2, Ms4a7, Slc11a1, Hpgd, Ccl3, Clec7a, Cd5l,*

	Ccl5, Cyth4, Cd3e, Cd19, Cd74, Csf1r, Lgals3, Cd68, Ucp2, Treml4, Fgr, Cybb, Cd200, Cd200r1, Gata6, Itgax, Pparg, Tyrobp, Rgs1, Dab2, P2ry6, Maf, Aif1, Clec10a, Adgre5, Slc15a3, Cyp27a1, Slc7a7, Runx3, Syk
Mast_cells	*Kit, Enpp3, Il2ra, Il17a, Crh, Hsd11b1, Cd274, Cxcr2, Cxcr4, Ccr3, Ccr5, Ltc4s, Gpr34, Mgst2, Ache, Cpa3, Cpa1, Cma1, Hnmt, Cfp, Rab27b, Sla, Il1rl1, Gnai1, Cfd, Osbpl8, Adora3, Hs3st1, Csf2rb2, Dapp1, Slc6a4, Maob, Csrnp1, Cyp11a1, Rgs1, Plek, Hdc, Tpsb2, Tph1, Cd55, Rgs13, Tpsg1, Socs1, Btk, Il4, Ms4a2, Vwa5a, Fcer1a, Mcemp1, Milr1, Ccl2, Creb3l1, Ednra, Hs6st2, Kcne3, Meis2, Mrgprb1, Plau, Adamts9, Chst1, Cobl, Ctsg, Dnm3, Grik2, Hpgds, Hs3st3a1, Papss2, Pcbd1, Pcp4l1, Rnf128, Slc29a1, Slc45a3, Stard13, A4galt, Acer3, Armcx3, Atl2, Bbs10, Bmpr2, C2, Ccdc141, Ccl7, Cdc42bpa, Cdh9, Copz2, Zdhhc13, Unc13b, Trank1, Tiam2, Tcf7l1, Stk32b, St6galnac3, Socs2, Spred1, Smpx, Smarca1, Slc7a5, Slc4a4, Slc31a2, Sgce, Ror1, Rnf180, Rbfox1, Rapgef2, Pde1c, Pcdh7, Optn, Oaf, Neo1, Ndst2, Nceh1, Mlph, Mitf, Mfge8, Lsamp, Lrrc66, Lima1, Krt4, Kdelr3, Ids, Hspa13, Hs2st1, Gpm6a, Gp1ba, Gnaz, Ext1, Esyt3, Eno2, Enah, Dusp1, Dgki, Ddc, Dclk3*
Megakaryocytes	*Itga2b, Gp1ba, Itgb3, Mpl, Lat, Plek, S100a9, S100a8, Pf4, F13a1, Cd9, Epor, Cxcr4, Gp5, Cxcr1, Cxcr2, Slamf1, Pde6b, Matk, Spon2, Tlr9, Kif5b, Gata1, Fli1, Prox1, Syp, Tspan9, Rgs18, Treml1, Gata2, Vwf, Alox12, Selp, Nfatc1, Cib1, Plk3, Mafg, Il2ra, Pecam1, Pparg, Il6, Il21r, Lrp1, Ache, Vegfa, Tgfb1, Gp9*
Monocytes	*Rgs1, Cd7, Tet2, Cd40, Dysf, Cmklr1, Mefv, Hck, Padi4, Ghsr, Itgax, Sele, Tlr4, Ar, Cxcr4, Cd86, Ccr2, Tnfrsf14, Mgmt, Cd14, Cd33, Itgam, Ace, Fut4, Icam1, Sell, Cd163, Fcgr2b, Acp5, Mrc1, Tnf, Plau, Irf7, Plscr1, Mx2, Il1rn, Ifit1bl1, Ido1, Ifit3, Tnfsf10, Cxcl10, S100a9, S100a8, S100a4, Clec7a, Csf3r, Ifi204, Zfp36l2, Lta4h, Clec12a, Cd48, Prtn3, Vcan, Ifitm3, Fn1, Adgre1, Cd44, Csf1r, Cx3cr1, Itgal, Pecam1, Ptprc, Spn, Psap, Rhoc, Pilra, Nfkbiz, Naaa, Ly6e, Lyn, Ms4a7, Cebpb, Cd68, Ifi30, Rgs2, Lst1, Spi1, Tymp, Fgl2, Pycard, Lyst, Ccl3, Il1b, Cfp, Cd36*
Myeloid-derived_suppressor_cells	*Itgam, Fut4, Cd33, Ccr2, Icam1, Cd80, Cxcr1, Adgre1, S100a9, S100a4, S100a8*
Natural_killer_T_cells	*Il12rb2, Il17ra, Il12rb1, Klrb1a, Rora, Itgb2, Egr1, Map3k8, Nr4a1, Gzmb, Tnfrsf8, Prf1, Il2rb, Zbtb16, Tbx21, Gata3, Ncam1, S1pr1, Styk1, Tcf7, Slamf7, Cxcr4, Cd44*
Neutrophils	*Mme, Itgam, Itgax, Cd14, Fut4, Pecam1, Cd33, Sell, C5ar1, Cxcr1, Cxcr2, Tlr2, Mylk, S100a9, Mpo, Ceacam1, Crp, Lcn2, Defa17, Ltf, Elane, S100a8, Il1b, Cxcl1, Hp, Ccl3, Hdc, Mmp8, Lrg1, Osm, Mmp9, Pilra, Clec4d, Clec4e, Asprv1, Ccrl2, Ccr1, Ncf1, Trem1, Sorl1, Arg2, Bst1, Il1r2, Cfp, Adam8, Cd177,*

	Ptgs2, Oas3, Prtn3, Ctsg, Camp, Slc1a5, Snx20, Adpgk, Pstpip1, Lyst, Dock8, S100a4, Nlrp3, Csf3r
NK_cells	*Itgam, Itgax, Cd69, Klrd1, Il2rb, Klrb1a, Cd244a, Klrk1, Slamf7, Ncr1, Slamf6, Kit, Cd27, Klrc1, Nkg7, Gzmm, Ctsw, Hmbox1, Ahr, Prf1, Ccl4, Sema6d, Fhl2, Cd2, Cd7, Cd3g, Cd33, Dpp4, Lat, Pcdh15, Ccl5, Gzma, Gzmb, Cma1, Dusp2, Txk, Dok2, Cst7, Hsd11b1, Samd3, Tbx21, Chsy1, Cxcr4, Il18r1, Serpinb9, Dock2, Sh2d2a, S100a4, Xcl1, Gzmk, Ifng, Ccl3, Csf2, Il2rg, Tgfb1, Kir3dl2, Klrg1, Adamts14, Itga2, Styk1, Clec2e, Cd247, Zbtb16, Spon2, Hopx, Cd8a*
Nuocytes	*Icos, Il1rl1, Rora, Il17rb, Il7r, Crlf2, Arg1, Gata3, Ptgds, Bcl11b, Il5, Il13*
Plasma_cells	*Sdc1, Irf4, Cd38, Prdm1, Cd93, Cd27, Enpp1, Klf4, Ms4a4a, Pias1, Cycs, Diablo, Htra2, Ms4a1, Cd9, Cd19, Cd37, Cd40, Cd74, Cxcr4, Gzmb, Tnfrsf17, Tnfrsf13b, Fcgr2b, Ccr10, Abcb9, Ampd1, Angpt4, Cd79a, Dennd5b, Eaf2, Gng7, Gpr25, Ighe, Ighm, Igkc, Kcna3, Kcng2, Lime1, Manea, Mast1, Mroh7, Mzb1, P2rx5, Pax7, Pdk1, Pnoc, Rasgrp3, Rgs13, Sik1, Spag4, St6galnac4, Tgm5, Tmem156, Zbp1, Ighg1, Iglc1, Jchain, Ssr4, Il16, Bhlha15, Ppib*
Plasmacytoid_dendritic_cells	*Cd4, Cd8a, Cd40, Cd80, Cd83, Cd86, Il3ra, Ccr7, Cxcr3, Tradd, Arhgap9, Lamp5, Gpr183, Map3k2, Tnfsf13b, Ptcra, Ern1, Etv6, Map3k8, Spib, Gzmb, Cxcr4, Pacsin1, Tlr7, Tlr9, Irf7, Irf8, Igll1, Cux2, Mctp2, Klk1, Blnk, Grm8, Prkca, Cdh1, Il17rb, Card6, Clec4g, S100a8, Jchain, Serpinf1, Itm2c, Tcf4, Bcl11a, Mzb1, Derl3, Zfat, Nrp1, Slc15a4, Ccr9, Irf5, Bst2*
Red_pulp_macrophages	*Hmox1, Treml4, Cd86, Nr1h3, Slc11a1, Adgre1, Mertk, Ccr3, Spic, Slc40a1, Vcam1, Siglec1*
T_cells	*Cd3d, Cd3g, Cd3e, Hopx, Ccl3, Ccl4, Syt3, Notch3, Sema6d, Dkk3, Ifit3, Cerk, Pmch, Cd4, Ltb, Cxcr6, Il7r, Satb1, Lef1, Itk, Ptprcap, Gem, Cd7, Maff, Tgif1, Rora, Tnfaip3, Crem, Pxdc1, Nabp1, Fam110a, Cd69, Cxcr4, Trbc1, Bcl2, Cytl1, Cd2, Ctsw, Ptpn22, Txk, Gdpd3, Traf1, Il2ra, Batf3, Lag3, Gzmk, Gzmb, Sh2d1a, Myo1g, Fmnl1, S1pr4, Cd247, Gimap5, Cd28, Cd160, Rhoh, Klrb1a, Ccr2, Il2rb, Mbd2, Icos, Il18r1, Tnfrsf4, Ccl20, Clec2e, Cd8a, Cd6, S100a4, Ccl5, Lck, Cd81, Thy1, Lat, Skap1, Tcf7, Ccr7, Myb, Gzma, Junb, Dusp2, Ifng, Cd52, Braf*
T_cytotoxic_cells	*Cd2, Cd27, Cd28, Cd5, Cd69, Cd8a, Gzmb*
T_follicular_helper_cells	*Bcl6, Cd3d, Cd4, Cd84, Slamf1, Cd40lg, Cxcr5, Tnfsf4, Icos, Pdcd1, Cd200, P2rx7*
T_helper_cells	*Ifng, Il2, Tbx21, Dpp4, Klrd1, Cxcr3, Ccr1, Ccr5, Tnfsf11, Havcr2, Il18r1, Ltbr, Tnf, Lta, Gata3, Csf2, Il4, Il5, Il6, Il10, Il13, Ccr8, Cxcr4, Ccr3, Ccr7, Icos, Ptgdr2, Havcr1, Il1r2, Irf4, Stat6, Il17a, Il17f, Il21, Il22b, Rora, Rorc, Cd38, Klrb1a, Ahr, Ccr10, Cd3d, Cd4, Ccr4, Ccr6, Il9, Cd2, Cd28, Cd5, Il1rl1, Foxp3, Cd3g, Tnfrsf4, Cd40lg, Il17rb, Hpgds, Nfatc2, Nfkb1, Nr3c1, Pparg*
T_memory_cells	*Sell, Ccr7, Cd4, Ccr5, Cd69, Itgb5, Bcl2, Grap2, Tbc1d10c, Cytip, Kpna4, Cd247, Epsti1, Apbb1ip, Cd27, Rhoh, Gimap1, Acp5, Ikzf1, Sp100, Dgka, Il7r, Itgb7, Nkg7, Cd28, Bin2,*

	Rapgef6, Arhgef1, S100a9, Arhgap15, Cd3e, Skap1, Gimap4, Cd2, Ptprc, Lat, Tcf7, Lef1, Arhgap45, Satb1, Ptprcap, S100a8, Ltb, Lck, Trbc1, Cd3g, Cd3d, Cd7, Cxcr6, Icos, Ccr6, Itgae, Enpp1
T_regulatory_cells	*Foxp3, Ikzf2, Cd4, Il2ra, Entpd1, Sell, Nt5e, Itgae, Tnfrsf4, Ctla4, Ccr4, Lag3, Izumo1r, Cngb1, Tnfrsf18, Maf, Il10, Il1r1, Itgb8, Lrrc32, Folr1*

Supplementary Table 3. LAM 22-gene signature

**Gene**
Trem2
Gpnmb
Cd63
Lgals3
Cd9
Fabp5
Cd36
Lpl
Atp6v0d2
Slc37a2
Mmp12
Tnip3
Akr1b8
Actn1
Gngt2
Dhrs9
Cd300lb
Plk2
Fblim1
Alas1
Msmo1
Ggta1

Supplementary Table 4. Pairwise comparison of D4 versus D7 cDC1 subclusters. Significant DEGs (adjusted p-value < 0.05) shown.

Gene	p_val	avg_log2FC	pct.1	pct.2	p_val_adj
Slc25a33	2.30E-08	4.304	0.443	0.033	3.14E-04
Il1b	1.55E-08	3.110	0.774	0.475	2.12E-04
Dusp2	9.13E-07	2.478	0.575	0.23	1.25E-02
Arl13b	1.82E-06	2.416	0.5	0.131	2.49E-02
Mpp7	1.70E-07	2.170	0.774	0.459	2.32E-03
Csrnp1	5.17E-07	2.090	0.547	0.148	7.06E-03
Tuba1a	8.11E-07	2.072	0.632	0.295	1.11E-02
Cd44	1.21E-08	1.884	0.915	0.77	1.65E-04
Tuba1c	1.33E-08	1.879	0.698	0.262	1.82E-04
Fbxo34	1.10E-06	1.788	0.557	0.164	1.50E-02
1700016P03Rik	1.03E-08	1.718	0.764	0.262	1.41E-04
Csf2rb	9.56E-07	1.716	0.745	0.426	1.31E-02
Dot1l	5.48E-08	1.709	0.736	0.328	7.49E-04
Taf7	1.02E-06	1.688	0.566	0.164	1.40E-02
Gpr65	3.30E-07	1.688	0.821	0.59	4.52E-03
Trib1	1.77E-07	1.682	0.717	0.361	2.42E-03
Tubb6	2.42E-06	1.651	0.651	0.311	3.31E-02
Crem	1.71E-07	1.640	0.726	0.295	2.34E-03
Lrrc8c	1.33E-08	1.617	0.906	0.738	1.82E-04
Got1	1.15E-06	1.603	0.755	0.443	1.57E-02
Lncpint	1.11E-07	1.556	0.877	0.623	1.51E-03
Atp6v0d1	7.59E-07	1.555	0.689	0.361	1.04E-02
Hspd1	3.24E-06	1.547	0.915	0.738	4.42E-02
Arl5b	8.69E-07	1.540	0.66	0.311	1.19E-02
Gm26532	8.24E-07	1.517	0.679	0.311	1.13E-02
Dnajb1	1.33E-06	1.516	0.887	0.689	1.82E-02
Snhg12	1.57E-06	1.502	0.708	0.344	2.15E-02
Ext1	1.12E-07	1.492	0.906	0.738	1.53E-03
Nr4a3	1.83E-07	1.459	0.811	0.443	2.51E-03
Gm47283	5.95E-07	1.447	0.868	0.672	8.14E-03
Tob2	1.01E-07	1.431	0.83	0.459	1.38E-03
Gpd2	9.19E-09	1.417	0.887	0.557	1.26E-04
Dusp1	2.53E-08	1.409	0.981	0.82	3.46E-04
Erlin1	1.28E-08	1.392	0.858	0.475	1.75E-04
Gpcpd1	1.15E-06	1.366	0.943	0.82	1.58E-02
Nr4a2	7.63E-09	1.339	0.943	0.607	1.04E-04
Hspa1b	2.29E-07	1.323	0.934	0.852	3.12E-03
Hspa9	2.59E-07	1.295	0.783	0.377	3.54E-03
Azin1	2.90E-06	1.288	0.717	0.41	3.97E-02
Btaf1	2.13E-06	1.285	0.83	0.557	2.92E-02
Lmnb1	1.97E-07	1.253	0.84	0.59	2.70E-03

Fyn	1.11E-08	1.231	0.934	0.885	1.51E-04
Brd2	3.15E-06	1.220	0.858	0.639	4.30E-02
Ier5	1.87E-07	1.203	0.962	0.869	2.55E-03
Ddx21	1.22E-06	1.173	0.821	0.574	1.66E-02
Nudc	1.39E-06	1.146	0.858	0.656	1.90E-02
Skil	4.96E-08	1.132	0.991	0.82	6.78E-04
Ago2	7.82E-07	1.130	0.868	0.508	1.07E-02
Stip1	2.80E-06	1.122	0.708	0.295	3.83E-02
Jun	6.76E-08	1.107	0.972	0.934	9.24E-04
Cacybp	3.51E-06	1.084	0.849	0.623	4.80E-02
Litaf	2.05E-06	1.034	0.868	0.689	2.81E-02
Picalm	1.70E-07	1.004	0.991	0.951	2.33E-03
Ubxn4	1.53E-06	0.958	0.896	0.705	2.10E-02
Hspe1	1.55E-06	0.945	1	1	2.12E-02
Eif5	1.03E-06	0.869	0.962	0.869	1.41E-02
Ncl	6.65E-07	0.830	0.991	0.951	9.09E-03
Rel	2.29E-06	0.802	0.981	0.918	3.13E-02
Pafah1b1	5.36E-07	0.791	0.972	0.836	7.32E-03
Srsf7	4.09E-07	0.771	0.981	0.836	5.59E-03
Eif3c	1.21E-06	0.744	0.962	0.852	1.65E-02
Rps27l	4.55E-07	0.724	0.962	0.951	6.22E-03
Cd83	4.64E-07	0.719	1	0.918	6.34E-03
Sub1	1.59E-08	0.672	1	1	2.17E-04
Calm1	1.25E-06	0.631	1	1	1.71E-02
Chchd2	3.01E-07	0.616	0.991	1	4.11E-03
Ywhaz	2.08E-06	0.614	1	0.984	2.85E-02
Ddx5	1.04E-06	0.543	1	1	1.42E-02
Rbm39	8.46E-07	0.512	1	1	1.16E-02
Ppt1	4.25E-07	-0.555	1	1	5.82E-03
Itm2b	2.42E-07	-0.566	1	1	3.30E-03
Clic1	1.90E-06	-0.622	0.981	1	2.60E-02
Ly6e	9.38E-08	-0.636	0.991	0.984	1.28E-03
Pid1	7.90E-07	-0.811	0.906	0.984	1.08E-02
Dpp4	2.50E-06	-0.857	0.745	0.967	3.41E-02
Pip4k2a	5.81E-07	-0.881	0.925	1	7.94E-03
BC028528	2.83E-06	-0.883	0.792	0.934	3.87E-02
Mef2a	1.68E-06	-0.884	0.811	0.951	2.30E-02
Fmnl2	1.16E-07	-0.888	0.943	0.984	1.58E-03
Slamf7	7.55E-08	-0.910	0.83	1	1.03E-03
Rnase6	3.24E-08	-0.975	0.745	0.951	4.43E-04
Ly86	8.36E-08	-0.978	0.792	1	1.14E-03
Fgd2	2.46E-08	-1.028	0.745	0.951	3.36E-04
Cbl	3.12E-07	-1.076	0.67	0.902	4.27E-03
Plin2	8.54E-09	-1.078	0.726	0.951	1.17E-04
Slfn2	2.04E-06	-1.087	0.509	0.82	2.79E-02
Lrch1	1.48E-06	-1.131	0.632	0.82	2.03E-02

Ncoa1	1.60E-06	-1.146	0.519	0.902	2.18E-02
Arhgap5	1.35E-08	-1.215	0.679	0.934	1.84E-04
Ppm1h	1.46E-06	-1.216	0.613	0.82	1.99E-02
Srgap3	1.06E-07	-1.230	0.651	0.885	1.44E-03
Fchsd2	2.59E-07	-1.344	0.764	0.869	3.53E-03
Ikzf1	6.48E-08	-1.382	0.462	0.82	8.85E-04
Nrg2	1.48E-06	-1.390	0.311	0.672	2.02E-02
Supt20	1.84E-06	-1.426	0.585	0.82	2.51E-02
Txnip	7.13E-07	-1.438	0.368	0.738	9.75E-03
Gm36723	9.73E-08	-1.469	0.755	0.918	1.33E-03
Zfp710	1.14E-07	-1.492	0.557	0.803	1.55E-03
Cdc14a	2.49E-06	-1.512	0.472	0.738	3.41E-02
Plcl2	2.65E-07	-1.589	0.302	0.672	3.62E-03
Rgs18	1.61E-06	-1.605	0.17	0.525	2.20E-02
Fkbp5	1.90E-06	-1.627	0.292	0.607	2.60E-02
Rmdn1	4.60E-07	-1.864	0.179	0.541	6.29E-03
Slc12a9	3.25E-06	-1.878	0.236	0.557	4.44E-02
Pik3ip1	2.01E-06	-1.890	0.132	0.459	2.75E-02
Sult1a1	4.56E-07	-1.967	0.311	0.623	6.23E-03
Zfp740	9.19E-07	-1.995	0.151	0.492	1.26E-02
Gm35188	2.43E-06	-2.020	0.123	0.443	3.32E-02
Sh2d3c	7.35E-08	-2.104	0.17	0.541	1.00E-03
Fbxl20	1.88E-07	-2.352	0.113	0.459	2.57E-03
Arl11	2.17E-06	-2.868	0.038	0.295	2.97E-02

Supplementary Table 5. Pairwise comparison of GAN-Adj.NT versus GAN cDC2 subpopulations. Significant DEGs (adjusted p-value < 0.05) shown.

Gene	p_val	avg_log2FC	pct.1	pct.2	p_val_adj
Slc25a33	8.87E-09	2.161	0.272	0.063	1.21E-04
Marcksl1	3.36E-06	1.589	0.592	0.464	4.59E-02
Sdc4	3.34E-08	1.582	0.35	0.126	4.56E-04
Arid5a	4.45E-08	1.310	0.447	0.198	6.08E-04
Sik3	9.07E-07	1.258	0.558	0.348	1.24E-02
Nfkbiz	3.49E-08	1.192	0.786	0.599	4.78E-04
Tob2	1.96E-08	1.126	0.641	0.406	2.67E-04
Sde2	8.27E-07	1.029	0.612	0.415	1.13E-02
Rilpl2	2.92E-07	1.007	0.655	0.444	3.99E-03
Fosl2	1.22E-06	1.007	0.578	0.357	1.66E-02
Zc3hav1	2.16E-06	0.950	0.772	0.628	2.95E-02
Relb	1.50E-08	0.939	0.587	0.295	2.05E-04
Sqstm1	1.81E-07	0.850	0.859	0.7	2.47E-03
Grasp	9.12E-07	0.824	0.675	0.459	1.25E-02
Ezr	3.43E-06	0.820	0.743	0.589	4.69E-02
Litaf	2.93E-08	0.813	0.83	0.662	4.00E-04
Dnajb1	1.62E-06	0.788	0.825	0.643	2.22E-02
Hspa1a	5.05E-08	0.785	0.966	0.913	6.90E-04
Egr1	4.47E-07	0.773	0.879	0.802	6.11E-03
Dusp2	2.78E-06	0.754	0.636	0.391	3.80E-02
Tiparp	1.19E-06	0.746	0.68	0.473	1.63E-02
Tagln2	8.98E-08	0.735	0.947	0.826	1.23E-03
Nr4a1	8.24E-08	0.704	0.927	0.836	1.13E-03
Ccnl1	1.40E-07	0.673	0.859	0.734	1.91E-03
Pnrc1	2.04E-08	0.616	0.942	0.918	2.79E-04
Hspa5	1.30E-07	0.598	0.985	0.937	1.77E-03
Pim1	6.37E-07	0.590	0.922	0.845	8.71E-03
Hsp90aa1	2.46E-07	0.588	0.99	0.99	3.36E-03
Zfand5	1.81E-08	0.581	0.951	0.845	2.48E-04
Hsp90ab1	3.01E-08	0.432	1	1	4.12E-04
Btg2	1.11E-06	0.415	0.981	0.937	1.52E-02
Hspa8	3.10E-06	0.282	1	1	4.23E-02
Tmsb4x	5.31E-07	-0.258	1	1	7.25E-03
Itm2b	4.86E-08	-0.385	1	1	6.65E-04
Otulinl	9.63E-07	-0.491	0.874	0.947	1.32E-02
Cd48	2.71E-07	-0.537	0.806	0.884	3.70E-03
Slamf7	3.46E-06	-0.617	0.82	0.879	4.73E-02
Ap1s2	5.72E-08	-0.957	0.714	0.831	7.82E-04
Id2	1.94E-08	-1.214	0.602	0.763	2.66E-04

Supplementary Table 6. Curated B cell signatures

B cell signature	Genes
ABC signature in Malaria [10]	*Itgax, Rtn4r, Il10, Cd274, Il21r, Cd84, Pdcd1, Six3, Il12rb2, Slamf7, Grn, Fas, Pdcd1lg2, Boc, Arhgef5, Tbx15, Il10ra, Clec2e, Pdgfa, Plxnc1, Chrna7, Cd86, Cmtm3, Tox2, Cd200r1, Sema7a, Aicda, Hoxa10, Xpot, Itgb2, Tln1, Irx6, Sox5, Myo9b, Gdi2, E2f7, Hoxb6, Il2rb, Aire, Cmtm6, Cd72, Lamp1, Ebi3, Hoxa3, Satb2, Notch1, Slamf1, Ly6e, Cd164, Pou3f3, Pirb, Cd22, Lilrb4b, Cd72, Cxcr3, Itgax, Ccr6, Pdcd1, Fcrl5, Pirb, Pirb, Cd33, Cd22, Cd72, Cd200, Cd200r1, Itgax, Cd19, Ms4a1, Cd86, Il21r, Batf, Foxo1, Ikzf2, Nfatc2, Maf, Nfil3, Nr4a3, Tbx21, Zeb2, Crem, Atf3, Zbtb32, Jazf1, Litaf, Hopx, Mybl1, Tox, Prdm15, Slc1a6, Hck, Rhoc, Adamts13, Ppl, Iqcg, Timm13, Gpatch2, Nelfe, Ccr5, Slpi, Rhobtb2, Il6, Blvra, Cinp, Arhgef3, Cdca7, Rnf38, I830077J02Rik, Themis2, Nfkb1, Smco4, Ccdc167, Abhd14b, Map3k14, Ccdc115, Gpr34, Tbx21, Il27ra, Gpr82, Trib2, Adam8, Vps33a, Rsl24d1, Repin1, Prmt2, Slc25a1, Casp4, Eea1, Dusp4, Eif3i, Ccdc141, Tnfsf13, Tnfrsf13b, Slamf7, Cd19, Cd84, Cd86, Tnfrsf1a, Ifnar2, Il7, Il10ra, Ifnlr1, Itgb1, Itgb2, Itgb7, Itgax, Cd72, Fcgr2b, Fcrl5, Pirb, Pirb, Pirb, Cd33, Siglecg, Lcp2, Syk, Hck, Prkch, Ptprs, Ptpn2, Ptpn12, Ptpn22, Fas, Casp1, Casp9, Card11, Nlrc4, Gas7, Cdkn1a, Bcl11b, Egr2, Hoxa3, Hoxb7, Hoxc4, Nfatc2, Rnf144a, Sox4, Tbx21, Tox, Zeb2*
Pre-GC signature [11]	*Ccnd2, Psme2, Fcer2a, Ebi3, Ddx21, Syngr2, Hsp90ab1, Nop16, Cd83, Ncl, Eif5a, Dnph1, Gpx4, Myc, Ifitm2, Cdkn1a, Nhp2, Nfkbid, Nme2, Nolc1, Parvb, Lilra6, Rpl22l1, Eno1, H2-Aa, Traf4, Pa2g4, Srm, Bhlhe40, Mat2a, Fkbp4, Prmt1, Calr, Batf, Mrto4, Prdx1, Ifrd2, Acp5, Dusp2, Atic, Ebna1bp2, Irf4, Fabp5, Ybx1, Paics, Gm2a*
LZ signature [12]	*Ebi3, Egr3, Prdm1, Il4i1b, Dusp2, Egr2, Slamf1, Ccl22, Cd83, Hivep3, Akap5, Irf4, Myc, Sema7a, Nfkb2, Nfkbie, Lrrk2, Batf, Traf4, Ccr6, Il27ra, Slc39a14, Tnfaip3, Icam1, Kynu, Srm, Ipo4, Ccdc86, Samsn1, Egr1, Iqsec1, Bhlhe40, Nfkbia, Snx11, Ciita, Nab2, Slc2a3, Mettl1, Abhd6, Zc3h12a, Rcn1, Lmo2, Ly75, Nop16, Aldoc, Mybbp1a, Map3k8, Plek, Dctd, Gpr183, Nfkbid, Ier5, Ddit4, Rrp15, Dusp10, Ifrd2, Mfsd2a, Junb, Bmi1, Rpp40, Lgals9, Lta, Rsad1, Vasp, Gnl3, Ier2, Cd72, Kctd12, Tnf, Ptpn6, Prmt3, Syngr2, Pdcd4, Ifi30, Pprc1, Tsen2, Fasn, Tlcd1, Ddx21, Slc23a2, Abcb4, Il18r1, Utp15, Rrp12, Hsp90b1, Rhof, Adcy3, Cln5, Bcl3, Il2rb, Polr3e, Rilpl2, Stat6, Hspa5, Abcc4, Ctsh, Crem, Akap1, Ankrd33b, Ccr7, Ide, Cdk5r1, Rgs1, Il6st, Nat10, Tspan33, Gatm, Ralgds, Cd40, Nop2, Ppargc1b, Cyfip1, Stat1, Cyth4, Mdn1, Birc3, Irf2bp2, Plk3, Grn, Fyco1, Tmem109, Unc119, Calr, Mapkapk3, Tpp1, Pdia6, Pou2f2, Il2rg, Dhcr7, Nolc1, Nr4a1, Ampd3, Extl2, Zbtb8a, Apex1, Alcam, Relb, Bid, Psme2, Aff1, Ctsc, Per1, Jarid2, Arhgap24, Aprt, Fam162a, Sub1, Cd86, Kdm6b, Gpr65, Phlda1, Zfp36l1, Tnfrsf18, Slc15a3, Ccdc107, Lck, Manf, Nop56, Erp44, Znhit6, Insig1, Dhodh, Tapbp, Psmg4, Top1mt, Fyn, Zmiz2, Pnpo, Pes1, Ltv1, Ndufaf4, Midn, Chd1, Cd82, Reep3, Clcf1, Hspe1, Naf1, Trnt1, Urb1, Orai1, Slc25a19, Tmc6, Ssr4, Kat2a, Pdia4,*

	Arid3b, Invs, Cd320, Cad, Cr2, Dctpp1, Tbl3, Runx3, Mast3, St3gal5, Nhp2, Mreg, Evl, Ctsa, Rrp1b, Nol10, Actr3b, Gpr132, Akt1s1, Arid5a, Tnfsf9, Heg1, Rfx5, Slc3a2, Per2, Lpin1, Trmt61a, Tfrc, Grwd1, Nab1, Aldh18a1, Ankrd37, Slc2a6, Ikbke, Sipa1, Hook2, Eef1e1, Fabp5, Sigmar1, Rogdi, Tet2, Chchd4, Armcx3, Plscr1, Pex10, Klf10, G6pc3, Zswim6, Cyba, Mtap, Tspan31, Dusp7, Slc16a6, Wdfy4, Creld2, Hvcn1, Noc2l, Nbeal2, Sdc4, Mogs, Phf1, Ppa1, Ube2d1, Immp2l, Trappc5, Ptpre, Rasal3, Txlna, Riok2, Pigyl, Ppp1r14b, Polr1a, Inf2, Rel, Tnfrsf13c, Traf3, Nfkbiz, Soat1, Wdr89
DZ signature [12]	*Gas2l3, Psrc1, Aicda, Kif20a, Nek2, Cenpe, Cdc20, Cenpf, Gpsm2, Aurka, Cxcr4, Cdc25c, Aspm, Ccnb2, Hmmr, Cdc25b, Dlgap5, Cenpa, Ifi205, Fam83d, Birc5, Ube2c, Anln, Cdkn3, Kif23, Prc1, Rell1, Hjurp, Ccnb1, Ect2, Pif1, Cdca8, Tpx2, Hspa2, Cdca3, Plk1, Depdc1b, Mnd1, Tacc3, Troap, Ube2s, Nuf2, Hmgb3, Top2a, Arhgap11a, Cdca2, Rrm2b, Ckap2l, Rdm1, Ccnf, Polh, Ndc80, Spc24, Cep55, Cep70, Espl1, Spag5, Bub1, Ttk, Kif18a, Ncapg, Kif11, Kif18b, Aurkb, Kif2c, Kifc1, Ift57, Ccna2, Cdk1, Tmed6, Ckap2, Oip5, Ncaph, Ncapd2, Eri2, Foxp1, Racgap1, Mki67, Nusap1, Ska1, Lig4, Cit, Dnase1, Ckap5, Asf1a, Nfyb, Arhgap19, Ypel3, Hyls1, Pkp4, Incenp, Rassf6, Tcf3, Gnaz, G2e3, Gmfg, Ctnnal1, Poc1a, Fam81a, Psip1, Smarca4, Cep76, Cep135, Lpp, Bub1b, Neurl1b, Ccnd3, Bcl7a, Gtse1, Gkap1, Shcbp1, Kif20b, Syne2, Mybl1, Tram2, Suv39h1, Eif2ak3, Fam107b, Tcea1, Cenpl, Lrwd1, Kpna2, Phf3, Dhfr, Cdkl1, Rnf26, Ube2j1, Tgfbr1, Cdc27, Ccdc18, Rap1gap2, Calm3, Rbm38, Hp1bp3, Gfod1, Oaz2, Brwd1, Mad2l1, Kctd9, Cebpg, Tax1bp3, Bpgm, Klf6, Esco2, Nbr1, Arrdc3, Ccm2, Rere, Nde1, Xkrx, Dbf4, Ccng2, Clasp2, Dcaf12, Pola1, Cdkn2aipnl, Top1, Lin9, Rhoh, Ctdspl2, Traf5, Ube2g1*

Supplementary Table 7. Pairwise comparison of GAN versus LFD endothelial cells. Significant DEGs (adjusted p-value < 0.05) shown.

Gene	p_val	avg_log2FC	pct.1	pct.2	p_val_adj
Depp1	0.00E+00	6.622	0.693	0.03	0.00E+00
Nin	5.94E-09	0.663	0.384	0.257	1.01E-04
Gm26802	6.10E-09	-0.957	0.086	0.201	1.04E-04
Serpinb6a	6.18E-09	-0.353	0.853	0.863	1.05E-04
Prickle1	6.28E-09	-0.412	0.765	0.85	1.07E-04
Ttc8	6.36E-09	2.003	0.063	0.018	1.08E-04
Ophn1	6.38E-09	0.628	0.425	0.296	1.08E-04
Hspa1a	6.40E-09	-0.747	0.544	0.645	1.09E-04
Frzb	6.41E-09	4.106	0.017	0.001	1.09E-04
4833422C13Rik	6.47E-09	4.106	0.017	0.001	1.10E-04
Pcsk5	6.49E-09	4.734	0.024	0.002	1.10E-04
Itgb8	6.59E-09	3.428	0.024	0.002	1.12E-04
Ier5	6.70E-09	-0.605	0.488	0.601	1.14E-04
Bmx	7.00E-09	2.691	0.032	0.005	1.19E-04
Ywhab	7.10E-09	-0.338	0.821	0.871	1.20E-04
Arhgef40	7.19E-09	-1.298	0.082	0.191	1.22E-04
Nid2	7.38E-09	2.251	0.056	0.015	1.25E-04
9930111J21Rik2	7.62E-09	-0.938	0.257	0.383	1.29E-04
Hmg20a	8.28E-09	0.637	0.447	0.318	1.40E-04
Eci2	8.92E-09	0.722	0.311	0.195	1.51E-04
Xkr6	8.93E-09	0.808	0.337	0.222	1.52E-04
Slc26a10	8.96E-09	0.727	0.397	0.277	1.52E-04
Dand5	9.20E-09	-1.727	0.058	0.158	1.56E-04
Zfp91	9.41E-09	0.419	0.637	0.502	1.60E-04
Ifitm2	9.44E-09	-0.244	0.998	1	1.60E-04
Map3k1	9.56E-09	0.699	0.352	0.233	1.62E-04
Ndufb7	9.60E-09	-0.555	0.54	0.639	1.63E-04
Map4k4	9.72E-09	0.494	0.715	0.616	1.65E-04
Sept4	9.72E-09	0.812	0.272	0.163	1.65E-04
Tnrc6c	9.73E-09	0.551	0.473	0.343	1.65E-04
Atp6v0c	9.74E-09	-0.347	0.873	0.906	1.65E-04
Ybx1	9.76E-09	0.273	0.974	0.959	1.66E-04
Ei24	9.79E-09	0.654	0.343	0.221	1.66E-04
Zc3h12c	9.79E-09	1.053	0.166	0.082	1.66E-04
Serhl	1.02E-08	1.039	0.166	0.082	1.73E-04
Tnfsf10	1.03E-08	0.671	0.434	0.313	1.75E-04
Gm26532	1.04E-08	-1.072	0.158	0.28	1.77E-04
Fbxl7	1.05E-08	0.612	0.866	0.863	1.78E-04
Vps8	1.05E-08	0.649	0.356	0.232	1.78E-04
Selenoi	1.06E-08	1.044	0.173	0.087	1.80E-04
Pcnx	1.07E-08	0.579	0.49	0.365	1.81E-04

Tubb5	1.08E-08	0.381	0.832	0.767	1.83E-04
Knop1	1.09E-08	0.535	0.432	0.297	1.85E-04
Hyal2	1.09E-08	-0.490	0.587	0.677	1.85E-04
Fmn1	1.13E-08	0.761	0.397	0.277	1.92E-04
Nrxn3	1.15E-08	2.864	0.045	0.01	1.94E-04
Gm36756	1.17E-08	3.351	0.026	0.003	1.98E-04
Vps13c	1.20E-08	0.481	0.497	0.362	2.03E-04
Rnf19b	1.21E-08	-0.901	0.212	0.341	2.05E-04
Clint1	1.25E-08	0.546	0.555	0.423	2.12E-04
Svil	1.26E-08	0.931	0.35	0.239	2.14E-04
Selenok	1.27E-08	-0.324	0.806	0.877	2.16E-04
Sem1	1.28E-08	-0.286	0.894	0.937	2.18E-04
Rasgrp4	1.30E-08	2.307	0.045	0.01	2.20E-04
Dop1a	1.35E-08	0.812	0.253	0.147	2.28E-04
Clasp2	1.35E-08	0.612	0.492	0.366	2.29E-04
Hspa1b	1.37E-08	-0.214	0.413	0.579	2.32E-04
Gm4258	1.40E-08	1.405	0.127	0.057	2.38E-04
Plscr1	1.41E-08	0.966	0.16	0.078	2.39E-04
Plscr4	1.44E-08	0.530	0.562	0.438	2.44E-04
Lck	1.44E-08	-1.663	0.037	0.128	2.45E-04
Plk2	1.45E-08	0.368	0.788	0.669	2.47E-04
Ltc4s	1.49E-08	-1.075	0.149	0.267	2.53E-04
Ermp1	1.51E-08	0.955	0.194	0.103	2.57E-04
S1pr1	1.51E-08	0.440	0.674	0.55	2.57E-04
Stim2	1.59E-08	0.409	0.706	0.596	2.69E-04
Agpat5	1.59E-08	0.426	0.773	0.701	2.70E-04
Erg	1.61E-08	0.396	0.784	0.669	2.73E-04
Vamp8	1.69E-08	-0.641	0.374	0.505	2.86E-04
Bmp4	1.72E-08	2.476	0.048	0.011	2.92E-04
P2ry1	1.73E-08	-1.055	0.143	0.26	2.93E-04
H2-D1	1.78E-08	0.218	0.994	0.997	3.02E-04
Pgp	1.78E-08	0.717	0.324	0.207	3.02E-04
Gm41556	1.81E-08	-2.703	0.015	0.092	3.07E-04
Samd5	1.84E-08	3.271	0.032	0.005	3.12E-04
Fbxl17	1.88E-08	0.493	0.823	0.752	3.19E-04
Sec1	1.89E-08	2.521	0.035	0.006	3.20E-04
Hexa	1.90E-08	-0.580	0.436	0.55	3.23E-04
Gm26782	1.94E-08	1.579	0.073	0.024	3.30E-04
Aimp1	1.94E-08	0.558	0.449	0.318	3.30E-04
Stk3	1.95E-08	0.570	0.579	0.461	3.31E-04
Cdc42ep5	1.96E-08	0.616	0.32	0.2	3.33E-04
Zfp664	2.10E-08	0.603	0.339	0.215	3.56E-04
Ndufa6	2.12E-08	0.412	0.706	0.607	3.59E-04
Gm19461	2.13E-08	-2.584	0.011	0.085	3.61E-04
Large1	2.13E-08	0.681	0.505	0.383	3.61E-04
Ifit3b	2.14E-08	1.210	0.136	0.063	3.63E-04

Plp2	2.15E-08	2.095	0.056	0.015	3.66E-04
Cx3cr1	2.17E-08	-1.423	0.102	0.209	3.68E-04
Fosl2	2.17E-08	0.793	0.369	0.256	3.69E-04
Hmox1	2.18E-08	-0.707	0.374	0.492	3.70E-04
Gpatch8	2.20E-08	0.536	0.486	0.367	3.74E-04
Uqcrq	2.21E-08	-0.441	0.644	0.716	3.75E-04
Cox7a2	2.22E-08	-0.345	0.834	0.877	3.77E-04
Rc3h1	2.24E-08	0.496	0.6	0.467	3.81E-04
Kdelr2	2.27E-08	-0.478	0.629	0.708	3.85E-04
Pik3r3	2.34E-08	-0.989	0.205	0.325	3.97E-04
Arhgap35	2.34E-08	0.517	0.464	0.33	3.97E-04
Prcp	2.39E-08	0.803	0.378	0.266	4.06E-04
Cpd	2.53E-08	0.414	0.672	0.565	4.29E-04
Dmd	2.59E-08	0.672	0.419	0.305	4.40E-04
Cd55	2.62E-08	-0.224	0.94	0.975	4.45E-04
AV356131	2.66E-08	2.138	0.045	0.011	4.52E-04
Etv6	2.72E-08	0.615	0.603	0.491	4.62E-04
Gm11476	2.96E-08	-1.504	0.071	0.171	5.02E-04
Tgfbr3	2.97E-08	0.396	0.801	0.718	5.04E-04
Cd209b	2.98E-08	-2.844	0.015	0.09	5.05E-04
Hivep3	2.98E-08	0.510	0.663	0.542	5.05E-04
Serinc3	2.99E-08	0.209	1	0.995	5.08E-04
Gstm3	3.00E-08	1.766	0.024	0.003	5.10E-04
Plxna4os3	3.03E-08	3.222	0.024	0.003	5.14E-04
Jcad	3.05E-08	0.576	0.514	0.395	5.17E-04
Phlda1	3.07E-08	-1.069	0.104	0.216	5.21E-04
Dcaf5	3.08E-08	0.730	0.328	0.216	5.22E-04
Plet1os	3.14E-08	-0.581	0.272	0.41	5.33E-04
Zfp950	3.16E-08	0.981	0.177	0.092	5.37E-04
Ifi27	3.16E-08	-0.501	0.521	0.622	5.37E-04
Scd1	3.22E-08	1.064	0.13	0.059	5.46E-04
Cbfa2t2	3.23E-08	0.878	0.261	0.16	5.48E-04
Trip11	3.24E-08	0.431	0.512	0.371	5.49E-04
Akr1b8	3.33E-08	-0.704	0.408	0.506	5.66E-04
Churc1	3.39E-08	1.050	0.168	0.086	5.75E-04
Frs2	3.41E-08	0.623	0.32	0.202	5.79E-04
Vangl2	3.45E-08	1.365	0.097	0.038	5.85E-04
Ano6	3.56E-08	1.034	0.216	0.123	6.04E-04
Pde12	3.58E-08	-1.173	0.095	0.202	6.08E-04
Fut8	3.60E-08	0.799	0.294	0.186	6.10E-04
Cep350	3.66E-08	0.493	0.492	0.358	6.22E-04
Zfp36	3.70E-08	-0.301	0.631	0.746	6.28E-04
Ces2g	3.72E-08	-0.780	0.227	0.352	6.32E-04
Spidr	3.81E-08	0.838	0.253	0.152	6.46E-04
Kmt2d	3.85E-08	0.680	0.289	0.179	6.53E-04
Icam2	4.00E-08	0.423	0.728	0.605	6.79E-04

Xaf1	4.14E-08	0.570	0.382	0.255	7.02E-04
Dip2c	4.18E-08	0.592	0.579	0.474	7.09E-04
H3f3a	4.19E-08	0.258	0.95	0.942	7.11E-04
Sh3pxd2a	4.19E-08	0.861	0.317	0.208	7.11E-04
Ube2s	4.24E-08	-0.564	0.447	0.564	7.20E-04
Glp1r	4.29E-08	3.384	0.026	0.003	7.28E-04
Srsf5	4.31E-08	-0.361	0.637	0.727	7.31E-04
Ube2h	4.39E-08	0.311	0.739	0.638	7.44E-04
Mfap3	4.41E-08	0.735	0.285	0.178	7.48E-04
Atp11c	4.51E-08	0.547	0.551	0.427	7.65E-04
Efnb1	4.52E-08	0.919	0.428	0.325	7.67E-04
Gclm	4.66E-08	-0.779	0.207	0.336	7.90E-04
Milr1	4.68E-08	-1.215	0.063	0.161	7.94E-04
Tnik	4.75E-08	-0.514	0.374	0.509	8.06E-04
Cwf19l2	4.78E-08	0.647	0.374	0.254	8.12E-04
Sik2	4.82E-08	0.697	0.281	0.172	8.18E-04
Gm35533	4.90E-08	-0.284	0.259	0.397	8.31E-04
Gm10369	4.90E-08	1.387	0.086	0.032	8.32E-04
Mtch1	5.00E-08	-0.379	0.711	0.768	8.50E-04
Tcf7l2	5.02E-08	0.589	0.505	0.392	8.52E-04
H2-T23	5.10E-08	0.390	0.86	0.79	8.65E-04
Zyx	5.18E-08	0.744	0.309	0.2	8.79E-04
Lyplal1	5.21E-08	2.521	0.032	0.006	8.85E-04
Igsf3	5.25E-08	1.042	0.162	0.082	8.92E-04
Rhoj	5.27E-08	0.414	0.778	0.657	8.94E-04
Gm2000	5.28E-08	0.584	0.41	0.286	8.97E-04
Ppp1r12b	5.33E-08	0.783	0.248	0.148	9.04E-04
Amigo2	5.42E-08	1.045	0.192	0.105	9.20E-04
Upf3b	5.60E-08	0.676	0.302	0.193	9.50E-04
Nop58	5.63E-08	-0.784	0.227	0.349	9.56E-04
Lix1l	5.66E-08	0.717	0.281	0.175	9.61E-04
Cpne1	5.72E-08	0.504	0.421	0.289	9.71E-04
St3gal1	5.85E-08	0.580	0.473	0.354	9.94E-04
Rasgrf2	5.90E-08	-0.670	0.145	0.268	1.00E-03
Tspan12	6.01E-08	0.565	0.395	0.272	1.02E-03
Abhd18	6.13E-08	1.222	0.14	0.068	1.04E-03
Zfp277	6.36E-08	0.609	0.322	0.207	1.08E-03
S100a8	6.36E-08	-0.618	0.302	0.428	1.08E-03
Kif13a	6.40E-08	0.885	0.287	0.184	1.09E-03
Pcf11	6.50E-08	-0.678	0.328	0.445	1.10E-03
Fcho2	6.65E-08	0.390	0.844	0.814	1.13E-03
Nr1d1	6.73E-08	-0.658	0.311	0.434	1.14E-03
Adm	6.76E-08	0.986	0.363	0.259	1.15E-03
Mrpl48	6.83E-08	0.659	0.333	0.219	1.16E-03
Myc	6.84E-08	1.604	0.084	0.032	1.16E-03
Camk2d	7.03E-08	0.432	0.754	0.666	1.19E-03

Slmap	7.16E-08	0.430	0.618	0.498	1.21E-03
Slc24a5	7.24E-08	-0.779	0.21	0.331	1.23E-03
Uba52	7.27E-08	0.396	0.788	0.724	1.23E-03
Rbfox2	7.32E-08	0.482	0.605	0.484	1.24E-03
Flrt3	7.36E-08	3.714	0.022	0.002	1.25E-03
Afap1	7.47E-08	1.392	0.091	0.035	1.27E-03
Ern1	7.57E-08	-0.324	0.322	0.46	1.28E-03
Fbxo3	7.74E-08	0.662	0.317	0.205	1.31E-03
Mtmr2	7.75E-08	0.623	0.35	0.236	1.31E-03
Ly6a	7.92E-08	0.520	0.79	0.695	1.34E-03
Ift74	8.05E-08	1.306	0.102	0.042	1.37E-03
Fam181b	8.29E-08	5.329	0.011	0	1.41E-03
Tbc1d22a	8.35E-08	0.482	0.583	0.481	1.42E-03
Cstb	8.42E-08	-0.602	0.389	0.502	1.43E-03
Tet3	8.44E-08	0.518	0.521	0.396	1.43E-03
Svbp	8.45E-08	0.603	0.348	0.231	1.43E-03
Prkcb	8.45E-08	-0.688	0.246	0.374	1.43E-03
Oaf	8.54E-08	2.310	0.037	0.008	1.45E-03
Neb	8.62E-08	2.396	0.037	0.008	1.46E-03
4732471J01Rik	8.63E-08	0.899	0.201	0.113	1.47E-03
Cep112	8.68E-08	1.196	0.14	0.068	1.47E-03
Rbm7	9.03E-08	0.586	0.382	0.265	1.53E-03
Kctd12b	9.10E-08	0.617	0.419	0.295	1.54E-03
Elmod3	9.30E-08	-1.675	0.052	0.141	1.58E-03
Prelp	9.37E-08	-1.170	0.108	0.213	1.59E-03
Itprip	9.44E-08	0.733	0.281	0.176	1.60E-03
Lamc1	9.50E-08	0.792	0.255	0.155	1.61E-03
Spag9	9.52E-08	-0.310	0.901	0.944	1.62E-03
Nrxn2	9.71E-08	-2.364	0.015	0.086	1.65E-03
Kmt2a	9.93E-08	0.395	0.575	0.443	1.69E-03
Snrnp70	9.99E-08	0.442	0.577	0.454	1.70E-03
Tfcp2	1.00E-07	1.166	0.16	0.083	1.70E-03
Tap2	1.01E-07	0.602	0.4	0.284	1.71E-03
Adam15	1.01E-07	0.711	0.337	0.23	1.72E-03
Mannr	1.05E-07	3.369	0.015	0.001	1.79E-03
Thoc7	1.06E-07	0.572	0.374	0.255	1.80E-03
Hprt	1.07E-07	-0.573	0.436	0.55	1.81E-03
Ube3c	1.10E-07	0.800	0.315	0.211	1.86E-03
Eef1g	1.10E-07	0.324	0.847	0.753	1.87E-03
Fbxo45	1.15E-07	1.033	0.132	0.062	1.94E-03
Asxl2	1.15E-07	0.541	0.447	0.324	1.95E-03
Nap1l1	1.16E-07	0.413	0.592	0.46	1.97E-03
Fam76b	1.16E-07	0.673	0.268	0.164	1.97E-03
Flna	1.17E-07	0.936	0.22	0.128	1.99E-03
Fndc3b	1.18E-07	0.465	0.706	0.632	2.00E-03
P3h4	1.19E-07	2.936	0.024	0.003	2.01E-03

Socs1	1.20E-07	-0.675	0.173	0.296	2.03E-03
Tmem30a	1.20E-07	-0.345	0.724	0.803	2.04E-03
Tns1	1.23E-07	0.352	0.84	0.757	2.10E-03
Rplp2	1.24E-07	0.192	0.994	0.992	2.10E-03
Neo1	1.24E-07	1.282	0.093	0.037	2.10E-03
Epb41l2	1.25E-07	-0.253	0.866	0.918	2.12E-03
Spock2	1.28E-07	2.439	0.032	0.006	2.18E-03
Tpbg	1.29E-07	4.329	0.013	0	2.19E-03
Rtraf	1.31E-07	0.458	0.585	0.474	2.22E-03
Id3	1.32E-07	-0.275	0.931	0.964	2.25E-03
Setd2	1.40E-07	0.533	0.469	0.35	2.38E-03
Cd244a	1.40E-07	2.862	0.03	0.005	2.38E-03
Plin2	1.44E-07	0.482	0.672	0.582	2.45E-03
Olfm1	1.45E-07	-0.781	0.274	0.391	2.45E-03
2610307P16Rik	1.45E-07	3.691	0.028	0.005	2.47E-03
Lgals8	1.49E-07	0.604	0.384	0.267	2.53E-03
Fam43a	1.51E-07	0.573	0.514	0.406	2.56E-03
F830016B08Rik	1.53E-07	0.720	0.197	0.109	2.60E-03
Gm12216	1.54E-07	-0.799	0.257	0.381	2.62E-03
Mark3	1.54E-07	0.486	0.6	0.485	2.62E-03
Xbp1	1.55E-07	-0.604	0.406	0.513	2.63E-03
Meis3	1.56E-07	2.521	0.028	0.005	2.64E-03
Mtss1	1.56E-07	-0.260	0.745	0.845	2.65E-03
Kcnb1	1.58E-07	-0.461	0.469	0.587	2.68E-03
Slc16a2	1.59E-07	0.593	0.462	0.351	2.70E-03
BC028528	1.61E-07	0.557	0.382	0.266	2.73E-03
Gm15918	1.63E-07	1.856	0.054	0.016	2.76E-03
Ptpn4	1.64E-07	0.942	0.153	0.078	2.78E-03
Snu13	1.64E-07	-0.554	0.484	0.573	2.78E-03
Tmed10	1.68E-07	-0.360	0.758	0.814	2.84E-03
Ctnna3	1.69E-07	-2.205	0.026	0.101	2.86E-03
Cgnl1	1.70E-07	0.959	0.246	0.154	2.88E-03
Gpr182	1.71E-07	-0.192	0.972	0.993	2.90E-03
Cbx4	1.74E-07	-0.949	0.231	0.341	2.96E-03
Akap9	1.75E-07	0.572	0.423	0.308	2.97E-03
Macrod2	1.78E-07	1.066	0.251	0.157	3.02E-03
Plekhh3	1.78E-07	1.176	0.121	0.056	3.02E-03
Eif5a	1.78E-07	-0.430	0.495	0.607	3.03E-03
Vps13d	1.79E-07	0.707	0.337	0.229	3.03E-03
Hace1	1.82E-07	0.842	0.251	0.154	3.08E-03
Arhgap28	1.82E-07	3.428	0.019	0.002	3.09E-03
Reep5	1.86E-07	-0.550	0.415	0.524	3.15E-03
Hmgcl	1.87E-07	0.621	0.339	0.226	3.17E-03
Dleu2	1.88E-07	-0.510	0.624	0.723	3.20E-03
Fam193a	1.94E-07	0.442	0.49	0.359	3.29E-03
Ccnl2	2.00E-07	0.535	0.451	0.335	3.39E-03

Nkd1	2.02E-07	-0.845	0.151	0.263	3.43E-03
Cnp	2.03E-07	-0.879	0.151	0.262	3.44E-03
Cela1	2.15E-07	-2.179	0.022	0.093	3.65E-03
Xdh	2.15E-07	0.364	0.842	0.775	3.65E-03
Rasgrp3	2.17E-07	-0.267	0.827	0.894	3.68E-03
Art4	2.19E-07	1.521	0.082	0.032	3.73E-03
Dcakd	2.28E-07	1.295	0.11	0.049	3.87E-03
Map1lc3b	2.28E-07	-0.327	0.803	0.844	3.87E-03
Trps1	2.31E-07	0.583	0.495	0.379	3.92E-03
Usp34	2.33E-07	0.372	0.724	0.625	3.96E-03
Gm11944	2.35E-07	1.219	0.108	0.048	3.99E-03
Antxr2	2.36E-07	1.123	0.119	0.055	4.00E-03
Serpina3b	2.37E-07	2.287	0.035	0.007	4.03E-03
Tfpi	2.37E-07	0.360	0.754	0.701	4.03E-03
Homer1	2.38E-07	-1.270	0.102	0.2	4.05E-03
Acvr2a	2.40E-07	0.853	0.283	0.185	4.07E-03
H13	2.41E-07	-0.580	0.363	0.478	4.09E-03
Cnot6l	2.46E-07	0.421	0.542	0.425	4.17E-03
Micu2	2.54E-07	0.896	0.266	0.171	4.31E-03
Pdia5	2.57E-07	0.936	0.212	0.124	4.37E-03
Adcy6	2.60E-07	0.775	0.238	0.144	4.42E-03
Dusp2	2.61E-07	1.114	0.149	0.076	4.42E-03
Phldb2	2.62E-07	0.390	0.758	0.662	4.44E-03
2210016F16Rik	2.65E-07	-1.272	0.073	0.166	4.50E-03
Cfl1	2.67E-07	-0.250	0.892	0.926	4.54E-03
Timp2	2.69E-07	-1.420	0.076	0.168	4.57E-03
Arl6	2.71E-07	1.762	0.054	0.016	4.60E-03
Rcn1	2.76E-07	-0.458	0.538	0.623	4.68E-03
Taf12	2.81E-07	0.707	0.229	0.135	4.76E-03
0610043K17Rik	2.82E-07	-1.110	0.13	0.234	4.78E-03
Plxnd1	2.86E-07	-0.313	0.842	0.87	4.85E-03
Bicra	2.88E-07	0.657	0.311	0.204	4.88E-03
Chfr	2.92E-07	0.945	0.199	0.115	4.95E-03
Cmklr1	2.94E-07	2.131	0.045	0.012	5.00E-03
Senp6	2.97E-07	0.433	0.544	0.419	5.04E-03
Itsn2	2.98E-07	0.435	0.592	0.483	5.05E-03
Pkp4	3.02E-07	0.792	0.337	0.237	5.13E-03
Nipbl	3.05E-07	0.274	0.814	0.687	5.18E-03
Alox12	3.08E-07	-1.508	0.045	0.128	5.23E-03
Apoc1	3.10E-07	0.495	0.562	0.441	5.27E-03
Khdrbs3	3.14E-07	0.977	0.294	0.198	5.32E-03
Akap11	3.28E-07	0.614	0.313	0.207	5.57E-03
Thumpd2	3.36E-07	1.150	0.114	0.053	5.70E-03
Emcn	3.42E-07	0.357	0.754	0.666	5.81E-03
Srek1ip1	3.46E-07	0.773	0.248	0.153	5.87E-03
Ifit2	3.59E-07	0.899	0.283	0.185	6.10E-03

Mbtps1	3.65E-07	0.737	0.296	0.194	6.20E-03
Il10rb	3.65E-07	0.517	0.484	0.362	6.20E-03
Fto	3.66E-07	0.616	0.441	0.333	6.21E-03
Plxdc1	3.80E-07	-1.220	0.073	0.164	6.45E-03
Spopl	3.87E-07	0.578	0.302	0.196	6.57E-03
Sfmbt1	3.90E-07	0.742	0.251	0.156	6.61E-03
Cflar	3.95E-07	-0.430	0.644	0.709	6.71E-03
Pias2	3.96E-07	0.642	0.341	0.235	6.72E-03
Epor	3.97E-07	1.173	0.121	0.058	6.74E-03
Plod1	3.99E-07	0.437	0.592	0.479	6.78E-03
Vat1	4.01E-07	-0.624	0.307	0.421	6.81E-03
Ptbp2	4.03E-07	0.601	0.369	0.26	6.83E-03
Herc3	4.04E-07	0.777	0.263	0.166	6.85E-03
Ampd3	4.09E-07	-1.002	0.104	0.203	6.95E-03
Chchd3	4.13E-07	0.547	0.469	0.359	7.00E-03
C1qtnf9	4.13E-07	1.521	0.063	0.021	7.01E-03
Klhdc8a	4.19E-07	-3.880	0.002	0.057	7.11E-03
Hs3st1	4.22E-07	2.769	0.026	0.004	7.16E-03
Zfp148	4.32E-07	0.432	0.594	0.494	7.33E-03
Cep170	4.33E-07	0.551	0.384	0.274	7.34E-03
Lipe	4.35E-07	0.864	0.184	0.103	7.39E-03
Abhd17b	4.37E-07	0.755	0.417	0.307	7.41E-03
Rc3h2	4.45E-07	0.599	0.408	0.297	7.55E-03
Stxbp4	4.73E-07	1.341	0.082	0.032	8.03E-03
Nsmaf	4.77E-07	0.621	0.259	0.16	8.09E-03
a	4.89E-07	-1.318	0.054	0.138	8.31E-03
9930021J03Rik	5.00E-07	0.557	0.421	0.309	8.49E-03
Heph	5.02E-07	0.912	0.179	0.1	8.53E-03
Tapt1	5.07E-07	0.836	0.173	0.094	8.61E-03
Sec22a	5.16E-07	0.998	0.156	0.082	8.75E-03
Ubxn7	5.26E-07	0.794	0.22	0.132	8.92E-03
Zcchc14	5.30E-07	0.432	0.581	0.468	8.99E-03
Pds5a	5.31E-07	0.489	0.557	0.455	9.01E-03
Cmas	5.32E-07	0.696	0.339	0.236	9.04E-03
Anapc15	5.40E-07	-0.547	0.425	0.538	9.16E-03
Fbxo22	5.41E-07	0.768	0.281	0.183	9.19E-03
Hk1	5.43E-07	0.623	0.341	0.236	9.22E-03
Fkbp1a	5.43E-07	-0.216	0.959	0.975	9.22E-03
Upp1	5.50E-07	0.627	0.367	0.26	9.33E-03
Thoc2	5.52E-07	0.424	0.629	0.523	9.36E-03
Apobec3	5.53E-07	0.660	0.27	0.172	9.38E-03
Aff4	5.54E-07	-0.514	0.553	0.631	9.40E-03
Mgst1	5.63E-07	0.457	0.413	0.293	9.56E-03
Cfap97	5.68E-07	0.825	0.212	0.126	9.64E-03
Dock1	5.75E-07	0.360	0.801	0.715	9.76E-03
Mdm4	5.77E-07	0.541	0.371	0.26	9.79E-03

Itpr3	5.77E-07	1.091	0.13	0.064	9.80E-03
Sar1a	5.83E-07	-0.549	0.309	0.432	9.89E-03
Clec1a	5.92E-07	0.456	0.549	0.438	1.01E-02
Kctd17	5.96E-07	0.729	0.248	0.155	1.01E-02
Slc7a5	5.99E-07	-0.699	0.229	0.34	1.02E-02
Srpr	6.04E-07	-0.442	0.443	0.559	1.02E-02
Acaa2	6.05E-07	0.529	0.395	0.284	1.03E-02
Cdc42ep3	6.15E-07	0.759	0.272	0.175	1.04E-02
Gatad2b	6.31E-07	0.533	0.495	0.386	1.07E-02
Prkcg	6.31E-07	-1.003	0.056	0.141	1.07E-02
Pik3c2b	6.33E-07	0.570	0.423	0.313	1.07E-02
Cdc40	6.43E-07	0.631	0.35	0.244	1.09E-02
Apcdd1	6.67E-07	2.184	0.032	0.007	1.13E-02
Abhd8	6.68E-07	1.132	0.121	0.058	1.13E-02
Serpina1e	6.75E-07	-2.977	0.009	0.067	1.15E-02
Kdm5b	6.75E-07	0.560	0.315	0.21	1.15E-02
Eif3k	6.84E-07	0.393	0.7	0.593	1.16E-02
Uri1	6.87E-07	0.579	0.365	0.255	1.17E-02
Rsf1	6.96E-07	0.449	0.492	0.372	1.18E-02
Akr1b3	6.99E-07	-0.598	0.322	0.431	1.19E-02
Arrb2	7.04E-07	-0.594	0.307	0.418	1.20E-02
Pdlim1	7.07E-07	-0.505	0.533	0.624	1.20E-02
Klhdc10	7.10E-07	0.686	0.281	0.182	1.20E-02
Cul1	7.11E-07	0.409	0.54	0.412	1.21E-02
Gabarap	7.21E-07	-0.256	0.89	0.903	1.22E-02
Zbtb11	7.27E-07	-0.729	0.289	0.406	1.23E-02
Obsl1	7.38E-07	2.047	0.037	0.009	1.25E-02
Atp6v1a	7.40E-07	-0.519	0.367	0.485	1.26E-02
Ftl1-ps1	7.42E-07	-0.887	0.13	0.231	1.26E-02
Asah2	7.42E-07	0.520	0.475	0.362	1.26E-02
Fip1l1	7.45E-07	0.512	0.376	0.263	1.26E-02
Vegfb	7.64E-07	-0.853	0.177	0.282	1.30E-02
Ergic3	7.66E-07	0.510	0.317	0.208	1.30E-02
Vta1	7.68E-07	0.640	0.326	0.224	1.30E-02
Btrc	7.70E-07	0.710	0.305	0.206	1.31E-02
Inpp5k	7.78E-07	0.521	0.369	0.255	1.32E-02
Hmgn1	7.87E-07	0.566	0.4	0.29	1.34E-02
Bcr	7.88E-07	0.682	0.27	0.175	1.34E-02
Fryl	7.99E-07	0.636	0.495	0.4	1.36E-02
Nmt2	8.01E-07	0.860	0.235	0.147	1.36E-02
Ndst1	8.04E-07	0.436	0.527	0.408	1.37E-02
Gm45345	8.16E-07	3.106	0.019	0.002	1.38E-02
Tnfaip8l1	8.18E-07	0.812	0.257	0.166	1.39E-02
Pcdhb4	8.19E-07	2.439	0.03	0.006	1.39E-02
Nup98	8.23E-07	-0.598	0.404	0.506	1.40E-02
Pot1b	8.32E-07	1.032	0.162	0.089	1.41E-02

Orm1	8.34E-07	1.394	0.071	0.027	1.42E-02
Clstn1	8.39E-07	2.287	0.03	0.006	1.42E-02
Dgkd	8.48E-07	0.831	0.233	0.146	1.44E-02
Mrpl30	8.54E-07	0.545	0.397	0.287	1.45E-02
Cdkal1	8.60E-07	0.534	0.395	0.283	1.46E-02
Eva1a	8.61E-07	2.043	0.045	0.013	1.46E-02
Otud4	8.79E-07	0.530	0.341	0.232	1.49E-02
Arfgap1	8.86E-07	0.716	0.212	0.125	1.50E-02
Calcoco1	8.86E-07	0.931	0.164	0.09	1.50E-02
Xpr1	8.93E-07	0.665	0.339	0.236	1.52E-02
Tmem221	8.95E-07	3.369	0.015	0.001	1.52E-02
Plpp1	9.08E-07	-0.188	0.965	0.986	1.54E-02
Tap1	9.10E-07	0.732	0.272	0.178	1.54E-02
Rab3gap2	9.21E-07	0.633	0.391	0.289	1.56E-02
Atp9a	9.23E-07	1.628	0.067	0.024	1.57E-02
Zmym3	9.28E-07	1.334	0.076	0.029	1.58E-02
Myl12a	9.29E-07	0.371	0.771	0.684	1.58E-02
Cox4i1	9.32E-07	-0.203	0.968	0.978	1.58E-02
Actn1	9.34E-07	0.799	0.231	0.143	1.59E-02
Camp	9.36E-07	-1.185	0.065	0.15	1.59E-02
Peak1	9.50E-07	0.330	0.903	0.887	1.61E-02
Gm12185	9.53E-07	1.091	0.138	0.071	1.62E-02
Sgk1	9.58E-07	-0.341	0.788	0.84	1.63E-02
Med13l	9.78E-07	0.440	0.531	0.422	1.66E-02
Dpm3	9.78E-07	-0.489	0.421	0.527	1.66E-02
Rpl9-ps6	9.88E-07	0.782	0.222	0.136	1.68E-02
Ppp2r5c	9.91E-07	0.378	0.579	0.461	1.68E-02
Gbp9	9.94E-07	0.829	0.222	0.136	1.69E-02
Usp3	9.95E-07	0.370	0.607	0.483	1.69E-02
Arhgap23	1.03E-06	-0.309	0.773	0.811	1.75E-02
Hspg2	1.04E-06	0.259	0.862	0.76	1.76E-02
Cnst	1.08E-06	0.750	0.222	0.136	1.83E-02
Inpp5d	1.08E-06	0.544	0.402	0.289	1.84E-02
Grap	1.09E-06	-0.594	0.305	0.415	1.84E-02
Cog4	1.09E-06	0.748	0.231	0.143	1.85E-02
Rflnb	1.10E-06	1.762	0.063	0.022	1.87E-02
Syt7	1.10E-06	1.106	0.125	0.062	1.87E-02
Gngt2	1.10E-06	-0.355	0.693	0.773	1.87E-02
Blmh	1.11E-06	0.521	0.384	0.276	1.88E-02
Dcst1	1.11E-06	2.659	0.022	0.003	1.88E-02
Lysmd2	1.11E-06	2.659	0.022	0.003	1.88E-02
Kcnk5	1.11E-06	2.884	0.026	0.005	1.88E-02
Gja5	1.12E-06	2.914	0.026	0.005	1.90E-02
Hs6st1	1.12E-06	0.747	0.255	0.164	1.91E-02
Kif1c	1.13E-06	0.597	0.289	0.191	1.92E-02
Cep295	1.13E-06	0.936	0.164	0.09	1.92E-02

Adk	1.14E-06	0.859	0.194	0.114	1.93E-02
Pold3	1.20E-06	0.923	0.192	0.113	2.04E-02
Slc6a8	1.21E-06	1.397	0.076	0.03	2.06E-02
Gtf3c1	1.21E-06	0.636	0.255	0.161	2.06E-02
Galk2	1.22E-06	0.715	0.317	0.221	2.07E-02
Prrg1	1.22E-06	0.647	0.339	0.238	2.08E-02
Ahr	1.23E-06	0.503	0.454	0.348	2.09E-02
Stim1	1.24E-06	0.605	0.395	0.287	2.10E-02
Slc38a6	1.24E-06	0.482	0.521	0.415	2.10E-02
Erlec1	1.26E-06	0.582	0.356	0.254	2.14E-02
Bax	1.27E-06	-0.487	0.523	0.609	2.16E-02
Hdgfl3	1.28E-06	0.576	0.363	0.259	2.17E-02
Snx10	1.30E-06	0.979	0.127	0.064	2.21E-02
Serpina3g	1.31E-06	0.514	0.594	0.498	2.22E-02
Rara	1.40E-06	0.836	0.231	0.145	2.37E-02
Ankrd10	1.40E-06	0.633	0.266	0.171	2.38E-02
Sfswap	1.41E-06	0.547	0.387	0.281	2.40E-02
Dennd1b	1.45E-06	0.644	0.322	0.224	2.46E-02
Ppme1	1.46E-06	0.643	0.309	0.209	2.48E-02
Nfix	1.46E-06	0.458	0.382	0.269	2.48E-02
Cngb1	1.47E-06	1.835	0.045	0.013	2.49E-02
Fam216a	1.47E-06	1.647	0.056	0.019	2.50E-02
Fam193b	1.49E-06	0.785	0.214	0.13	2.52E-02
Atg2b	1.49E-06	0.889	0.168	0.094	2.53E-02
Csnk1g1	1.51E-06	0.611	0.35	0.244	2.56E-02
Sdc4	1.52E-06	0.613	0.285	0.188	2.58E-02
Ndrg2	1.53E-06	-1.007	0.104	0.196	2.60E-02
Sh2d3c	1.61E-06	0.450	0.486	0.372	2.73E-02
Lrig1	1.62E-06	2.199	0.039	0.01	2.74E-02
Sgce	1.62E-06	0.981	0.151	0.082	2.75E-02
Rnf216	1.64E-06	0.429	0.492	0.37	2.78E-02
Srpk2	1.64E-06	0.505	0.475	0.369	2.78E-02
Cox6b2	1.64E-06	3.936	0.013	0.001	2.79E-02
Map2k5	1.66E-06	0.526	0.421	0.309	2.82E-02
Hnrnph1	1.70E-06	-0.386	0.618	0.69	2.89E-02
Chp2	1.72E-06	1.936	0.039	0.01	2.92E-02
Erc1	1.76E-06	0.602	0.397	0.296	2.99E-02
Gm34455	1.76E-06	-1.005	0.188	0.287	3.00E-02
AU021092	1.78E-06	-0.403	0.197	0.313	3.01E-02
Unc45bos	1.78E-06	2.361	0.03	0.006	3.03E-02
Fuca1	1.80E-06	0.489	0.4	0.289	3.05E-02
Ncor1	1.82E-06	0.315	0.81	0.706	3.09E-02
Stab2	1.86E-06	0.320	0.924	0.988	3.16E-02
Desi2	1.90E-06	0.434	0.469	0.354	3.23E-02
Clec4b1	1.94E-06	1.631	0.058	0.02	3.29E-02
Naa16	1.96E-06	0.700	0.233	0.146	3.33E-02

Slc45a3	1.97E-06	1.981	0.041	0.011	3.34E-02
Sema6b	1.99E-06	0.582	0.38	0.275	3.37E-02
Phax	1.99E-06	0.625	0.298	0.202	3.37E-02
Vwa8	1.99E-06	0.659	0.266	0.173	3.38E-02
Csrnp1	2.20E-06	-0.799	0.132	0.229	3.73E-02
Col4a3bp	2.23E-06	0.478	0.555	0.462	3.79E-02
Fam160b1	2.28E-06	0.530	0.305	0.204	3.88E-02
Atp5h	2.29E-06	-0.218	0.909	0.942	3.89E-02
Lox	2.31E-06	4.106	0.011	0	3.93E-02
Mlf1	2.35E-06	-1.925	0.028	0.094	3.99E-02
Cep83	2.37E-06	-0.655	0.24	0.35	4.03E-02
P3h3	2.38E-06	1.071	0.11	0.053	4.04E-02
Btbd1	2.39E-06	0.620	0.35	0.253	4.05E-02
Zfp30	2.39E-06	1.232	0.076	0.03	4.06E-02
Pnn	2.41E-06	0.509	0.402	0.292	4.10E-02
Mrps7	2.45E-06	0.642	0.253	0.162	4.16E-02
Acvr1	2.47E-06	0.540	0.417	0.316	4.20E-02
C230035I16Rik	2.50E-06	1.661	0.05	0.016	4.25E-02
Ncoa2	2.52E-06	0.449	0.512	0.411	4.29E-02
Cmtm4	2.53E-06	0.791	0.229	0.144	4.29E-02
Armcx2	2.55E-06	1.026	0.162	0.092	4.32E-02
Dis3l2	2.56E-06	0.565	0.315	0.217	4.35E-02
Sumo3	2.60E-06	-0.557	0.4	0.498	4.41E-02
Meis2	2.63E-06	0.242	0.957	0.964	4.46E-02
Apobec1	2.67E-06	0.929	0.201	0.123	4.53E-02
Junos	2.68E-06	-1.105	0.065	0.144	4.55E-02
Rbp4	2.73E-06	0.797	0.184	0.108	4.64E-02
Herc2	2.76E-06	0.546	0.523	0.423	4.68E-02
Gm50333	2.77E-06	-2.517	0.011	0.066	4.69E-02
Klhl29	2.77E-06	2.599	0.026	0.005	4.71E-02
Cplx2	2.78E-06	1.073	0.13	0.067	4.71E-02
Pear1	2.80E-06	-0.502	0.395	0.494	4.75E-02
Prpf40b	2.82E-06	1.064	0.132	0.069	4.79E-02
Ajuba	2.86E-06	1.821	0.032	0.008	4.86E-02
Kdm5c	2.87E-06	0.464	0.341	0.237	4.87E-02
Nup205	2.88E-06	0.873	0.143	0.076	4.89E-02
Gnpat	2.88E-06	0.914	0.158	0.088	4.89E-02
Mllt6	2.91E-06	0.636	0.281	0.188	4.94E-02
Qars	2.92E-06	0.814	0.171	0.097	4.95E-02
Adam12	2.93E-06	-0.943	0.082	0.167	4.98E-02
Ube2r2	2.93E-06	0.416	0.598	0.494	4.98E-02
Slc25a23	2.94E-06	1.170	0.106	0.051	4.99E-02

Supplementary Table 8. Pairwise comparison of GAN-Adj.NT verses GAN endothelial cells. Significant DEGs (adjusted p-value < 0.05) shown.

Gene	p_val	avg_log2FC	pct.1	pct.2	p_val_adj
Gm17749	8.13E-08	2.028	0.112	0.026	1.38E-03
Ifit1	1.70E-08	1.734	0.253	0.125	2.89E-04
Nabp1	6.96E-09	1.489	0.212	0.086	1.18E-04
Oasl1	8.31E-07	1.295	0.196	0.091	1.41E-02
Slc5a3	9.31E-08	1.268	0.216	0.099	1.58E-03
Camp	1.48E-06	1.206	0.159	0.065	2.51E-02
9930111J21Rik2	4.62E-08	1.182	0.39	0.257	7.84E-04
Ngp	7.13E-07	1.158	0.198	0.091	1.21E-02
Csrnp1	4.61E-08	1.141	0.261	0.132	7.82E-04
Hsph1	1.52E-08	1.127	0.425	0.285	2.59E-04
Hbegf	1.06E-07	1.099	0.352	0.222	1.79E-03
Sertad2	1.61E-08	1.096	0.431	0.289	2.73E-04
Gda	8.93E-07	1.054	0.231	0.119	1.52E-02
Ezh2	1.85E-06	1.040	0.276	0.16	3.15E-02
Sertad1	7.95E-09	1.038	0.384	0.238	1.35E-04
Daxx	1.08E-06	1.029	0.28	0.162	1.83E-02
Cdkn1a	2.73E-07	1.025	0.453	0.324	4.64E-03
Hspa1b	9.91E-09	1.003	0.556	0.413	1.68E-04
Rsad2	2.55E-08	0.987	0.398	0.251	4.32E-04
Ifi211	2.61E-07	0.980	0.352	0.222	4.43E-03
Polg2	6.46E-09	0.906	0.351	0.192	1.10E-04
Dusp5	2.98E-07	0.873	0.292	0.164	5.06E-03
Mup20	2.58E-06	0.834	0.445	0.315	4.38E-02
Ccl2	3.55E-07	0.808	0.155	0.056	6.02E-03
Fosb	1.07E-07	0.776	0.616	0.501	1.81E-03
Pde4b	1.45E-07	0.771	0.547	0.41	2.47E-03
Itga8	4.05E-07	0.724	0.352	0.214	6.88E-03
Azin1	2.33E-07	0.697	0.415	0.276	3.96E-03
Btg1	2.64E-08	0.678	0.637	0.525	4.48E-04
Ern1	2.79E-08	0.676	0.466	0.322	4.73E-04
Cxcl9	2.80E-08	0.666	0.769	0.702	4.75E-04
Rnd3	3.03E-07	0.642	0.413	0.261	5.15E-03
Iigp1	1.84E-06	0.639	0.825	0.771	3.13E-02
Pnp	5.67E-08	0.595	0.759	0.693	9.62E-04
Rhob	2.24E-08	0.583	0.782	0.674	3.80E-04
Jun	1.90E-08	0.536	0.925	0.89	3.22E-04
Ccnl1	4.35E-08	0.533	0.618	0.473	7.38E-04
Gja1	6.97E-09	0.529	0.708	0.564	1.18E-04
Manf	1.25E-06	0.489	0.579	0.46	2.11E-02
Dusp1	1.01E-07	0.477	0.858	0.732	1.71E-03
Gm12840	4.13E-07	0.475	0.825	0.756	7.01E-03

Nfkbia	6.10E-08	0.417	0.888	0.834	1.03E-03
Bmp2	4.56E-08	0.321	0.921	0.844	7.75E-04
Jund	2.13E-06	0.310	0.862	0.773	3.62E-02
Calm1	5.84E-07	0.274	0.991	0.983	9.92E-03
Ptma	9.59E-08	0.254	1	0.996	1.63E-03
Irf1	2.35E-06	0.241	0.743	0.596	3.99E-02
Actb	1.39E-06	0.231	0.997	0.998	2.35E-02
Rps12	1.39E-06	-0.158	0.999	0.998	2.36E-02
Rpl13a	1.04E-06	-0.182	0.996	1	1.76E-02
Rps20	1.71E-07	-0.205	0.999	0.996	2.90E-03
Igfbp7	3.63E-07	-0.215	0.997	0.996	6.16E-03
Sptbn1	2.65E-07	-0.222	0.993	0.998	4.49E-03
Rps16	1.78E-07	-0.224	0.996	1	3.03E-03
Rps27a	2.32E-07	-0.229	1	0.996	3.93E-03
Rpl27	6.67E-07	-0.230	0.981	0.987	1.13E-02
Rps8	9.73E-09	-0.231	0.999	0.998	1.65E-04
Rps13	7.03E-07	-0.234	0.989	0.991	1.19E-02
Rps14	7.59E-09	-0.236	0.999	0.996	1.29E-04
Sparc	2.71E-06	-0.236	0.969	0.985	4.61E-02
Rpl11	2.47E-07	-0.238	0.988	0.991	4.20E-03
Rps23	6.75E-09	-0.240	0.997	0.998	1.15E-04
Rps24	1.10E-08	-0.247	0.997	0.998	1.86E-04
Rpl18	8.26E-07	-0.249	0.977	0.991	1.40E-02
Rps9	1.25E-08	-0.250	0.996	0.994	2.13E-04
Rpl15	1.96E-06	-0.254	0.976	0.987	3.33E-02
Cd47	1.33E-06	-0.256	0.968	0.976	2.26E-02
Rpl30	3.30E-08	-0.256	0.999	0.991	5.60E-04
Rps26	8.92E-07	-0.263	0.972	0.983	1.51E-02
Rpl35	1.36E-07	-0.263	0.977	0.985	2.30E-03
Rpsa	2.13E-06	-0.264	0.981	0.991	3.62E-02
Rbm39	3.18E-07	-0.267	0.958	0.972	5.40E-03
Pfdn5	8.68E-08	-0.278	0.921	0.959	1.47E-03
Rps18	6.46E-08	-0.279	0.984	0.985	1.10E-03
Rpl4	2.89E-06	-0.281	0.849	0.916	4.90E-02
Rps3	2.10E-08	-0.282	0.987	0.996	3.57E-04
Rps19	7.95E-09	-0.285	0.988	0.991	1.35E-04
Rps5	4.09E-08	-0.290	0.991	0.989	6.94E-04
Atrx	4.74E-07	-0.290	0.872	0.916	8.05E-03
Rpl3	1.07E-08	-0.297	0.974	0.983	1.82E-04
Rpl9	8.57E-09	-0.300	0.996	0.996	1.46E-04
Psap	1.36E-08	-0.312	0.938	0.968	2.30E-04
Itgb1	1.17E-07	-0.324	0.91	0.95	1.99E-03
Itga9	2.25E-06	-0.324	0.839	0.875	3.82E-02
Rps7	3.40E-08	-0.325	0.981	0.991	5.78E-04
Cd36	2.97E-08	-0.328	0.935	0.952	5.05E-04
Elk3	2.45E-07	-0.332	0.837	0.896	4.16E-03

Mef2c	4.08E-08	-0.338	0.777	0.879	6.93E-04
Ndfip1	7.90E-07	-0.341	0.758	0.836	1.34E-02
Esam	2.82E-08	-0.342	0.868	0.937	4.79E-04
Flnb	1.61E-06	-0.352	0.722	0.825	2.74E-02
F2r	1.54E-06	-0.354	0.702	0.81	2.62E-02
Peak1	9.68E-07	-0.361	0.887	0.903	1.64E-02
Sh3glb1	8.16E-09	-0.364	0.853	0.905	1.39E-04
Ccdc85b	1.34E-07	-0.370	0.679	0.821	2.28E-03
Nid1	1.50E-07	-0.383	0.595	0.726	2.55E-03
Rapgef1	9.91E-07	-0.384	0.598	0.726	1.68E-02
Eif3h	2.39E-06	-0.384	0.645	0.737	4.05E-02
Anp32a	5.21E-07	-0.390	0.626	0.749	8.84E-03
Ncor1	8.82E-07	-0.397	0.703	0.81	1.50E-02
Car8	2.64E-06	-0.397	0.516	0.657	4.48E-02
Ly6a	1.66E-06	-0.400	0.691	0.79	2.82E-02
Stab2	3.21E-08	-0.402	0.921	0.924	5.46E-04
Uba52	8.68E-07	-0.404	0.69	0.788	1.47E-02
Btnl9	1.25E-07	-0.407	0.703	0.795	2.13E-03
Adam23	1.32E-07	-0.407	0.901	0.924	2.24E-03
Cd59a	1.83E-08	-0.411	0.778	0.866	3.10E-04
St3gal4	1.71E-07	-0.417	0.767	0.851	2.91E-03
Zmiz1	1.29E-07	-0.420	0.734	0.847	2.20E-03
Rnf130	1.07E-06	-0.423	0.618	0.717	1.81E-02
Pura	1.42E-07	-0.434	0.544	0.689	2.42E-03
Cpd	5.25E-07	-0.436	0.563	0.672	8.91E-03
Itpr2	1.34E-06	-0.440	0.531	0.657	2.27E-02
Scaf11	7.18E-07	-0.449	0.536	0.657	1.22E-02
Notch1	1.96E-07	-0.453	0.618	0.724	3.33E-03
Raph1	1.97E-07	-0.454	0.659	0.765	3.34E-03
Zeb1	8.54E-08	-0.459	0.879	0.92	1.45E-03
Rora	1.38E-06	-0.461	0.77	0.827	2.34E-02
Ushbp1	1.17E-07	-0.468	0.608	0.726	1.98E-03
Fkbp5	7.16E-08	-0.471	0.602	0.724	1.21E-03
Oip5os1	1.81E-08	-0.474	0.664	0.786	3.08E-04
Stim2	6.40E-08	-0.477	0.587	0.706	1.09E-03
Dennd4c	5.10E-07	-0.506	0.477	0.624	8.65E-03
Afdn	2.06E-06	-0.516	0.478	0.583	3.50E-02
Cd2ap	6.22E-08	-0.530	0.612	0.724	1.06E-03
H2-Q7	5.42E-08	-0.533	0.612	0.702	9.21E-04
Ndufa6	3.69E-08	-0.534	0.605	0.706	6.26E-04
Tcf7l1	6.56E-09	-0.535	0.688	0.778	1.11E-04
Gpi1	3.06E-07	-0.537	0.47	0.611	5.19E-03
Map4k5	2.71E-06	-0.538	0.64	0.717	4.60E-02
Grb2	1.60E-06	-0.538	0.409	0.533	2.71E-02
Pkn1	2.56E-06	-0.545	0.359	0.488	4.35E-02
Jcad	8.92E-07	-0.554	0.382	0.514	1.51E-02

Eif4b	5.59E-07	-0.557	0.466	0.587	9.48E-03
Slc25a4	1.20E-06	-0.558	0.499	0.611	2.04E-02
Ryk	1.58E-06	-0.565	0.363	0.497	2.69E-02
Evi5	2.10E-06	-0.573	0.407	0.527	3.56E-02
Nova2	2.75E-07	-0.576	0.327	0.471	4.67E-03
Itch	9.77E-07	-0.587	0.422	0.542	1.66E-02
Galnt18	2.48E-08	-0.592	0.591	0.73	4.21E-04
Pip4k2a	1.50E-06	-0.596	0.515	0.605	2.55E-02
Eif4ebp1	3.44E-07	-0.599	0.347	0.484	5.84E-03
Lyve1	2.16E-08	-0.607	0.602	0.728	3.67E-04
Wdr11	1.33E-06	-0.624	0.208	0.337	2.25E-02
Cbfa2t3	7.76E-07	-0.625	0.435	0.551	1.32E-02
Wipf1	6.94E-07	-0.629	0.382	0.514	1.18E-02
Man2b1	1.59E-08	-0.641	0.452	0.59	2.70E-04
Ei24	8.79E-07	-0.647	0.212	0.343	1.49E-02
Rdx	2.09E-08	-0.655	0.528	0.657	3.55E-04
Arrb1	1.64E-08	-0.659	0.503	0.609	2.78E-04
Pgam1	2.53E-06	-0.684	0.349	0.462	4.30E-02
Pfkfb3	2.04E-08	-0.700	0.25	0.404	3.46E-04
Nhsl2	4.37E-08	-0.702	0.305	0.458	7.41E-04
B130024G19Rik	4.11E-08	-0.720	0.198	0.343	6.98E-04
Fmn1	2.31E-06	-0.720	0.273	0.397	3.93E-02
Abhd6	2.55E-08	-0.722	0.241	0.391	4.33E-04
Inhbb	2.22E-06	-0.726	0.297	0.419	3.77E-02
Tef	1.30E-07	-0.750	0.233	0.374	2.21E-03
Cbx6	2.55E-06	-0.758	0.263	0.38	4.32E-02
Sema6d	1.30E-07	-0.771	0.36	0.49	2.21E-03
Glb1	2.01E-06	-0.774	0.258	0.378	3.42E-02
Snx25	2.18E-07	-0.776	0.269	0.404	3.70E-03
BC028528	1.69E-08	-0.779	0.234	0.382	2.86E-04
Akap11	3.64E-07	-0.803	0.187	0.313	6.18E-03
Hic1	3.31E-08	-0.819	0.204	0.348	5.63E-04
Fam102a	1.68E-06	-0.820	0.216	0.333	2.86E-02
C1qtnf6	4.93E-08	-0.896	0.14	0.268	8.37E-04
Tspan5	8.35E-07	-0.910	0.194	0.315	1.42E-02
Prelid2	2.19E-07	-0.981	0.159	0.279	3.72E-03
Cxxc5	8.47E-08	-1.056	0.114	0.229	1.44E-03
Tbc1d30	8.39E-07	-1.061	0.117	0.22	1.42E-02
Adora2a	2.47E-06	-1.090	0.097	0.19	4.20E-02
Pdpr	1.21E-06	-1.126	0.086	0.179	2.05E-02
Plod2	8.52E-08	-1.138	0.157	0.279	1.45E-03
Slc46a3	4.97E-07	-1.353	0.044	0.123	8.43E-03
Gpr183	1.81E-07	-1.871	0.035	0.11	3.07E-03
Ak4	1.99E-07	-2.638	0.008	0.058	3.38E-03

Supplementary Table 9. Curated PLVAP^{+} signatures.

C3_PLVAP_signature	C4_PLVAP_signature	C9_PLVAP_signature
Alb	Abcg2	Ackr1
Ambp	Apoa2	Actn1
Aplnr	Aqp1	Aplnr
Apoa1	Arrdc3	Bgn
Apoa2	B2m	Bnc2
Apoc1	Car4	Col15a1
Apoc3	Cd74	Col6a2
Col15a1	Cxcr4	Cpxm2
Col4a1	Dnase1l3	Csrp1
Col4a2	Esm1	Donson
Cpm	Flt1	Dusp23
Cyba	Fos	Fabp5
Cysltr1	Gja1	Fam118a
Ddit4	H2-Q10	Fam167b
Dusp23	H2-Q7	Fn1
Dusp6	Gm8909	Hapln3
Dysf	H2-D1	Htra1
Eng	H2-M2	Impdh2
Fabp1	H2-Q10	Lamb1
Fam167b	H2-Q7	Lhx6
Fgb	H2-Q10	Mmrn1
Fgg	H2-Q7	Mpzl2
Folh1	H2-DMa	Myh9
Gabrd	H2-Aa	Myl9
Gmfg	H2-Aa	Myo5a
Hecw2	H2-Ab1	Nnmt
Kdr	H2-Ea	Nuak1
Lbh	H2-Eb1	Pgf
Ldhb	H2-Eb1	Pkm
Nrp1	H2-T23	Plp2
Nxph1	Hsp90aa1	Postn
Pla2g2a	Hspa1a	Prcp
Plvap	Iglc1	Rai14
Plxnd1	Iglc2	Rgs16
Ppib	Iglc3	Rora
Rab13	Inhbb	Sele
Rasgrp3	Insr	Selp
Rbp4	Isg20	Serpine1
Serpina1b	Ivns1abp	Smad1
Serpina1e	Kcnk5	Snai1
Sparc	Lpar6	Sox4
Spink1	Pcdh17	Tagln

Spp1	Plk2	Tpd52l1
Thy1	Plvap	Tshz2
Tmem204	Slc2a3	Vwa1
Tmem255b	Sparc	Zfp521
Vtn	Sparcl1	
	Spry1	
	Stc1	
	Tgfbr2	
	Ttr	
	Ubc	
	Ugcg	

References (Author names in bold designate shared co-first authorship)

[1] Chen S, Zhou Y, Chen Y, Gu J. Fastp: An ultra-fast all-in-one FASTQ preprocessor. Bioinformatics 2018;34(17):i884-i890. https://doi.org/10.1093/bioinformatics/bty560.

[2] Angerer P, Haghverdi L, Büttner M, Theis FJ, et al. Destiny: Diffusion maps for large-scale single-cell data in R. Bioinformatics 2016;32(8):1241–3. https://doi.org/10.1093/bioinformatics/btv715.

[3] La Manno G, Soldatov R, Zeisel A, et al. RNA velocity of single cells. Nature 2018;560(7719):494–8. https://doi.org/10.1038/s41586-018-0414-6.

[4] Virshup I, Rybakov S, Theis FJ, et al. anndata: Access and store annotated data matrices. J Open Source Softw 2024;9:4371. https://doi.org/10.21105/joss.04371.

[5] Bergen V, Lange M, Peidli S, et al. Generalizing RNA velocity to transient cell states through dynamical modeling. Nat Biotechnol 2020;38:1408–14. https://doi.org/10.1038/s41587-020-0591-3.

[6] Wolf FA, Angerer P, Theis FJ. SCANPY: Large-scale single-cell gene expression data analysis. Genome Biol 2018;19. https://doi.org/10.1186/s13059-017-1382-0.

[7] **Weiler P, Lange M**, Klein M, et al. CellRank 2: unified fate mapping in multiview single-cell data. Nat Methods 2024;21:1196–205. https://doi.org/10.1038/s41592-024-02303-9.

[8] Gulati GS, Sikandar SS, Wesche DJ, et al. Single-cell transcriptional diversity is a hallmark of developmental potential. Science 2020;367:405–11. https://doi.org/10.1126/science.aax0249.

[9] **Ng MSF, Kwok I, Tan L**, et al. Deterministic reprogramming of neutrophils within tumors. Science 2024;383:eadf6493. https://doi.org/10.1126/science.adf6493.

[10] Holla P, Dizon B, Ambegaonkar AA, et al. Shared transcriptional profiles of atypical B cells suggest common drivers of expansion and function in malaria, HIV, and autoimmunity. Sci Adv 2021;7:eabg8384. https://doi.org/10.1126/sciadv.abg8384.

[11] King HW, Orban N, Riches JC, et al. Single-cell analysis of human B cell maturation predicts how antibody class switching shapes selection dynamics. Sci Immunol 2021;6:eabe6291. https://doi.org/10.1126/sciimmunol.abe6291.

[12] Victora GD, Dominguez-Sola D, Holmes AB, et al. Identification of human germinal center light and dark zone cells and their relationship to human B-cell lymphomas. Blood 2012;120:2240–8. https://doi.org/10.1182/blood-2012-03-415380.
