# Supplementary material for: Hyperinflammatory reprogramming marks the transition from MASH to HCC
Source: JHEP Rep. 2026 Oct 7;8(12):102055. doi: 10.1016/j.jhepr.2026.102055 (PMC13756514; doi:10.1016/j.jhepr.2026.102055)
Supplement: Supplementary file 3 [file mmc4.pdf]

JHEP Reports

Hyperinflammatory reprogramming marks the transition from MASH to HCC

Authors

Shawn Lu Wen Tan, Erez Israeli, Jillian Wee, ..., Immanuel Kwok, Shu Zhen Chong, Weiping Han

Correspondence

weiping_han@a-star.edu.sg (W. Han).

Graphical abstract

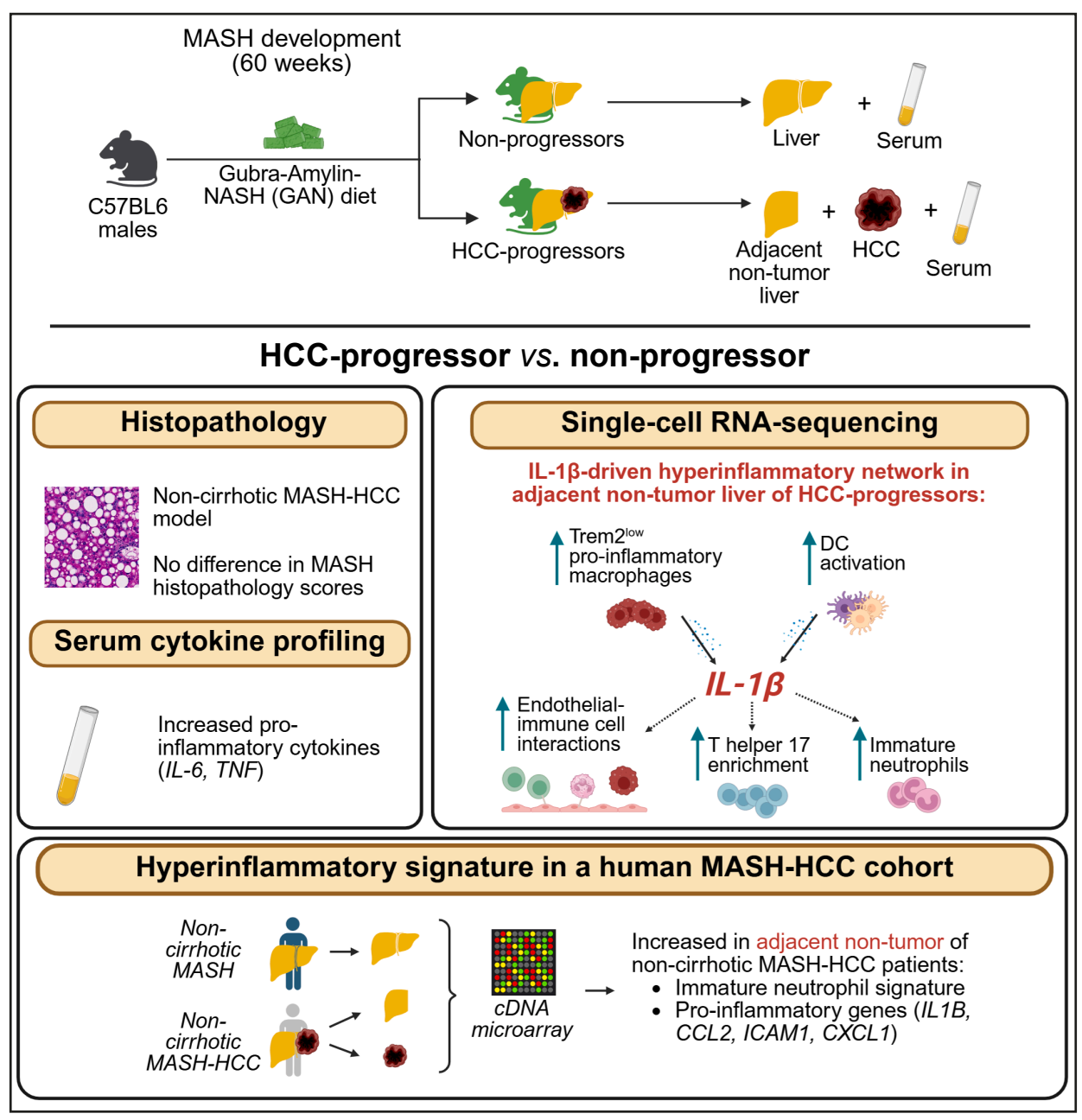

Highlights:

- Non-cirrhotic MASH-HCC progression is marked by coordinated hyperinflammatory reprogramming.
- *Trem2*^{low} macrophages and immature neutrophils accumulate in cancer-primed MASH liver.
- Dendritic and endothelial cells acquire activated, pro-inflammatory states during HCC progression.
- Myeloid-derived IL-1β emerges as a dominant signaling axis linking immune and stromal compartments.
- Human non-cirrhotic MASH-HCC recapitulates IL-1β-associated inflammatory programs.

Impact and implications:

The biological factors that distinguish patients with metabolic dysfunction-associated steatohepatitis (MASH) who progress to hepatocellular carcinoma (HCC) in the absence of cirrhosis remain poorly understood, limiting current approaches to HCC risk stratification and surveillance. Here, we identify coordinated hyperinflammatory reprogramming across immune and stromal compartments, centered on a myeloid-driven IL-1β signaling network, as a defining feature associated with non-cirrhotic MASH-HCC progression. These findings are particularly relevant to patients with non-cirrhotic MASH who may fall outside current fibrosis-based HCC surveillance strategies, and raise the possibility that inflammatory signatures could complement existing clinical parameters to identify individuals at elevated HCC risk. Prospective studies and functional perturbation will be required to establish whether this hyperinflammatory state precedes and contributes causally to HCC development, and to determine its utility for patient stratification and preventive intervention.

https://doi.org/10.1016/j.jhepr.2026.102055

© 2026 The Author(s). Published by Elsevier B.V. on behalf of European Association for the Study of the Liver (EASL). This is an open access article under the CC BY license (http://creativecommons.org/licenses/by/4.0/).

Hyperinflammatory reprogramming marks the transition from MASH to HCC

Shawn Lu Wen Tan[1], Erez Israeli[1], Jillian Wee[1], Hui Min Tan[1], Ravisankar Rajarethinam[1], Immanuel Kwok[2], Shu Zhen Chong[2], Weiping Han[1,*]

JHEP Reports **2026**. vol. 8 | 1–18

Check for updates

Background & Aims: Up to 40% of metabolic dysfunction-associated steatohepatitis (MASH)-related hepatocellular carcinoma (HCC) occurs in the absence of cirrhosis. Yet the biological features underlying non-cirrhotic MASH-to-HCC progression remain poorly understood. We sought to define the cellular and molecular programs associated with non-cirrhotic MASH-to-HCC progression.

Methods: We integrated histopathological and biochemical assessments, bulk and single-cell RNA sequencing, serum cytokine profiling, and multiplex immunohistochemistry in Gubra-Amylin-NASH diet-fed mice that remained HCC-naïve or developed non-cirrhotic HCC. Key findings were evaluated in an independent human cohort with non-cirrhotic MASH and MASH-HCC.

Results: Body composition metrics, fibrosis severity, histopathology, and liver injury markers poorly distinguished HCC progressors from non-progressors. In contrast, bulk transcriptomics identified prominent immune dysregulation in cancer-primed adjacent non-tumor liver. Single-cell RNA sequencing revealed coordinated hyperinflammatory reprogramming characterized by loss of *Trem2*$^{+}$ macrophages, expansion of *Trem2*^{low} pro-inflammatory macrophages, accumulation of immature neutrophils, T helper 17 skewing and heightened activation of dendritic and endothelial cells. Cell-cell communication analysis identified IL-1 as the most altered signaling pathway in HCC progressors (pathway distance = 7.95, top-ranked), with myeloid-derived IL-1β signaling emerging as a dominant axis targeting endothelial and immune populations. Elevated circulating IL-6 (154.3 *vs.* 30.1 pg/ml, p = 0.065) and TNF (25.2 *vs.* 16.0 pg/ml, p = 0.062) in HCC progressors were consistent with a systemic hyperinflammatory phenotype. Human non-cirrhotic MASH-HCC adjacent non-tumor tissues similarly exhibited increased *IL1B* expression ($\log_2$ expression = 4.21 *vs.* 4.03, p <0.01) and enrichment of IL-1β-responsive inflammatory programs.

Conclusions: Non-cirrhotic MASH-to-HCC progression is associated with coordinated hyperinflammatory reprogramming across immune and stromal compartments. Myeloid-derived IL-1β is a central signaling axis within the cancer-permissive MASH microenvironment, providing a potential basis for HCC risk stratification and preventive intervention.

© 2026 The Author(s). Published by Elsevier B.V. on behalf of European Association for the Study of the Liver (EASL). This is an open access article under the CC BY license (http://creativecommons.org/licenses/by/4.0/).

Introduction

Up to 30-50% of metabolic dysfunction-associated steatohepatitis (MASH)-related hepatocellular carcinoma (HCC) cases develop in the absence of cirrhosis, placing many patients outside current HCC surveillance strategies.[1] This is a challenge compounded by the fast-growing global burden of MASH.[2] Therapeutic options for advanced MASH-related HCC remain relatively limited, with evidence suggesting that patients with MASH-HCC are relatively resistant to immune checkpoint inhibitor combination therapies compared to HCCs derived from other etiologies (*e.g*. viral hepatitis).[3] Collectively, these factors underscore the need to define the mechanisms driving HCC development in non-cirrhotic MASH to improve cancer prevention and patient risk stratification.

MASH is driven by a confluence of factors, including lipotoxicity, endoplasmic reticulum stress, and chronic inflammation, resulting in sustained liver injury and fibrosis.[2,4] MASH studies have implicated specific immune subsets in HCC development, including neutrophils,[5] immunoglobulin A-producing B cell subsets,[6] and 'auto-aggressive' cytotoxic CD8^{+} T cell populations,[3,7] which emerge in pre-malignant MASH livers and promote hepatocarcinogenesis. These findings highlight the pivotal role of liver immunity in MASH-associated HCC. However, an unbiased, integrative view of how diverse immune populations evolve in concert across the MASH-to-HCC transition remains lacking, limiting our understanding of its immunological underpinnings.

Here, we employ a diet-induced murine model of MASH that progresses to non-cirrhotic HCC with partial penetrance, enabling dissection of features distinguishing HCC progressors from non-progressors. Bulk transcriptomics of adjacent non-tumor liver and serum cytokine profiling revealed a hyperinflammatory state in HCC progressors compared with non-progressors. Single-cell transcriptomics further generated a single-cell atlas spanning HCC-naïve MASH liver, adjacent non-tumor tissue and HCC, providing a cross-sectional view of

* Corresponding author. Address: 11 Biopolis Way #01-02, Helios, 138667, Singapore. Tel.: +65 64788721.
E-mail address: weiping_han@a-star.edu.sg (W. Han).
https://doi.org/10.1016/j.jhepr.2026.102055

the MASH-to-HCC transition. Together, our findings establish a foundation for further mechanistic dissection of HCC-promoting immune alterations in MASH that may inform future strategies for HCC prevention and early detection.

Materials and methods

In vivo mouse studies

Six-week-old male C57BL6/J mice (Invivos) were acclimatized until 8 weeks of age, then fed either GAN diet (Research Diets, D09100310i) or low-fat diet (LFD) (D09100304i) for 16, 32 or 55–60 weeks. Body weight was measured weekly and body composition monthly using EchoMRI. At harvest, blood was collected by cardiac puncture for serum preparation, and livers were weighed, photographed and processed for downstream analyses. Liver tissues were either snap-frozen, preserved in RNAlater or formalin-fixed and paraffin-embedded. Serum and frozen tissues were stored at −80°C. Mice were maintained at the A*STAR Biological Resource Centre under Institutional Animal Care and Use Committee approval (IACUC No. 231792).

Serum proteomics

Mouse serum samples were analyzed using the Olink Target 48 Mouse Cytokine Panel (Olink Proteomics, Uppsala, Sweden) based on proximity extension assay (PEA) technology.

Pathology evaluation of H&E liver sections

H&E- and Sirius Red-stained liver sections were evaluated by a certified veterinary pathologist blinded to treatment groups. Steatosis was scored as 0 (<5% hepatocytes affected), 1 (5–33%), 2 (>33–66%) or 3 (>66%); lobular inflammation as 0 (no foci), 1 (<2 foci), 2 (2–4 foci) or 3 (>4 foci per 200 × field); and hepatocyte ballooning as 0 (none), 1 (few ballooned hepatocytes) or 2 (many/prominent ballooned hepatocytes). MASLD activity score was calculated as the sum of these three scores. Fibrosis was staged as 0 (none), 1A (mild zone 3 perisinusoidal), 1B (moderate zone 3 perisinusoidal), 1C (portal/periportal), 2 (perisinusoidal and portal/periportal), 3 (bridging) or 4 (cirrhosis). Large lipogranulomas were scored as absent (0) or present (1).

Single-cell RNA sequencing

Liver single-cell suspensions were labeled with TotalSeq-B anti-mouse hashtag antibodies (BioLegend), washed and resuspended in PBS with 1% bovine serum albumin. Two pools of five samples were generated using equal cell numbers and non-overlapping hashtags. Each pool was loaded onto the Chromium iX (10x Genomics) targeting 21,500 recovered cells. Gene expression and Feature Barcode libraries were prepared using the 10x Genomics Next GEM Single Cell 3′ v3.1 and 3′ Feature Barcode kits according to the manufacturer's protocol. Libraries were sequenced on an Illumina NovaSeq 6000 (2 × 151 bp), targeting 50,000 reads/cell for gene expression and 5,000 reads/cell for Feature Barcodes.

Pre-processing of scRNA-sequencing data, quality control and clustering

Raw reads were processed using Cell Ranger v7.1.0 and analyzed using Seurat v5.0.0. Quality control was performed on individual samples using mitochondrial read percentage, total counts, detected genes and count-to-gene relationships, as detailed in Supplementary Methods. Data were normalized using *SCTransform*, integrated using *IntegrateData*, and subjected to clustering and uniform manifold approximation and projection (UMAP) visualization. Clusters and subclusters were annotated based on differential marker expression.

Cell-cell communication analysis

Cell–cell communication was inferred using CellChat v2.1.0[39] after excluding hepatocytes and cholangiocytes. GAN and GAN-Adj.NT cells were analyzed separately, with communication probabilities inferred between subclusters using *computeCommunProb* (type = "truncatedMean"). *LiftCellChat* was used to harmonize subcluster identities across groups, and subclusters with <10 cells in either group were excluded from comparative analyses.

mRNA *in situ* hybridisation

Camp mRNA was detected in 5-μm mouse FFPE liver sections using a murine *Camp* RNAscope probe (ACDbio, 446231) and RNAscope 2.5 HD Reagent Kit–Red (ACDbio, 322373) according to the manufacturer's instructions.

Processing of human MASH-HCC microarray dataset

Human MASH and MASH-HCC liver microarray data and metadata were obtained from GEO (GSE164760). Probes lacking unique gene annotations or with ambiguous RefSeq assignments were excluded, and multiple probes mapping to the same gene were averaged. Gene-level expression values were $\log_2$-transformed and quantile-normalized using *preprocessCore*. PCA outliers (|z-score| >2.5 for PC1 or PC2) were excluded. Single-sample gene set enrichment analysis was performed using GSVA v1.44.5 (method = "ssgsea").

Multiplex immunohistochemistry

Multiplex immunohistochemistry of liver FFPE sections was performed using the MACSima platform as previously described.[50] Following deparaffinization, antigen retrieval, permeabilization and blocking, sections underwent iterative staining with FITC-, PE- and APC-conjugated antibodies and imaging on the MACSima instrument. Images were processed using MACS iQ View software. Antibodies used were anti-TREM2 (Abcam, ab305103), anti-F4/80 (Miltenyi Biotec, 130-116-499), anti-CD31 (130-111-710), anti-CD146 (130-118-407), anti-albumin (Proteintech, 16475-1-AP) and anti-α-SMA (Miltenyi Biotec, 130-123-363).

Quantification and statistical analyses

Statistical analyses were performed in R. Logistic regression was performed using *glm(family = "binomial")*. Multiple-testing correction used the Benjamini–Hochberg method unless otherwise stated. Significance was denoted as: p >0.1, n.s.; p <0.1, #; p <0.05, *; p <0.01, **; p <0.001, ***.

Results

A murine model of non-cirrhotic MASH-to-HCC progression with partial HCC penetrance

To model MASH-HCC development, we challenged C57BL6 male mice *ad libitum* with the Gubra-Amylin-NASH (GAN) diet,[8] which is composed of 40% kcal fat, 20% kcal fructose and 2% kcal cholesterol for up to 60 weeks. A calorie-matched diet containing 10% kcal fat was used as a control diet (LFD). A total of 89 and 52 mice were placed on GAN and LFD diets, respectively, and sampled at 16, 32 and 55-60 weeks (hereafter 60 weeks) to characterize MASH-to-HCC progression (Fig. 1A). As expected, GAN diet exposure increased body weight, fat mass and liver weight and impaired glucose homeostasis (Fig. S1A–C). By 16 weeks, >90% of GAN-fed mice exhibited significant hepatic steatosis (score ≥2), preceding hepatocyte proliferation (Ki67) and fibrosis (Sirius Red), which emerged at 32 and 60 weeks, consistent with progression from steatosis to MASH (Fig. S1D and E). Bridging fibrosis (stage 3, 42%), increased lobular inflammation (score ≥2, 53%), high MASLD activity scores (score ≥5, 37.5%) and lipogranulomas (87.5%) emerged only in the 60-week GAN cohort, indicating advanced MASH (Fig. S1D and E). Hepatocyte ballooning was infrequent (3/64 mice; Fig. S1E), consistent with its limited prevalence in murine MASH models.[9]

At 60 weeks, 20/64 GAN mice (31.2%) developed visible liver tumors, with none observed at earlier timepoints (Fig. 1B). Tumor burden was generally low, with 19/20 tumor-bearing (TB) mice harboring only 1–2 visible nodules (Figs 1C and S1F). Histopathological evaluation of H&E-stained liver sections from TB mice identified HCC in 7/20 mice, hepatocellular adenoma (HCA) in 8/20, both HCC and HCA in 4/20, and hepatocholangiocellular adenoma (HCCA) in 1/20 (Fig. 1D). HCCs exhibited diverse histological subtypes resembling those observed in human HCC, including trabecular HCCs with varying steatosis (Fig. S1G–I), steatohepatitic HCCs (Fig. S1J), clear cell-like variants (Fig. S1K,L), and mixed solid-trabecular patterns (Fig. S1M,N). One HCC displayed signs of vascular invasion, indicative of advanced disease (Fig. S1O). Thus, this model recapitulates MASH-to-HCC progression with partial HCC penetrance and histological features of human HCC.

To focus on MASH-to-HCC progression, we excluded 60-week GAN mice with only HCAs or HCCAs and stratified the remaining 60-week GAN cohort as non-tumor-bearing (NTB, n = 44) or HCC-bearing (HB, n = 11). Notably, 8/11 HB mice developed HCC on a background of stage ≤2 fibrosis, and none had cirrhosis (stage 4) (Fig. 1E,F). Fibrosis severity also trended lower in HB than NTB livers, together indicating that advanced fibrosis was not required for HCC development in this model. HB livers also showed a modest trend towards lower steatosis, potentially reflecting altered lipid metabolism, while other histopathological features were comparable between groups (Fig. S2A).

We next compared body composition, serum alanine aminotransferase (ALT) and total bile acids as markers of hepatocellular injury and hepatobiliary dysfunction, respectively, as well as the HCC biomarker alpha-fetoprotein (AFP), between HB and NTB mice and assessed their association with HCC progression by logistic regression. Serum AFP was the only marker significantly elevated in HB mice (Fig. S2B) but only modestly predictive of HCC onset (odds ratio = 1.02, p = 0.007) (Fig. 1G). Together, these findings indicate that HCC can emerge in the GAN model without advanced fibrosis or overt systemic and biochemical features that distinguish HCC progressors from non-progressors.

Bulk liver transcriptomics reveals immune dysregulation in adjacent non-tumor liver of HCC progressors

Non-tumor tissues adjacent to tumors are increasingly recognized as cancer-primed fields harboring molecular and microenvironmental alterations that promote or permit malignant transformation.[10] To identify mechanisms underlying HCC development in MASH, we sequenced the bulk transcriptomes of NTB livers (n = 27) and adjacent non-tumor samples from HB livers (HB-Adj.NT, n = 6) (Fig. 1H). Pairwise comparison identified 48 differentially expressed genes (DEGs), all of which were upregulated (fold change >2, adjusted p <0.05) in HB-Adj.NT tissues (Fig. 1I, Table S1). These DEGs were enriched for immune-related transcripts, including the B-cell chemoattractant, *Cxcl13*, the macrophage scavenger receptor, *Marco,* myeloid activation markers *Ms4a8a*, *Fpr1, Fpr2*, and *S100a8* and myeloid chemoattractants *Ppbp*, *Ccl8*, and *Ccl12* (Fig. 1I). Despite the modest number of DEGs, KEGG and Reactome gene set overrepresentation analyses robustly identified enrichment of immune pathways, including “Cytokine-cytokine receptor interaction” and “Neutrophil degranulation” (Fig. 1J,K). Neutrophils represented the most enriched immune cell signature, suggesting their accumulation in adjacent non-tumor liver of HB mice (Fig. 1L, Table S2). These findings suggest heightened immune activation in the cancer-primed liver of HCC progressors.

To validate the heightened inflammatory state in HB mice, we next profiled 39 serum cytokines in LFD (n = 6), NTB (n = 14) and HB (n = 7) mice. Several pro-inflammatory cytokines, including IL-6, TNF, CSF1, CSF3, CXCL1, IL-2 and CCL12, were elevated in HB mice (Figs 1M and S2C). Notably, IL-6 and CSF3 promote neutrophil production and expansion,[11] consistent with the neutrophil enrichment identified by bulk transcriptomics. CCL12, a CCR2 ligand associated with monocyte recruitment, was also among the upregulated DEGs in HB-Adj.NT liver. Together, these findings support a heightened systemic and hepatic inflammatory state in HCC progressors.

A cross-sectional single-cell atlas of the MASH-to-HCC transition

We next performed single-cell RNA (scRNA)-sequencing of ten 60-week liver samples comprising two LFD controls, two NTB GAN mice, and three paired adjacent non-tumor (GAN-Adj.NT) and tumor (GAN-Tumor) samples from three HB mice (Fig. S3A). Histopathological evaluation of matched liver sections confirmed all tumor samples as HCC (Fig. S3B). Steatosis, lobular inflammation, MASLD activity and fibrosis scores were generally comparable between HB and NTB GAN mice (hereafter GAN) with none of the HB livers exceeding stage 2 fibrosis (Fig. S3A).

A total of 19,178 cells passed quality control, comprising 3,280 LFD, 4,153 GAN, 5,978 GAN-Adj.NT and 5,767 GAN-Tumor cells, with all samples well represented (Fig. S3C). Louvain clustering and annotation using established markers

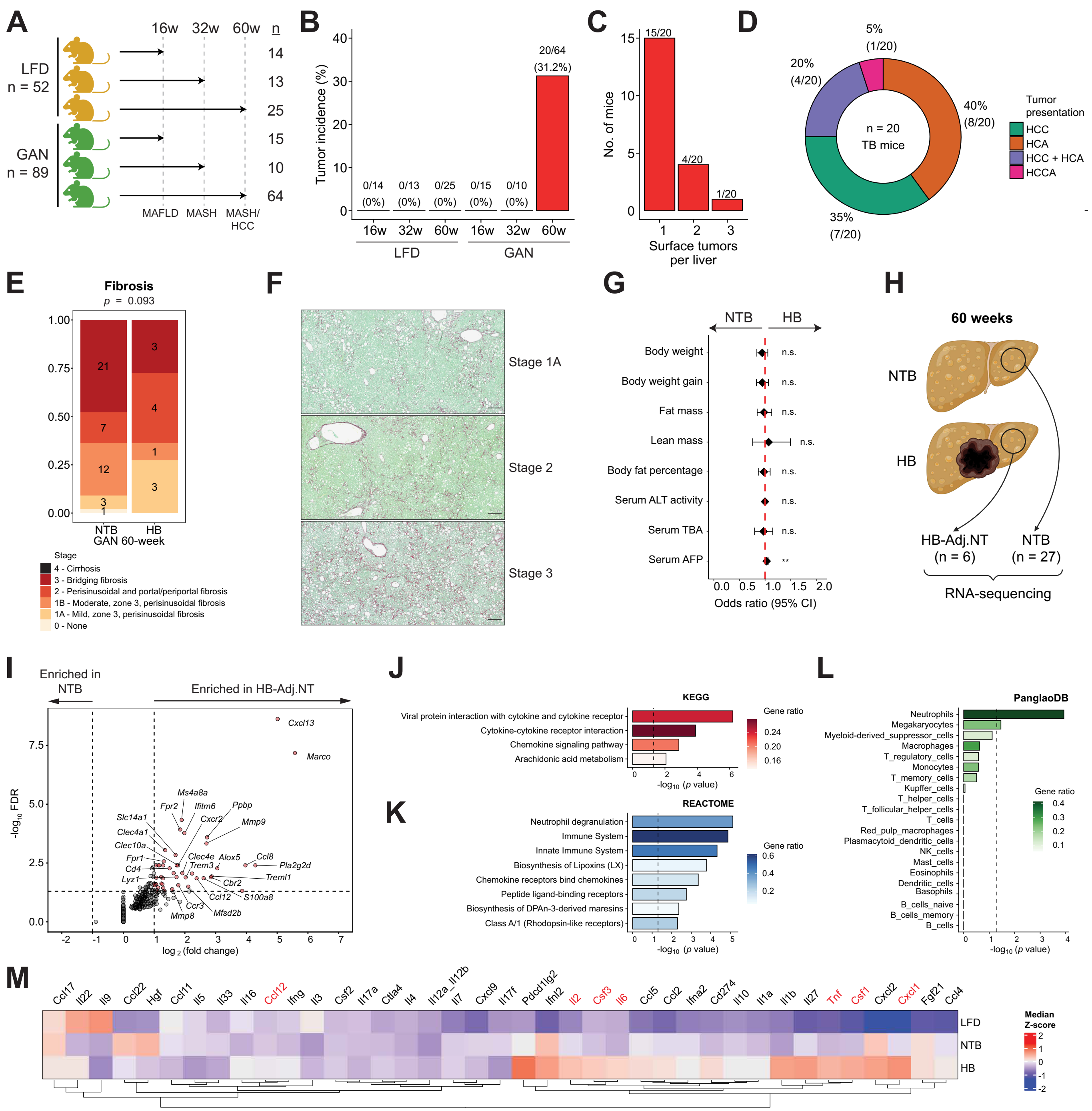

Fig. 1. A dietary model of non-cirrhotic MASH-to-HCC progression with partial HCC penetrance highlights an augmented local and systemic inflammatory response in HCC progressors. (A) Experimental set-up for the GAN murine model of MASH-to-HCC progression. (B) Liver tumor incidence across MASH-to-HCC progression in the GAN model. (C) Number of surface tumor nodules more than 2 mm^3 in each tumor-bearing liver. (D) Tumor presentation of tumor-bearing mice. (E) Histopathology scoring of NTB and HB 60-week GAN liver sections for fibrosis stage. Fisher's exact test. (F) Representative images of non-tumor regions of 60-week HB liver sections stained with picrosirius red and counterstained with Fast Green. Liver sections from three independent HB mice displaying the indicated fibrosis stages are shown. Scale bar represents 200 µm. (G) Logistic regression of metabolic and serum parameters of 60-week GAN-treated NTB and HB mice. (H) Schematic of bulk RNA-sequencing of 27 NTB liver tissues and 6 HB-Adj.NT tissues from HB mice. (I) Differential expression analysis of bulk transcriptomes of 60-week GAN-treated Adj.NT liver samples of HB mice and NTB livers. BH-adjusted *p* values. (J) KEGG pathway enrichment of 48 upregulated DEGs in HB-Adj.NT tissues. *p* values calculated using the g:SCS algorithm of gProfiler2. (K) Reactome pathway enrichment of 48 upregulated DEGs in HB-Adj.NT tissues. *p* values calculated using the g:SCS algorithm of gProfiler2. (L) Immune signature enrichment using signatures from PanglaoDB of 48 upregulated DEGs in HB-Adj.NT tissues. *p* values calculated using the g:SCS algorithm of gProfiler2. (M) Relative abundance of cytokines in sera of 60-week mice measured using the Olink Target 48 mouse cytokine panel. Highlighted in red are cytokines that show a significant difference (p <0.1) between NTB and HB mice. *Post hoc* Dunn's test following Kruskal-Wallis test, Benjamini-Hochberg adjusted *p* values. Adj.NT, adjacent non-tumor; BH, Benjamini-Hochberg; DEG, differentially expressed gene; GAN, Gubra-Amylin-NASH; HB, HCC-bearing; HCA, hepatocellular adenoma; HCC, hepatocellular carcinoma; HCCA, hepatocholangiocellular adenoma; MASH, metabolic dysfunction-associated steatohepatitis; NTB, non-tumor-bearing.

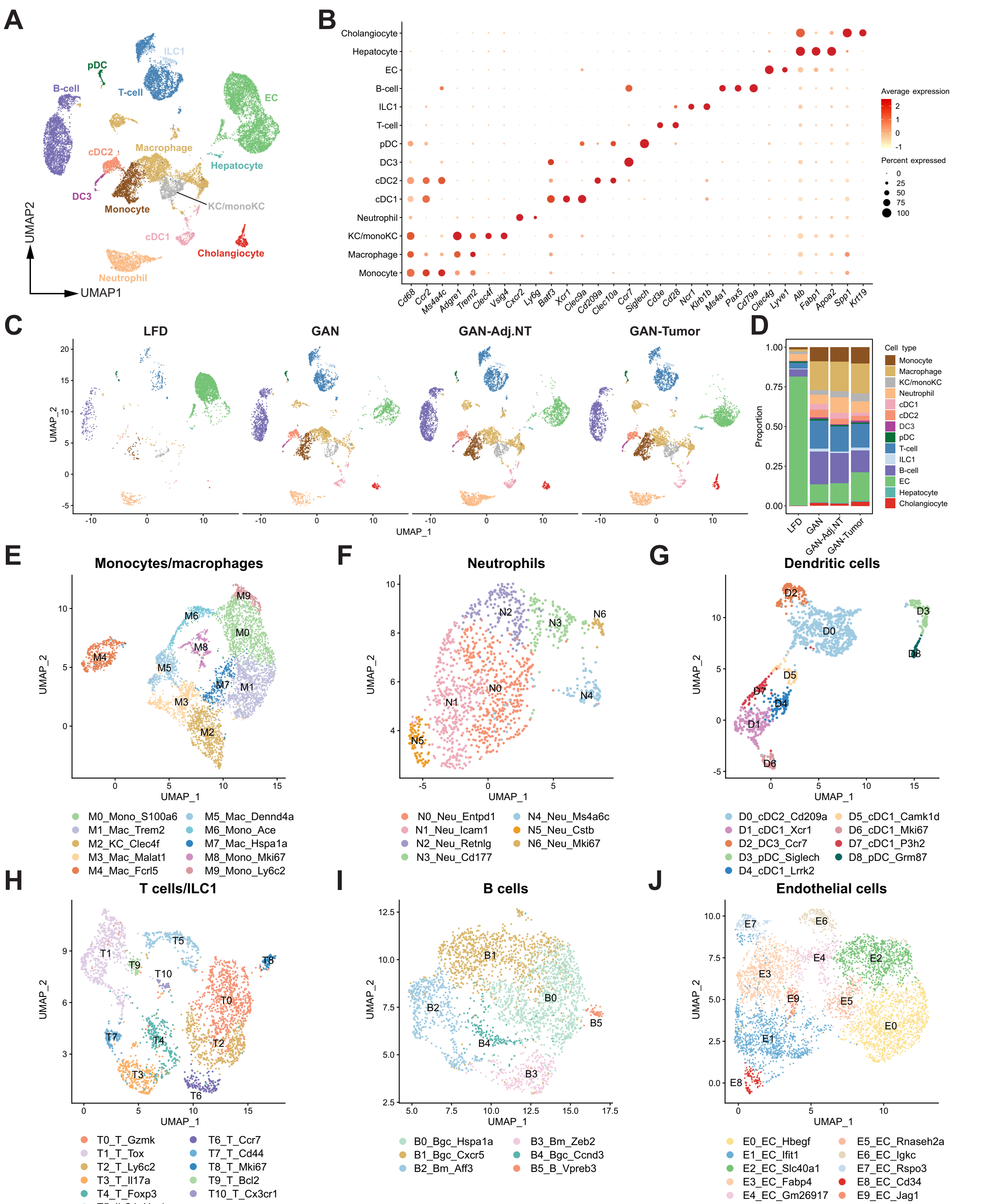

Fig. 2. A cross-sectional single-cell atlas of the MASH-to-HCC transition. (A) UMAP plot of 19,178 cells showing broad cell types captured. (B) Dot plot of marker genes for each cell type. (C) UMAP plot separated by sample group. (D) Proportions of each cell type across sample groups. (E) UMAP plot of monocytes and macrophages after re-clustering into 10 subclusters. (F) UMAP plot of neutrophils after re-clustering into 7 subclusters. (G) UMAP plot of dendritic cells after re-clustering into 9 subclusters. (H) UMAP plot of T cells and ILC1s after re-clustering into 11 subclusters. (I) UMAP plot of B cells after re-clustering into 6

identified 14 major cell types visualized by UMAP (Fig. 2A,B). Myeloid populations included monocytes (*Ccr2*), macrophages (*Adgre1, Trem2*), Kupffer cells (*Clec4f*), neutrophils (*Cxcr2*), conventional type 1 dendritic cell (cDC1) (*Xcr1*), cDC2 (*Cd209a*), migratory DC3 (*Ccr7, Batf3*) and plasmacytoid dendritic cells (pDCs) (*Siglech*) (Figs 2B and S3D). Lymphoid populations comprised T cells (*Cd3e*), B cells (*Pax5*) and ILC1s (*Ncr1, Klrb1b*), while non-immune populations included endothelial cells (*Clec4g*), cholangiocytes (*Spp1, Krt19*) and hepatocytes (*Alb, Apoa2*). Despite their abundance in the liver, hepatocytes were poorly captured, likely due to limited viability during tissue dissociation, while hepatic stellate cells were not detected. Nevertheless, the dataset captured diverse immune and stromal populations across the MASH-to-HCC transition.

LFD samples were dominated by ECs, which comprised >75% of LFD cells and approximately half of all ECs captured. LFD- and GAN-associated ECs occupied distinct UMAP regions, indicating substantial endothelial transcriptomic reprogramming in MASH (Figs 2C,D and S3D). In contrast, GAN-associated samples (GAN, GAN-Adj.NT and GAN-Tumor) were predominantly composed of immune cells, consistent with heightened inflammation in MASH (Figs 2D and S3D-F). Broad cell-type composition was largely similar across GAN-associated samples, except for neutrophils, which were nearly doubled in GAN-Adj.NT compared with GAN livers (9.9% *vs*. 5.6%), consistent with their enrichment in bulk transcriptomics (Figs 1L and S3G).

To resolve more subtle cellular changes across the MASH-to-HCC transition, we separately re-clustered six compartments: monocytes/macrophages, neutrophils, dendritic cells, T cells/ILC1s, B cells and ECs. Cholangiocytes and hepatocytes were excluded due to low cell numbers. This higher-resolution analysis identified 53 subclusters, which are characterized below (Fig. 2E–J).

Depletion of *Trem2*⁺ macrophages and expansion of *Trem2*^{low} pro-inflammatory macrophages in HCC progressors

Re-clustering of monocytes and macrophages identified 10 subclusters (M0–M9; Fig. 3A) with differential enrichment across sample groups quantified by chi-square analysis (Fig. 3B,C, see supplementary methods). Four monocyte populations were identified: three classical monocyte clusters (M0, M8, M9; *Ccr2*$^+$*Ly6c2*$^+$) and one *Ace*$^+$ patrolling monocyte cluster (M6). M8 monocytes additionally expressed proliferation-associated genes, including *Mki67*, consistent with a differentiating monocyte population. Notably, *Ly6c2*$^+$ M9 infiltrating monocytes and M8 differentiating monocytes were strongly enriched in GAN-Adj.NT compared with GAN livers, indicating enhanced monocyte recruitment and differentiation in the cancer-primed microenvironment (Fig. 3B–D).

Macrophages were segregated into six subclusters. The largest, M1, expressed lipid-associated macrophage (LAM) markers (*Trem2, Gpnmb, Cd63*), identifying them as *Trem2*$^+$ LAMs, which have reported protective functions in MASH.[12,13] Notably, *Trem2*$^+$ LAMs were enriched in GAN livers but diminished in GAN-Adj.NT and GAN-Tumor tissue, suggesting progressive depletion during HCC transition (Fig. 3B–D). M2 expressed *Clec4f* and *Pcolce2*, consistent with Kupffer cells (KCs) (Fig. 3D). Compared with LFD KCs, GAN-associated KCs showed marked loss of *Timd4* and *Cd163*, consistent with a predominantly monocyte-derived origin[14] (Fig. S4A). *Trem2* expression was also increased in KCs from GAN and GAN-Adj.NT compared to LFD KCs, suggesting that monocyte-derived KCs may acquire LAM-like tissue-reparative functions.

The remaining macrophage subclusters (M3, M4, M5, M7) were enriched in HCC-transitioned livers (Fig. 3B). M3-M5 macrophages showed negligible *Trem2* expression and low enrichment for a 22-gene LAM signature[12] (Table S3) and lipid metabolism pathways, leading us to annotate them as *Trem2*^{low} macrophages (Fig. 3E,F). Compared with M1 LAMs, M3-M5 macrophages were more differentiated and exhibited reduced metabolic, macrophage activation and antigen presentation programs, consistent with chronically stimulated macrophage states with diminished function (Figs 3F and S4B,C). Notably, M4 and M5 showed increased *Il1b* expression and enrichment of immune activation pathways, indicating a pro-inflammatory phenotype (Fig. 3E,F). Thus, HCC transition was associated with a shift from *Trem2*$^+$ LAMs towards *Trem2*^{low} pro-inflammatory macrophage states.

M7 macrophages were highly enriched in tumors (Fig. 3B) and expressed *Trem2*, albeit at reduced levels compared to M1 LAMs, alongside stress response genes (*Hspa1a, Egr1*) and the pro-inflammatory cytokines *Il1b* and *Tnf* (Fig. 3D,E). They were also enriched for inflammatory Hallmark pathways, including TNFα/NFκB, IL6/JAK/STAT3 and type I/II interferon signaling (Fig. 3F), together identifying M7 as a stress-responsive, pro-inflammatory tumor-associated macrophage population.

To investigate the origins of M7 macrophages, we mapped the five macrophage populations (M1, M3, M4, M5 and M7) by diffusion analysis.[15] M1, M7, M3 and M5 formed a transcriptional continuum, while M4 remained distinct (Fig. S4D). RNA velocity revealed strong directional vectors from M1 towards M7, suggesting a transition from *Trem2*$^+$ LAMs to the M7 state (Fig. S4D). Consistent with this trajectory, *Trem2* showed negative velocity residuals in M1 and M7 macrophages, indicating progressive *Trem2* downregulation (Fig. S4E).

Compared with M1 LAMs, M7 macrophages were enriched for genes previously linked to cancer cell proliferation (*Pde4c, Cd81*)[16,17] and immunosuppression (*Hes1, Kdm6b, Stk17b*)[18–20] in the tumor microenvironment (Fig. S4F). Thus, our analyses suggest that *Trem2*$^+$ macrophages undergo a transcriptional transition within tumors, marked by loss of the LAM program and acquisition of a stress-activated phenotype with pro-inflammatory, tumor-promoting and immunosuppressive features.

Given the progressive loss of *Trem2*$^+$ LAMs and accumulation of *Trem2*^{low} macrophage states identified by scRNA-sequencing, we next examined their spatial organization by multiplex immunohistochemistry. Murine liver tissues were

subclusters. (J) UMAP plot of ECs after re-clustering into 10 subclusters. cDC, conventional dendritic cell; pDC, plasmacytoid dendritic cell; EC, endothelial cell; GAN, Gubra-Amylin-NASH; HCC, hepatocellular carcinoma; ILC1, innate lymphoid cell type 1; KC, Kupffer cell; MASH, metabolic dysfunction-associated steatohepatitis; UMAP, uniform manifold approximation and projection.

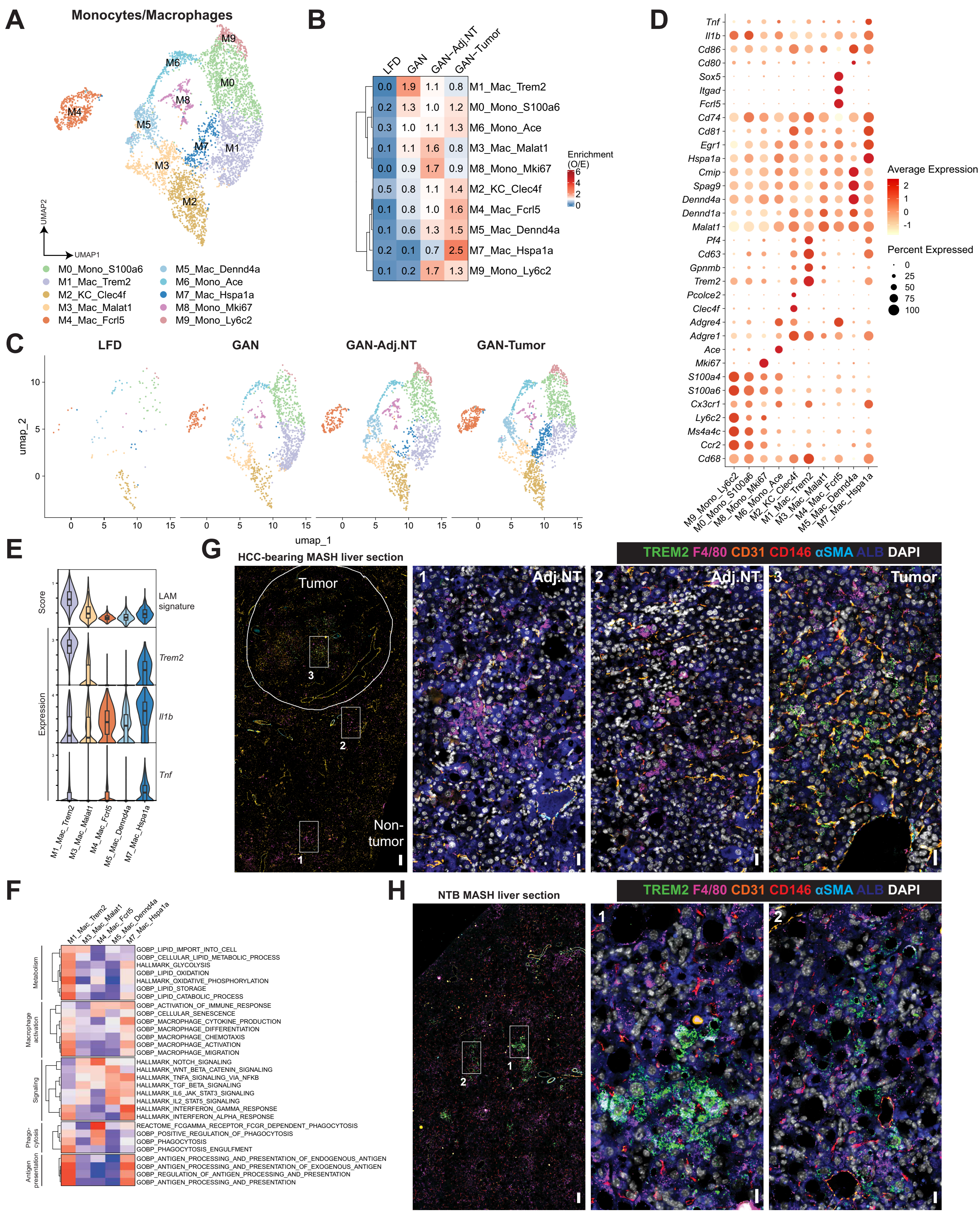

Fig. 3. Depletion of *Trem2*⁺ macrophages and accumulation of *Trem2*^{low} pro-inflammatory macrophages in HCC progressors. (A) UMAP of monocytes and macrophages showing 10 subclusters. (B) Enrichment scores of monocytes and macrophages. (C) UMAP of monocytes and macrophages separated by sample group. (D) Dot plot of marker genes for monocytes and macrophages. (E) Violin plot showing the enrichment score for the LAM signature (top row) and expression levels of *Trem2, Il1b* and *Tnf* across macrophage subclusters. (F) Single-cell gene set enrichment Z-scores averaged across macrophage subclusters. (G) Multiplex

stained for macrophage/LAM markers (TREM2, F4/80), endothelial markers (CD31, CD146), hepatocytes (ALB) and activated myofibroblast/smooth muscle cell marker (α-SMA). TREM2 antibody specificity was validated in CRISPR/Cas9-engineered *Trem2*-knockout RAW264.7 macrophages (Fig. S4G). In HCC-transitioned MASH liver, F4/80$^+$TREM2^{low} macrophages predominated in the adjacent non-tumor regions (Fig. 3G, regions 1 and 2). In contrast, F4/80$^+$ TREM2$^+$ macrophages were enriched within the tumor core and closely associated with endothelial and myofibroblast stromal networks (Fig. 3G, region 3), suggesting potential crosstalk with endothelial cells and fibroblasts within the tumor microenvironment. Whether the F4/80$^+$TREM2$^+$ macrophages observed within tumors correspond to M1 LAMs, *Trem2*$^+$ KCs (M2), or M7 macrophages remains unclear. In contrast, distinct clusters of F4/80$^+$ TREM2$^+$ macrophages were abundant throughout the liver parenchyma of the NTB GAN liver, though F4/80$^+$ TREM2^{low} macrophages were also present and uniformly distributed (Fig. 3H, regions 1 and 2). Some F4/80$^+$ TREM2$^+$ macrophages formed crown-like structures around lipid-laden hepatocytes or residual lipid droplets, consistent with macrophage-mediated clearance of damaged hepatocytes. Thus, these spatial findings corroborate our scRNA-sequencing data, supporting a shift from reparative *Trem2*$^+$ LAMs towards *Trem2*^{low} macrophage states within the cancer-primed Adj.NT microenvironment.

Enrichment of immature neutrophils in adjacent non-tumor regions of HCC-transitioned livers

Subclustering of neutrophils identified seven populations (N0–N6; Fig. 4A), of which N2, N3, N4 and N6 were preferentially enriched in GAN-Adj.NT compared with GAN livers, with N3 showing the strongest enrichment (Fig. 4B,C). N3 and N6 highly expressed *Cd177, Ngp, Camp* and *Ltf*, markers of immature bone marrow neutrophils[21] (Fig. 4D). N6 additionally expressed the proliferation marker *Mki67*, consistent with a cycling population at an earlier stage of neutrophil differentiation than N3.

To validate the immature states of N6 and N3 neutrophils, we mapped our cells onto a published single-cell dataset spanning neutrophil development across murine bone marrow, peripheral blood, spleen and pancreatic tumors.[22] N6 overlapped predominantly with bone marrow-derived preNeu cells, whereas N3 mapped to the immature IMM-1, IMM-2 and MAT1 populations (Fig. S5A–C). CytoTRACE and RNA velocity-based CellRank2 trajectory inference further supported their immature states, with N6 predicted as an initial state transitioning towards N3 (Figs 4E-G and S5D,E). Notably, a subset of N3 cells showed minimal RNA velocity and was predicted by CellRank2 as a terminal state (Figs 4G and S5F), suggesting that N3 represents a stable immature neutrophil state that accumulates in GAN-Adj.NT liver.

We further validated this finding by *in situ* hybridisation of *Camp* mRNA, a marker of immature neutrophils, which revealed significant enrichment of *Camp*$^+$ clusters in adjacent non-tumor regions of HB livers (Fig. 4H,I). To assess whether immature neutrophils similarly accumulate in human MASH-HCC, we derived a stringent eight-gene signature specific to N3/N6 neutrophils, of which five genes have human orthologues (*CAMP, CD177, GPR27, ITGB2, LTF*; Fig. S5G). In a human MASH/MASH-HCC transcriptomic cohort,[23] this signature was markedly enriched in adjacent non-tumor tissues from MASH-HCC compared with HCC-naïve MASH liver (Fig. 4J), supporting conservation in human disease. As immature neutrophils are normally restricted to the bone marrow but can enter circulation during inflammation-driven emergency granulopoiesis,[11] their accumulation in GAN-Adj.NT liver further supports a hyperinflammatory microenvironment associated with HCC progression.

N4 neutrophils, also enriched in HCC-transitioned livers (Fig. 4B), co-expressed neutrophil (*Cxcr2, Csf3r*) and monocyte (*Ms4a6c, Ace, Ccr2*) markers (Fig. 4D), resembling previously described neutrophil-like monocytes.[24] These cells emerge during severe inflammatory conditions, including tissue injury and lipopolysaccharide exposure, through emergency monopoiesis. Their enrichment in HCC-transitioned livers therefore provides further evidence of a heightened inflammatory state.

Given the marked enrichment of immature neutrophils in GAN-Adj.NT liver, we tested whether immature neutrophils directly promote HCC development by treating LFD and GAN mice from 48 weeks with six fortnightly doses of G-CSF/anti-G-CSF complexes (G-CSFcx) to induce immature neutrophil expansion and mobilization[25] (Fig. S5H). Despite significant accumulation of immature neutrophils (Cd101$^-$Ly6G$^+$Cd11b$^+$) in peripheral blood and liver (Fig. S5I and J), G-CSFcx treatment did not alter tumor incidence or burden (Fig. S5L,M), indicating that immature neutrophil accumulation alone is insufficient to promote HCC. Nevertheless, the enrichment of immature neutrophils and neutrophil-like monocytes in HCC-transitioned liver strongly reflects heightened emergency granulopoiesis and monopoiesis associated with the hyperinflammatory state of HCC progressors.

Enhanced stress-induced activation of cDC1 and cDC2 in HCC progressors

Subclustering identified nine dendritic cell populations, comprising five cDC1 (D1, D4–D7; *Xcr1, Batf3, Irf8*), one cDC2 (D0; *Cd209a, Clec10a*), one migratory DC3 (D2; *Ccr7, Mreg, Fscn1*) and two pDC (*Siglech*; D3, D8) subclusters (Fig. 5A,D). Notably, cDC1 composition shifted from D7 enrichment in GAN liver to D4 enrichment in GAN-Adj.NT liver (Fig. 5B,C). Compared with D7, D4 cDC1s exhibited 195 upregulated DEGs, including immediate-early (*Fosb, Junb, Egr1*) and heat-

immunohistochemistry of a liver section from an HCC-bearing mouse fed the GAN diet for 60 weeks. Leftmost panel: the tumor region demarcated in white; scale bar represents 200 μm. Albumin and DAPI signals were removed from the leftmost panel for enhanced clarity. Enlarged regions of interest 1, 2 and 3 are shown in the panels to the right; scale bars represent 25 μm. (H) Multiplex immunohistochemistry of a liver section from a non-tumor-bearing mouse fed the GAN diet for 60 weeks. Leftmost panel's scale bar represents 200 μm. Albumin and DAPI signals were removed from the left-most panel for enhanced clarity. Enlarged regions of interest 1 and 2 are shown in the panels to the right; scale bars represent 25 μm. Adj.NT, adjacent non-tumor; DEG, differentially expressed gene; GAN, Gubra-Amylin-NASH; HCC, hepatocellular carcinoma; LAM, lipid-associated macrophage; UMAP, uniform manifold approximation and projection; NTB, non-tumor-bearing.

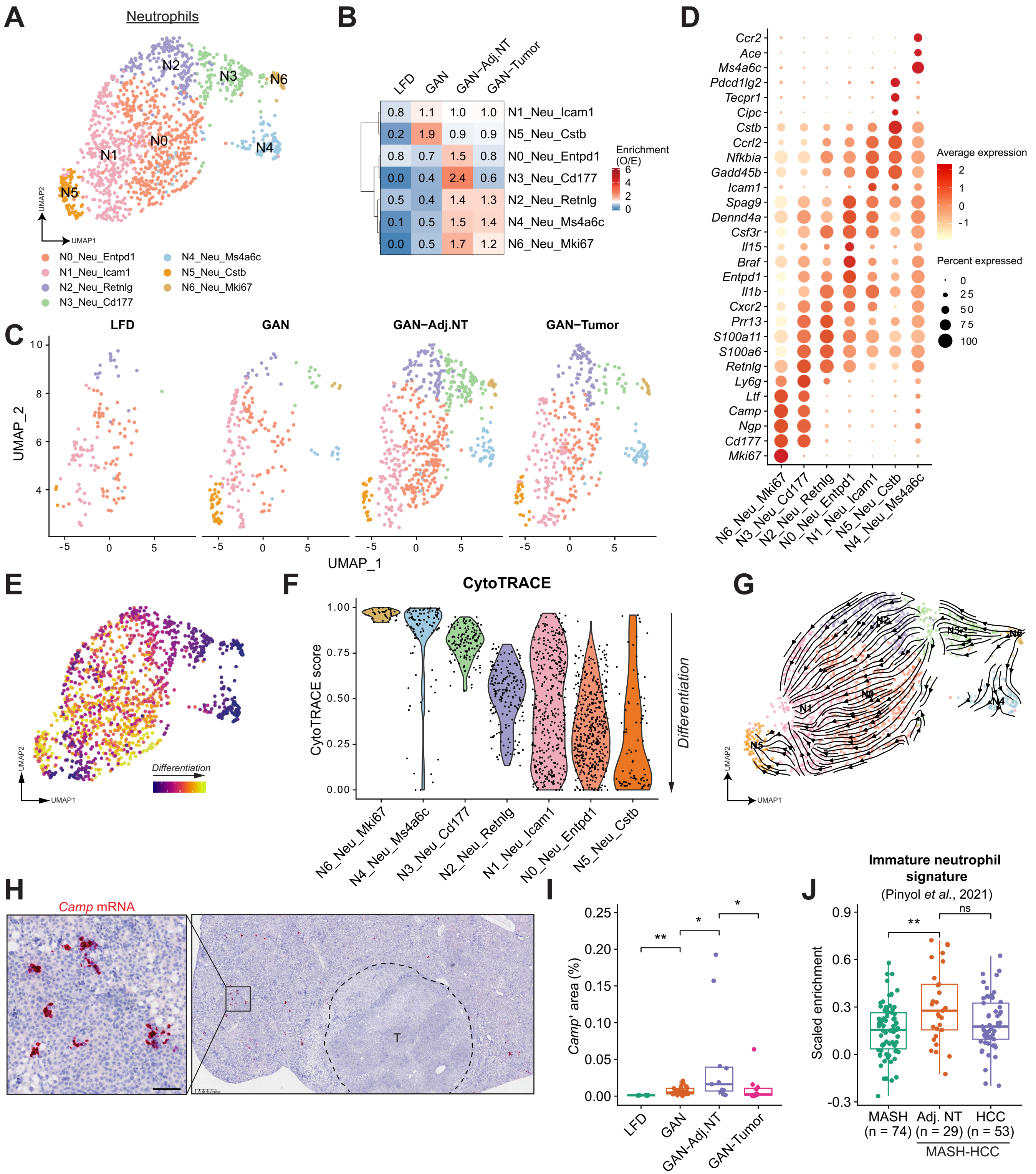

	LFD	GAN	GAN-Adj.NT	GAN-Tumor
N1_Neu_Icam1	0.8	1.1	1.0	1.0
N5_Neu_Cstb	0.2	1.9	0.9	0.9
N0_Neu_Entpd1	0.8	0.7	1.5	0.8
N3_Neu_Cd177	0.0	0.4	2.4	0.6
N2_Neu_Retnlg	0.5	0.4	1.4	1.3
N4_Neu_Ms4a6c	0.1	0.5	1.5	1.4
N6_Neu_Mki67	0.0	0.5	1.7	1.2

Fig. 4. Enrichment of immature neutrophils in adjacent non-tumor regions of HCC-transitioned livers. (A) UMAP of neutrophils showing 7 subclusters. (B) Enrichment scores of neutrophil subclusters. (C) UMAP of neutrophils separated by sample group. (D) Dot plot of marker genes for neutrophils. (E) UMAP showing CytoTRACE scores of neutrophils used to predict degree of differentiation. (F) Violin plot of CytoTRACE scores across neutrophil subclusters. (G) CellRank2 trajectory analysis integrating RNA velocity and gene similarity of neutrophils. (H) Representative image of an HCC-bearing liver section stained for *Camp* mRNA by RNAscope. Scale bars of left and right panels represent 100 µm and 300 µm, respectively. (I) Quantification of percentage of *Camp*$^{+}$ area in liver sections. Wilcoxon signed-rank test. (J) Gene set enrichment scores for the 5-gene immature neutrophil signature in a human MASH-HCC cohort. Wilcoxon signed-rank test. HCC, hepatocellular carcinoma; MASH, metabolic dysfunction-associated steatohepatitis; UMAP, uniform manifold approximation and projection.

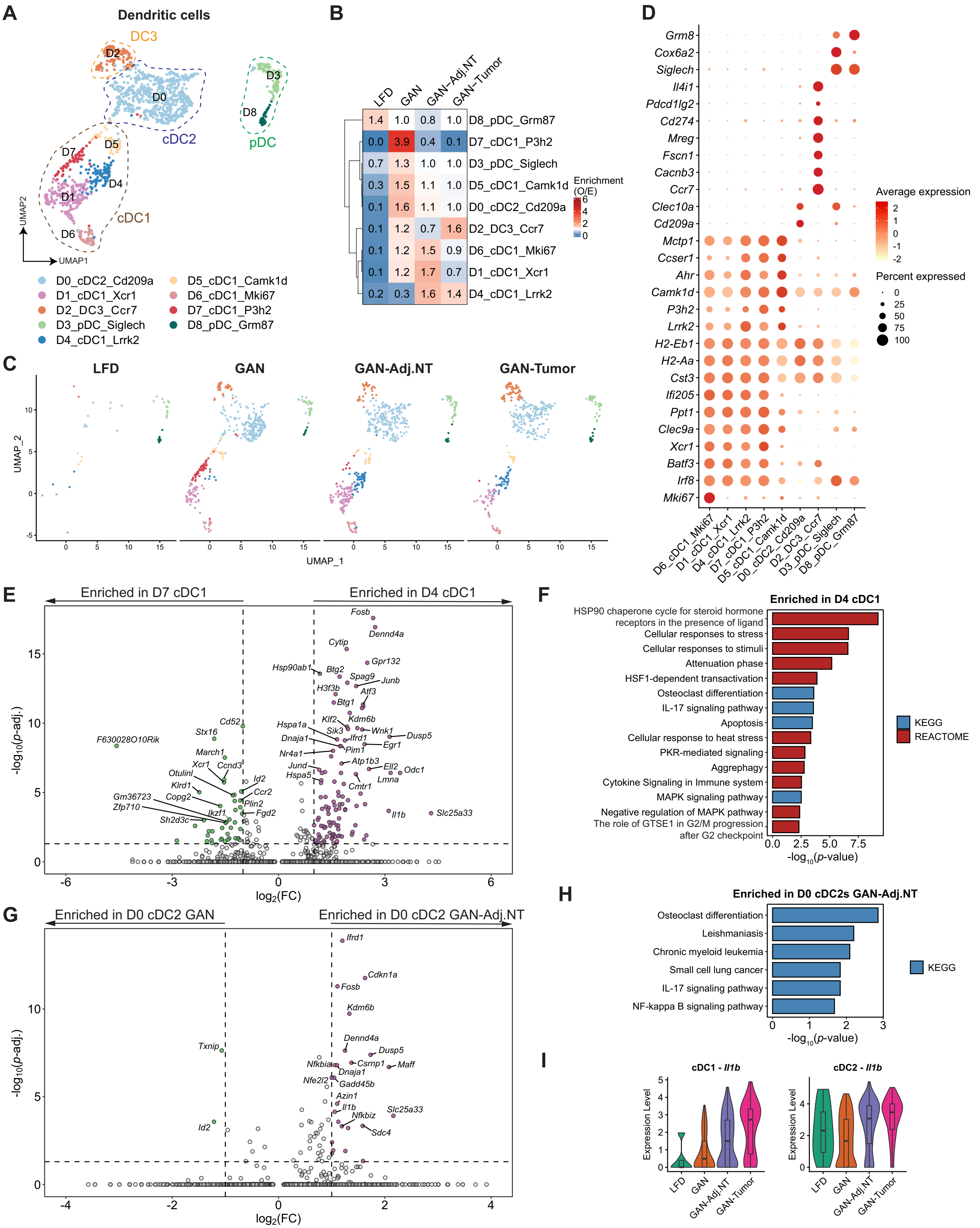

Fig. 5. Enhanced stress-induced activation of cDC1 and cDC2 in HCC progressors. (A) UMAP of dendritic cells showing 9 subclusters, comprising cDC1, cDC2, DC3 and pDC subsets. (B) Enrichment scores of dendritic cell subclusters. (C) UMAP of dendritic cells separated by sample group. (D) Dot plot of marker genes for dendritic cells. (E) Differential expression analysis of D4 cDC1s (enriched in GAN-Adj.NT tissues) *vs.* D7 cDC1s (enriched in GAN tissues). BH-adjusted *p* values. (F) KEGG and Reactome gene set enrichment of DEGs enriched in D4 cDC1s. Top 15 gene sets are shown. *p* values calculated using the g:SCS algorithm of gProfiler2.

shock (*Hsp90ab1, Hspa1a*) genes, alongside reduced *Xcr1* expression (Fig. 5D,E; Table S4). Pathway analysis further revealed enrichment of stress-response and immune-activation programs, including IL-17 signaling (Fig. 5F), consistent with a more activated, stress-responsive cDC1 state in GAN-Adj.NT liver.

Trajectory analysis indicated that D4 and D7 cDC1s arise from D1 and converge on D5 (Fig. S6A). D5 displayed greater differentiation by CytoTRACE and reduced *Xcr1*, *Batf3* and MHC class II expression, consistent with a terminally differentiated, post-stimulated state (Figs 5D and S6B,C). Conversely, D1 was metabolically active and enriched for antigen processing and presentation pathways, consistent with a pre-stimulated state (Fig. S6D). Thus, GAN-Adj.NT cDC1s follow a distinct trajectory through an activated, pro-inflammatory D4 intermediate before converging on the shared D5 terminal state.

Differential expression analysis of cDC2s (D0) similarly revealed increased activation and stress responses in GAN-Adj.NT compared with GAN cells, including upregulation of immediate-early (*Fosb, Gadd45b, Dnaja1*), NFκB-associated (*Il1b, Nfkbia*) and oxidative stress-response (*Nfe2l2, Maff*) genes (Fig. 5G,H, Table S5). Notably, *Il1b* expression was elevated in both cDC1 and cDC2 populations from GAN-Adj.NT and GAN-Tumor tissues compared with GAN liver (Fig. 5I). Together, these findings reveal activated, pro-inflammatory dendritic cell states associated with HCC progression.

Increased Th17/Treg ratio and dysfunctional T cells in livers of HCC progressors

Subclustering of T cells and ILC1s identified 10 T-cell populations (T0–T4, T6–T10) and one ILC1 cluster (T5; *Ncr1, Klrb1b, Eomes*^{low}) (Fig. 6A,D). T-cell populations included four CD8+ cytotoxic T lymphocyte (CTL) clusters (T0, T2, T8, T10; *Cd8b1, Ccl5, Nkg7*), T regulatory cells (Treg) (T4; *Foxp3*), T helper 17 (Th17) cells (T3; *Il17a, Rorc*) and naïve T cells (T6; *Tcf7, Lef1, Sell*) (Fig. 6A,D).

Tregs were enriched in HCC-naïve GAN livers but markedly reduced in GAN-Adj.NT and GAN-Tumor tissues, accompanied by progressive Th17 enrichment (Fig. 6B,C). This shift towards an increased Th17/Treg balance is characteristic of pro-inflammatory immune states and further supports a hyperinflammatory microenvironment associated with HCC progression.

Three T-cell subclusters (T1, T7, and T9) displayed reduced expression of T-cell receptor signaling genes (*Trac, Trbc1, Trbc2, Cd3d, Cd3e, Lat*) (Fig. 6E) and enrichment of anergy-associated signatures, consistent with hyporesponsive states (Fig. 6E,F). T7 and T9 were enriched in GAN-Adj.NT compared to GAN livers, while T1, which expressed the exhaustion-associated gene *Tox*,[26] was similarly abundant across both groups (Fig. 6B). CytoTRACE and CellRank2 further indicated that these were highly differentiated states, with T7 and T9 arising from Th17 (T3) and CTL (T10) populations, respectively (Fig. S7A–C). Together, these findings suggest accumulation of differentiated, dysfunctional T-cell states associated with chronic stimulation,[27] further supporting a hyperinflammatory yet functionally dysregulated immune landscape in HCC progressors.

Loss of dark-zone germinal center B cells in HCC progressors and atypical B cells in MASH

B cells were re-clustered into six subclusters comprising three germinal center B-cell (Bgc) populations (B0, B1, B4; *Bcl6, Myc*), two atypical memory B-cell populations (B2, B3) and one immature B-cell population (B5; *Vpreb3, Sox4, Cd83*^{low}) (Fig. 6G,J). B4 Bgc were enriched in GAN livers, whereas B1 Bgc predominated in GAN-Adj.NT and GAN-Tumor tissues, indicating divergent Bgc phenotypes between HCC-naïve and HCC-progressed liver (Fig. 6H,I). Gene signature analysis further resolved distinct Bgc states (Fig. 6K, Table S6). B0 resembled antigen-experienced pre-germinal center B cells, whereas B1 exhibited light-zone germinal center features, including *Cxcr5*, *Cd69* and *Cd86* expression and enrichment of TNFα/NFκB and cytokine production pathways (Fig. 6J–K).[28,29]

In contrast, B4 showed dark-zone germinal center characteristics, with enriched B-cell activation, B-cell receptor signaling and proliferation programs, high *Ccnd3* and *Irf4* expression, and reduced MYC target activity, consistent with late-stage dark-zone Bgc transitioning towards plasma cell differentiation.[30,31] The depletion of B4 dark-zone Bgc in GAN-Adj.NT therefore suggests impaired germinal center function and a compromised humoral response during HCC progression.

Interestingly, two atypical B-cell subclusters (B2 and B3) were similarly enriched in GAN and GAN-Adj.NT livers, suggesting their emergence during MASH progression (Fig. 6H–J). B3 expressed *Zeb2, Cd19, Cd86, Fcrl5* and *Itgb1*, while lacking *Ighd, Fcer2a, Cr2* and *Bach2*, resembling age-associated B cells (ABCs) described in autoimmune and infectious diseases.[32–34] Consistently, B3 was enriched for multiple ABC signatures (Fig. 6K) and expressed *Mzb1* (Fig. 6J). B3 also showed enrichment of mTORC1 signaling, fatty acid oxidation, antigen processing/presentation and type II interferon-response programs (Fig. 6K). As ABCs are autoreactive memory B cells with the capacity to generate autoantibody-producing cells,[35] their identification here suggests a previously unrecognized B-cell state in MASH.

In contrast, B2 displayed memory B-cell features, including increased *Bcl2* and reduced MHC class II genes (*H2-Aa, H2-Ab1*), but lacked key B-cell receptor signaling components (*Cd19, Cd79a, Cd79b*), consistent with an anergic phenotype (Fig. 6J).[36] B2 cells were also terminally differentiated (Fig. S7D–F), metabolically less active and enriched for type I interferon-response programs, resembling atypical memory B

(G) Differential expression analysis of D0 cDC2 cells in GAN-Adj.NT tissues *vs*. GAN counterparts. BH-adjusted *p* values. (H) KEGG and Reactome gene set enrichment of DEGs enriched in GAN-Adj.NT D0 cDC2s. Only KEGG gene sets showed significant enrichment. *p* values calculated using the g:SCS algorithm of gProfiler2. (I) Expression of *Il1b* in cDC1 (D1, D4, D5, D6, D7) and cDC2 (D0) cells across sample groups. Adj.NT, adjacent non-tumor; cDC1, conventional dendritic cell type 1; cDC2, conventional dendritic cell type 2; DC3, dendritic cell 3; DEG, differentially expressed gene; GAN, Gubra-Amylin-NASH; HCC, hepatocellular carcinoma; pDC, plasmacytoid dendritic cell; UMAP, uniform manifold approximation and projection; BH, Benjamini-Hochberg.

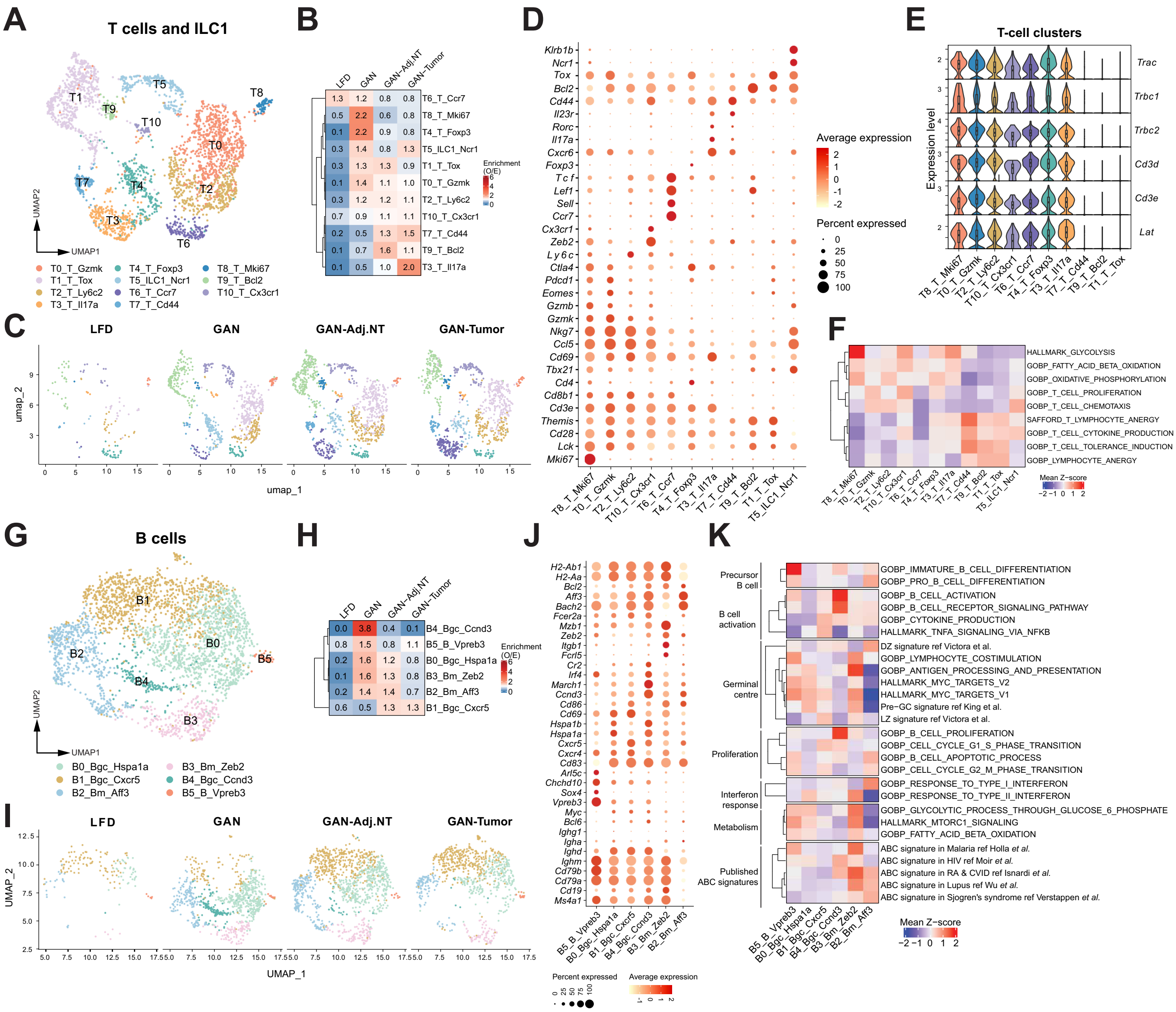

Fig. 6. Increased Th17/Treg ratio and loss of dark zone germinal center B cells in livers of HCC progressors. (A) UMAP of T cells and ILC1 revealing 11 subclusters comprising 10 T-cell and 1 ILC1 subclusters. (B) Enrichment scores of T-cell and ILC1 subclusters. (C) UMAP of T cells and ILC1 separated by sample group. (D) Dot plot of marker genes for T cells and ILC1. (E) Expression of genes involved in T-cell receptor signaling across T-cell subclusters. (F) Single-cell gene set enrichment Z-scores of gene sets across T-cell and ILC1 subclusters. (G) UMAP of B cells revealing 6 subclusters. (H) Enrichment scores of B-cell subclusters. (I) UMAP of B cells separated by sample group. (J) Dot plot of marker genes for B cells. (K) Single-cell gene set enrichment Z-scores of gene sets across B cell subclusters. HCC, hepatocellular carcinoma; ILC1, innate lymphoid cell type 1; Th17, T helper 17; Treg, T regulatory cell; UMAP, uniform manifold approximation and projection.

cells described in chronic viral infection.[37] Thus, distinct atypical memory B-cell states associated with autoimmune and chronic infectious diseases also emerge during MASH progression.

Augmented endothelial cell activation in livers of HCC progressors and fetal-like EC reprogramming in MASH-HCC tumors

Re-clustering of ECs identified ten subclusters with distinct tissue distributions (Fig. 7A–C). E0, E2 and E5 expressed canonical LSEC markers (*Clec4g, Hpgd, Stab1*) and were almost exclusively derived from LFD liver, whereas E3, characterized by lipid metabolism genes (*Fabp4, Fabp5, Cd36*), was restricted to GAN-associated tissues (Fig. 7B–D). Accordingly, GAN-associated ECs showed increased lipid metabolism, particularly lipid import and storage (Fig. 7E,F), together with enrichment of extracellular matrix organization and cell adhesion programs compared with LFD ECs (Fig. S8A and B, Table S7). Importantly, ECs from GAN-Adj.NT and GAN-Tumor tissues were enriched for pro-inflammatory programs, including IL-6, type I/II interferon and TNFα signaling (Fig. 7F). Consistently, GAN-Adj.NT ECs upregulated the alarmin genes *S100a8/S100a9* and

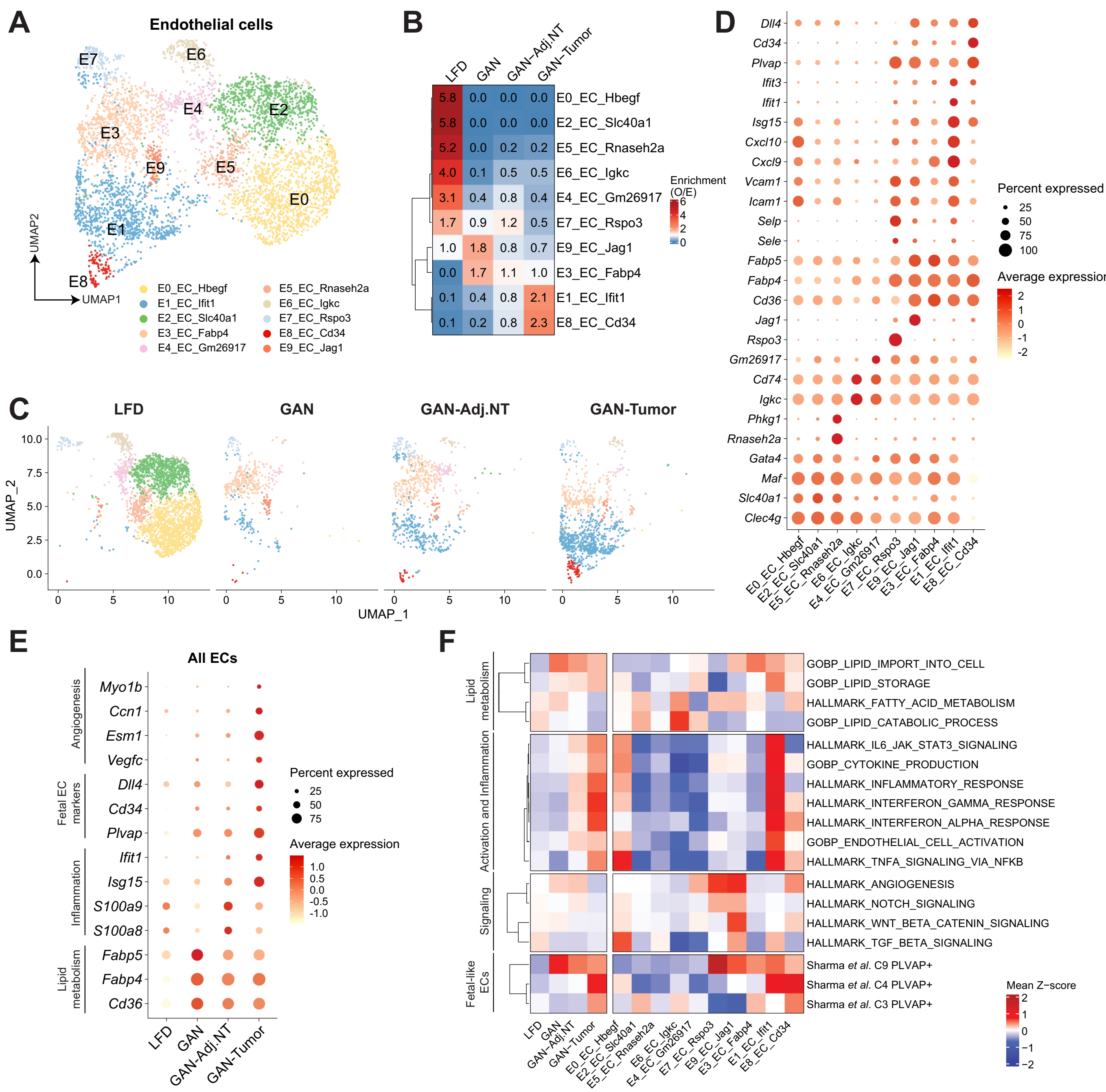

Fig. 7. Augmented endothelial cell activation in livers of HCC progressors and emergence of fetal-like EC subsets in HCCs. (A) UMAP of ECs revealing 10 subclusters. (B) Enrichment scores of EC subclusters. (C) UMAP of ECs separated by sample group. (D) Dot plot of marker genes for ECs. (E) Expression of chosen genes across all ECs within each sample group. (F) Single-cell gene set enrichment Z-scores of gene sets across all ECs separated by sample group (heatmap, left) and across EC subclusters (heatmap, right). EC, endothelial cell; HCC, hepatocellular carcinoma; UMAP, uniform manifold approximation and projection.

interferon-response genes *Isg15/Ifit1* compared with GAN ECs (Figs 7E and S8C, Table S8), indicating heightened endothelial inflammatory activation in the cancer-primed microenvironment.

E1 and E8 ECs were strongly enriched in GAN-Tumor tissue and present at lower abundance in GAN-Adj.NT liver (Fig. 7B,C). E1 exhibited an activated, pro-inflammatory phenotype, with enrichment of interferon-response programs and expression of *Cxcl9, Cxcl10, Isg15, Ifit1* and *Ifit3* (Fig. 7D,F). E8 expressed the fetal LSEC-associated gene *Plvap* and *Cd34*, while showing reduced LSEC identity markers (*Clec4g, Maf, Gata4*), consistent with a fetal-like phenotype. Notably, PLVAP^{+} "oncofetal" ECs have been described in human HCC and implicated in DLL4/NOTCH2-mediated immunosuppression.[38] Indeed, E1 and E8 were enriched for published PLVAP^{+} oncofetal EC signatures,[38] supporting their oncofetal identities (Fig. 7F; Table S9). GAN-Tumor ECs also upregulated pro-angiogenic factors (*Vegfc, Esm1, Ccn1, Myo1b*) compared with GAN-Adj.NT ECs (Fig. 7E), several of which were enriched in E1/E8 (Fig. S8D).

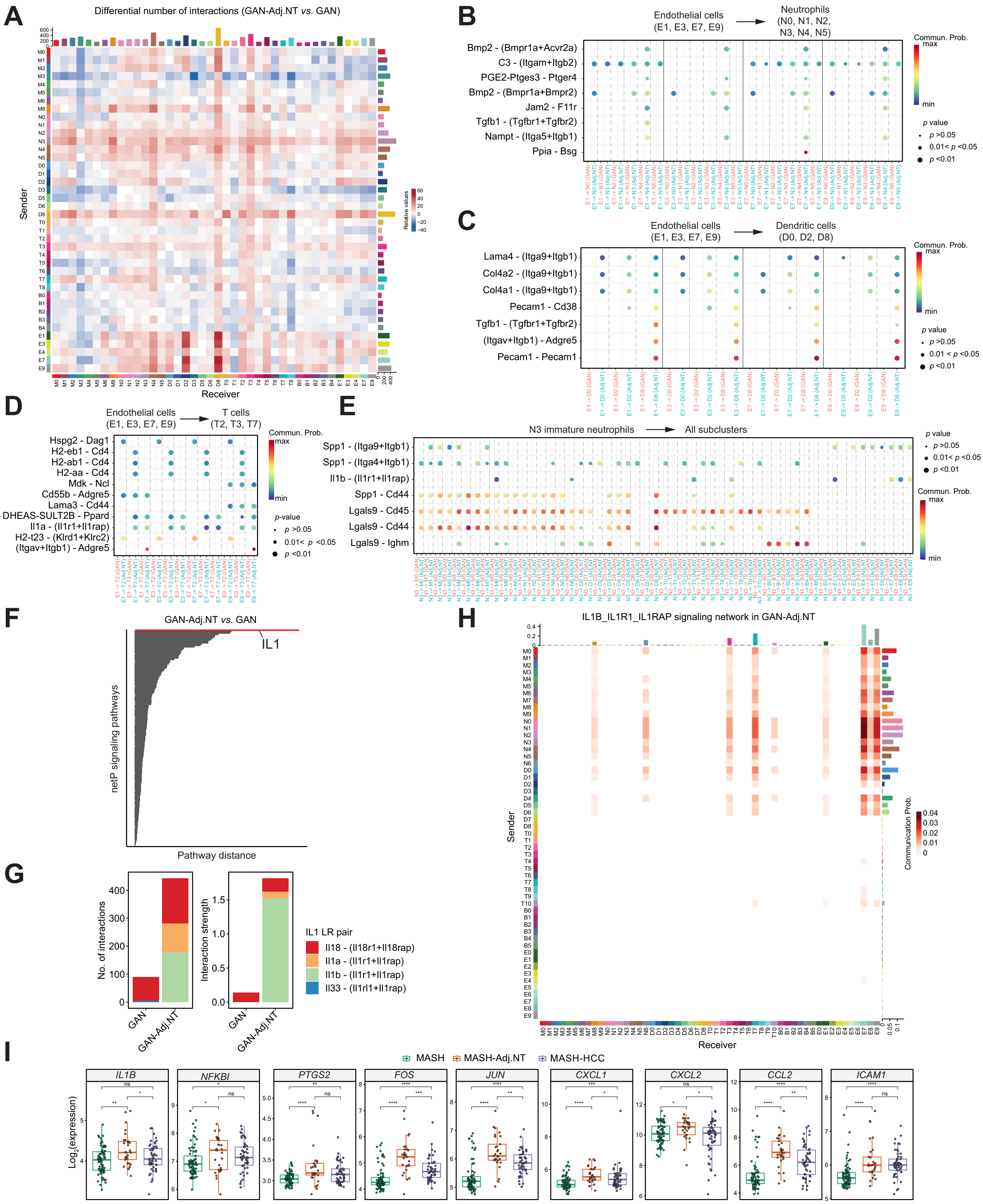

Fig. 8. Endothelial-immune cell-cell communication and a myeloid-driven IL-1β signaling network underlie the hyperinflammatory phenotype in HCC progressors. (A) Differential number of interactions between subclusters in the GAN-Adj.NT and GAN cell-cell communication networks. Red indicates an increase in the number of interactions in GAN-Adj.NT, while blue indicates an increase in the GAN network. (B) Top ligand–receptor interactions between endothelial cells and neutrophils uniquely identified in GAN-Adj.NT but not in the overall GAN network. (C) Top ligand–receptor interactions between endothelial cells and dendritic cells

Altogether, ECs undergo metabolic and extracellular matrix remodeling in MASH, acquire heightened inflammatory states in HCC progressors, and develop tumor-enriched oncofetal phenotypes resembling those observed in human HCC.

Endothelial-immune cell-cell communication and a myeloid-driven IL-1β signaling network underlie the hyperinflammatory phenotype in HCC progressors

Our analyses revealed 53 distinct immune and non-parenchymal subclusters with differential enrichment across MASH-to-HCC progression (Fig. S9A). To identify signaling pathways underlying the hyperinflammatory phenotype of HCC progressors, we used CellChat[39] to infer ligand–receptor (LR) communication between these subclusters, focusing on GAN *vs*. GAN-Adj.NT tissues.

After excluding subclusters with <10 cells in either group, GAN-Adj.NT tissue showed a marked increase in predicted LR interactions, particularly from EC subclusters (E1, E3, E4, E7, E9) towards neutrophils (N0–N5), dendritic cells (D0, D2, D8) and T cells (T2, T3, T7) (Fig. 8A). These senders included pro-inflammatory E1, peri-central E7 (*Rspo3*) and peri-portal E9 (*Jag1*) ECs, suggesting widespread endothelial–immune crosstalk across vascular niches in GAN-Adj.NT tissue.

Among EC–neutrophil interactions uniquely enriched in GAN-Adj.NT tissue, CellChat identified LR pairs involved in leukocyte adhesion and migration, including *C3–(Itgam+Itgb2)* and *Jam2–F11r*, consistent with enhanced neutrophil recruitment (Fig. 8B). Increased *Bmp2* signaling was also predicted from ECs to mature (N0), immature (N3) and monocyte-like (N4) neutrophils.

EC communication with cDC2s (D0), DC3s (D2) and pDCs (D8) was similarly increased in GAN-Adj.NT tissue (Fig. 8C), predominantly through ECM–integrin interactions, including collagen IV and laminin (*Col4a1, Col4a2, Lama4*) signaling to α9β1 integrins (*Itga9, Itgb1*). These interactions suggest enhanced endothelial support for dendritic cell recruitment and retention, potentially amplifying local immune responses.

EC communication with *Ly6c2*$^+$ CTLs (T2), Th17 cells (T3) and dysfunctional Th17-derived T cells (T7) was also increased in GAN-Adj.NT tissue (Fig. 8D). Predicted MHC class II–*Cd4* and *Il1a–(Il1r1+Il1rap)* interactions with Th17 cells suggest enhanced antigen presentation and IL-1-driven Th17 activation,[40] while ECM interactions (*Lama3, Hspg2*) with Ly6c2$^+$ CTLs may promote their recruitment and retention. These findings support enhanced endothelial–T-cell crosstalk within the inflammatory GAN-Adj.NT niche.

N3 immature neutrophils, which were strongly enriched in GAN-Adj.NT livers, also showed increased predicted communication with multiple immune populations. *Lgals9* and *Spp1* emerged as dominant N3-derived signals, particularly through *Lgals9–Cd44* and *Spp1–Cd44/integrin* interactions associated with immune-cell recruitment, adhesion and retention (Fig. 8E). These findings suggest that N3 neutrophils may contribute to an inflammatory, pro-tumorigenic niche despite being insufficient to independently promote HCC. Collectively, CellChat analysis revealed extensive rewiring of endothelial–immune and neutrophil–immune communication in GAN-Adj.NT livers, consistent with enhanced inflammatory cell recruitment, retention and intercellular crosstalk.

To unbiasedly identify signaling pathways most altered in GAN-Adj.NT tissue, we performed joint manifold learning of CellChat communication networks based on functional similarity across conditions. IL-1 signaling emerged as the most prominently altered pathway between GAN and GAN-Adj.NT tissues (Fig. 8F). Within this pathway, *Il1b–(Il1r1+Il1rap)* showed the greatest increase in both interaction number and strength in GAN-Adj.NT livers, while no significant interactions were detected in GAN livers (Fig. 8G). Consistently, pro-IL-1β protein was increased in Adj.NT liver from HB mice compared with NTB GAN mice, supporting increased IL-1β production (Fig. S9B).

Analysis of the *Il1b–(Il1r1+Il1rap)* network identified myeloid cells, including monocytes/macrophages, cDC1/2s and neutrophils, as the major IL-1β senders in GAN-Adj.NT livers (Fig. 8H). Dominant receivers included pro-inflammatory (E1), fetal-like (E8), peri-central (E7) and peri-portal (E9) ECs, as well as Th17 cells (T3), proliferating monocytes (M8) and immature proliferating neutrophils (N6) (Fig. 8H). These findings identify myeloid-derived IL-1β as a prominent signaling axis in HCC-transitioned MASH liver, potentially reinforcing endothelial activation, leukocyte recruitment and Th17 inflammation in a feed-forward hyper-inflammatory microenvironment.

IL-1β-associated hyperinflammatory reprogramming is recapitulated in human MASH-HCC

To assess the relevance of our findings to human disease, we analyzed liver transcriptomic data from non-cirrhotic patients with MASH and MASH-HCC.[23] Indeed, *IL1B* expression was significantly elevated in MASH-HCC adjacent non-tumor tissue, together with canonical IL-1β-responsive genes (*NFKBIA, PTGS2, FOS, JUN, CXCL1, CXCL2, CCL2, ICAM1*), consistent with activation of NFκB and immediate-early inflammatory programs (Fig. 8I). Single-sample GSEA further revealed enrichment of “IL-1β production”, “Response to IL-1”, “Inflammatory Response”, “TNFα signaling via NFκB” and “IL-6/JAK/STAT3 signaling” pathways in adjacent non-tumor compared with MASH-only liver (Fig. S9C). Together, these findings support the presence of a heightened IL-1β-associated inflammatory program in human non-cirrhotic MASH-HCC adjacent non-tumor liver, mirroring our observations in mice.

uniquely identified in GAN-Adj.NT but not in the overall GAN network. (D) Top ligand–receptor interactions between endothelial cells and T cells uniquely identified in GAN-Adj.NT but not in the overall GAN network. (E) Top ligand–receptor interactions between N3 immature neutrophils and all subclusters uniquely identified in GAN-Adj.NT but not in the overall GAN network. (F) Joint manifold learning and classification analysis of the inferred communication networks based on functional similarity by CellChat comparing signaling networks between GAN-Adj.NT and GAN subclusters. (G) Number of interactions (left) and interaction strengths (right) of IL-1 LR pairs among GAN-Adj.NT and GAN subclusters. (H) Communication probabilities of Il1b-(Il1r1+Il1rap) signaling between senders (vertical axis) and receivers (horizontal axis) in the GAN-Adj.NT network, as predicted by CellChat. (I) Log_2-normalised expression of indicated genes in liver tissues from a human cohort of non-cirrhotic MASH and MASH-HCC patients. Wilcoxon signed-rank test. Adj.NT, adjacent non-tumor; GAN, Gubra-Amylin-NASH; HCC, hepatocellular carcinoma; LR, ligand-receptor; MASH, metabolic dysfunction-associated steatohepatitis.

Discussion

In this study, we present a high-resolution, cross-sectional single-cell atlas of immune and stromal remodeling across HCC-naïve and HCC-progressed MASH liver. Our analyses reveal coordinated changes across multiple cellular compartments that converge on a hyperinflammatory microenvironment in the adjacent non-tumor liver of HCC progressors. These included depletion of *Trem2*$^{+}$ LAMs with expansion of *Trem2*^{low} pro-inflammatory macrophages, accumulation of immature neutrophils, stress-responsive activation of cDC1 and cDC2 populations, increased Th17/Treg balance, loss of dark-zone germinal center B cells, and inflammatory endothelial activation. Cell–cell communication analysis further identified myeloid-derived IL-1β signaling as a prominent axis potentially linking these cellular changes. Elevated circulating pro-inflammatory cytokines further indicated that this hyperinflammatory phenotype extends beyond the liver.

Importantly, human non-cirrhotic MASH-HCC adjacent non-tumor tissues similarly exhibited an immature neutrophil signature and heightened *IL1B*-associated inflammatory programs, supporting the clinical relevance of this inflammatory state. Collectively, our findings suggest that non-cirrhotic HCC progression in MASH is associated with coordinated hyperinflammatory reprogramming.

A highlight of this reprogramming was the shift in macrophage states from *Trem2*$^{+}$ LAMs towards *Trem2*^{low} pro-inflammatory populations. *TREM2*$^{+}$ macrophages are increasingly recognized for their reparative functions in MASH, including clearance of dying, lipid-laden hepatocytes.[12,13] Their depletion could therefore compromise tissue repair and permit inflammatory signals to persist. Consistent with this possibility, *Trem2* has been shown to suppress tumorigenesis in chemical models of HCC,[41] while *Trem2* deficiency enhances macrophage *Il1b* expression and inflammatory signaling in pancreatic cancer.[42] Our trajectory analyses further suggest that *Trem2*$^{+}$ LAMs may transition towards a stress-responsive, IL-1β-expressing macrophage state within tumors. Together, these observations raise the possibility that disruption of TREM2-associated reparative macrophage programs contributes to escalating myeloid inflammation during MASH-to-HCC progression.

Myeloid-derived IL-1β may provide an organizing signal connecting this macrophage reprogramming to the broader cellular changes observed in HCC progressors. IL-1β cooperates with IL-6 and IL-23 to promote Th17 differentiation,[40] and with G-CSF, IL-6 and IL-17A to drive emergency granulopoiesis and mobilization of immature neutrophils.[11] It also potently activates endothelial cells and promotes leukocyte recruitment.[43] Accordingly, the increased Th17/Treg balance, accumulation of immature neutrophils and endothelial activation observed in adjacent non-tumor liver could represent interconnected manifestations of an IL-1β-associated inflammatory circuit rather than independent phenomena.

IL-17A itself can promote HCC development in experimental MASH,[44] while IL-1β and IL-6/STAT3 signaling have established pro-tumorigenic functions,[45,46] providing potential mechanisms through which sustained inflammation could establish a tumor-permissive environment.

Endothelial cells may be particularly important amplifiers of this circuit. MASH was associated with loss of canonical LSEC states and acquisition of lipid metabolic and extracellular matrix remodeling programs, followed by inflammatory endothelial activation in HCC progressors. CellChat predicted extensive communication between ECs and neutrophils, dendritic cells and T cells, with myeloid-derived IL-1β representing a major input to multiple endothelial populations. Activated ECs could therefore translate myeloid inflammatory signals into enhanced leukocyte recruitment and retention, potentially establishing a feed-forward circuit that sustains inflammation within the cancer-primed liver. The emergence of oncofetal EC states within tumors further indicates progressive endothelial reprogramming as MASH transitions to established HCC.

Our lymphocyte analyses provide additional evidence of chronic immune dysregulation. Alongside Th17 skewing, we identified dysfunctional, anergic-like T-cell and atypical B-cell states resembling populations described during chronic infection and autoimmunity.[32] Previous studies have implicated autoreactive T cells in MASH-associated hepatocarcinogenesis,[3,7] suggesting that persistent antigenic stimulation may contribute to this immune landscape. Whether the dysfunctional lymphocyte populations identified here actively promote HCC progression or instead reflect chronic inflammatory exposure remains unclear.

These findings lead us to propose a model in which loss or reprogramming of reparative Trem2^{+} macrophages permits increased myeloid IL-1β signaling, which in turn reinforces endothelial activation, emergency granulopoiesis and Th17 inflammation. These interacting processes could establish a self-sustaining hyperinflammatory field permissive for malignant transformation. Importantly, however, the cross-sectional design of our study cannot establish the temporal sequence or causality of these events, and the inflammatory state could partly arise as a consequence of established tumorigenesis. Prospective studies tracking these cellular states before tumor development, together with targeted perturbation of TREM2 and IL-1β signaling, will be required to test this model.

The hyperinflammatory and autoimmune-like features of HCC-progressed MASH raise the possibility that immunosuppressive therapies could prevent HCC development. However, glucocorticoids induce hyperglycemia, hyperinsulinemia and insulin resistance, potentially worsening metabolic dysfunction,[47] while IL-1β blockade failed to ameliorate disease in a preclinical MASH model.[48] Similarly, CCR2/CCR5 inhibition targeting inflammatory monocyte/macrophage recruitment failed in phase III MASH trials.[49] These outcomes highlight the complexity and redundancy of inflammatory networks in MASH and caution against targeting individual inflammatory pathways in isolation.

Affiliations
[1]*Institute of Molecular and Cell Biology (IMCB), Agency for Science, Technology and Research (A*STAR), 61 Biopolis Drive, Proteos, 138673, Singapore;* [2]*Singapore Immunology Network, Agency for Science, Technology and Research (A*STAR), 8A Biomedical Grove, Immunos, 138648, Singapore*

Abbreviations

ABC, age-associated B cell; Adj.NT, adjacent non-tumor; AFP, alpha-fetoprotein; ALT, alanine transaminase; Bgc, germinal center B cell; CTL, cytotoxic T lymphocyte; DC3, dendritic cell 3; DEG, differentially expressed gene; EC, endothelial cell; FFPE, formalin-fixed paraffin-embedded; GAN, Gubra-Amylin-NASH; HB, HCC-bearing; HCA, hepatocellular adenoma; HCCA, hepatocholangiocellular adenoma; HCC, hepatocellular carcinoma; ILC1, innate lymphoid cell type 1; KC, Kupffer cell; LAM, lipid-associated macrophage; LFD, low-fat diet; LR, ligand-receptor; LSEC, liver sinusoidal endothelial cell; MASH, metabolic dysfunction-associated steatohepatitis; MASLD, metabolic dysfunction-associated steatotic liver disease; MHC, major histocompatibility complex; NTB, non-tumor-bearing; TBA, total bile acids; TCR, T-cell receptor; Th17, T helper 17; Treg, T regulatory cell; UMAP, uniform manifold approximation and projection.

Financial support

This work was funded by Competitive Research Program grant (NRF-CRP23-2019-0004) from the National Research Foundation, Singapore.

Conflict of interest

The authors declare that they have no conflict of interest.

Please refer to the accompanying ICMJE disclosure forms for further details.

Authors' contributions

Shawn Lu Wen Tan: Conceptualization, Data Curation, Formal analysis, Investigation, Methodology, Visualization, Writing – original draft, Writing – review & editing. Erez Israeli: Formal analysis, Writing – review & editing. Jillian Wee: Investigation. Hui Min Tan: Investigation. Ravisankar Rajarethinam: Formal analysis. Immanuel Kwok: Methodology, Writing – review & editing. Shu Zhen Chong: Methodology, Writing – review & editing. Weiping Han: Supervision, Funding acquisition, Conceptualization, Writing – review & editing.

Data availability

All bulk RNA-sequencing and single-cell transcriptomic data have been deposited in the GEO Database under accession numbers GSE308029 and GSE308046, respectively. Additional data supporting the findings of this study are available from the corresponding author upon reasonable request.

Declaration of generative AI and AI-assisted technologies in the writing process

During the preparation of this work the authors used ChatGPT (OpenAI) in order to improve language and readability. After using this tool/service, the authors reviewed and edited the content as needed and take full responsibility for the content of the manuscript.

Acknowledgements

We thank the Advanced Molecular Pathology Laboratory (AMPL) of A*STAR for assistance in preparation of paraffin-embedded tissue sections and immunohistochemistry. We thank Alicia Tay and Shanshan Wu Howland from the Singapore Immunology Network Immunogenomics platform of A*STAR for assistance in single-cell RNA sequencing. We thank Leonard Tan from A*STAR Spatial Biology & Immune Imaging Centre of Excellence for assistance in multiplex immunohistochemistry.

Supplementary data

Supplementary data to this article can be found online at https://doi.org/10.1016/j.jhepr.2026.102055.

References

Author names in bold designate shared co-first authorship

[1] Tan DJH, Ng CH, Lin SY, et al. Clinical characteristics, surveillance, treatment allocation, and outcomes of non-alcoholic fatty liver disease-related hepatocellular carcinoma: a systematic review and meta-analysis. Lancet Oncol 2022;23:521–530. https://doi.org/10.1016/S1470-2045(22)00078-X.

[2] Huang DQ, El-Serag HB, Loomba R. Global epidemiology of NAFLD-related HCC: trends, predictions, risk factors and prevention. Nat Rev Gastroenterol Hepatol 2021;18:223–238. https://doi.org/10.1038/s41575-020-00381-6.

[3] Pfister D, Núñez NG, Pinyol R, et al. NASH limits anti-tumour surveillance in immunotherapy-treated HCC. Nature 2021;592(7854):450–456. https://doi.org/10.1038/s41586-021-03362-0.

[4] **Anstee QM**, **Reeves HL**, **Kotsiliti E**, et al. From NASH to HCC: current concepts and future challenges. Nat Rev Gastroenterol Hepatol 2019;16:411–428. https://doi.org/10.1038/s41575-019-0145-7.

[5] Leslie J, Mackey JBG, Jamieson T, et al. CXCR2 inhibition enables NASH-HCC immunotherapy. Gut 2022;71:2093–2106. https://doi.org/10.1136/gutjnl-2021-326259.

[6] Shalapour S, Lin XJ, Bastian IN, et al. Inflammation-induced IgA+ cells dismantle anti-liver cancer immunity. Nature 2017;551:340–345. https://doi.org/10.1038/nature24302.

[7] **Dudek M**, **Pfister D**, **Donakonda S**, et al. Auto-aggressive CXCR6+ CD8 T cells cause liver immune pathology in NASH. Nature 2021;592:444–449. https://doi.org/10.1038/s41586-021-03233-8.

[8] Hansen HH, Ægidius HM, Oró D, et al. Human translatability of the GAN diet-induced obese mouse model of non-alcoholic steatohepatitis. BMC Gastroenterol 2020;20. https://doi.org/10.1186/s12876-020-01356-2.

[9] Liang W, Menke AL, Driessen A, et al. Establishment of a general NAFLD scoring system for rodent models and comparison to human liver pathology. PLoS One 2014;9:1–17. https://doi.org/10.1371/journal.pone.0115922.

[10] Curtius K, Wright NA, Graham TA. An evolutionary perspective on field cancerization. Nat Rev Cancer 2018;18(1):19–32. https://doi.org/10.1038/nrc.2017.102.

[11] Manz MG, Boettcher S. Emergency granulopoiesis. Nat Rev Immunol 2014;14(5):302–314. https://doi.org/10.1038/nri3660.

[12] Jaitin DA, Adlung L, Thaiss CA, et al. Lipid-associated macrophages control metabolic homeostasis in a Trem2-dependent Manner. Cell 2019;178:686–698.e14. https://doi.org/10.1016/j.cell.2019.05.054.

[13] Wang X, He Q, Zhou C, et al. Prolonged hypernutrition impairs TREM2-dependent efferocytosis to license chronic liver inflammation and NASH development. Immunity 2023;56:58–77.e11. https://doi.org/10.1016/j.immuni.2022.11.013.

[14] Remmerie A, Martens L, Thoné T, et al. Osteopontin expression Identifies a subset of recruited macrophages distinct from Kupffer cells in the fatty liver. Immunity 2020;53:641–657.e14. https://doi.org/10.1016/j.immuni.2020.08.004.

[15] Angerer P, Haghverdi L, Büttner M, et al. Destiny: diffusion maps for large-scale single-cell data in R. Bioinformatics 2016;32:1241–1243. https://doi.org/10.1093/bioinformatics/btv715.

[16] Pan F, Pan R, Hu R, et al. Analysis of the effects of M2 macrophage-derived PDE4C on the prognosis, metastasis and immunotherapy benefit of osteosarcoma. J Cel Mol Med 2024;28(10):e18395. https://doi.org/10.1111/jcmm.18395.

[17] Ni J, Li X, Wu Y, et al. CD81 Aggravates Ovarian cancer progression via p-Cresyl Sulfate-mediated Mitophagy in Tim4+ tumour-associated macrophages. J Cel Mol Med 2025;29(13):e70701. https://doi.org/10.1111/jcmm.70701.

[18] **Kim MS**, **Kang H**, Baek JH, et al. Disrupting Notch signaling related HES1 in myeloid cells reinvigorates antitumor T cell responses. Exp Hematol Oncol 2024;13. https://doi.org/10.1186/s40164-024-00588-2.

[19] **Goswami S**, **Raychaudhuri D**, **Singh P**, et al. Myeloid-specific KDM6B inhibition sensitizes glioblastoma to PD1 blockade. Nat Cancer 2023;4:1455–1473. https://doi.org/10.1038/s43018-023-00620-0.

[20] Lan Y, Han J, Wang Y, et al. STK17B promotes carcinogenesis and metastasis via AKT/GSK-3β/Snail signaling in hepatocellular carcinoma. Cell Death Dis 2018;9(2):236. https://doi.org/10.1038/s41419-018-0262-1.

[21] Grieshaber-Bouyer R, Radtke FA, Cunin P, et al. The neutrotime transcriptional signature defines a single continuum of neutrophils across biological compartments. Nat Commun 2021;12(1):2856. https://doi.org/10.1038/s41467-021-22973-9.

[22] **Ng MSF**, **Kwok I**, **Tan L**, et al. Deterministic reprogramming of neutrophils within tumors. Science 2024;383(6679):eadf6493. https://doi.org/10.1126/science.adf6493.

[23] Pinyol R, Torrecilla S, Wang H, et al. Molecular characterisation of hepatocellular carcinoma in patients with non-alcoholic steatohepatitis. J Hepatol 2021;75:865–878. https://doi.org/10.1016/j.jhep.2021.04.049.

[24] Ikeda N, Kubota H, Suzuki R, et al. The early neutrophil-committed progenitors aberrantly differentiate into immunoregulatory monocytes during emergency myelopoiesis. Cell Rep 2023;42(3):112165. https://doi.org/10.1016/j.celrep.2023.112165.

[25] Rubinstein MP, Salem ML, Doedens AL, et al. G-CSF/anti-G-CSF antibody complexes drive the potent recovery and expansion of CD11b+Gr-1+

myeloid cells without compromising CD8+ T cell immune responses. J Hematol Oncol 2013;6:75. https://doi.org/10.1186/1756-8722-6-75.
[26] Sekine T, Perez-Potti A, Nguyen S, et al. TOX is expressed by exhausted and polyfunctional human effector memory CD8+ T cells. Sci Immunol 2020;5(49):eaba7918. https://doi.org/10.1126/sciimmunol.aba7918.
[27] Schwartz RH. T cell anergy. Annu Rev Immunol 2003;21:305–334. https://doi.org/10.1146/annurev.immunol.21.120601.141110.
[28] Holla P, Dizon B, Ambegaonkar AA, et al. Shared transcriptional profiles of atypical B cells suggest common drivers of expansion and function in malaria, HIV, and autoimmunity. Sci Adv 2021;7(22):eabg8384. https://doi.org/10.1126/sciadv.abg8384.
[29] Victora GD, Dominguez-Sola D, Holmes AB, et al. Identification of human germinal center light and dark zone cells and their relationship to human B-cell lymphomas. Blood 2012;120(11):2240–2248. https://doi.org/10.1182/blood-2012-03-415380.
[30] Ramezani-Rad P, Chen C, Zhu Z, et al. Cyclin D3 Governs Clonal expansion of dark zone germinal center B cells. Cel Rep 2020;33(7):108403. https://doi.org/10.1016/j.celrep.2020.108403.
[31] Klein U, Casola S, Cattoretti G, et al. Transcription factor IRF4 controls plasma cell differentiation and class-switch recombination. Nat Immunol 2006;7(7):773–782. https://doi.org/10.1038/ni1357.
[32] Portugal S, Tipton C, Sohn H, et al. Malaria-associated atypical memory B cells exhibit markedly reduced B cell receptor signaling and effector function. Elife 2015;4:e07218. https://doi.org/10.7554/eLife.07218.001.
[33] **Dai D**, **Gu S**, **Han X**, et al. The transcription factor ZEB2 drives the formation of age-associated B cells. Science 2024;383(6681):413–421. https://doi.org/10.1126/science.adf8531.
[34] Cancro MP. Age-associated B cells. Annu Rev Immunol 2020;38:315–340. https://doi.org/10.1146/annurev-immunol-092419.
[35] Jenks SA, Cashman KS, Zumaquero E, et al. Distinct effector B cells induced by Unregulated Toll-like receptor 7 contribute to Pathogenic responses in systemic Lupus Erythematosus. Immunity 2018;49(4):725–739.e6. https://doi.org/10.1016/j.immuni.2018.08.015.
[36] Cambier JC, Gauld SB, Merrell KT, et al. B-cell anergy: from transgenic models to naturally occurring anergic B cells? Nat Rev Immunol 2007;7(8):633–643. https://doi.org/10.1038/nri2133.
[37] Cooper L, Xu H, Polmear J, et al. Type I interferons induce an epigenetically distinct memory B cell subset in chronic viral infection. Immunity 2024;57(5):1037–1055.e6. https://doi.org/10.1016/j.immuni.2024.03.016.
[38] Sharma A, Seow JJW, Dutertre CA, et al. Onco-fetal reprogramming of endothelial cells drives immunosuppressive macrophages in hepatocellular carcinoma. Cell 2020;183(2):377–394.e21. https://doi.org/10.1016/j.cell.2020.08.040.
[39] Jin S, Plikus MV, Nie Q. CellChat for systematic analysis of cell–cell communication from single-cell transcriptomics. Nat Protoc 2025;20:180–219. https://doi.org/10.1038/s41596-024-01045-4.
[40] Chung Y, Chang SH, Martinez GJ, et al. Critical regulation of early Th17 cell differentiation by Interleukin-1 signaling. Immunity 2009;30(4):576–587. https://doi.org/10.1016/j.immuni.2009.02.007.
[41] Esparza-Baquer A, Labiano I, Sharif O, et al. TREM-2 defends the liver against hepatocellular carcinoma through multifactorial protective mechanisms. Gut 2021;70(7):1345–1361. https://doi.org/10.1136/gutjnl-2019-319227.
[42] Yang D, Sun X, Wang H, et al. TREM2 depletion in pancreatic cancer Elicits Pathogenic inflammation and Accelerates tumor progression via enriching IL-1β+ macrophages. Gastroenterology 2025;168(6):1153–1169. https://doi.org/10.1053/j.gastro.2025.01.244.
[43] Pober JS, Sessa WC. Evolving functions of endothelial cells in inflammation. Nat Rev Immunol 2007;7(10):803–815. https://doi.org/10.1038/NRI2171.
[44] Gomes AL, Teijeiro A, Burén S, et al. Metabolic inflammation-associated IL-17A Causes non-alcoholic steatohepatitis and hepatocellular carcinoma. Cancer Cell 2016;30(1):161–175. https://doi.org/10.1016/j.ccell.2016.05.020.
[45] Rébé C, Ghiringhelli F. Interleukin-1β and cancer. Cancers (Basel) 2020;12(7):1791. https://doi.org/10.3390/cancers12071791.
[46] **Grohmann M**, **Wiede F**, Dodd GT, et al. Obesity drives STAT-1-dependent NASH and STAT-3-dependent HCC. Cell 2018;175(5):1289–1306.e20. https://doi.org/10.1016/j.cell.2018.09.053.
[47] Polyzos SA, Targher G. Role of glucocorticoids in metabolic dysfunction-associated steatotic liver disease. Curr Obes Rep 2024;13(2):242–255. https://doi.org/10.1007/S13679-024-00556-1.
[48] Kucsera D, Tóth VE, Sayour NV, et al. IL-1β neutralization prevents diastolic dysfunction development, but lacks hepatoprotective effect in an aged mouse model of NASH. Sci Rep 2023;13(1):356. https://doi.org/10.1038/s41598-022-26896-3.
[49] Anstee QM, Neuschwander-Tetri BA, Wong VW-S, et al. Cenicriviroc lacked Efficacy to Treat liver fibrosis in Nonalcoholic steatohepatitis: AURORA phase III Randomized study. Clin Gastroenterol Hepatol 2024;22(1):124–134.e1. https://doi.org/10.1016/j.cgh.2023.04.003.
[50] Kinkhabwala A, Herbel C, Pankratz J, et al. MACSima imaging cyclic staining (MICS) technology reveals combinatorial target pairs for CAR T cell treatment of solid tumors. Sci Rep 2022;12(1):1911. https://doi.org/10.1038/s41598-022-05841-4.

Keywords: liver cancer; tumor microenvironment; lipid-associated macrophage; granulopoiesis; endothelial activation.

Received 15 December 2025; received in revised form 22 September 2026; accepted 24 September 2026; Available online 7 October 2026

Journal of Hepatology, Volume *8*

Supplemental information

Hyperinflammatory reprogramming marks the transition from MASH to HCC

Shawn Lu Wen Tan, Erez Israeli, Jillian Wee, Hui Min Tan, Ravisankar Rajarethinam, Immanuel Kwok, Shu Zhen Chong, and Weiping Han

Hyperinflammatory reprogramming marks the transition from MASH to HCC

Shawn Lu Wen Tan, Erez Israeli, Jillian Wee, Hui Min Tan, Ravisankar Rajarethinam, Immanuel Kwok, Chong Shu Zhen, Weiping Han

Table of contents

Supplementary methods……………………………………………………………………………..2
Fig. S1…………………………………………………………………………………..........9
Fig. S2……………………………………………………………………………………11
Fig. S3……………………………………………………………………………………12
Fig. S4……………………………………………………………………………………14
Fig. S5……………………………………………………………………………………16
Fig. S6……………………………………………………………………………………18
Fig. S7……………………………………………………………………………………19
Fig. S8……………………………………………………………………………………20
Fig. S9……………………………………………………………………………………21
Table S1………………………………………………………………………………....22
Table S2………………………………………………………………………………....25
Table S3………………………………………………………………………………....30
Table S4………………………………………………………………………………....31
Table S5………………………………………………………………………………....34
Table S6………………………………………………………………………………....35
Table S7………………………………………………………………………………....37
Table S8………………………………………………………………………………....49
Table S9………………………………………………………………………………....53
Supplementary references…………………………………………………………….....55

Supplementary Methods

Serum measurements of ALT activity, TBA and AFP

Serum ALT activity, TBA and AFP were measured using the ALT Activity Assay Kit (Abcam, ab105134; 2 μL serum in duplicate), TBA Kit (Cell Biolabs, STA-631; 10 μL) and Mouse AFP ELISA Kit (R&D Systems, MAFP00; 2.5 μL), respectively, according to the manufacturers' instructions.

Immunohistochemistry of liver sections

FFPE liver sections (5 μm) were stained with H&E or Fast Green/Sirius Red (0.1% Direct Red 80, 0.1% Fast Green in 1.3% picric acid; Sigma-Aldrich). For Ki67 immunohistochemistry, sections were deparaffinized, rehydrated and subjected to antigen retrieval in citrate buffer (pH 6.0) at 90 °C for 20 min. Following peroxidase blocking (Bloxall, Vector Laboratories, SP-6000) and blocking with 10% goat serum, sections were incubated with anti-Ki67 (Abcam, ab15580; 1:1000) overnight at 4 °C, followed by biotinylated goat secondary antibody (1:200) for 30 min. Signal was developed using VECTASTAIN Elite ABC HRP and ImmPACT DAB substrate (Vector Laboratories, SK-4105). Sections were counterstained with hematoxylin, dehydrated and mounted in Permount (Fisher Scientific, SP15-500).

Preparation for bulk RNA-sequencing

RNAlater-preserved mouse liver (10–20 mg) was homogenized in RLT buffer containing β-mercaptoethanol using a TissueLyser II (20 Hz, 2 × 2 min). RNA was extracted using the RNeasy Plus Kit (Qiagen) according to the manufacturer's instructions, except that 50% instead of 70% ethanol was used for RNA precipitation.

RNA quantity and quality were assessed using a NanoDrop (Thermo Fisher Scientific) and Agilent 2100/2200 Bioanalyzer (Agilent Technologies). One microgram of total RNA was used for library preparation.

Library preparation for bulk RNA-sequencing

Sequencing libraries were prepared according to the manufacturer's protocol. Poly(A) mRNA was isolated using the Poly(A) mRNA Magnetic Isolation Module. RNA was fragmented and primed, followed by first- and second-strand cDNA synthesis using ProtoScript II Reverse Transcriptase and Second Strand Synthesis Enzyme Mix, respectively. Purified double-stranded cDNA was end-repaired, dA-tailed, adaptor-ligated and size-selected to recover ~400-bp fragments (~300-bp inserts). Libraries were PCR-amplified with indexed P5/P7 primers, bead-purified, validated using a Qsep100 (Bioptic), and quantified using a Qubit 3.0 Fluorometer (Invitrogen). Indexed libraries were multiplexed and sequenced on an Illumina NovaSeq using 2 × 150-bp paired-end reads.

Pre-processing of bulk RNA-sequencing

Fastq files were trimmed (15 bp) and checked for quality control metrics using Fastp [1] and aligned to mm10 mouse reference genome using STAR (2.7.11a) and further filtered to remove non-primary alignments, reads that fail platform quality checks, optical duplicates, or supplementary alignments using Samtools (1.6). Raw counts and normalized counts were extracted using HTSeq (2.0.3) and Cufflinks (2.2.1). Batch correction was performed using the *ComBat-seq* function from the sva R package (3.44.0). ComBat-seq-corrected counts were used for differential expression analyses

using DESeq2. Gene set enrichment analyses were performed using gprofiler2 R package.

Sample preparation for scRNA-sequencing

Mouse liver tissues were stored overnight in MACS Tissue Storage Solution (Miltenyi Biotec, 130-100-008), with tumor-bearing livers separated into tumor and adjacent non-tumor (Adj.NT) regions. Up to 1 g of tissue was minced and dissociated using the Tumor Dissociation Kit and gentleMACS Octo Dissociator (Miltenyi Biotec, 130-096-730; 37C_m_TDK_2 program) according to the manufacturer's instructions. Dissociated cells were filtered through a 70-μm strainer, subjected to red blood cell lysis (Miltenyi Biotec, 130-094-183), and washed with cold PBS before downstream processing.

Pre-processing of scRNA-sequencing data, quality control and clustering

Raw reads were processed using Cell Ranger v7.1.0 (*cellranger multi*) for hashtag-based sample deconvolution, alignment to the mm10 reference genome, and generation of gene expression and Feature Barcode matrices. Data were analyzed using Seurat v5.0.0. Cells were excluded if mitochondrial read percentage deviated by >2.5 median absolute deviations (MADs), or total counts or detected genes by >3 MADs from the sample median. Cells with abnormally high counts relative to detected genes were additionally excluded using a log-log linear regression model (intercept offset, 0.16). Data were normalized and scaled using *SCTransform* and integrated using *IntegrateData*. Clustering, UMAP visualization and differential expression analysis (*FindAllMarkers*, Wilcoxon test) were performed using Seurat. Clusters were

annotated based on marker gene expression. Major cell types were re-clustered and iteratively refined to remove suspected doublets and annotate subclusters.

Calculation of enrichment scores between sample groups for subclusters of the single-cell RNA-sequencing dataset.

To calculate enrichment of cell clusters across sample groups, we derived expected cell numbers for each cluster in each sample group using the Chi-square formula. Enrichment scores were quantified by dividing the observed number of cells by the expected number of cells for each cluster in each sample group.

Diffusion mapping and trajectory analyses

Diffusion maps were plotted using the destiny (3.23) R package [2]. The *Velocyto* python implementation [3] was used to generate loom files from BAM files of previous CellRanger output. Expression count matrices, gene names, PCA and UMAP embeddings were extracted from seurat objects and used to construct an anndata object using the anndata python package [4]. Utilising anndata objects, scVelo [5] and Scanpy [6] python packages were used to pre-process and compute RNA velocities using the dynamical model in *scVelo*. CellRank2 [7] was used to integrate RNA velocity kernels with gene similarity kernels in a 4:1 ratio to infer cellular trajectories. Initial and terminal macrostates were predicted by CellRank2 using the Generalized Perron Cluster Cluster Analysis (GPCCA) algorithm. Cellular differentiation scores were estimated using the CytoTRACE R package (0.3.3) based on the observation that the number of expressed genes decreases during differentiation [8].

Single-cell gene set enrichment

The *AddModuleScore* function in Seurat (5.0.0) was used to quantify enrichment scores for selected gene sets in single-cells using default parameters. The function calculates the average expression level of each gene set on the single-cell level and subtracts the aggregated expression of a control gene set randomly selected from each bin of genes binned based on averaged expression. Z-scores were calculated based on the enrichment scores and the average Z-score within each cluster/group was plotted in heatmaps using ComplexHeatmap R package. Hallmark, KEGG, Reactome and Gene Ontology Gene sets were derived from MSigDB database.

Curation of immature neutrophil signature and single sample gene set enrichment of human dataset.

Positive DEGs of subcluster N3 versus all other subclusters except for N6 subcluster (also an immature neutrophil subcluster) were extracted using *FindMarkers* function utilizing the Wilcoxon statistical test. A stringent filtering criteria of $\log_2$ fold change > 7 and adjusted *p*-value < 1e-20 was applied which resulted in eight top genes remaining (*Camp, Ngp, Cd177, Gpr27, Itgb2l, Ltf, F730016J06Rik, 4930438A08Rik*). Human orthologs for these genes are derived using the babelgene (22.9) R package, for which 5 human orthologs were available (*CAMP, CD177, GPR27, ITGB2, LTF*). These five genes constituted the human immature neutrophil signature.

G-CSFcx mouse experiment, immune cell isolation and flow cytometry

For each dose, 1.5 µg G-CSF (Neupogen, Amgen) was incubated with 7.5 µg anti-G-CSF monoclonal antibody (Southern Biotech, 10128-01) at 37 °C for 20 min to form G-CSF/anti-G-CSF complexes (G-CSFcx), then diluted to 150 µL with sterile saline. Starting at 48 weeks, GAN- and LFD-fed mice received six retro-orbital doses of G-

CSFcx or saline at 2-week intervals over 12 weeks. At sacrifice, 10 µL tail blood was mixed with 10 µL 0.5 M EDTA. Following red blood cell lysis, cells were stained with primary antibodies (1:1000 each) for 20 min at 4 °C, washed, and resuspended in PBS containing 1 µM DAPI and CountBright beads (1:100; Thermo Fisher Scientific, C36950) for flow cytometry. Livers were separated into tumor and adjacent non-tumor regions where applicable. Up to 1 g of tissue was minced and digested for 30 min at 37 °C in RPMI containing DNase I (0.25 mg/mL; Sigma-Aldrich, 10104159001), collagenase IV (385 U/mL; Sigma-Aldrich, C51380) and 10% FBS, using 2 mL digestion buffer per gram of tissue. Samples were processed on a gentleMACS Octo Dissociator using the m_impTumour_03 and m_impTumour01 programs twice each, filtered through 70-µm MACS SmartStrainers (Miltenyi Biotec, 130-098-462), and enriched by 33% Percoll centrifugation (700 × g, 12 min, room temperature). Following red blood cell lysis, cells were stained with anti-Ly6G (BioLegend, 127606), anti-CD11b (BD Biosciences, 563402), anti-Siglec-F (BD Biosciences, 562757) and anti-CD101 (Thermo Fisher Scientific, 12-1011-82) at 1:200 for 20 min at 4°C. Cells were washed and resuspended in PBS containing 3 µM DAPI and CountBright beads (1:10) before analysis on a NovoCyte Penteon flow cytometer (Agilent Technologies). Data were analyzed using FlowJo v10.9.0.

Generation and cell culture of RAW264.7 Trem2 KO using CRISPR/Cas9.

Single-guide RNAs (sgRNAs) targeting murine *Trem2* were cloned into PX458 plasmid (Addgene, 48138) and transfected into RAW264.7 macrophages using HiPerFect Transfection Reagent (Qiagen, 301704). Single GFP-positive cells were sorted into individual wells of a 96-well plate. Clones were picked and screened for successful Trem2 KO using anti-Trem2 antibody (Cell Signaling, 61788) via western blot.

Successful Trem2 KO clones were verified using Sanger sequencing. RAW264.7 macrophages were cultured in DMEM containing 10% dialyzed fetal bovine serum (ThermoFisher, 26400044) and 1% penicillin/streptomycin in humidified 37°C incubators with 5% CO2.

Immunofluorescence of RAW264.7 cells

RAW264.7 cells were seeded on glass coverslips and fixed the next day with 4% paraformaldehyde for 10 min at room temperature. Following PBS washes, cells were permeabilized with 1X PBS/0.1% Triton-X for 10 min and blocked with 2% bovine serum albumin for 30 min. Cells were incubated with primary antibodies (anti-Trem2, Abcam, ab305103, 1:250) for 2h at room temperature. Cells were washed 3x with 1X PBS. Anti-rabbit Alexa Fluor 647 antibodies were applied for 30 min at room temperature. Cells were washed 3x with 1X PBS, treated with 1 µg/ml DAPI for 5 min and mounted on glass slides using FluorSave Reagent (Sigma Aldrich, 345789). Images were acquired using a Nikon Ni-E Upright microscope and analyzed using ImageJ.

Figure S1, related to Figure 1

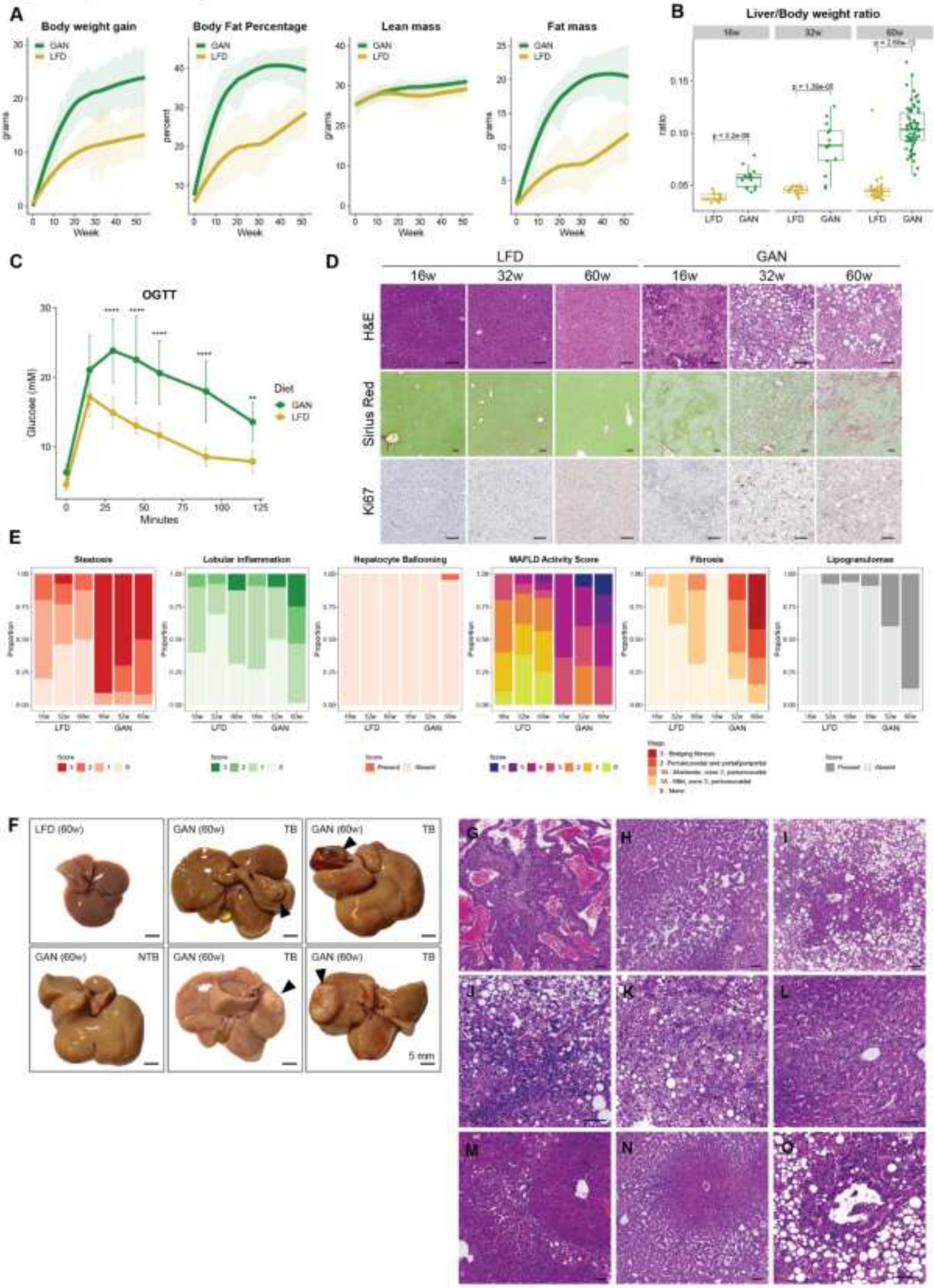

Figure S1, related to Figure 1

A) Longitudinal measurements of body weight and composition parameters of mice challenged with LFD or GAN diets across the study duration. Darker solid lines indicate the smoothed mean values, lighter shaded areas indicate the standard deviation.
B) Liver-to-body weight ratios of mice at the various timepoints. WIlcoxon rank-sum test.
C) Oral glucose intolerance test of LFD (n=5) and GAN (n=5) mice following 16 weeks of diet exposure. Mean ± SD shown, two-tailed Student’s t-test.
D) Representative images of liver sections stained with hematoxylin and eosin, Sirius red, and Ki67. Scale bars represent 50 μm.
E) Histopathology scoring of liver sections across the entire cohort. See Methods for details on scoring criteria.
F) Representative pictures of whole livers. TB – tumor-bearing; NTB – non-tumor-bearing. Black arrows indicate where tumors are observed.
G) H&E image of a HCC with trabecular pattern featuring blood lakes.
H) H&E image of a HCC with trabecular pattern.
I) H&E image of a HCC with trabecular pattern with macrovesicular steatosis.
J) H&E image of a steatohepatitis HCC with macrovesicular steatosis and immune infiltration.
K) H&E image of a HCC with clear-cell pattern.
L) H&E image of a HCC with clear-cell pattern.
M) H&E image of a HCC with mixed trabecular and solid patterns.
N) H&E image of a HCC with mixed trabecular and solid patterns.
O) H&E image of vascular invasion of cancer cells into a neighbouring vessel. Scale bars represent 50 μm.

Figure S2, related to Figure 1

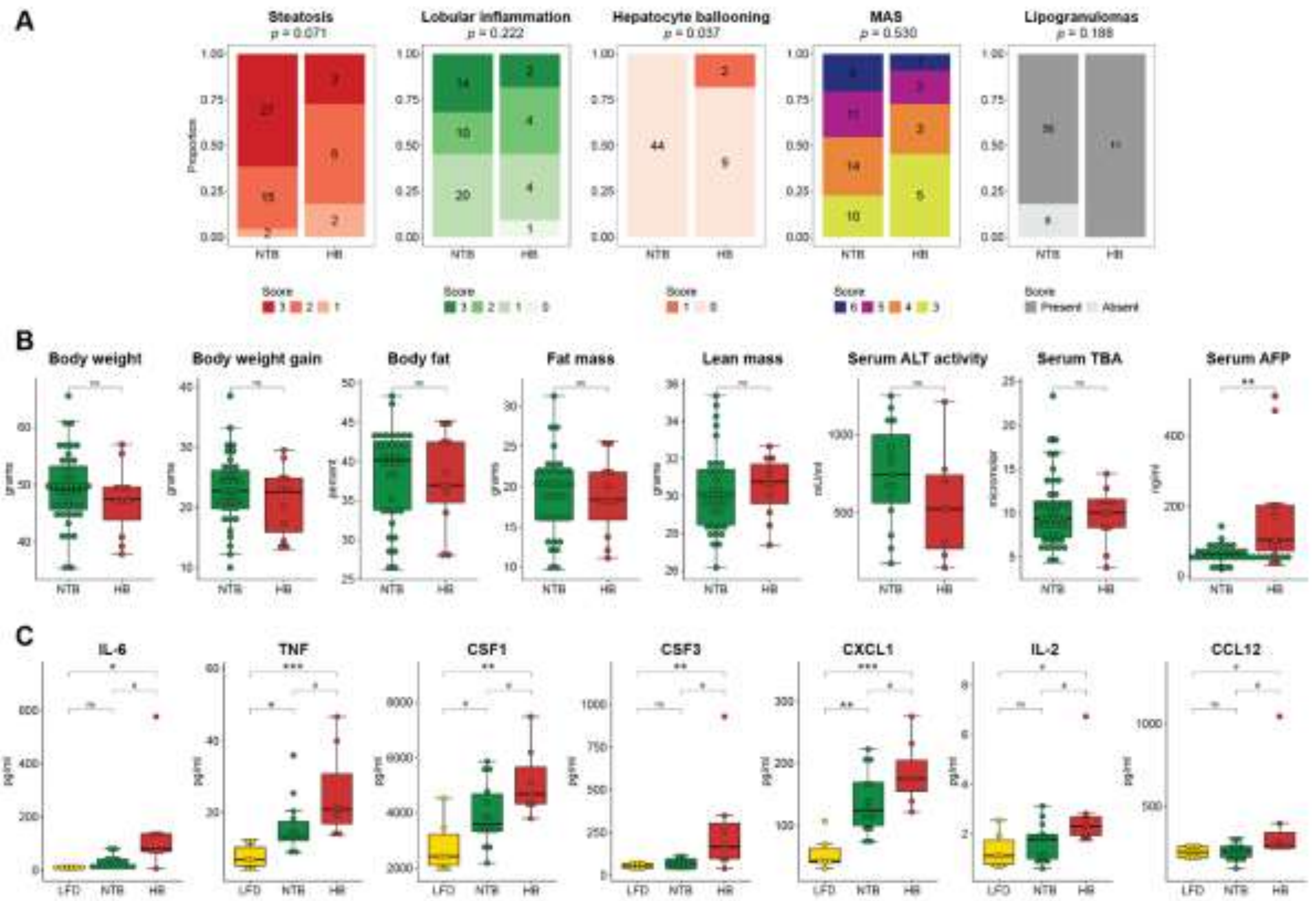

Figure S2, related to Figure 1

A) Pathology evaluation scores of NTB and HB 60-week GAN liver sections for steatosis, lobular inflammation, hepatocyte ballooning, MAFLD activity score (MAS) and presence of lipogranulomas. Fisher's exact test.

B) Body weight, body composition parameters and abundance of serum markers in NTB and HB 60-week GAN mice. WIlcoxon rank-sum test.

C) Absolute abundance of significantly different cytokines highlighted in red in Figure 1L. Post-hoc Dunn's test following Kruskal-Wallis test, Benjamini-Hochberg adjusted *p*-values. *p* >0.1, ns; *p* <0.1, #; *p* <0.05, *; *p* <0.01, **; *p* <0.001, ***.

Figure S3, related to Figure 2

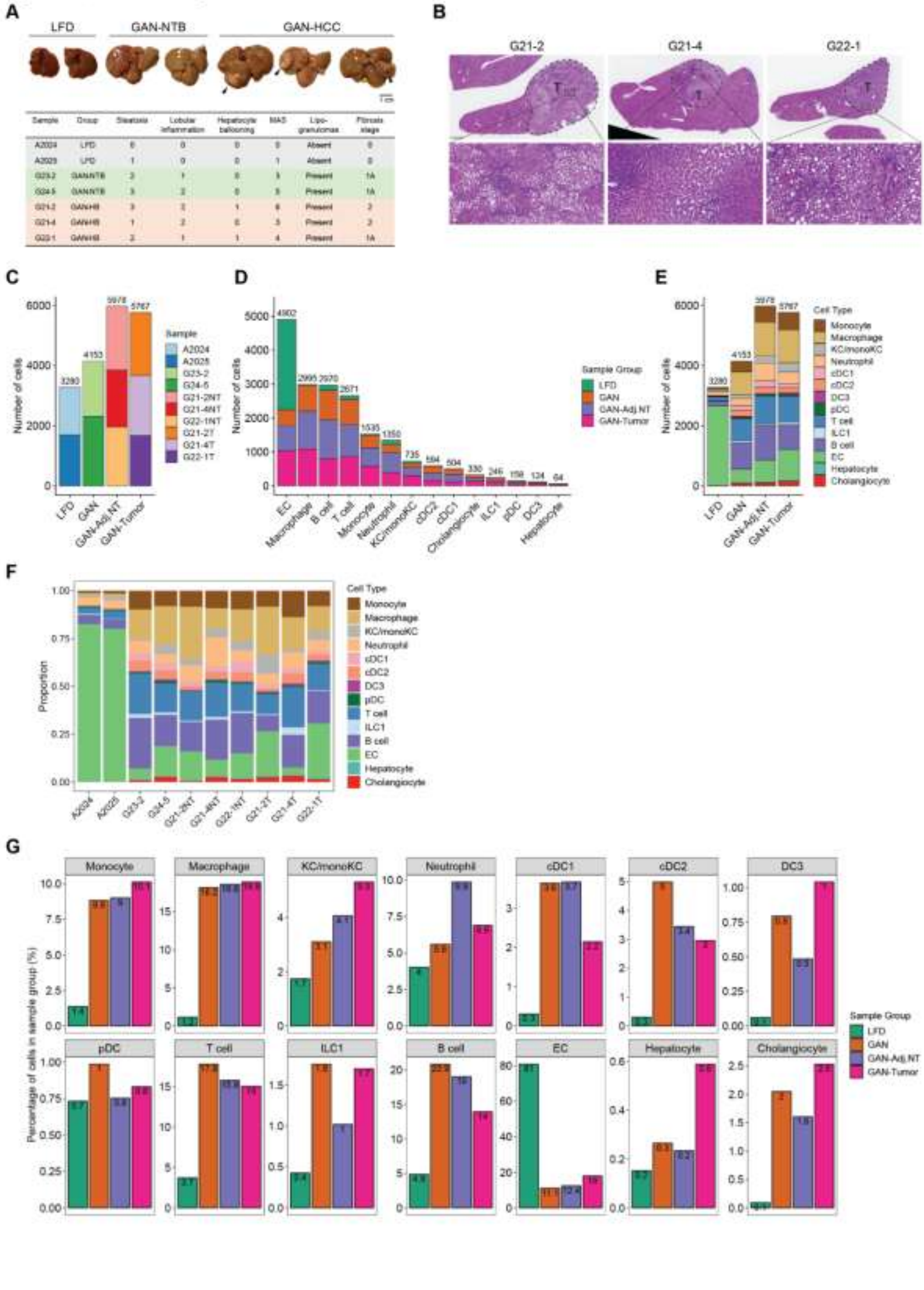

Sample	Group	Steatosis	Lobular inflammation	Hepatocyte ballooning	NAS	Lipo-granulomas	Fibrosis stage
A2024	LFD	0	0	0	0	Absent	0
A2025	LFD	1	0	0	1	Absent	0
G23-2	GAN-NTB	2	1	0	3	Present	1A
G24-5	GAN-NTB	3	2	0	5	Present	1A
G21-2	GAN-HB	3	2	1	6	Present	2
G21-4	GAN-HB	1	2	0	3	Present	2
G22-1	GAN-HB	2	1	1	4	Present	1A

Figure S3, related to Figure 2.

A) Images of livers used for single-cell transcriptomics. Bottom table shows histopathology scores of liver sections taken from each liver.
B) H&E images of GAN-HCC livers used for single-cell RNA-sequencing.
C) Number of cells in each sample group contributed by individual samples.
D) Number of cells in each cell type contributed by each sample group.
E) Number of cells in each sample group contributed by each cell type.
F) Proportion of cell types in each sample.
G) Percentage of each cell type in each sample group.

Figure S4, related to Figure 3

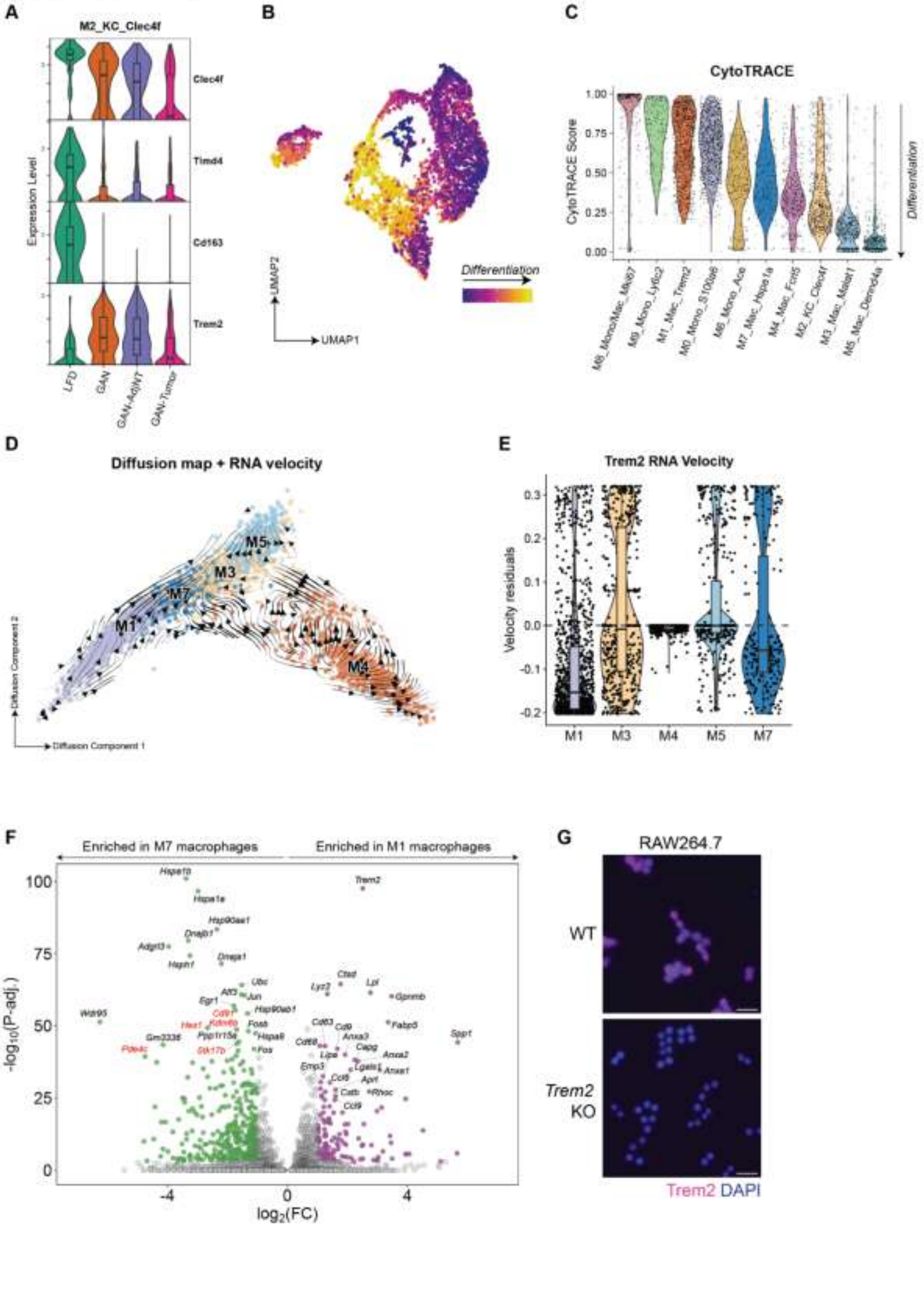

Figure S4, related to Figure 3.

A) Expression of genes in M2_KC_Clec4f cells across sample groups.
B) UMAP showing CytoTRACE scores of monocytes and macrophages to predict degree of differentiation.
C) CytoTRACE scores across monocyte and macrophage subclusters.
D) Diffusion map and RNA velocity vectors of macrophage subclusters.
E) RNA velocity residuals of Trem2 in macrophage subclusters.
F) Differential expression analysis of M1 and M7 macrophages.
G) Immunofluorescence staining of RAW264.7 macrophages for Trem2 (magenta) and DAPI (dark blue). Scale bars represent 20 μm. WT – wild type, KO – knockout.

Figure S5, related to Figure 4

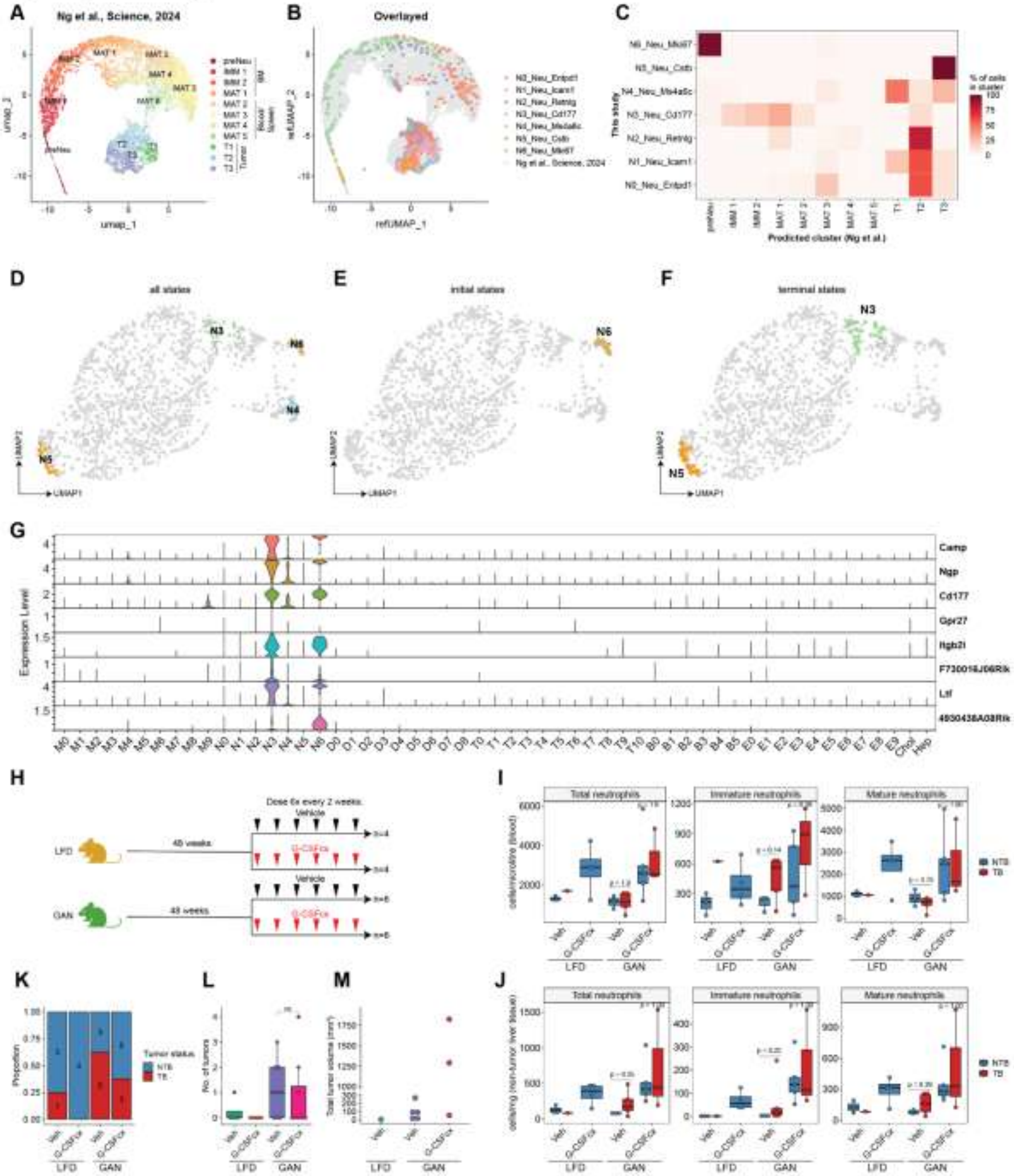

Figure S5, related to Figure 4.

A) UMAP of neutrophils from[9] to be used as reference for S5B.

B) Overlaying of neutrophils from this study onto the reference UMAP of S5A.

C) Percentage of each neutrophil subcluster in this study predicted to overlap with respective clusters from [9].

D) Prediction of macrostates by CellRank2.

E) Prediction of initial macrostates by CellRank2.

F) Prediction of terminal macrostates by CellRank2.

G) Expression of the top eight genes enriched in N3 and N6 compared with all other subclusters in the dataset.

H) Experimental set-up for G-CSFcx *in vivo* experiment.

I) Absolute number of neutrophils ($Ly6G^{+}Cd11b^{+}$), immature neutrophils ($Cd101^{-}Ly6G^{+}Cd11b^{+}$) and mature neutrophils ($Cd101^{+}Ly6G^{+}Cd11b^{+}$) in peripheral blood as measured by flow cytometry. WIlcoxon rank-sum test.

J) Absolute number of neutrophils ($Ly6G^{+}Cd11b^{+}$), immature neutrophils ($Cd101^{-}Ly6G^{+}Cd11b^{+}$) and mature neutrophils ($Cd101^{+}Ly6G^{+}Cd11b^{+}$) in liver tissues as measured by flow cytometry. WIlcoxon rank-sum test.

K) Proportion of non-tumor-bearing (NTB) and tumor-bearing (TB) mice in the G-CSFcx experiment.

L) Number of tumors (> 2 mm^3) in each liver in the G-CSFcx experiment. WIlcoxon rank-sum test.

M) Total tumor volume per liver in the G-CSFcx experiment.

Figure S6, related to Figure 5

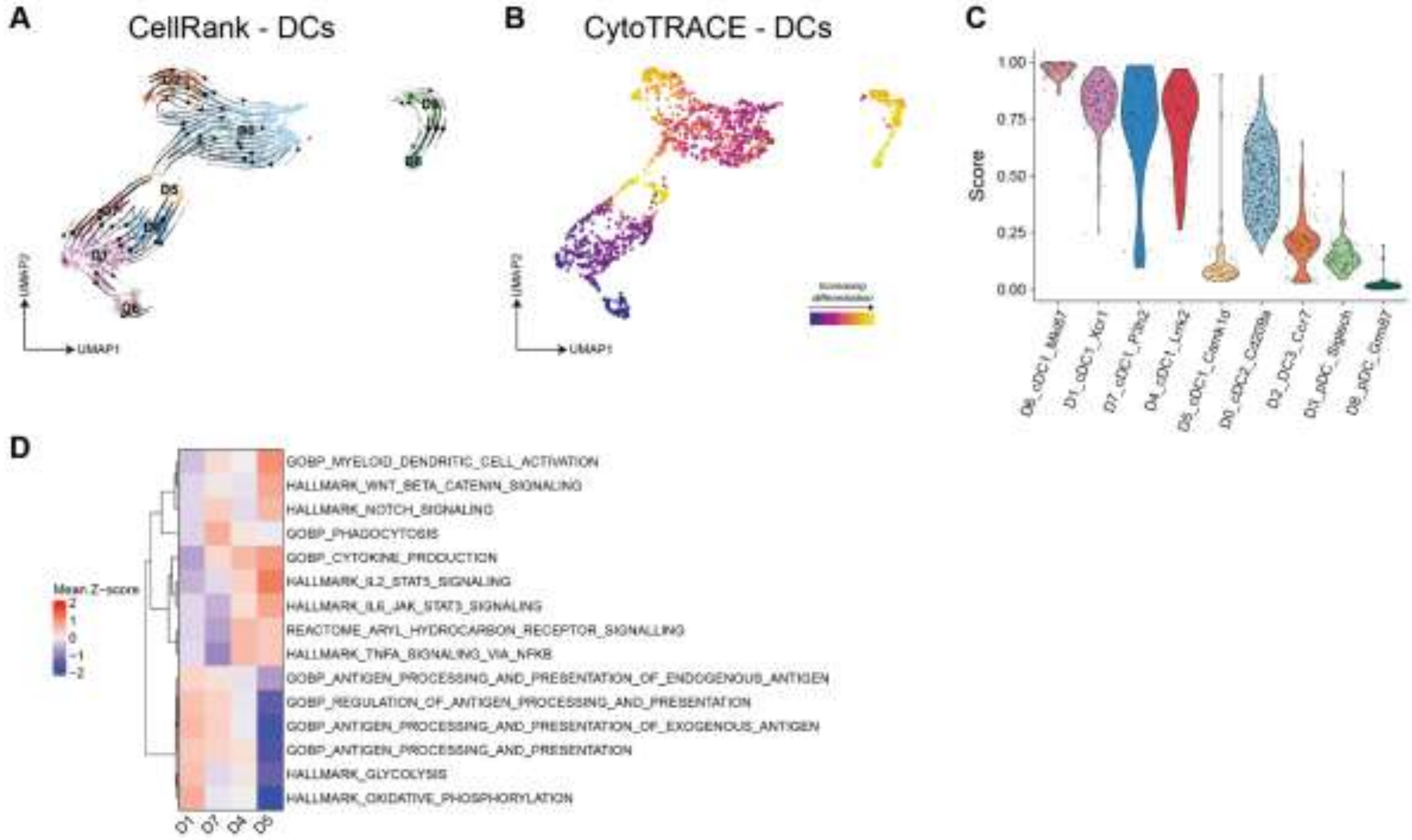

Figure S6, related to Figure 5

A) Trajectory analysis of dendritic cells by CellRank2 based on RNA velocity and gene similarity.
B) UMAP showing CytoTRACE scores of dendritic cells.
C) CytoTRACE scores of dendritic cells across subclusters.
D) Single-cell gene set enrichment Z-scores of gene sets across cDC1 clusters.

Figure S7, related to Figure 6

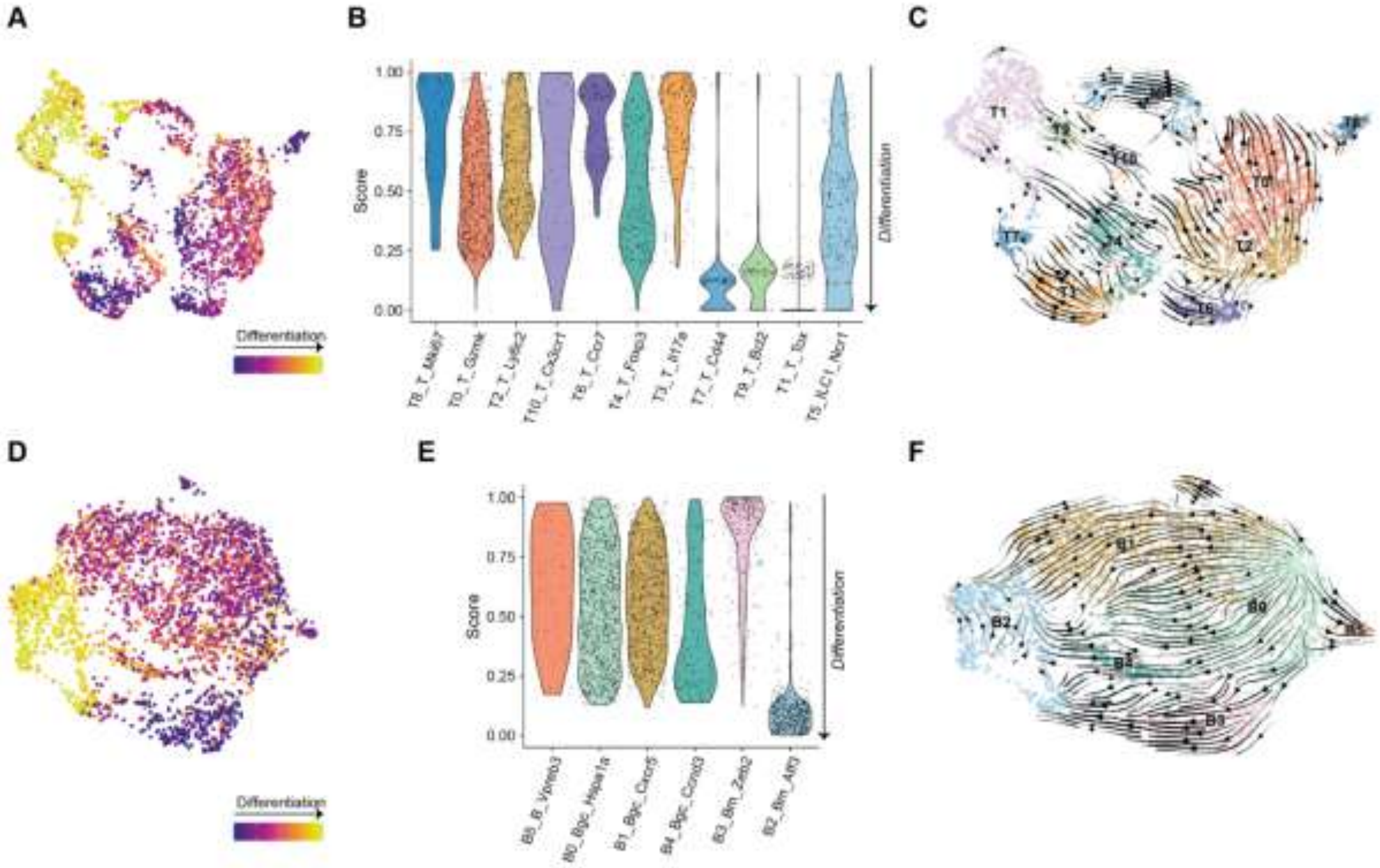

Figure S7, related to Figure 6.

A) UMAP showing CytoTRACE scores of T cells and ILC1.

B) CytoTRACE scores of T cells and ILC1 across subclusters.

C) Trajectory analysis of T cells and ILC1 by CellRank2 based on RNA velocity and gene similarity.

D) UMAP showing CytoTRACE scores of B cells.

E) CytoTRACE scores of B cells across subclusters.

F) Trajectory analysis of B cells by CellRank2 based on RNA velocity and gene similarity.

Figure S8, related to Figure 7

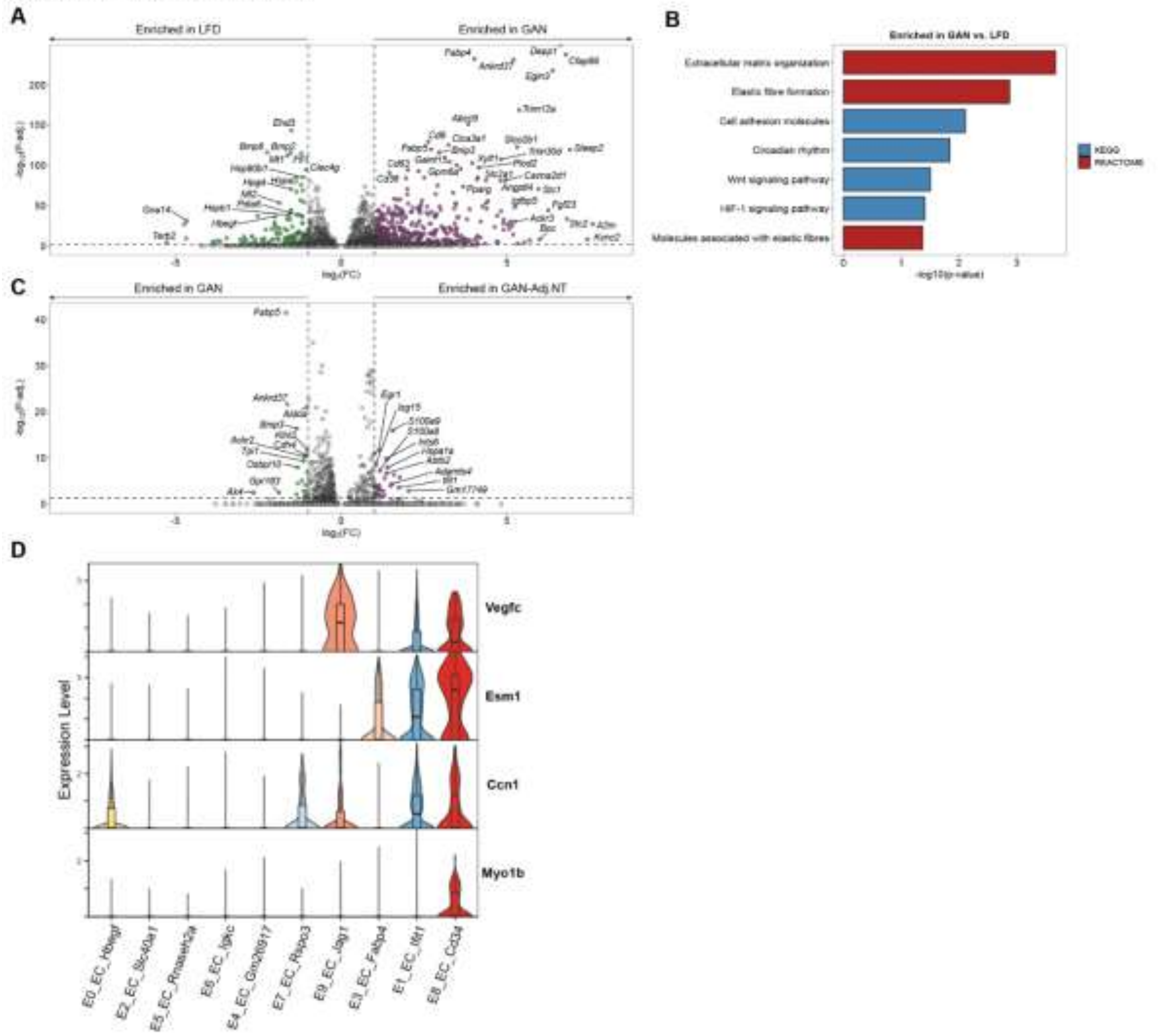

Figure S8, related to Figure 7.

A) Volcano plot showing differential gene expression between GAN and LFD ECs.

B) KEGG and Reactome pathway enrichment analysis of genes upregulated in GAN versus LFD ECs. P-values calculated using the g:SCS algorithm of gProfiler2.

C) Volcano plot showing differential gene expression between GAN-Adj.NT vs GAN ECs.

D) Expression of pro-angiogenic factors across EC subclusters.

Figure S9, related to Figure 8

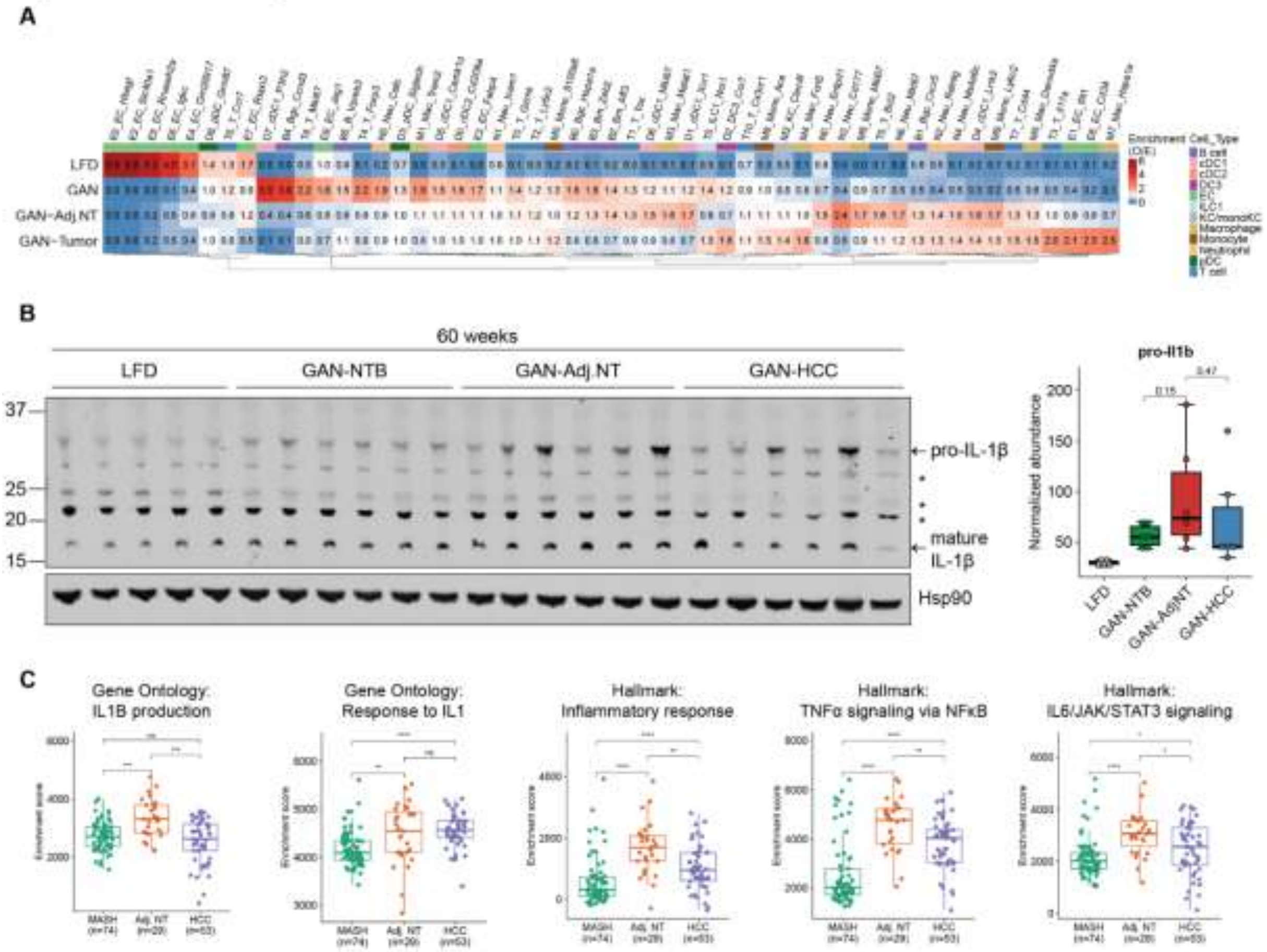

Figure S9, related to Figure 8

A) Hierarchical clustering of enrichment scores for all 53 subclusters across sample groups.

B) Western blot of IL-1β in liver tissues from mice fed the LFD or GAN diets for 60 weeks. Boxplot on the right shows the densitometric measurements of pro-IL1b normalised to Hsp90. WIlcoxon rank-sum test.

C) ssGSEA enrichment scores for the indicated gene signatures in liver tissues from a human cohort of non-cirrhotic MASH and MASH-HCC patients. WIlcoxon rank-sum test.

Supplementary Table 1. Pairwise transcriptomic comparison between HB-Adj.NT and NTB liver tissues. Significant DEGs (adjusted p-value < 0.05) shown.

Gene	baseMean	log2FoldChange	lfcSE	pvalue	padj
Pla2g2d	32.656	4.912	0.993	2.81E-07	4.98E-04
Ccl8	75.854	4.088	0.893	1.26E-06	1.35E-03
Cbr2	9.751	2.799	0.776	4.09E-05	1.45E-02
Ltf	129.477	2.685	0.897	3.04E-07	4.98E-04
Olfm4	6.270	2.660	0.784	8.22E-05	2.00E-02
Cd177	35.935	2.645	0.732	4.01E-05	1.45E-02
Mmp9	114.983	2.623	0.562	4.31E-07	4.98E-04
Alox5	31.890	2.534	0.708	4.23E-05	1.46E-02
Ppbp	111.669	2.523	0.579	1.64E-06	1.52E-03
S100a8	202.046	2.516	0.700	4.07E-05	1.45E-02
Ngp	230.503	2.267	0.941	1.78E-06	1.54E-03
Ccl12	10.320	2.263	0.719	0.00016	3.13E-02
Pglyrp1	29.339	2.135	0.562	1.50E-05	7.40E-03
Tubb1	76.243	2.110	0.623	6.87E-05	1.79E-02
Clec10a	41.364	1.864	0.404	4.02E-07	4.98E-04
Ifitm6	34.359	1.801	0.392	3.98E-07	4.98E-04
Clec4e	44.175	1.792	0.531	6.33E-05	1.72E-02
Trem3	9.437	1.774	0.568	0.00014	2.82E-02
Xlr	31.952	1.764	0.488	2.67E-05	1.19E-02
Ccr3	12.915	1.763	0.570	0.00016	3.13E-02
Lyz1	73.502	1.749	0.499	4.02E-05	1.45E-02
Slc1a3	26.471	1.730	0.422	3.82E-06	2.64E-03
Slc14a1	17.165	1.687	0.405	2.56E-06	2.08E-03
Clec4a1	342.499	1.458	0.299	8.92E-08	2.47E-04
Cd4	78.757	1.402	0.422	6.53E-05	1.74E-02
Fpr1	174.160	1.387	0.304	3.88E-07	4.98E-04
Mcemp1	17.378	1.359	0.457	0.00018	3.39E-02
Clec4n	381.365	1.348	0.283	1.44E-07	3.33E-04
Itgam	446.432	1.346	0.388	3.93E-05	1.45E-02
Pf4	154.457	1.324	0.477	0.00036	4.92E-02
G530011O06Rik	70.967	1.304	0.393	6.23E-05	1.72E-02
Ly6c2	80.186	1.282	0.458	0.00033	4.87E-02
Slc28a2	70.377	1.272	0.299	1.61E-06	1.52E-03
F630028O10Rik	12.342	1.238	0.402	0.00012	2.62E-02
Wfdc17	752.983	1.227	0.307	4.55E-06	3.00E-03
Alox5ap	217.141	1.194	0.314	9.93E-06	5.73E-03
A530064D06Rik	21.969	1.169	0.325	2.07E-05	9.86E-03
Cd300e	93.689	1.100	0.394	0.00030	4.63E-02
Rab37	12.556	1.098	0.362	0.00012	2.48E-02
Il2rg	450.681	1.088	0.341	8.69E-05	2.01E-02
Aoah	267.281	1.083	0.327	5.57E-05	1.64E-02

Ebi3	49.745	1.075	0.314	3.75E-05	1.45E-02
Ltb4r1	20.841	1.050	0.323	6.04E-05	1.72E-02
Pira2	47.565	1.037	0.325	8.23E-05	2.00E-02
Siglece	276.246	1.004	0.268	1.15E-05	6.24E-03
Clec4a2	230.006	0.963	0.304	8.61E-05	2.01E-02
Adgre4	323.501	0.958	0.252	8.15E-06	4.91E-03
Prune2	62.098	0.945	0.347	0.00036	4.92E-02
Pirb	499.728	0.945	0.255	1.17E-05	6.24E-03
1600010M07Rik	11.786	0.944	0.355	0.00033	4.87E-02
Emilin2	221.185	0.919	0.327	0.00027	4.41E-02
Sell	121.831	0.918	0.310	0.00017	3.25E-02
Klra2	180.291	0.915	0.247	1.29E-05	6.59E-03
Cfp	1153.663	0.901	0.234	6.96E-06	4.38E-03
Vsig4	2155.248	0.872	0.316	0.00030	4.64E-02
Clec4a3	371.114	0.860	0.257	4.38E-05	1.48E-02
Ncf1	494.359	0.851	0.248	3.35E-05	1.45E-02
Rac2	675.392	0.842	0.266	7.65E-05	1.93E-02
Csf3r	183.341	0.817	0.294	0.00028	4.51E-02
Cd300lf	73.501	0.803	0.288	0.00026	4.28E-02
P2rx7	301.100	0.795	0.241	4.79E-05	1.58E-02
Cybb	3443.118	0.790	0.250	8.44E-05	2.01E-02
Dock11	183.378	0.788	0.262	0.00014	2.85E-02
Chp2	94.727	0.783	0.193	2.98E-06	2.30E-03
Lrrc25	199.423	0.778	0.236	5.21E-05	1.61E-02
Adgre1	2086.430	0.774	0.235	5.24E-05	1.61E-02
Pilrb2	67.532	0.755	0.246	0.00011	2.33E-02
Fcer1g	750.294	0.749	0.280	0.00035	4.92E-02
Pilrb1	78.800	0.725	0.258	0.00025	4.21E-02
Fgd2	112.764	0.724	0.223	6.19E-05	1.72E-02
Ctsc	4704.155	0.720	0.183	3.28E-06	2.39E-03
Fcgr4	646.624	0.714	0.253	0.00021	3.90E-02
Naaa	242.579	0.697	0.223	9.34E-05	2.09E-02
Prkcb	387.397	0.678	0.246	0.00027	4.41E-02
Hck	454.440	0.668	0.239	0.00024	4.20E-02
Pld4	649.666	0.658	0.233	0.00023	4.03E-02
Nlrp3	72.413	0.652	0.238	0.00028	4.47E-02
P2ry12	169.497	0.628	0.223	0.00022	3.99E-02
P2ry13	247.545	0.612	0.184	4.08E-05	1.45E-02
Fam49b	658.257	0.610	0.192	7.15E-05	1.83E-02
Nfam1	407.598	0.599	0.217	0.00025	4.20E-02
Msr1	1205.189	0.596	0.220	0.00029	4.51E-02
Trpm2	253.465	0.594	0.222	0.00032	4.74E-02
Csf1r	3938.611	0.582	0.209	0.00025	4.20E-02
Gmfg	285.282	0.527	0.205	0.00036	4.92E-02
Zxdb	75.643	0.496	0.172	0.00016	3.12E-02
Lbr	368.007	0.454	0.140	4.90E-05	1.58E-02

Mxd1	229.890	0.436	0.146	0.00011	2.34E-02
Atp13a2	651.094	0.435	0.148	0.00014	2.88E-02
Tmem164	487.919	0.429	0.148	0.00016	3.13E-02
Ogfrl1	725.001	0.421	0.130	5.56E-05	1.64E-02
Slc9a9	254.112	0.381	0.143	0.00034	4.91E-02
Lyn	1869.741	0.358	0.140	0.00031	4.65E-02
Hps3	274.856	0.355	0.104	2.55E-05	1.18E-02
Zdhhc20	600.596	0.311	0.114	0.00031	4.70E-02
Fbxl14	355.235	0.292	0.095	8.87E-05	2.01E-02
Tbc1d14	798.027	0.220	0.083	0.00036	4.92E-02
Grb2	2105.647	0.181	0.071	0.00035	4.92E-02
Pmm2	1362.557	-0.192	0.074	0.00037	4.94E-02

Supplementary Table 2. Immune cell gene signatures from PanglaoDB.

Cell Type	Gene signature (PangLaoDB)
B_cells	*Cd2, Cd5, Ms4a1, Cr2, Cd22, Cd40, Cd69, Cd70, Cd79a, Cd79b, Cd80, Cd86, Tnfrsf9, Sdc1, Tnfsf4, Tnfrsf13b, Tnfrsf13c, Pdcd1, Ighm, Rasgrp3, Ltb, Fli1, Cd14, Sema6d, Lair1, Ifit3, Igll1, Dntt, Mme, Spn, Cd19, Cd27, B3gat1, Cd72, Pax5, Jchain, Mzb1, Ly6d, Fcmr, Bank1, Edem1, Vpreb3, Pou2af1, Creld2, Derl3, Ralgps2, Fchsd2, Pold4, Tnfrsf17, Hvcn1, Fcrla, Edem2, Blnk, Txndc11, Btla, Smap2, Fkbp11, Sec61a1, Spcs3, Spib, Eaf2, Cxcr4, Birc3, Iglc1, Il21r, Igkc, Vpreb1, Klhl6, Slamf6, Bst1, Msh5, Dok3, Bach2, Pxk, Ighg1, Cd38, Ptprc, Ebf1, Bcl11a, Ccr7, Cd55, Cd74, Cd52, Tlr9, Swap70, Hmga1*
B_cells_memory	*Cd38, Cd80, Cd84, Cd86, Nt5e, Pax5, Ptprc, Spib, Adam28, Aim2, Alox5, Bach2, Bank1, Blk, Ccr6, Cd180, Cd19, Cd22, Cd27, Cd37, Cd69, Cd72, Cd79a, Cd79b, Cr2, Cxcr5, Dennd5b, Fcgr2b, Frk, Gng7, Hhex, Ighm, Igkc, Il7, Irf8, Ltb, Ly86, Mbl2, Ms4a1, Nmbr, P2rx5, Pnoc, Ptprcap, Ralgps2, Rasgrp2, Sik1, Sit1, Slc12a1, Sp140, Stap1, Tmem156, Tnfrsf13b, Tnfrsf17, Traf4, Vpreb3, Zbtb32*
B_cells_naive	*Cd19, Cd2, Cd72, Il2ra, Mme, Abcb4, Adam28, Bach2, Bank1, Bcl7a, Bend5, Blk, Braf, Cd180, Cd22, Cd37, Cd69, Cd79a, Cd79b, Cr2, Cxcr5, Eaf2, Fcgr2b, Frk, Hhex, Ighm, Igkc, Irf8, Ltb, Ly86, Mep1a, Mical3, Ms4a1, Nmbr, P2rx5, F630111L10Rik, Pnoc, Ptprcap, Ralgps2, Rasgrp2, Sell, Sik1, Slc12a1, Spib, Stap1, Ugt1a10, Vpreb3, Tnfsf8, Foxp1, Mitf, Cd52, Tnfrsf13c, Stag3, Birc3, Cd74*
Basophils	*Cd69, Il3ra, Ccr3, Enpp3, Fcer1a, Tlr4, Ltc4s, Cpa3, Itgal, Itgam, Itgax, Itgb7, Il4, Ms4a2, Ms4a3, Hdc, Gata2, Ccl3, Cd33, Cd44, Cdh1, Htr1b, Igfbp7, Dnase2b, Fads2, Gpc4, Grm6, Acss2, Adora2b, Aqp9, Bace1, Bmp4, Cables1, Ccl4, Cebpa, E2f8, Fads1, Far2, Fbxo9, Gabbr1, Gpr183, Hgf, Ifitm1, Inf2, Jazf1, L1cam, Lama5, Mboat1, Nfil3, Npl, Osbpl5, Otub2, P2ry1, Pdk3, Pdzd4, Perp, Plekhg5, Plg, Rnf39, Slc26a3, Slc26a8, Sncaip, St6galnac5, Tulp3, Tec, Unc93a, Wrn, Id1*
Cholangiocytes	*Sstr2, Slc4a2, Sctr, Krt7, Kcnn2, Itgb4, Ggt6, Cftr, Cckbr, Aqp4, Aqp1, Pigr, Ggt1, Jag1, Gpbar1, Ggt7, Krt19, Onecut2, Alpl, Hnf1b, Alb, Agr2, Tff3, Tff1, Sox9, Epcam, Cldn4, Mmp7, Tff2, Scgb3a1, Fxyd2, Defb1, Lcn2, Cxcl1, Lgals2, Tacstd2, Elf3, Spp1, Muc5b, Lgals4*
Dendritic_cells	*Il6, Cd86, Cd83, Cr2, Tlr9, Lamp3, Trem2, Fabp4, S100a9, Arg1, Clec10a, Dnase1l3, Clec9a, Etv6, Cd163, Cxcr4, Vsig4, Nr4a3, Ccr7, Traf1, Relb, Batf3, Ccl22, Slamf7, Xcr1, Cxcl16, Scimp, Fcgr2b, Fgd2, Rab7b, Naaa, Hck, Cd180, Hfe, Ccr2, Ryr1, Itgae, Sema4a, Dpp4, Slamf8, Cxcr3, Btla, Flt3, Tlr3, Itgax, Gpr132, Adam19, Ap1s3, Ass1, Adgrg5, Gpr68, Kit, Kmo, P2ry10, Rab30, Zbtb46, S100a4, Clec7a, Aif1, Lst1, Ctss, Irf8, Adgre1, Ccl17, Cd14, Cd207, Cd8a, Cx3cr1, Itgam, Ly75, Pdcd1lg2, Ptprc, Sirpa, Axl, Ppp1r14a, Cd22, Dab2, S100a8, Vcan, Anxa1, Fcer1a, Cadm1, Camk2d, Lgals3, Napsa, Plbd1,*

	Rnase6, Plac8, Slc11a1, Pdpn, S100b, Cd28, Ppl, Slurp1, Serpinb9
Eosinophils	*C3ar1, Fut4, C5ar1, Cxcr3, Ccr1, Ccr3, Cd244a, Ptgdr2, S100a9, Pglyrp1, Ltc4s, Epx, Cd69, Il5, Prg2, Prg3, Cpa3, Ikzf2, Ccl11, Ccl5, Csf2, Il5ra*
Gamma_delta_T_cells	*Ptgds, Gzmb, S100b, Nkg7, Prf1, Hopx, Cst7, Klrd1, Ctsw, Spon2, Ifitm1, Gzma, Cd247, Clic3, Cd7, Adgrg1, Ccl5, Stmn1, Hmgb2, Tyms, Mki67, Ube2c, Tuba1b, Aspm, Cenpa, Top2a, Pcna, Aurkb, Birc5, Nusap1, Troap, Cenpf, Ccnb1, Trgv7, Trdv5*
Hepatic_stellate_cells	*Adamts13, Cygb, Pparg, Pdgfra, Rgs5, Ppara, Foxf1, Alb, Nr1h4, Sema7a, Pnpla3, Wt1, Fgf10, Gfap, Ngfr, Vegfa, Acta2, Myb, Des, Fap, Slc8a1, Reln, Syp, Vcl, Timp1, Col1a1, Tagln, Col1a2, Col3a1, Sparc, Rbp1, Dcn, Myl9, Tpm2, Bgn, Igfbp7, Igfbp3, Olfml3, Igfbp6, Ccl2, Colec11, Hgf*
Hepatoblasts	*Afp, Alb, Cebpa, Krt14, Dlk1, Cdh1, Foxm1, Ggt1, Hnf4a, Prox1, Map2k4, Smad5, Krt18, Krt8, Hnf1b, Hhex, Met*
Hepatocytes	*Fosl1, Eppk1, Ucp2, Gck, Lrp5, Slc10a1, Nos2, Atp7b, Hnf4a, Gjb2, Fgfr4, Prox1, Crp, Slc2a2, Tfr2, Kif13b, Lipc, Vdr, Asgr1, Arg1, Afp, Alb, Otc, Ugt1a1, Zhx2, Hhex, Foxa3, Foxa2, Foxa1, Cyp7a1, Cyp2e1, Cyp1a2, Cebpa, Cdh1, Gpc3, Ahr, Cyp1a1, Nr1i3, Cps1, Gls2, Pck1, Tat, Wt1, Prrg4, Sult1a1, Apoh, Ctnnb1, Abcc3, Fgb, Aqp3, Plscr1, Fga, Apob, Ang, Anxa13, Sat2, Sfrp5, A1cf, Apoa1, Bnip3, Fgl1, Pah, Serpina6, Apoa2, Azgp1, Fgg, Apoc3, Defb1, Tm4sf4, Gc, Ambp, Orm1, Ttr, Hal, Ass1, Hmgcs1, Acss2, Tm7sf2, Sec16b, Slbp, Rnd3, Bche, Ghr, Aldh6a1, Masp2, Hamp, Glul, Acly, Asl, Tmem97, Cp, Slpi, Acat3, Tm4sf5, Msmo1, Lepr, Rcan1, Ar, Plin1, Rpp25l, Hsd11b1, Apom, Tkfc, G0s2, Pon3, Ttc36, Golt1a, Fst, Mcc, Aqp9, Gsta1, Nnt, Saa4, Mrps18c, Ociad1, Apoa5, Entpd5, C4b, Eid2, Atic, Serpinh1, Samd5, Grb14, Abcd3, Rhob, Epb41l4b, Gpat4, Sptbn1, Sdc2, Phlda1, Wtap, Acadm*
Kupffer_cells	*Tnf, Mpo, Vsig4, Prok2, Stard5, Msr1, Tlr9, Ppara, Trem1, Slc11a1, Tlr4, Mmp13, Cd14, Vdr, Chit1, Osm, Ppard, Clec4f, Adgre1, Il1b, Slc40a1, Timd4, Ifi204, Slc15a3, Dnase1l3, Marco, Hfe, Cd38, Cd163, Ccr5, Clec1b, Clec4g, Gpihbp1, Folr2, Pltp, Irf7, Spic, Csf1r, C1qc, C1qa, C1qb, Clec4e*
Macrophages	*Itgal, Itgam, Cd14, Fut4, Cd33, Cd80, Cd86, Cd163, Ccr5, Tlr2, Tlr4, Adgre1, Gpr34, Trem2, Fabp4, S100a8, Cpm, Chit1, F13a1, Cx3cr1, Cxcl16, Tgfbr1, Slamf9, Scimp, Lilra5, Cd83, C3ar1, Stab1, Mrc1, Parp14, Fgd2, Rab7b, Rbpj, Slco2b1, Naaa, Egln3, Fgl2, Gpnmb, Clec4d, Adam8, Arl11, Mmp12, Lyve1, Pltp, Vsig4, Ms4a4a, Fpr1, Cd180, Gdf15, Rab20, Hfe, Tnf, Ccr2, Snx20, Fmnl1, Gpr132, Slamf7, Nceh1, Ccl24, C5ar1, Cd300a, Ccl7, Ccl2, Il1b, Irf5, Ahr, Myo1g, Dusp5, Gpr171, Ccr7, Dnase1l3, Cxcl1, Samsn1, Nr4a3, Ccl22, S100a4, Mmp9, Hilpda, Nrp2, Slc37a2, Ctsk, Cd36, Lpcat2, Hpgds, Ifnar2, Ms4a7, Slc11a1, Hpgd, Ccl3, Clec7a, Cd5l,*

	Ccl5, Cyth4, Cd3e, Cd19, Cd74, Csf1r, Lgals3, Cd68, Ucp2, Treml4, Fgr, Cybb, Cd200, Cd200r1, Gata6, Itgax, Pparg, Tyrobp, Rgs1, Dab2, P2ry6, Maf, Aif1, Clec10a, Adgre5, Slc15a3, Cyp27a1, Slc7a7, Runx3, Syk
Mast_cells	*Kit, Enpp3, Il2ra, Il17a, Crh, Hsd11b1, Cd274, Cxcr2, Cxcr4, Ccr3, Ccr5, Ltc4s, Gpr34, Mgst2, Ache, Cpa3, Cpa1, Cma1, Hnmt, Cfp, Rab27b, Sla, Il1rl1, Gnai1, Cfd, Osbpl8, Adora3, Hs3st1, Csf2rb2, Dapp1, Slc6a4, Maob, Csrnp1, Cyp11a1, Rgs1, Plek, Hdc, Tpsb2, Tph1, Cd55, Rgs13, Tpsg1, Socs1, Btk, Il4, Ms4a2, Vwa5a, Fcer1a, Mcemp1, Milr1, Ccl2, Creb3l1, Ednra, Hs6st2, Kcne3, Meis2, Mrgprb1, Plau, Adamts9, Chst1, Cobl, Ctsg, Dnm3, Grik2, Hpgds, Hs3st3a1, Papss2, Pcbd1, Pcp4l1, Rnf128, Slc29a1, Slc45a3, Stard13, A4galt, Acer3, Armcx3, Atl2, Bbs10, Bmpr2, C2, Ccdc141, Ccl7, Cdc42bpa, Cdh9, Copz2, Zdhhc13, Unc13b, Trank1, Tiam2, Tcf7l1, Stk32b, St6galnac3, Socs2, Spred1, Smpx, Smarca1, Slc7a5, Slc4a4, Slc31a2, Sgce, Ror1, Rnf180, Rbfox1, Rapgef2, Pde1c, Pcdh7, Optn, Oaf, Neo1, Ndst2, Nceh1, Mlph, Mitf, Mfge8, Lsamp, Lrrc66, Lima1, Krt4, Kdelr3, Ids, Hspa13, Hs2st1, Gpm6a, Gp1ba, Gnaz, Ext1, Esyt3, Eno2, Enah, Dusp1, Dgki, Ddc, Dclk3*
Megakaryocytes	*Itga2b, Gp1ba, Itgb3, Mpl, Lat, Plek, S100a9, S100a8, Pf4, F13a1, Cd9, Epor, Cxcr4, Gp5, Cxcr1, Cxcr2, Slamf1, Pde6b, Matk, Spon2, Tlr9, Kif5b, Gata1, Fli1, Prox1, Syp, Tspan9, Rgs18, Treml1, Gata2, Vwf, Alox12, Selp, Nfatc1, Cib1, Plk3, Mafg, Il2ra, Pecam1, Pparg, Il6, Il21r, Lrp1, Ache, Vegfa, Tgfb1, Gp9*
Monocytes	*Rgs1, Cd7, Tet2, Cd40, Dysf, Cmklr1, Mefv, Hck, Padi4, Ghsr, Itgax, Sele, Tlr4, Ar, Cxcr4, Cd86, Ccr2, Tnfrsf14, Mgmt, Cd14, Cd33, Itgam, Ace, Fut4, Icam1, Sell, Cd163, Fcgr2b, Acp5, Mrc1, Tnf, Plau, Irf7, Plscr1, Mx2, Il1rn, Ifit1bl1, Ido1, Ifit3, Tnfsf10, Cxcl10, S100a9, S100a8, S100a4, Clec7a, Csf3r, Ifi204, Zfp36l2, Lta4h, Clec12a, Cd48, Prtn3, Vcan, Ifitm3, Fn1, Adgre1, Cd44, Csf1r, Cx3cr1, Itgal, Pecam1, Ptprc, Spn, Psap, Rhoc, Pilra, Nfkbiz, Naaa, Ly6e, Lyn, Ms4a7, Cebpb, Cd68, Ifi30, Rgs2, Lst1, Spi1, Tymp, Fgl2, Pycard, Lyst, Ccl3, Il1b, Cfp, Cd36*
Myeloid-derived_suppressor_cells	*Itgam, Fut4, Cd33, Ccr2, Icam1, Cd80, Cxcr1, Adgre1, S100a9, S100a4, S100a8*
Natural_killer_T_cells	*Il12rb2, Il17ra, Il12rb1, Klrb1a, Rora, Itgb2, Egr1, Map3k8, Nr4a1, Gzmb, Tnfrsf8, Prf1, Il2rb, Zbtb16, Tbx21, Gata3, Ncam1, S1pr1, Styk1, Tcf7, Slamf7, Cxcr4, Cd44*
Neutrophils	*Mme, Itgam, Itgax, Cd14, Fut4, Pecam1, Cd33, Sell, C5ar1, Cxcr1, Cxcr2, Tlr2, Mylk, S100a9, Mpo, Ceacam1, Crp, Lcn2, Defa17, Ltf, Elane, S100a8, Il1b, Cxcl1, Hp, Ccl3, Hdc, Mmp8, Lrg1, Osm, Mmp9, Pilra, Clec4d, Clec4e, Asprv1, Ccrl2, Ccr1, Ncf1, Trem1, Sorl1, Arg2, Bst1, Il1r2, Cfp, Adam8, Cd177,*

	Ptgs2, Oas3, Prtn3, Ctsg, Camp, Slc1a5, Snx20, Adpgk, Pstpip1, Lyst, Dock8, S100a4, Nlrp3, Csf3r
NK_cells	*Itgam, Itgax, Cd69, Klrd1, Il2rb, Klrb1a, Cd244a, Klrk1, Slamf7, Ncr1, Slamf6, Kit, Cd27, Klrc1, Nkg7, Gzmm, Ctsw, Hmbox1, Ahr, Prf1, Ccl4, Sema6d, Fhl2, Cd2, Cd7, Cd3g, Cd33, Dpp4, Lat, Pcdh15, Ccl5, Gzma, Gzmb, Cma1, Dusp2, Txk, Dok2, Cst7, Hsd11b1, Samd3, Tbx21, Chsy1, Cxcr4, Il18r1, Serpinb9, Dock2, Sh2d2a, S100a4, Xcl1, Gzmk, Ifng, Ccl3, Csf2, Il2rg, Tgfb1, Kir3dl2, Klrg1, Adamts14, Itga2, Styk1, Clec2e, Cd247, Zbtb16, Spon2, Hopx, Cd8a*
Nuocytes	*Icos, Il1rl1, Rora, Il17rb, Il7r, Crlf2, Arg1, Gata3, Ptgds, Bcl11b, Il5, Il13*
Plasma_cells	*Sdc1, Irf4, Cd38, Prdm1, Cd93, Cd27, Enpp1, Klf4, Ms4a4a, Pias1, Cycs, Diablo, Htra2, Ms4a1, Cd9, Cd19, Cd37, Cd40, Cd74, Cxcr4, Gzmb, Tnfrsf17, Tnfrsf13b, Fcgr2b, Ccr10, Abcb9, Ampd1, Angpt4, Cd79a, Dennd5b, Eaf2, Gng7, Gpr25, Ighe, Ighm, Igkc, Kcna3, Kcng2, Lime1, Manea, Mast1, Mroh7, Mzb1, P2rx5, Pax7, Pdk1, Pnoc, Rasgrp3, Rgs13, Sik1, Spag4, St6galnac4, Tgm5, Tmem156, Zbp1, Ighg1, Iglc1, Jchain, Ssr4, Il16, Bhlha15, Ppib*
Plasmacytoid_dendritic_cells	*Cd4, Cd8a, Cd40, Cd80, Cd83, Cd86, Il3ra, Ccr7, Cxcr3, Tradd, Arhgap9, Lamp5, Gpr183, Map3k2, Tnfsf13b, Ptcra, Ern1, Etv6, Map3k8, Spib, Gzmb, Cxcr4, Pacsin1, Tlr7, Tlr9, Irf7, Irf8, Igll1, Cux2, Mctp2, Klk1, Blnk, Grm8, Prkca, Cdh1, Il17rb, Card6, Clec4g, S100a8, Jchain, Serpinf1, Itm2c, Tcf4, Bcl11a, Mzb1, Derl3, Zfat, Nrp1, Slc15a4, Ccr9, Irf5, Bst2*
Red_pulp_macrophages	*Hmox1, Treml4, Cd86, Nr1h3, Slc11a1, Adgre1, Mertk, Ccr3, Spic, Slc40a1, Vcam1, Siglec1*
T_cells	*Cd3d, Cd3g, Cd3e, Hopx, Ccl3, Ccl4, Syt3, Notch3, Sema6d, Dkk3, Ifit3, Cerk, Pmch, Cd4, Ltb, Cxcr6, Il7r, Satb1, Lef1, Itk, Ptprcap, Gem, Cd7, Maff, Tgif1, Rora, Tnfaip3, Crem, Pxdc1, Nabp1, Fam110a, Cd69, Cxcr4, Trbc1, Bcl2, Cytl1, Cd2, Ctsw, Ptpn22, Txk, Gdpd3, Traf1, Il2ra, Batf3, Lag3, Gzmk, Gzmb, Sh2d1a, Myo1g, Fmnl1, S1pr4, Cd247, Gimap5, Cd28, Cd160, Rhoh, Klrb1a, Ccr2, Il2rb, Mbd2, Icos, Il18r1, Tnfrsf4, Ccl20, Clec2e, Cd8a, Cd6, S100a4, Ccl5, Lck, Cd81, Thy1, Lat, Skap1, Tcf7, Ccr7, Myb, Gzma, Junb, Dusp2, Ifng, Cd52, Braf*
T_cytotoxic_cells	*Cd2, Cd27, Cd28, Cd5, Cd69, Cd8a, Gzmb*
T_follicular_helper_cells	*Bcl6, Cd3d, Cd4, Cd84, Slamf1, Cd40lg, Cxcr5, Tnfsf4, Icos, Pdcd1, Cd200, P2rx7*
T_helper_cells	*Ifng, Il2, Tbx21, Dpp4, Klrd1, Cxcr3, Ccr1, Ccr5, Tnfsf11, Havcr2, Il18r1, Ltbr, Tnf, Lta, Gata3, Csf2, Il4, Il5, Il6, Il10, Il13, Ccr8, Cxcr4, Ccr3, Ccr7, Icos, Ptgdr2, Havcr1, Il1r2, Irf4, Stat6, Il17a, Il17f, Il21, Il22b, Rora, Rorc, Cd38, Klrb1a, Ahr, Ccr10, Cd3d, Cd4, Ccr4, Ccr6, Il9, Cd2, Cd28, Cd5, Il1rl1, Foxp3, Cd3g, Tnfrsf4, Cd40lg, Il17rb, Hpgds, Nfatc2, Nfkb1, Nr3c1, Pparg*
T_memory_cells	*Sell, Ccr7, Cd4, Ccr5, Cd69, Itgb5, Bcl2, Grap2, Tbc1d10c, Cytip, Kpna4, Cd247, Epsti1, Apbb1ip, Cd27, Rhoh, Gimap1, Acp5, Ikzf1, Sp100, Dgka, Il7r, Itgb7, Nkg7, Cd28, Bin2,*

	Rapgef6, Arhgef1, S100a9, Arhgap15, Cd3e, Skap1, Gimap4, Cd2, Ptprc, Lat, Tcf7, Lef1, Arhgap45, Satb1, Ptprcap, S100a8, Ltb, Lck, Trbc1, Cd3g, Cd3d, Cd7, Cxcr6, Icos, Ccr6, Itgae, Enpp1
T_regulatory_cells	*Foxp3, Ikzf2, Cd4, Il2ra, Entpd1, Sell, Nt5e, Itgae, Tnfrsf4, Ctla4, Ccr4, Lag3, Izumo1r, Cngb1, Tnfrsf18, Maf, Il10, Il1r1, Itgb8, Lrrc32, Folr1*

Supplementary Table 3. LAM 22-gene signature

**Gene**
Trem2
Gpnmb
Cd63
Lgals3
Cd9
Fabp5
Cd36
Lpl
Atp6v0d2
Slc37a2
Mmp12
Tnip3
Akr1b8
Actn1
Gngt2
Dhrs9
Cd300lb
Plk2
Fblim1
Alas1
Msmo1
Ggta1

Supplementary Table 4. Pairwise comparison of D4 versus D7 cDC1 subclusters. Significant DEGs (adjusted p-value < 0.05) shown.

Gene	p_val	avg_log2FC	pct.1	pct.2	p_val_adj
Slc25a33	2.30E-08	4.304	0.443	0.033	3.14E-04
Il1b	1.55E-08	3.110	0.774	0.475	2.12E-04
Dusp2	9.13E-07	2.478	0.575	0.23	1.25E-02
Arl13b	1.82E-06	2.416	0.5	0.131	2.49E-02
Mpp7	1.70E-07	2.170	0.774	0.459	2.32E-03
Csrnp1	5.17E-07	2.090	0.547	0.148	7.06E-03
Tuba1a	8.11E-07	2.072	0.632	0.295	1.11E-02
Cd44	1.21E-08	1.884	0.915	0.77	1.65E-04
Tuba1c	1.33E-08	1.879	0.698	0.262	1.82E-04
Fbxo34	1.10E-06	1.788	0.557	0.164	1.50E-02
1700016P03Rik	1.03E-08	1.718	0.764	0.262	1.41E-04
Csf2rb	9.56E-07	1.716	0.745	0.426	1.31E-02
Dot1l	5.48E-08	1.709	0.736	0.328	7.49E-04
Taf7	1.02E-06	1.688	0.566	0.164	1.40E-02
Gpr65	3.30E-07	1.688	0.821	0.59	4.52E-03
Trib1	1.77E-07	1.682	0.717	0.361	2.42E-03
Tubb6	2.42E-06	1.651	0.651	0.311	3.31E-02
Crem	1.71E-07	1.640	0.726	0.295	2.34E-03
Lrrc8c	1.33E-08	1.617	0.906	0.738	1.82E-04
Got1	1.15E-06	1.603	0.755	0.443	1.57E-02
Lncpint	1.11E-07	1.556	0.877	0.623	1.51E-03
Atp6v0d1	7.59E-07	1.555	0.689	0.361	1.04E-02
Hspd1	3.24E-06	1.547	0.915	0.738	4.42E-02
Arl5b	8.69E-07	1.540	0.66	0.311	1.19E-02
Gm26532	8.24E-07	1.517	0.679	0.311	1.13E-02
Dnajb1	1.33E-06	1.516	0.887	0.689	1.82E-02
Snhg12	1.57E-06	1.502	0.708	0.344	2.15E-02
Ext1	1.12E-07	1.492	0.906	0.738	1.53E-03
Nr4a3	1.83E-07	1.459	0.811	0.443	2.51E-03
Gm47283	5.95E-07	1.447	0.868	0.672	8.14E-03
Tob2	1.01E-07	1.431	0.83	0.459	1.38E-03
Gpd2	9.19E-09	1.417	0.887	0.557	1.26E-04
Dusp1	2.53E-08	1.409	0.981	0.82	3.46E-04
Erlin1	1.28E-08	1.392	0.858	0.475	1.75E-04
Gpcpd1	1.15E-06	1.366	0.943	0.82	1.58E-02
Nr4a2	7.63E-09	1.339	0.943	0.607	1.04E-04
Hspa1b	2.29E-07	1.323	0.934	0.852	3.12E-03
Hspa9	2.59E-07	1.295	0.783	0.377	3.54E-03
Azin1	2.90E-06	1.288	0.717	0.41	3.97E-02
Btaf1	2.13E-06	1.285	0.83	0.557	2.92E-02
Lmnb1	1.97E-07	1.253	0.84	0.59	2.70E-03

Fyn	1.11E-08	1.231	0.934	0.885	1.51E-04
Brd2	3.15E-06	1.220	0.858	0.639	4.30E-02
Ier5	1.87E-07	1.203	0.962	0.869	2.55E-03
Ddx21	1.22E-06	1.173	0.821	0.574	1.66E-02
Nudc	1.39E-06	1.146	0.858	0.656	1.90E-02
Skil	4.96E-08	1.132	0.991	0.82	6.78E-04
Ago2	7.82E-07	1.130	0.868	0.508	1.07E-02
Stip1	2.80E-06	1.122	0.708	0.295	3.83E-02
Jun	6.76E-08	1.107	0.972	0.934	9.24E-04
Cacybp	3.51E-06	1.084	0.849	0.623	4.80E-02
Litaf	2.05E-06	1.034	0.868	0.689	2.81E-02
Picalm	1.70E-07	1.004	0.991	0.951	2.33E-03
Ubxn4	1.53E-06	0.958	0.896	0.705	2.10E-02
Hspe1	1.55E-06	0.945	1	1	2.12E-02
Eif5	1.03E-06	0.869	0.962	0.869	1.41E-02
Ncl	6.65E-07	0.830	0.991	0.951	9.09E-03
Rel	2.29E-06	0.802	0.981	0.918	3.13E-02
Pafah1b1	5.36E-07	0.791	0.972	0.836	7.32E-03
Srsf7	4.09E-07	0.771	0.981	0.836	5.59E-03
Eif3c	1.21E-06	0.744	0.962	0.852	1.65E-02
Rps27l	4.55E-07	0.724	0.962	0.951	6.22E-03
Cd83	4.64E-07	0.719	1	0.918	6.34E-03
Sub1	1.59E-08	0.672	1	1	2.17E-04
Calm1	1.25E-06	0.631	1	1	1.71E-02
Chchd2	3.01E-07	0.616	0.991	1	4.11E-03
Ywhaz	2.08E-06	0.614	1	0.984	2.85E-02
Ddx5	1.04E-06	0.543	1	1	1.42E-02
Rbm39	8.46E-07	0.512	1	1	1.16E-02
Ppt1	4.25E-07	-0.555	1	1	5.82E-03
Itm2b	2.42E-07	-0.566	1	1	3.30E-03
Clic1	1.90E-06	-0.622	0.981	1	2.60E-02
Ly6e	9.38E-08	-0.636	0.991	0.984	1.28E-03
Pid1	7.90E-07	-0.811	0.906	0.984	1.08E-02
Dpp4	2.50E-06	-0.857	0.745	0.967	3.41E-02
Pip4k2a	5.81E-07	-0.881	0.925	1	7.94E-03
BC028528	2.83E-06	-0.883	0.792	0.934	3.87E-02
Mef2a	1.68E-06	-0.884	0.811	0.951	2.30E-02
Fmnl2	1.16E-07	-0.888	0.943	0.984	1.58E-03
Slamf7	7.55E-08	-0.910	0.83	1	1.03E-03
Rnase6	3.24E-08	-0.975	0.745	0.951	4.43E-04
Ly86	8.36E-08	-0.978	0.792	1	1.14E-03
Fgd2	2.46E-08	-1.028	0.745	0.951	3.36E-04
Cbl	3.12E-07	-1.076	0.67	0.902	4.27E-03
Plin2	8.54E-09	-1.078	0.726	0.951	1.17E-04
Slfn2	2.04E-06	-1.087	0.509	0.82	2.79E-02
Lrch1	1.48E-06	-1.131	0.632	0.82	2.03E-02

Ncoa1	1.60E-06	-1.146	0.519	0.902	2.18E-02
Arhgap5	1.35E-08	-1.215	0.679	0.934	1.84E-04
Ppm1h	1.46E-06	-1.216	0.613	0.82	1.99E-02
Srgap3	1.06E-07	-1.230	0.651	0.885	1.44E-03
Fchsd2	2.59E-07	-1.344	0.764	0.869	3.53E-03
Ikzf1	6.48E-08	-1.382	0.462	0.82	8.85E-04
Nrg2	1.48E-06	-1.390	0.311	0.672	2.02E-02
Supt20	1.84E-06	-1.426	0.585	0.82	2.51E-02
Txnip	7.13E-07	-1.438	0.368	0.738	9.75E-03
Gm36723	9.73E-08	-1.469	0.755	0.918	1.33E-03
Zfp710	1.14E-07	-1.492	0.557	0.803	1.55E-03
Cdc14a	2.49E-06	-1.512	0.472	0.738	3.41E-02
Plcl2	2.65E-07	-1.589	0.302	0.672	3.62E-03
Rgs18	1.61E-06	-1.605	0.17	0.525	2.20E-02
Fkbp5	1.90E-06	-1.627	0.292	0.607	2.60E-02
Rmdn1	4.60E-07	-1.864	0.179	0.541	6.29E-03
Slc12a9	3.25E-06	-1.878	0.236	0.557	4.44E-02
Pik3ip1	2.01E-06	-1.890	0.132	0.459	2.75E-02
Sult1a1	4.56E-07	-1.967	0.311	0.623	6.23E-03
Zfp740	9.19E-07	-1.995	0.151	0.492	1.26E-02
Gm35188	2.43E-06	-2.020	0.123	0.443	3.32E-02
Sh2d3c	7.35E-08	-2.104	0.17	0.541	1.00E-03
Fbxl20	1.88E-07	-2.352	0.113	0.459	2.57E-03
Arl11	2.17E-06	-2.868	0.038	0.295	2.97E-02

Supplementary Table 5. Pairwise comparison of GAN-Adj.NT versus GAN cDC2 subpopulations. Significant DEGs (adjusted p-value < 0.05) shown.

Gene	p_val	avg_log2FC	pct.1	pct.2	p_val_adj
Slc25a33	8.87E-09	2.161	0.272	0.063	1.21E-04
Marcksl1	3.36E-06	1.589	0.592	0.464	4.59E-02
Sdc4	3.34E-08	1.582	0.35	0.126	4.56E-04
Arid5a	4.45E-08	1.310	0.447	0.198	6.08E-04
Sik3	9.07E-07	1.258	0.558	0.348	1.24E-02
Nfkbiz	3.49E-08	1.192	0.786	0.599	4.78E-04
Tob2	1.96E-08	1.126	0.641	0.406	2.67E-04
Sde2	8.27E-07	1.029	0.612	0.415	1.13E-02
Rilpl2	2.92E-07	1.007	0.655	0.444	3.99E-03
Fosl2	1.22E-06	1.007	0.578	0.357	1.66E-02
Zc3hav1	2.16E-06	0.950	0.772	0.628	2.95E-02
Relb	1.50E-08	0.939	0.587	0.295	2.05E-04
Sqstm1	1.81E-07	0.850	0.859	0.7	2.47E-03
Grasp	9.12E-07	0.824	0.675	0.459	1.25E-02
Ezr	3.43E-06	0.820	0.743	0.589	4.69E-02
Litaf	2.93E-08	0.813	0.83	0.662	4.00E-04
Dnajb1	1.62E-06	0.788	0.825	0.643	2.22E-02
Hspa1a	5.05E-08	0.785	0.966	0.913	6.90E-04
Egr1	4.47E-07	0.773	0.879	0.802	6.11E-03
Dusp2	2.78E-06	0.754	0.636	0.391	3.80E-02
Tiparp	1.19E-06	0.746	0.68	0.473	1.63E-02
Tagln2	8.98E-08	0.735	0.947	0.826	1.23E-03
Nr4a1	8.24E-08	0.704	0.927	0.836	1.13E-03
Ccnl1	1.40E-07	0.673	0.859	0.734	1.91E-03
Pnrc1	2.04E-08	0.616	0.942	0.918	2.79E-04
Hspa5	1.30E-07	0.598	0.985	0.937	1.77E-03
Pim1	6.37E-07	0.590	0.922	0.845	8.71E-03
Hsp90aa1	2.46E-07	0.588	0.99	0.99	3.36E-03
Zfand5	1.81E-08	0.581	0.951	0.845	2.48E-04
Hsp90ab1	3.01E-08	0.432	1	1	4.12E-04
Btg2	1.11E-06	0.415	0.981	0.937	1.52E-02
Hspa8	3.10E-06	0.282	1	1	4.23E-02
Tmsb4x	5.31E-07	-0.258	1	1	7.25E-03
Itm2b	4.86E-08	-0.385	1	1	6.65E-04
Otulinl	9.63E-07	-0.491	0.874	0.947	1.32E-02
Cd48	2.71E-07	-0.537	0.806	0.884	3.70E-03
Slamf7	3.46E-06	-0.617	0.82	0.879	4.73E-02
Ap1s2	5.72E-08	-0.957	0.714	0.831	7.82E-04
Id2	1.94E-08	-1.214	0.602	0.763	2.66E-04

Supplementary Table 6. Curated B cell signatures

B cell signature	Genes
ABC signature in Malaria [10]	*Itgax, Rtn4r, Il10, Cd274, Il21r, Cd84, Pdcd1, Six3, Il12rb2, Slamf7, Grn, Fas, Pdcd1lg2, Boc, Arhgef5, Tbx15, Il10ra, Clec2e, Pdgfa, Plxnc1, Chrna7, Cd86, Cmtm3, Tox2, Cd200r1, Sema7a, Aicda, Hoxa10, Xpot, Itgb2, Tln1, Irx6, Sox5, Myo9b, Gdi2, E2f7, Hoxb6, Il2rb, Aire, Cmtm6, Cd72, Lamp1, Ebi3, Hoxa3, Satb2, Notch1, Slamf1, Ly6e, Cd164, Pou3f3, Pirb, Cd22, Lilrb4b, Cd72, Cxcr3, Itgax, Ccr6, Pdcd1, Fcrl5, Pirb, Pirb, Cd33, Cd22, Cd72, Cd200, Cd200r1, Itgax, Cd19, Ms4a1, Cd86, Il21r, Batf, Foxo1, Ikzf2, Nfatc2, Maf, Nfil3, Nr4a3, Tbx21, Zeb2, Crem, Atf3, Zbtb32, Jazf1, Litaf, Hopx, Mybl1, Tox, Prdm15, Slc1a6, Hck, Rhoc, Adamts13, Ppl, Iqcg, Timm13, Gpatch2, Nelfe, Ccr5, Slpi, Rhobtb2, Il6, Blvra, Cinp, Arhgef3, Cdca7, Rnf38, I830077J02Rik, Themis2, Nfkb1, Smco4, Ccdc167, Abhd14b, Map3k14, Ccdc115, Gpr34, Tbx21, Il27ra, Gpr82, Trib2, Adam8, Vps33a, Rsl24d1, Repin1, Prmt2, Slc25a1, Casp4, Eea1, Dusp4, Eif3i, Ccdc141, Tnfsf13, Tnfrsf13b, Slamf7, Cd19, Cd84, Cd86, Tnfrsf1a, Ifnar2, Il7, Il10ra, Ifnlr1, Itgb1, Itgb2, Itgb7, Itgax, Cd72, Fcgr2b, Fcrl5, Pirb, Pirb, Pirb, Cd33, Siglecg, Lcp2, Syk, Hck, Prkch, Ptprs, Ptpn2, Ptpn12, Ptpn22, Fas, Casp1, Casp9, Card11, Nlrc4, Gas7, Cdkn1a, Bcl11b, Egr2, Hoxa3, Hoxb7, Hoxc4, Nfatc2, Rnf144a, Sox4, Tbx21, Tox, Zeb2*
Pre-GC signature [11]	*Ccnd2, Psme2, Fcer2a, Ebi3, Ddx21, Syngr2, Hsp90ab1, Nop16, Cd83, Ncl, Eif5a, Dnph1, Gpx4, Myc, Ifitm2, Cdkn1a, Nhp2, Nfkbid, Nme2, Nolc1, Parvb, Lilra6, Rpl22l1, Eno1, H2-Aa, Traf4, Pa2g4, Srm, Bhlhe40, Mat2a, Fkbp4, Prmt1, Calr, Batf, Mrto4, Prdx1, Ifrd2, Acp5, Dusp2, Atic, Ebna1bp2, Irf4, Fabp5, Ybx1, Paics, Gm2a*
LZ signature [12]	*Ebi3, Egr3, Prdm1, Il4i1b, Dusp2, Egr2, Slamf1, Ccl22, Cd83, Hivep3, Akap5, Irf4, Myc, Sema7a, Nfkb2, Nfkbie, Lrrk2, Batf, Traf4, Ccr6, Il27ra, Slc39a14, Tnfaip3, Icam1, Kynu, Srm, Ipo4, Ccdc86, Samsn1, Egr1, Iqsec1, Bhlhe40, Nfkbia, Snx11, Ciita, Nab2, Slc2a3, Mettl1, Abhd6, Zc3h12a, Rcn1, Lmo2, Ly75, Nop16, Aldoc, Mybbp1a, Map3k8, Plek, Dctd, Gpr183, Nfkbid, Ier5, Ddit4, Rrp15, Dusp10, Ifrd2, Mfsd2a, Junb, Bmi1, Rpp40, Lgals9, Lta, Rsad1, Vasp, Gnl3, Ier2, Cd72, Kctd12, Tnf, Ptpn6, Prmt3, Syngr2, Pdcd4, Ifi30, Pprc1, Tsen2, Fasn, Tlcd1, Ddx21, Slc23a2, Abcb4, Il18r1, Utp15, Rrp12, Hsp90b1, Rhof, Adcy3, Cln5, Bcl3, Il2rb, Polr3e, Rilpl2, Stat6, Hspa5, Abcc4, Ctsh, Crem, Akap1, Ankrd33b, Ccr7, Ide, Cdk5r1, Rgs1, Il6st, Nat10, Tspan33, Gatm, Ralgds, Cd40, Nop2, Ppargc1b, Cyfip1, Stat1, Cyth4, Mdn1, Birc3, Irf2bp2, Plk3, Grn, Fyco1, Tmem109, Unc119, Calr, Mapkapk3, Tpp1, Pdia6, Pou2f2, Il2rg, Dhcr7, Nolc1, Nr4a1, Ampd3, Extl2, Zbtb8a, Apex1, Alcam, Relb, Bid, Psme2, Aff1, Ctsc, Per1, Jarid2, Arhgap24, Aprt, Fam162a, Sub1, Cd86, Kdm6b, Gpr65, Phlda1, Zfp36l1, Tnfrsf18, Slc15a3, Ccdc107, Lck, Manf, Nop56, Erp44, Znhit6, Insig1, Dhodh, Tapbp, Psmg4, Top1mt, Fyn, Zmiz2, Pnpo, Pes1, Ltv1, Ndufaf4, Midn, Chd1, Cd82, Reep3, Clcf1, Hspe1, Naf1, Trnt1, Urb1, Orai1, Slc25a19, Tmc6, Ssr4, Kat2a, Pdia4,*

	Arid3b, Invs, Cd320, Cad, Cr2, Dctpp1, Tbl3, Runx3, Mast3, St3gal5, Nhp2, Mreg, Evl, Ctsa, Rrp1b, Nol10, Actr3b, Gpr132, Akt1s1, Arid5a, Tnfsf9, Heg1, Rfx5, Slc3a2, Per2, Lpin1, Trmt61a, Tfrc, Grwd1, Nab1, Aldh18a1, Ankrd37, Slc2a6, Ikbke, Sipa1, Hook2, Eef1e1, Fabp5, Sigmar1, Rogdi, Tet2, Chchd4, Armcx3, Plscr1, Pex10, Klf10, G6pc3, Zswim6, Cyba, Mtap, Tspan31, Dusp7, Slc16a6, Wdfy4, Creld2, Hvcn1, Noc2l, Nbeal2, Sdc4, Mogs, Phf1, Ppa1, Ube2d1, Immp2l, Trappc5, Ptpre, Rasal3, Txlna, Riok2, Pigyl, Ppp1r14b, Polr1a, Inf2, Rel, Tnfrsf13c, Traf3, Nfkbiz, Soat1, Wdr89
DZ signature [12]	*Gas2l3, Psrc1, Aicda, Kif20a, Nek2, Cenpe, Cdc20, Cenpf, Gpsm2, Aurka, Cxcr4, Cdc25c, Aspm, Ccnb2, Hmmr, Cdc25b, Dlgap5, Cenpa, Ifi205, Fam83d, Birc5, Ube2c, Anln, Cdkn3, Kif23, Prc1, Rell1, Hjurp, Ccnb1, Ect2, Pif1, Cdca8, Tpx2, Hspa2, Cdca3, Plk1, Depdc1b, Mnd1, Tacc3, Troap, Ube2s, Nuf2, Hmgb3, Top2a, Arhgap11a, Cdca2, Rrm2b, Ckap2l, Rdm1, Ccnf, Polh, Ndc80, Spc24, Cep55, Cep70, Espl1, Spag5, Bub1, Ttk, Kif18a, Ncapg, Kif11, Kif18b, Aurkb, Kif2c, Kifc1, Ift57, Ccna2, Cdk1, Tmed6, Ckap2, Oip5, Ncaph, Ncapd2, Eri2, Foxp1, Racgap1, Mki67, Nusap1, Ska1, Lig4, Cit, Dnase1, Ckap5, Asf1a, Nfyb, Arhgap19, Ypel3, Hyls1, Pkp4, Incenp, Rassf6, Tcf3, Gnaz, G2e3, Gmfg, Ctnnal1, Poc1a, Fam81a, Psip1, Smarca4, Cep76, Cep135, Lpp, Bub1b, Neurl1b, Ccnd3, Bcl7a, Gtse1, Gkap1, Shcbp1, Kif20b, Syne2, Mybl1, Tram2, Suv39h1, Eif2ak3, Fam107b, Tcea1, Cenpl, Lrwd1, Kpna2, Phf3, Dhfr, Cdkl1, Rnf26, Ube2j1, Tgfbr1, Cdc27, Ccdc18, Rap1gap2, Calm3, Rbm38, Hp1bp3, Gfod1, Oaz2, Brwd1, Mad2l1, Kctd9, Cebpg, Tax1bp3, Bpgm, Klf6, Esco2, Nbr1, Arrdc3, Ccm2, Rere, Nde1, Xkrx, Dbf4, Ccng2, Clasp2, Dcaf12, Pola1, Cdkn2aipnl, Top1, Lin9, Rhoh, Ctdspl2, Traf5, Ube2g1*

Supplementary Table 7. Pairwise comparison of GAN versus LFD endothelial cells. Significant DEGs (adjusted p-value < 0.05) shown.

Gene	p_val	avg_log2FC	pct.1	pct.2	p_val_adj
Depp1	0.00E+00	6.622	0.693	0.03	0.00E+00
Nin	5.94E-09	0.663	0.384	0.257	1.01E-04
Gm26802	6.10E-09	-0.957	0.086	0.201	1.04E-04
Serpinb6a	6.18E-09	-0.353	0.853	0.863	1.05E-04
Prickle1	6.28E-09	-0.412	0.765	0.85	1.07E-04
Ttc8	6.36E-09	2.003	0.063	0.018	1.08E-04
Ophn1	6.38E-09	0.628	0.425	0.296	1.08E-04
Hspa1a	6.40E-09	-0.747	0.544	0.645	1.09E-04
Frzb	6.41E-09	4.106	0.017	0.001	1.09E-04
4833422C13Rik	6.47E-09	4.106	0.017	0.001	1.10E-04
Pcsk5	6.49E-09	4.734	0.024	0.002	1.10E-04
Itgb8	6.59E-09	3.428	0.024	0.002	1.12E-04
Ier5	6.70E-09	-0.605	0.488	0.601	1.14E-04
Bmx	7.00E-09	2.691	0.032	0.005	1.19E-04
Ywhab	7.10E-09	-0.338	0.821	0.871	1.20E-04
Arhgef40	7.19E-09	-1.298	0.082	0.191	1.22E-04
Nid2	7.38E-09	2.251	0.056	0.015	1.25E-04
9930111J21Rik2	7.62E-09	-0.938	0.257	0.383	1.29E-04
Hmg20a	8.28E-09	0.637	0.447	0.318	1.40E-04
Eci2	8.92E-09	0.722	0.311	0.195	1.51E-04
Xkr6	8.93E-09	0.808	0.337	0.222	1.52E-04
Slc26a10	8.96E-09	0.727	0.397	0.277	1.52E-04
Dand5	9.20E-09	-1.727	0.058	0.158	1.56E-04
Zfp91	9.41E-09	0.419	0.637	0.502	1.60E-04
Ifitm2	9.44E-09	-0.244	0.998	1	1.60E-04
Map3k1	9.56E-09	0.699	0.352	0.233	1.62E-04
Ndufb7	9.60E-09	-0.555	0.54	0.639	1.63E-04
Map4k4	9.72E-09	0.494	0.715	0.616	1.65E-04
Sept4	9.72E-09	0.812	0.272	0.163	1.65E-04
Tnrc6c	9.73E-09	0.551	0.473	0.343	1.65E-04
Atp6v0c	9.74E-09	-0.347	0.873	0.906	1.65E-04
Ybx1	9.76E-09	0.273	0.974	0.959	1.66E-04
Ei24	9.79E-09	0.654	0.343	0.221	1.66E-04
Zc3h12c	9.79E-09	1.053	0.166	0.082	1.66E-04
Serhl	1.02E-08	1.039	0.166	0.082	1.73E-04
Tnfsf10	1.03E-08	0.671	0.434	0.313	1.75E-04
Gm26532	1.04E-08	-1.072	0.158	0.28	1.77E-04
Fbxl7	1.05E-08	0.612	0.866	0.863	1.78E-04
Vps8	1.05E-08	0.649	0.356	0.232	1.78E-04
Selenoi	1.06E-08	1.044	0.173	0.087	1.80E-04
Pcnx	1.07E-08	0.579	0.49	0.365	1.81E-04

Tubb5	1.08E-08	0.381	0.832	0.767	1.83E-04
Knop1	1.09E-08	0.535	0.432	0.297	1.85E-04
Hyal2	1.09E-08	-0.490	0.587	0.677	1.85E-04
Fmn1	1.13E-08	0.761	0.397	0.277	1.92E-04
Nrxn3	1.15E-08	2.864	0.045	0.01	1.94E-04
Gm36756	1.17E-08	3.351	0.026	0.003	1.98E-04
Vps13c	1.20E-08	0.481	0.497	0.362	2.03E-04
Rnf19b	1.21E-08	-0.901	0.212	0.341	2.05E-04
Clint1	1.25E-08	0.546	0.555	0.423	2.12E-04
Svil	1.26E-08	0.931	0.35	0.239	2.14E-04
Selenok	1.27E-08	-0.324	0.806	0.877	2.16E-04
Sem1	1.28E-08	-0.286	0.894	0.937	2.18E-04
Rasgrp4	1.30E-08	2.307	0.045	0.01	2.20E-04
Dop1a	1.35E-08	0.812	0.253	0.147	2.28E-04
Clasp2	1.35E-08	0.612	0.492	0.366	2.29E-04
Hspa1b	1.37E-08	-0.214	0.413	0.579	2.32E-04
Gm4258	1.40E-08	1.405	0.127	0.057	2.38E-04
Plscr1	1.41E-08	0.966	0.16	0.078	2.39E-04
Plscr4	1.44E-08	0.530	0.562	0.438	2.44E-04
Lck	1.44E-08	-1.663	0.037	0.128	2.45E-04
Plk2	1.45E-08	0.368	0.788	0.669	2.47E-04
Ltc4s	1.49E-08	-1.075	0.149	0.267	2.53E-04
Ermp1	1.51E-08	0.955	0.194	0.103	2.57E-04
S1pr1	1.51E-08	0.440	0.674	0.55	2.57E-04
Stim2	1.59E-08	0.409	0.706	0.596	2.69E-04
Agpat5	1.59E-08	0.426	0.773	0.701	2.70E-04
Erg	1.61E-08	0.396	0.784	0.669	2.73E-04
Vamp8	1.69E-08	-0.641	0.374	0.505	2.86E-04
Bmp4	1.72E-08	2.476	0.048	0.011	2.92E-04
P2ry1	1.73E-08	-1.055	0.143	0.26	2.93E-04
H2-D1	1.78E-08	0.218	0.994	0.997	3.02E-04
Pgp	1.78E-08	0.717	0.324	0.207	3.02E-04
Gm41556	1.81E-08	-2.703	0.015	0.092	3.07E-04
Samd5	1.84E-08	3.271	0.032	0.005	3.12E-04
Fbxl17	1.88E-08	0.493	0.823	0.752	3.19E-04
Sec1	1.89E-08	2.521	0.035	0.006	3.20E-04
Hexa	1.90E-08	-0.580	0.436	0.55	3.23E-04
Gm26782	1.94E-08	1.579	0.073	0.024	3.30E-04
Aimp1	1.94E-08	0.558	0.449	0.318	3.30E-04
Stk3	1.95E-08	0.570	0.579	0.461	3.31E-04
Cdc42ep5	1.96E-08	0.616	0.32	0.2	3.33E-04
Zfp664	2.10E-08	0.603	0.339	0.215	3.56E-04
Ndufa6	2.12E-08	0.412	0.706	0.607	3.59E-04
Gm19461	2.13E-08	-2.584	0.011	0.085	3.61E-04
Large1	2.13E-08	0.681	0.505	0.383	3.61E-04
Ifit3b	2.14E-08	1.210	0.136	0.063	3.63E-04

Plp2	2.15E-08	2.095	0.056	0.015	3.66E-04
Cx3cr1	2.17E-08	-1.423	0.102	0.209	3.68E-04
Fosl2	2.17E-08	0.793	0.369	0.256	3.69E-04
Hmox1	2.18E-08	-0.707	0.374	0.492	3.70E-04
Gpatch8	2.20E-08	0.536	0.486	0.367	3.74E-04
Uqcrq	2.21E-08	-0.441	0.644	0.716	3.75E-04
Cox7a2	2.22E-08	-0.345	0.834	0.877	3.77E-04
Rc3h1	2.24E-08	0.496	0.6	0.467	3.81E-04
Kdelr2	2.27E-08	-0.478	0.629	0.708	3.85E-04
Pik3r3	2.34E-08	-0.989	0.205	0.325	3.97E-04
Arhgap35	2.34E-08	0.517	0.464	0.33	3.97E-04
Prcp	2.39E-08	0.803	0.378	0.266	4.06E-04
Cpd	2.53E-08	0.414	0.672	0.565	4.29E-04
Dmd	2.59E-08	0.672	0.419	0.305	4.40E-04
Cd55	2.62E-08	-0.224	0.94	0.975	4.45E-04
AV356131	2.66E-08	2.138	0.045	0.011	4.52E-04
Etv6	2.72E-08	0.615	0.603	0.491	4.62E-04
Gm11476	2.96E-08	-1.504	0.071	0.171	5.02E-04
Tgfbr3	2.97E-08	0.396	0.801	0.718	5.04E-04
Cd209b	2.98E-08	-2.844	0.015	0.09	5.05E-04
Hivep3	2.98E-08	0.510	0.663	0.542	5.05E-04
Serinc3	2.99E-08	0.209	1	0.995	5.08E-04
Gstm3	3.00E-08	1.766	0.024	0.003	5.10E-04
Plxna4os3	3.03E-08	3.222	0.024	0.003	5.14E-04
Jcad	3.05E-08	0.576	0.514	0.395	5.17E-04
Phlda1	3.07E-08	-1.069	0.104	0.216	5.21E-04
Dcaf5	3.08E-08	0.730	0.328	0.216	5.22E-04
Plet1os	3.14E-08	-0.581	0.272	0.41	5.33E-04
Zfp950	3.16E-08	0.981	0.177	0.092	5.37E-04
Ifi27	3.16E-08	-0.501	0.521	0.622	5.37E-04
Scd1	3.22E-08	1.064	0.13	0.059	5.46E-04
Cbfa2t2	3.23E-08	0.878	0.261	0.16	5.48E-04
Trip11	3.24E-08	0.431	0.512	0.371	5.49E-04
Akr1b8	3.33E-08	-0.704	0.408	0.506	5.66E-04
Churc1	3.39E-08	1.050	0.168	0.086	5.75E-04
Frs2	3.41E-08	0.623	0.32	0.202	5.79E-04
Vangl2	3.45E-08	1.365	0.097	0.038	5.85E-04
Ano6	3.56E-08	1.034	0.216	0.123	6.04E-04
Pde12	3.58E-08	-1.173	0.095	0.202	6.08E-04
Fut8	3.60E-08	0.799	0.294	0.186	6.10E-04
Cep350	3.66E-08	0.493	0.492	0.358	6.22E-04
Zfp36	3.70E-08	-0.301	0.631	0.746	6.28E-04
Ces2g	3.72E-08	-0.780	0.227	0.352	6.32E-04
Spidr	3.81E-08	0.838	0.253	0.152	6.46E-04
Kmt2d	3.85E-08	0.680	0.289	0.179	6.53E-04
Icam2	4.00E-08	0.423	0.728	0.605	6.79E-04

Xaf1	4.14E-08	0.570	0.382	0.255	7.02E-04
Dip2c	4.18E-08	0.592	0.579	0.474	7.09E-04
H3f3a	4.19E-08	0.258	0.95	0.942	7.11E-04
Sh3pxd2a	4.19E-08	0.861	0.317	0.208	7.11E-04
Ube2s	4.24E-08	-0.564	0.447	0.564	7.20E-04
Glp1r	4.29E-08	3.384	0.026	0.003	7.28E-04
Srsf5	4.31E-08	-0.361	0.637	0.727	7.31E-04
Ube2h	4.39E-08	0.311	0.739	0.638	7.44E-04
Mfap3	4.41E-08	0.735	0.285	0.178	7.48E-04
Atp11c	4.51E-08	0.547	0.551	0.427	7.65E-04
Efnb1	4.52E-08	0.919	0.428	0.325	7.67E-04
Gclm	4.66E-08	-0.779	0.207	0.336	7.90E-04
Milr1	4.68E-08	-1.215	0.063	0.161	7.94E-04
Tnik	4.75E-08	-0.514	0.374	0.509	8.06E-04
Cwf19l2	4.78E-08	0.647	0.374	0.254	8.12E-04
Sik2	4.82E-08	0.697	0.281	0.172	8.18E-04
Gm35533	4.90E-08	-0.284	0.259	0.397	8.31E-04
Gm10369	4.90E-08	1.387	0.086	0.032	8.32E-04
Mtch1	5.00E-08	-0.379	0.711	0.768	8.50E-04
Tcf7l2	5.02E-08	0.589	0.505	0.392	8.52E-04
H2-T23	5.10E-08	0.390	0.86	0.79	8.65E-04
Zyx	5.18E-08	0.744	0.309	0.2	8.79E-04
Lyplal1	5.21E-08	2.521	0.032	0.006	8.85E-04
Igsf3	5.25E-08	1.042	0.162	0.082	8.92E-04
Rhoj	5.27E-08	0.414	0.778	0.657	8.94E-04
Gm2000	5.28E-08	0.584	0.41	0.286	8.97E-04
Ppp1r12b	5.33E-08	0.783	0.248	0.148	9.04E-04
Amigo2	5.42E-08	1.045	0.192	0.105	9.20E-04
Upf3b	5.60E-08	0.676	0.302	0.193	9.50E-04
Nop58	5.63E-08	-0.784	0.227	0.349	9.56E-04
Lix1l	5.66E-08	0.717	0.281	0.175	9.61E-04
Cpne1	5.72E-08	0.504	0.421	0.289	9.71E-04
St3gal1	5.85E-08	0.580	0.473	0.354	9.94E-04
Rasgrf2	5.90E-08	-0.670	0.145	0.268	1.00E-03
Tspan12	6.01E-08	0.565	0.395	0.272	1.02E-03
Abhd18	6.13E-08	1.222	0.14	0.068	1.04E-03
Zfp277	6.36E-08	0.609	0.322	0.207	1.08E-03
S100a8	6.36E-08	-0.618	0.302	0.428	1.08E-03
Kif13a	6.40E-08	0.885	0.287	0.184	1.09E-03
Pcf11	6.50E-08	-0.678	0.328	0.445	1.10E-03
Fcho2	6.65E-08	0.390	0.844	0.814	1.13E-03
Nr1d1	6.73E-08	-0.658	0.311	0.434	1.14E-03
Adm	6.76E-08	0.986	0.363	0.259	1.15E-03
Mrpl48	6.83E-08	0.659	0.333	0.219	1.16E-03
Myc	6.84E-08	1.604	0.084	0.032	1.16E-03
Camk2d	7.03E-08	0.432	0.754	0.666	1.19E-03

Slmap	7.16E-08	0.430	0.618	0.498	1.21E-03
Slc24a5	7.24E-08	-0.779	0.21	0.331	1.23E-03
Uba52	7.27E-08	0.396	0.788	0.724	1.23E-03
Rbfox2	7.32E-08	0.482	0.605	0.484	1.24E-03
Flrt3	7.36E-08	3.714	0.022	0.002	1.25E-03
Afap1	7.47E-08	1.392	0.091	0.035	1.27E-03
Ern1	7.57E-08	-0.324	0.322	0.46	1.28E-03
Fbxo3	7.74E-08	0.662	0.317	0.205	1.31E-03
Mtmr2	7.75E-08	0.623	0.35	0.236	1.31E-03
Ly6a	7.92E-08	0.520	0.79	0.695	1.34E-03
Ift74	8.05E-08	1.306	0.102	0.042	1.37E-03
Fam181b	8.29E-08	5.329	0.011	0	1.41E-03
Tbc1d22a	8.35E-08	0.482	0.583	0.481	1.42E-03
Cstb	8.42E-08	-0.602	0.389	0.502	1.43E-03
Tet3	8.44E-08	0.518	0.521	0.396	1.43E-03
Svbp	8.45E-08	0.603	0.348	0.231	1.43E-03
Prkcb	8.45E-08	-0.688	0.246	0.374	1.43E-03
Oaf	8.54E-08	2.310	0.037	0.008	1.45E-03
Neb	8.62E-08	2.396	0.037	0.008	1.46E-03
4732471J01Rik	8.63E-08	0.899	0.201	0.113	1.47E-03
Cep112	8.68E-08	1.196	0.14	0.068	1.47E-03
Rbm7	9.03E-08	0.586	0.382	0.265	1.53E-03
Kctd12b	9.10E-08	0.617	0.419	0.295	1.54E-03
Elmod3	9.30E-08	-1.675	0.052	0.141	1.58E-03
Prelp	9.37E-08	-1.170	0.108	0.213	1.59E-03
Itprip	9.44E-08	0.733	0.281	0.176	1.60E-03
Lamc1	9.50E-08	0.792	0.255	0.155	1.61E-03
Spag9	9.52E-08	-0.310	0.901	0.944	1.62E-03
Nrxn2	9.71E-08	-2.364	0.015	0.086	1.65E-03
Kmt2a	9.93E-08	0.395	0.575	0.443	1.69E-03
Snrnp70	9.99E-08	0.442	0.577	0.454	1.70E-03
Tfcp2	1.00E-07	1.166	0.16	0.083	1.70E-03
Tap2	1.01E-07	0.602	0.4	0.284	1.71E-03
Adam15	1.01E-07	0.711	0.337	0.23	1.72E-03
Mannr	1.05E-07	3.369	0.015	0.001	1.79E-03
Thoc7	1.06E-07	0.572	0.374	0.255	1.80E-03
Hprt	1.07E-07	-0.573	0.436	0.55	1.81E-03
Ube3c	1.10E-07	0.800	0.315	0.211	1.86E-03
Eef1g	1.10E-07	0.324	0.847	0.753	1.87E-03
Fbxo45	1.15E-07	1.033	0.132	0.062	1.94E-03
Asxl2	1.15E-07	0.541	0.447	0.324	1.95E-03
Nap1l1	1.16E-07	0.413	0.592	0.46	1.97E-03
Fam76b	1.16E-07	0.673	0.268	0.164	1.97E-03
Flna	1.17E-07	0.936	0.22	0.128	1.99E-03
Fndc3b	1.18E-07	0.465	0.706	0.632	2.00E-03
P3h4	1.19E-07	2.936	0.024	0.003	2.01E-03

Socs1	1.20E-07	-0.675	0.173	0.296	2.03E-03
Tmem30a	1.20E-07	-0.345	0.724	0.803	2.04E-03
Tns1	1.23E-07	0.352	0.84	0.757	2.10E-03
Rplp2	1.24E-07	0.192	0.994	0.992	2.10E-03
Neo1	1.24E-07	1.282	0.093	0.037	2.10E-03
Epb41l2	1.25E-07	-0.253	0.866	0.918	2.12E-03
Spock2	1.28E-07	2.439	0.032	0.006	2.18E-03
Tpbg	1.29E-07	4.329	0.013	0	2.19E-03
Rtraf	1.31E-07	0.458	0.585	0.474	2.22E-03
Id3	1.32E-07	-0.275	0.931	0.964	2.25E-03
Setd2	1.40E-07	0.533	0.469	0.35	2.38E-03
Cd244a	1.40E-07	2.862	0.03	0.005	2.38E-03
Plin2	1.44E-07	0.482	0.672	0.582	2.45E-03
Olfm1	1.45E-07	-0.781	0.274	0.391	2.45E-03
2610307P16Rik	1.45E-07	3.691	0.028	0.005	2.47E-03
Lgals8	1.49E-07	0.604	0.384	0.267	2.53E-03
Fam43a	1.51E-07	0.573	0.514	0.406	2.56E-03
F830016B08Rik	1.53E-07	0.720	0.197	0.109	2.60E-03
Gm12216	1.54E-07	-0.799	0.257	0.381	2.62E-03
Mark3	1.54E-07	0.486	0.6	0.485	2.62E-03
Xbp1	1.55E-07	-0.604	0.406	0.513	2.63E-03
Meis3	1.56E-07	2.521	0.028	0.005	2.64E-03
Mtss1	1.56E-07	-0.260	0.745	0.845	2.65E-03
Kcnb1	1.58E-07	-0.461	0.469	0.587	2.68E-03
Slc16a2	1.59E-07	0.593	0.462	0.351	2.70E-03
BC028528	1.61E-07	0.557	0.382	0.266	2.73E-03
Gm15918	1.63E-07	1.856	0.054	0.016	2.76E-03
Ptpn4	1.64E-07	0.942	0.153	0.078	2.78E-03
Snu13	1.64E-07	-0.554	0.484	0.573	2.78E-03
Tmed10	1.68E-07	-0.360	0.758	0.814	2.84E-03
Ctnna3	1.69E-07	-2.205	0.026	0.101	2.86E-03
Cgnl1	1.70E-07	0.959	0.246	0.154	2.88E-03
Gpr182	1.71E-07	-0.192	0.972	0.993	2.90E-03
Cbx4	1.74E-07	-0.949	0.231	0.341	2.96E-03
Akap9	1.75E-07	0.572	0.423	0.308	2.97E-03
Macrod2	1.78E-07	1.066	0.251	0.157	3.02E-03
Plekhh3	1.78E-07	1.176	0.121	0.056	3.02E-03
Eif5a	1.78E-07	-0.430	0.495	0.607	3.03E-03
Vps13d	1.79E-07	0.707	0.337	0.229	3.03E-03
Hace1	1.82E-07	0.842	0.251	0.154	3.08E-03
Arhgap28	1.82E-07	3.428	0.019	0.002	3.09E-03
Reep5	1.86E-07	-0.550	0.415	0.524	3.15E-03
Hmgcl	1.87E-07	0.621	0.339	0.226	3.17E-03
Dleu2	1.88E-07	-0.510	0.624	0.723	3.20E-03
Fam193a	1.94E-07	0.442	0.49	0.359	3.29E-03
Ccnl2	2.00E-07	0.535	0.451	0.335	3.39E-03

Nkd1	2.02E-07	-0.845	0.151	0.263	3.43E-03
Cnp	2.03E-07	-0.879	0.151	0.262	3.44E-03
Cela1	2.15E-07	-2.179	0.022	0.093	3.65E-03
Xdh	2.15E-07	0.364	0.842	0.775	3.65E-03
Rasgrp3	2.17E-07	-0.267	0.827	0.894	3.68E-03
Art4	2.19E-07	1.521	0.082	0.032	3.73E-03
Dcakd	2.28E-07	1.295	0.11	0.049	3.87E-03
Map1lc3b	2.28E-07	-0.327	0.803	0.844	3.87E-03
Trps1	2.31E-07	0.583	0.495	0.379	3.92E-03
Usp34	2.33E-07	0.372	0.724	0.625	3.96E-03
Gm11944	2.35E-07	1.219	0.108	0.048	3.99E-03
Antxr2	2.36E-07	1.123	0.119	0.055	4.00E-03
Serpina3b	2.37E-07	2.287	0.035	0.007	4.03E-03
Tfpi	2.37E-07	0.360	0.754	0.701	4.03E-03
Homer1	2.38E-07	-1.270	0.102	0.2	4.05E-03
Acvr2a	2.40E-07	0.853	0.283	0.185	4.07E-03
H13	2.41E-07	-0.580	0.363	0.478	4.09E-03
Cnot6l	2.46E-07	0.421	0.542	0.425	4.17E-03
Micu2	2.54E-07	0.896	0.266	0.171	4.31E-03
Pdia5	2.57E-07	0.936	0.212	0.124	4.37E-03
Adcy6	2.60E-07	0.775	0.238	0.144	4.42E-03
Dusp2	2.61E-07	1.114	0.149	0.076	4.42E-03
Phldb2	2.62E-07	0.390	0.758	0.662	4.44E-03
2210016F16Rik	2.65E-07	-1.272	0.073	0.166	4.50E-03
Cfl1	2.67E-07	-0.250	0.892	0.926	4.54E-03
Timp2	2.69E-07	-1.420	0.076	0.168	4.57E-03
Arl6	2.71E-07	1.762	0.054	0.016	4.60E-03
Rcn1	2.76E-07	-0.458	0.538	0.623	4.68E-03
Taf12	2.81E-07	0.707	0.229	0.135	4.76E-03
0610043K17Rik	2.82E-07	-1.110	0.13	0.234	4.78E-03
Plxnd1	2.86E-07	-0.313	0.842	0.87	4.85E-03
Bicra	2.88E-07	0.657	0.311	0.204	4.88E-03
Chfr	2.92E-07	0.945	0.199	0.115	4.95E-03
Cmklr1	2.94E-07	2.131	0.045	0.012	5.00E-03
Senp6	2.97E-07	0.433	0.544	0.419	5.04E-03
Itsn2	2.98E-07	0.435	0.592	0.483	5.05E-03
Pkp4	3.02E-07	0.792	0.337	0.237	5.13E-03
Nipbl	3.05E-07	0.274	0.814	0.687	5.18E-03
Alox12	3.08E-07	-1.508	0.045	0.128	5.23E-03
Apoc1	3.10E-07	0.495	0.562	0.441	5.27E-03
Khdrbs3	3.14E-07	0.977	0.294	0.198	5.32E-03
Akap11	3.28E-07	0.614	0.313	0.207	5.57E-03
Thumpd2	3.36E-07	1.150	0.114	0.053	5.70E-03
Emcn	3.42E-07	0.357	0.754	0.666	5.81E-03
Srek1ip1	3.46E-07	0.773	0.248	0.153	5.87E-03
Ifit2	3.59E-07	0.899	0.283	0.185	6.10E-03

Mbtps1	3.65E-07	0.737	0.296	0.194	6.20E-03
Il10rb	3.65E-07	0.517	0.484	0.362	6.20E-03
Fto	3.66E-07	0.616	0.441	0.333	6.21E-03
Plxdc1	3.80E-07	-1.220	0.073	0.164	6.45E-03
Spopl	3.87E-07	0.578	0.302	0.196	6.57E-03
Sfmbt1	3.90E-07	0.742	0.251	0.156	6.61E-03
Cflar	3.95E-07	-0.430	0.644	0.709	6.71E-03
Pias2	3.96E-07	0.642	0.341	0.235	6.72E-03
Epor	3.97E-07	1.173	0.121	0.058	6.74E-03
Plod1	3.99E-07	0.437	0.592	0.479	6.78E-03
Vat1	4.01E-07	-0.624	0.307	0.421	6.81E-03
Ptbp2	4.03E-07	0.601	0.369	0.26	6.83E-03
Herc3	4.04E-07	0.777	0.263	0.166	6.85E-03
Ampd3	4.09E-07	-1.002	0.104	0.203	6.95E-03
Chchd3	4.13E-07	0.547	0.469	0.359	7.00E-03
C1qtnf9	4.13E-07	1.521	0.063	0.021	7.01E-03
Klhdc8a	4.19E-07	-3.880	0.002	0.057	7.11E-03
Hs3st1	4.22E-07	2.769	0.026	0.004	7.16E-03
Zfp148	4.32E-07	0.432	0.594	0.494	7.33E-03
Cep170	4.33E-07	0.551	0.384	0.274	7.34E-03
Lipe	4.35E-07	0.864	0.184	0.103	7.39E-03
Abhd17b	4.37E-07	0.755	0.417	0.307	7.41E-03
Rc3h2	4.45E-07	0.599	0.408	0.297	7.55E-03
Stxbp4	4.73E-07	1.341	0.082	0.032	8.03E-03
Nsmaf	4.77E-07	0.621	0.259	0.16	8.09E-03
a	4.89E-07	-1.318	0.054	0.138	8.31E-03
9930021J03Rik	5.00E-07	0.557	0.421	0.309	8.49E-03
Heph	5.02E-07	0.912	0.179	0.1	8.53E-03
Tapt1	5.07E-07	0.836	0.173	0.094	8.61E-03
Sec22a	5.16E-07	0.998	0.156	0.082	8.75E-03
Ubxn7	5.26E-07	0.794	0.22	0.132	8.92E-03
Zcchc14	5.30E-07	0.432	0.581	0.468	8.99E-03
Pds5a	5.31E-07	0.489	0.557	0.455	9.01E-03
Cmas	5.32E-07	0.696	0.339	0.236	9.04E-03
Anapc15	5.40E-07	-0.547	0.425	0.538	9.16E-03
Fbxo22	5.41E-07	0.768	0.281	0.183	9.19E-03
Hk1	5.43E-07	0.623	0.341	0.236	9.22E-03
Fkbp1a	5.43E-07	-0.216	0.959	0.975	9.22E-03
Upp1	5.50E-07	0.627	0.367	0.26	9.33E-03
Thoc2	5.52E-07	0.424	0.629	0.523	9.36E-03
Apobec3	5.53E-07	0.660	0.27	0.172	9.38E-03
Aff4	5.54E-07	-0.514	0.553	0.631	9.40E-03
Mgst1	5.63E-07	0.457	0.413	0.293	9.56E-03
Cfap97	5.68E-07	0.825	0.212	0.126	9.64E-03
Dock1	5.75E-07	0.360	0.801	0.715	9.76E-03
Mdm4	5.77E-07	0.541	0.371	0.26	9.79E-03

Itpr3	5.77E-07	1.091	0.13	0.064	9.80E-03
Sar1a	5.83E-07	-0.549	0.309	0.432	9.89E-03
Clec1a	5.92E-07	0.456	0.549	0.438	1.01E-02
Kctd17	5.96E-07	0.729	0.248	0.155	1.01E-02
Slc7a5	5.99E-07	-0.699	0.229	0.34	1.02E-02
Srpr	6.04E-07	-0.442	0.443	0.559	1.02E-02
Acaa2	6.05E-07	0.529	0.395	0.284	1.03E-02
Cdc42ep3	6.15E-07	0.759	0.272	0.175	1.04E-02
Gatad2b	6.31E-07	0.533	0.495	0.386	1.07E-02
Prkcg	6.31E-07	-1.003	0.056	0.141	1.07E-02
Pik3c2b	6.33E-07	0.570	0.423	0.313	1.07E-02
Cdc40	6.43E-07	0.631	0.35	0.244	1.09E-02
Apcdd1	6.67E-07	2.184	0.032	0.007	1.13E-02
Abhd8	6.68E-07	1.132	0.121	0.058	1.13E-02
Serpina1e	6.75E-07	-2.977	0.009	0.067	1.15E-02
Kdm5b	6.75E-07	0.560	0.315	0.21	1.15E-02
Eif3k	6.84E-07	0.393	0.7	0.593	1.16E-02
Uri1	6.87E-07	0.579	0.365	0.255	1.17E-02
Rsf1	6.96E-07	0.449	0.492	0.372	1.18E-02
Akr1b3	6.99E-07	-0.598	0.322	0.431	1.19E-02
Arrb2	7.04E-07	-0.594	0.307	0.418	1.20E-02
Pdlim1	7.07E-07	-0.505	0.533	0.624	1.20E-02
Klhdc10	7.10E-07	0.686	0.281	0.182	1.20E-02
Cul1	7.11E-07	0.409	0.54	0.412	1.21E-02
Gabarap	7.21E-07	-0.256	0.89	0.903	1.22E-02
Zbtb11	7.27E-07	-0.729	0.289	0.406	1.23E-02
Obsl1	7.38E-07	2.047	0.037	0.009	1.25E-02
Atp6v1a	7.40E-07	-0.519	0.367	0.485	1.26E-02
Ftl1-ps1	7.42E-07	-0.887	0.13	0.231	1.26E-02
Asah2	7.42E-07	0.520	0.475	0.362	1.26E-02
Fip1l1	7.45E-07	0.512	0.376	0.263	1.26E-02
Vegfb	7.64E-07	-0.853	0.177	0.282	1.30E-02
Ergic3	7.66E-07	0.510	0.317	0.208	1.30E-02
Vta1	7.68E-07	0.640	0.326	0.224	1.30E-02
Btrc	7.70E-07	0.710	0.305	0.206	1.31E-02
Inpp5k	7.78E-07	0.521	0.369	0.255	1.32E-02
Hmgn1	7.87E-07	0.566	0.4	0.29	1.34E-02
Bcr	7.88E-07	0.682	0.27	0.175	1.34E-02
Fryl	7.99E-07	0.636	0.495	0.4	1.36E-02
Nmt2	8.01E-07	0.860	0.235	0.147	1.36E-02
Ndst1	8.04E-07	0.436	0.527	0.408	1.37E-02
Gm45345	8.16E-07	3.106	0.019	0.002	1.38E-02
Tnfaip8l1	8.18E-07	0.812	0.257	0.166	1.39E-02
Pcdhb4	8.19E-07	2.439	0.03	0.006	1.39E-02
Nup98	8.23E-07	-0.598	0.404	0.506	1.40E-02
Pot1b	8.32E-07	1.032	0.162	0.089	1.41E-02

Orm1	8.34E-07	1.394	0.071	0.027	1.42E-02
Clstn1	8.39E-07	2.287	0.03	0.006	1.42E-02
Dgkd	8.48E-07	0.831	0.233	0.146	1.44E-02
Mrpl30	8.54E-07	0.545	0.397	0.287	1.45E-02
Cdkal1	8.60E-07	0.534	0.395	0.283	1.46E-02
Eva1a	8.61E-07	2.043	0.045	0.013	1.46E-02
Otud4	8.79E-07	0.530	0.341	0.232	1.49E-02
Arfgap1	8.86E-07	0.716	0.212	0.125	1.50E-02
Calcoco1	8.86E-07	0.931	0.164	0.09	1.50E-02
Xpr1	8.93E-07	0.665	0.339	0.236	1.52E-02
Tmem221	8.95E-07	3.369	0.015	0.001	1.52E-02
Plpp1	9.08E-07	-0.188	0.965	0.986	1.54E-02
Tap1	9.10E-07	0.732	0.272	0.178	1.54E-02
Rab3gap2	9.21E-07	0.633	0.391	0.289	1.56E-02
Atp9a	9.23E-07	1.628	0.067	0.024	1.57E-02
Zmym3	9.28E-07	1.334	0.076	0.029	1.58E-02
Myl12a	9.29E-07	0.371	0.771	0.684	1.58E-02
Cox4i1	9.32E-07	-0.203	0.968	0.978	1.58E-02
Actn1	9.34E-07	0.799	0.231	0.143	1.59E-02
Camp	9.36E-07	-1.185	0.065	0.15	1.59E-02
Peak1	9.50E-07	0.330	0.903	0.887	1.61E-02
Gm12185	9.53E-07	1.091	0.138	0.071	1.62E-02
Sgk1	9.58E-07	-0.341	0.788	0.84	1.63E-02
Med13l	9.78E-07	0.440	0.531	0.422	1.66E-02
Dpm3	9.78E-07	-0.489	0.421	0.527	1.66E-02
Rpl9-ps6	9.88E-07	0.782	0.222	0.136	1.68E-02
Ppp2r5c	9.91E-07	0.378	0.579	0.461	1.68E-02
Gbp9	9.94E-07	0.829	0.222	0.136	1.69E-02
Usp3	9.95E-07	0.370	0.607	0.483	1.69E-02
Arhgap23	1.03E-06	-0.309	0.773	0.811	1.75E-02
Hspg2	1.04E-06	0.259	0.862	0.76	1.76E-02
Cnst	1.08E-06	0.750	0.222	0.136	1.83E-02
Inpp5d	1.08E-06	0.544	0.402	0.289	1.84E-02
Grap	1.09E-06	-0.594	0.305	0.415	1.84E-02
Cog4	1.09E-06	0.748	0.231	0.143	1.85E-02
Rflnb	1.10E-06	1.762	0.063	0.022	1.87E-02
Syt7	1.10E-06	1.106	0.125	0.062	1.87E-02
Gngt2	1.10E-06	-0.355	0.693	0.773	1.87E-02
Blmh	1.11E-06	0.521	0.384	0.276	1.88E-02
Dcst1	1.11E-06	2.659	0.022	0.003	1.88E-02
Lysmd2	1.11E-06	2.659	0.022	0.003	1.88E-02
Kcnk5	1.11E-06	2.884	0.026	0.005	1.88E-02
Gja5	1.12E-06	2.914	0.026	0.005	1.90E-02
Hs6st1	1.12E-06	0.747	0.255	0.164	1.91E-02
Kif1c	1.13E-06	0.597	0.289	0.191	1.92E-02
Cep295	1.13E-06	0.936	0.164	0.09	1.92E-02

Adk	1.14E-06	0.859	0.194	0.114	1.93E-02
Pold3	1.20E-06	0.923	0.192	0.113	2.04E-02
Slc6a8	1.21E-06	1.397	0.076	0.03	2.06E-02
Gtf3c1	1.21E-06	0.636	0.255	0.161	2.06E-02
Galk2	1.22E-06	0.715	0.317	0.221	2.07E-02
Prrg1	1.22E-06	0.647	0.339	0.238	2.08E-02
Ahr	1.23E-06	0.503	0.454	0.348	2.09E-02
Stim1	1.24E-06	0.605	0.395	0.287	2.10E-02
Slc38a6	1.24E-06	0.482	0.521	0.415	2.10E-02
Erlec1	1.26E-06	0.582	0.356	0.254	2.14E-02
Bax	1.27E-06	-0.487	0.523	0.609	2.16E-02
Hdgfl3	1.28E-06	0.576	0.363	0.259	2.17E-02
Snx10	1.30E-06	0.979	0.127	0.064	2.21E-02
Serpina3g	1.31E-06	0.514	0.594	0.498	2.22E-02
Rara	1.40E-06	0.836	0.231	0.145	2.37E-02
Ankrd10	1.40E-06	0.633	0.266	0.171	2.38E-02
Sfswap	1.41E-06	0.547	0.387	0.281	2.40E-02
Dennd1b	1.45E-06	0.644	0.322	0.224	2.46E-02
Ppme1	1.46E-06	0.643	0.309	0.209	2.48E-02
Nfix	1.46E-06	0.458	0.382	0.269	2.48E-02
Cngb1	1.47E-06	1.835	0.045	0.013	2.49E-02
Fam216a	1.47E-06	1.647	0.056	0.019	2.50E-02
Fam193b	1.49E-06	0.785	0.214	0.13	2.52E-02
Atg2b	1.49E-06	0.889	0.168	0.094	2.53E-02
Csnk1g1	1.51E-06	0.611	0.35	0.244	2.56E-02
Sdc4	1.52E-06	0.613	0.285	0.188	2.58E-02
Ndrg2	1.53E-06	-1.007	0.104	0.196	2.60E-02
Sh2d3c	1.61E-06	0.450	0.486	0.372	2.73E-02
Lrig1	1.62E-06	2.199	0.039	0.01	2.74E-02
Sgce	1.62E-06	0.981	0.151	0.082	2.75E-02
Rnf216	1.64E-06	0.429	0.492	0.37	2.78E-02
Srpk2	1.64E-06	0.505	0.475	0.369	2.78E-02
Cox6b2	1.64E-06	3.936	0.013	0.001	2.79E-02
Map2k5	1.66E-06	0.526	0.421	0.309	2.82E-02
Hnrnph1	1.70E-06	-0.386	0.618	0.69	2.89E-02
Chp2	1.72E-06	1.936	0.039	0.01	2.92E-02
Erc1	1.76E-06	0.602	0.397	0.296	2.99E-02
Gm34455	1.76E-06	-1.005	0.188	0.287	3.00E-02
AU021092	1.78E-06	-0.403	0.197	0.313	3.01E-02
Unc45bos	1.78E-06	2.361	0.03	0.006	3.03E-02
Fuca1	1.80E-06	0.489	0.4	0.289	3.05E-02
Ncor1	1.82E-06	0.315	0.81	0.706	3.09E-02
Stab2	1.86E-06	0.320	0.924	0.988	3.16E-02
Desi2	1.90E-06	0.434	0.469	0.354	3.23E-02
Clec4b1	1.94E-06	1.631	0.058	0.02	3.29E-02
Naa16	1.96E-06	0.700	0.233	0.146	3.33E-02

Slc45a3	1.97E-06	1.981	0.041	0.011	3.34E-02
Sema6b	1.99E-06	0.582	0.38	0.275	3.37E-02
Phax	1.99E-06	0.625	0.298	0.202	3.37E-02
Vwa8	1.99E-06	0.659	0.266	0.173	3.38E-02
Csrnp1	2.20E-06	-0.799	0.132	0.229	3.73E-02
Col4a3bp	2.23E-06	0.478	0.555	0.462	3.79E-02
Fam160b1	2.28E-06	0.530	0.305	0.204	3.88E-02
Atp5h	2.29E-06	-0.218	0.909	0.942	3.89E-02
Lox	2.31E-06	4.106	0.011	0	3.93E-02
Mlf1	2.35E-06	-1.925	0.028	0.094	3.99E-02
Cep83	2.37E-06	-0.655	0.24	0.35	4.03E-02
P3h3	2.38E-06	1.071	0.11	0.053	4.04E-02
Btbd1	2.39E-06	0.620	0.35	0.253	4.05E-02
Zfp30	2.39E-06	1.232	0.076	0.03	4.06E-02
Pnn	2.41E-06	0.509	0.402	0.292	4.10E-02
Mrps7	2.45E-06	0.642	0.253	0.162	4.16E-02
Acvr1	2.47E-06	0.540	0.417	0.316	4.20E-02
C230035I16Rik	2.50E-06	1.661	0.05	0.016	4.25E-02
Ncoa2	2.52E-06	0.449	0.512	0.411	4.29E-02
Cmtm4	2.53E-06	0.791	0.229	0.144	4.29E-02
Armcx2	2.55E-06	1.026	0.162	0.092	4.32E-02
Dis3l2	2.56E-06	0.565	0.315	0.217	4.35E-02
Sumo3	2.60E-06	-0.557	0.4	0.498	4.41E-02
Meis2	2.63E-06	0.242	0.957	0.964	4.46E-02
Apobec1	2.67E-06	0.929	0.201	0.123	4.53E-02
Junos	2.68E-06	-1.105	0.065	0.144	4.55E-02
Rbp4	2.73E-06	0.797	0.184	0.108	4.64E-02
Herc2	2.76E-06	0.546	0.523	0.423	4.68E-02
Gm50333	2.77E-06	-2.517	0.011	0.066	4.69E-02
Klhl29	2.77E-06	2.599	0.026	0.005	4.71E-02
Cplx2	2.78E-06	1.073	0.13	0.067	4.71E-02
Pear1	2.80E-06	-0.502	0.395	0.494	4.75E-02
Prpf40b	2.82E-06	1.064	0.132	0.069	4.79E-02
Ajuba	2.86E-06	1.821	0.032	0.008	4.86E-02
Kdm5c	2.87E-06	0.464	0.341	0.237	4.87E-02
Nup205	2.88E-06	0.873	0.143	0.076	4.89E-02
Gnpat	2.88E-06	0.914	0.158	0.088	4.89E-02
Mllt6	2.91E-06	0.636	0.281	0.188	4.94E-02
Qars	2.92E-06	0.814	0.171	0.097	4.95E-02
Adam12	2.93E-06	-0.943	0.082	0.167	4.98E-02
Ube2r2	2.93E-06	0.416	0.598	0.494	4.98E-02
Slc25a23	2.94E-06	1.170	0.106	0.051	4.99E-02

Supplementary Table 8. Pairwise comparison of GAN-Adj.NT verses GAN endothelial cells. Significant DEGs (adjusted p-value < 0.05) shown.

Gene	p_val	avg_log2FC	pct.1	pct.2	p_val_adj
Gm17749	8.13E-08	2.028	0.112	0.026	1.38E-03
Ifit1	1.70E-08	1.734	0.253	0.125	2.89E-04
Nabp1	6.96E-09	1.489	0.212	0.086	1.18E-04
Oasl1	8.31E-07	1.295	0.196	0.091	1.41E-02
Slc5a3	9.31E-08	1.268	0.216	0.099	1.58E-03
Camp	1.48E-06	1.206	0.159	0.065	2.51E-02
9930111J21Rik2	4.62E-08	1.182	0.39	0.257	7.84E-04
Ngp	7.13E-07	1.158	0.198	0.091	1.21E-02
Csrnp1	4.61E-08	1.141	0.261	0.132	7.82E-04
Hsph1	1.52E-08	1.127	0.425	0.285	2.59E-04
Hbegf	1.06E-07	1.099	0.352	0.222	1.79E-03
Sertad2	1.61E-08	1.096	0.431	0.289	2.73E-04
Gda	8.93E-07	1.054	0.231	0.119	1.52E-02
Ezh2	1.85E-06	1.040	0.276	0.16	3.15E-02
Sertad1	7.95E-09	1.038	0.384	0.238	1.35E-04
Daxx	1.08E-06	1.029	0.28	0.162	1.83E-02
Cdkn1a	2.73E-07	1.025	0.453	0.324	4.64E-03
Hspa1b	9.91E-09	1.003	0.556	0.413	1.68E-04
Rsad2	2.55E-08	0.987	0.398	0.251	4.32E-04
Ifi211	2.61E-07	0.980	0.352	0.222	4.43E-03
Polg2	6.46E-09	0.906	0.351	0.192	1.10E-04
Dusp5	2.98E-07	0.873	0.292	0.164	5.06E-03
Mup20	2.58E-06	0.834	0.445	0.315	4.38E-02
Ccl2	3.55E-07	0.808	0.155	0.056	6.02E-03
Fosb	1.07E-07	0.776	0.616	0.501	1.81E-03
Pde4b	1.45E-07	0.771	0.547	0.41	2.47E-03
Itga8	4.05E-07	0.724	0.352	0.214	6.88E-03
Azin1	2.33E-07	0.697	0.415	0.276	3.96E-03
Btg1	2.64E-08	0.678	0.637	0.525	4.48E-04
Ern1	2.79E-08	0.676	0.466	0.322	4.73E-04
Cxcl9	2.80E-08	0.666	0.769	0.702	4.75E-04
Rnd3	3.03E-07	0.642	0.413	0.261	5.15E-03
Iigp1	1.84E-06	0.639	0.825	0.771	3.13E-02
Pnp	5.67E-08	0.595	0.759	0.693	9.62E-04
Rhob	2.24E-08	0.583	0.782	0.674	3.80E-04
Jun	1.90E-08	0.536	0.925	0.89	3.22E-04
Ccnl1	4.35E-08	0.533	0.618	0.473	7.38E-04
Gja1	6.97E-09	0.529	0.708	0.564	1.18E-04
Manf	1.25E-06	0.489	0.579	0.46	2.11E-02
Dusp1	1.01E-07	0.477	0.858	0.732	1.71E-03
Gm12840	4.13E-07	0.475	0.825	0.756	7.01E-03

Nfkbia	6.10E-08	0.417	0.888	0.834	1.03E-03
Bmp2	4.56E-08	0.321	0.921	0.844	7.75E-04
Jund	2.13E-06	0.310	0.862	0.773	3.62E-02
Calm1	5.84E-07	0.274	0.991	0.983	9.92E-03
Ptma	9.59E-08	0.254	1	0.996	1.63E-03
Irf1	2.35E-06	0.241	0.743	0.596	3.99E-02
Actb	1.39E-06	0.231	0.997	0.998	2.35E-02
Rps12	1.39E-06	-0.158	0.999	0.998	2.36E-02
Rpl13a	1.04E-06	-0.182	0.996	1	1.76E-02
Rps20	1.71E-07	-0.205	0.999	0.996	2.90E-03
Igfbp7	3.63E-07	-0.215	0.997	0.996	6.16E-03
Sptbn1	2.65E-07	-0.222	0.993	0.998	4.49E-03
Rps16	1.78E-07	-0.224	0.996	1	3.03E-03
Rps27a	2.32E-07	-0.229	1	0.996	3.93E-03
Rpl27	6.67E-07	-0.230	0.981	0.987	1.13E-02
Rps8	9.73E-09	-0.231	0.999	0.998	1.65E-04
Rps13	7.03E-07	-0.234	0.989	0.991	1.19E-02
Rps14	7.59E-09	-0.236	0.999	0.996	1.29E-04
Sparc	2.71E-06	-0.236	0.969	0.985	4.61E-02
Rpl11	2.47E-07	-0.238	0.988	0.991	4.20E-03
Rps23	6.75E-09	-0.240	0.997	0.998	1.15E-04
Rps24	1.10E-08	-0.247	0.997	0.998	1.86E-04
Rpl18	8.26E-07	-0.249	0.977	0.991	1.40E-02
Rps9	1.25E-08	-0.250	0.996	0.994	2.13E-04
Rpl15	1.96E-06	-0.254	0.976	0.987	3.33E-02
Cd47	1.33E-06	-0.256	0.968	0.976	2.26E-02
Rpl30	3.30E-08	-0.256	0.999	0.991	5.60E-04
Rps26	8.92E-07	-0.263	0.972	0.983	1.51E-02
Rpl35	1.36E-07	-0.263	0.977	0.985	2.30E-03
Rpsa	2.13E-06	-0.264	0.981	0.991	3.62E-02
Rbm39	3.18E-07	-0.267	0.958	0.972	5.40E-03
Pfdn5	8.68E-08	-0.278	0.921	0.959	1.47E-03
Rps18	6.46E-08	-0.279	0.984	0.985	1.10E-03
Rpl4	2.89E-06	-0.281	0.849	0.916	4.90E-02
Rps3	2.10E-08	-0.282	0.987	0.996	3.57E-04
Rps19	7.95E-09	-0.285	0.988	0.991	1.35E-04
Rps5	4.09E-08	-0.290	0.991	0.989	6.94E-04
Atrx	4.74E-07	-0.290	0.872	0.916	8.05E-03
Rpl3	1.07E-08	-0.297	0.974	0.983	1.82E-04
Rpl9	8.57E-09	-0.300	0.996	0.996	1.46E-04
Psap	1.36E-08	-0.312	0.938	0.968	2.30E-04
Itgb1	1.17E-07	-0.324	0.91	0.95	1.99E-03
Itga9	2.25E-06	-0.324	0.839	0.875	3.82E-02
Rps7	3.40E-08	-0.325	0.981	0.991	5.78E-04
Cd36	2.97E-08	-0.328	0.935	0.952	5.05E-04
Elk3	2.45E-07	-0.332	0.837	0.896	4.16E-03

Mef2c	4.08E-08	-0.338	0.777	0.879	6.93E-04
Ndfip1	7.90E-07	-0.341	0.758	0.836	1.34E-02
Esam	2.82E-08	-0.342	0.868	0.937	4.79E-04
Flnb	1.61E-06	-0.352	0.722	0.825	2.74E-02
F2r	1.54E-06	-0.354	0.702	0.81	2.62E-02
Peak1	9.68E-07	-0.361	0.887	0.903	1.64E-02
Sh3glb1	8.16E-09	-0.364	0.853	0.905	1.39E-04
Ccdc85b	1.34E-07	-0.370	0.679	0.821	2.28E-03
Nid1	1.50E-07	-0.383	0.595	0.726	2.55E-03
Rapgef1	9.91E-07	-0.384	0.598	0.726	1.68E-02
Eif3h	2.39E-06	-0.384	0.645	0.737	4.05E-02
Anp32a	5.21E-07	-0.390	0.626	0.749	8.84E-03
Ncor1	8.82E-07	-0.397	0.703	0.81	1.50E-02
Car8	2.64E-06	-0.397	0.516	0.657	4.48E-02
Ly6a	1.66E-06	-0.400	0.691	0.79	2.82E-02
Stab2	3.21E-08	-0.402	0.921	0.924	5.46E-04
Uba52	8.68E-07	-0.404	0.69	0.788	1.47E-02
Btnl9	1.25E-07	-0.407	0.703	0.795	2.13E-03
Adam23	1.32E-07	-0.407	0.901	0.924	2.24E-03
Cd59a	1.83E-08	-0.411	0.778	0.866	3.10E-04
St3gal4	1.71E-07	-0.417	0.767	0.851	2.91E-03
Zmiz1	1.29E-07	-0.420	0.734	0.847	2.20E-03
Rnf130	1.07E-06	-0.423	0.618	0.717	1.81E-02
Pura	1.42E-07	-0.434	0.544	0.689	2.42E-03
Cpd	5.25E-07	-0.436	0.563	0.672	8.91E-03
Itpr2	1.34E-06	-0.440	0.531	0.657	2.27E-02
Scaf11	7.18E-07	-0.449	0.536	0.657	1.22E-02
Notch1	1.96E-07	-0.453	0.618	0.724	3.33E-03
Raph1	1.97E-07	-0.454	0.659	0.765	3.34E-03
Zeb1	8.54E-08	-0.459	0.879	0.92	1.45E-03
Rora	1.38E-06	-0.461	0.77	0.827	2.34E-02
Ushbp1	1.17E-07	-0.468	0.608	0.726	1.98E-03
Fkbp5	7.16E-08	-0.471	0.602	0.724	1.21E-03
Oip5os1	1.81E-08	-0.474	0.664	0.786	3.08E-04
Stim2	6.40E-08	-0.477	0.587	0.706	1.09E-03
Dennd4c	5.10E-07	-0.506	0.477	0.624	8.65E-03
Afdn	2.06E-06	-0.516	0.478	0.583	3.50E-02
Cd2ap	6.22E-08	-0.530	0.612	0.724	1.06E-03
H2-Q7	5.42E-08	-0.533	0.612	0.702	9.21E-04
Ndufa6	3.69E-08	-0.534	0.605	0.706	6.26E-04
Tcf7l1	6.56E-09	-0.535	0.688	0.778	1.11E-04
Gpi1	3.06E-07	-0.537	0.47	0.611	5.19E-03
Map4k5	2.71E-06	-0.538	0.64	0.717	4.60E-02
Grb2	1.60E-06	-0.538	0.409	0.533	2.71E-02
Pkn1	2.56E-06	-0.545	0.359	0.488	4.35E-02
Jcad	8.92E-07	-0.554	0.382	0.514	1.51E-02

Eif4b	5.59E-07	-0.557	0.466	0.587	9.48E-03
Slc25a4	1.20E-06	-0.558	0.499	0.611	2.04E-02
Ryk	1.58E-06	-0.565	0.363	0.497	2.69E-02
Evi5	2.10E-06	-0.573	0.407	0.527	3.56E-02
Nova2	2.75E-07	-0.576	0.327	0.471	4.67E-03
Itch	9.77E-07	-0.587	0.422	0.542	1.66E-02
Galnt18	2.48E-08	-0.592	0.591	0.73	4.21E-04
Pip4k2a	1.50E-06	-0.596	0.515	0.605	2.55E-02
Eif4ebp1	3.44E-07	-0.599	0.347	0.484	5.84E-03
Lyve1	2.16E-08	-0.607	0.602	0.728	3.67E-04
Wdr11	1.33E-06	-0.624	0.208	0.337	2.25E-02
Cbfa2t3	7.76E-07	-0.625	0.435	0.551	1.32E-02
Wipf1	6.94E-07	-0.629	0.382	0.514	1.18E-02
Man2b1	1.59E-08	-0.641	0.452	0.59	2.70E-04
Ei24	8.79E-07	-0.647	0.212	0.343	1.49E-02
Rdx	2.09E-08	-0.655	0.528	0.657	3.55E-04
Arrb1	1.64E-08	-0.659	0.503	0.609	2.78E-04
Pgam1	2.53E-06	-0.684	0.349	0.462	4.30E-02
Pfkfb3	2.04E-08	-0.700	0.25	0.404	3.46E-04
Nhsl2	4.37E-08	-0.702	0.305	0.458	7.41E-04
B130024G19Rik	4.11E-08	-0.720	0.198	0.343	6.98E-04
Fmn1	2.31E-06	-0.720	0.273	0.397	3.93E-02
Abhd6	2.55E-08	-0.722	0.241	0.391	4.33E-04
Inhbb	2.22E-06	-0.726	0.297	0.419	3.77E-02
Tef	1.30E-07	-0.750	0.233	0.374	2.21E-03
Cbx6	2.55E-06	-0.758	0.263	0.38	4.32E-02
Sema6d	1.30E-07	-0.771	0.36	0.49	2.21E-03
Glb1	2.01E-06	-0.774	0.258	0.378	3.42E-02
Snx25	2.18E-07	-0.776	0.269	0.404	3.70E-03
BC028528	1.69E-08	-0.779	0.234	0.382	2.86E-04
Akap11	3.64E-07	-0.803	0.187	0.313	6.18E-03
Hic1	3.31E-08	-0.819	0.204	0.348	5.63E-04
Fam102a	1.68E-06	-0.820	0.216	0.333	2.86E-02
C1qtnf6	4.93E-08	-0.896	0.14	0.268	8.37E-04
Tspan5	8.35E-07	-0.910	0.194	0.315	1.42E-02
Prelid2	2.19E-07	-0.981	0.159	0.279	3.72E-03
Cxxc5	8.47E-08	-1.056	0.114	0.229	1.44E-03
Tbc1d30	8.39E-07	-1.061	0.117	0.22	1.42E-02
Adora2a	2.47E-06	-1.090	0.097	0.19	4.20E-02
Pdpr	1.21E-06	-1.126	0.086	0.179	2.05E-02
Plod2	8.52E-08	-1.138	0.157	0.279	1.45E-03
Slc46a3	4.97E-07	-1.353	0.044	0.123	8.43E-03
Gpr183	1.81E-07	-1.871	0.035	0.11	3.07E-03
Ak4	1.99E-07	-2.638	0.008	0.058	3.38E-03

Supplementary Table 9. Curated PLVAP⁺ signatures.

C3_PLVAP_signature	C4_PLVAP_signature	C9_PLVAP_signature
Alb	Abcg2	Ackr1
Ambp	Apoa2	Actn1
Aplnr	Aqp1	Aplnr
Apoa1	Arrdc3	Bgn
Apoa2	B2m	Bnc2
Apoc1	Car4	Col15a1
Apoc3	Cd74	Col6a2
Col15a1	Cxcr4	Cpxm2
Col4a1	Dnase1l3	Csrp1
Col4a2	Esm1	Donson
Cpm	Flt1	Dusp23
Cyba	Fos	Fabp5
Cysltr1	Gja1	Fam118a
Ddit4	H2-Q10	Fam167b
Dusp23	H2-Q7	Fn1
Dusp6	Gm8909	Hapln3
Dysf	H2-D1	Htra1
Eng	H2-M2	Impdh2
Fabp1	H2-Q10	Lamb1
Fam167b	H2-Q7	Lhx6
Fgb	H2-Q10	Mmrn1
Fgg	H2-Q7	Mpzl2
Folh1	H2-DMa	Myh9
Gabrd	H2-Aa	Myl9
Gmfg	H2-Aa	Myo5a
Hecw2	H2-Ab1	Nnmt
Kdr	H2-Ea	Nuak1
Lbh	H2-Eb1	Pgf
Ldhb	H2-Eb1	Pkm
Nrp1	H2-T23	Plp2
Nxph1	Hsp90aa1	Postn
Pla2g2a	Hspa1a	Prcp
Plvap	Iglc1	Rai14
Plxnd1	Iglc2	Rgs16
Ppib	Iglc3	Rora
Rab13	Inhbb	Sele
Rasgrp3	Insr	Selp
Rbp4	Isg20	Serpine1
Serpina1b	Ivns1abp	Smad1
Serpina1e	Kcnk5	Snai1
Sparc	Lpar6	Sox4
Spink1	Pcdh17	Tagln

Spp1	Plk2	Tpd52l1
Thy1	Plvap	Tshz2
Tmem204	Slc2a3	Vwa1
Tmem255b	Sparc	Zfp521
Vtn	Sparcl1	
	Spry1	
	Stc1	
	Tgfbr2	
	Ttr	
	Ubc	
	Ugcg	

References (Author names in bold designate shared co-first authorship)

[1] Chen S, Zhou Y, Chen Y, Gu J. Fastp: An ultra-fast all-in-one FASTQ preprocessor. Bioinformatics 2018;34(17):i884-i890. https://doi.org/10.1093/bioinformatics/bty560.

[2] Angerer P, Haghverdi L, Büttner M, Theis FJ, et al. Destiny: Diffusion maps for large-scale single-cell data in R. Bioinformatics 2016;32(8):1241–3. https://doi.org/10.1093/bioinformatics/btv715.

[3] La Manno G, Soldatov R, Zeisel A, et al. RNA velocity of single cells. Nature 2018;560(7719):494–8. https://doi.org/10.1038/s41586-018-0414-6.

[4] Virshup I, Rybakov S, Theis FJ, et al. anndata: Access and store annotated data matrices. J Open Source Softw 2024;9:4371. https://doi.org/10.21105/joss.04371.

[5] Bergen V, Lange M, Peidli S, et al. Generalizing RNA velocity to transient cell states through dynamical modeling. Nat Biotechnol 2020;38:1408–14. https://doi.org/10.1038/s41587-020-0591-3.

[6] Wolf FA, Angerer P, Theis FJ. SCANPY: Large-scale single-cell gene expression data analysis. Genome Biol 2018;19. https://doi.org/10.1186/s13059-017-1382-0.

[7] **Weiler P, Lange M**, Klein M, et al. CellRank 2: unified fate mapping in multiview single-cell data. Nat Methods 2024;21:1196–205. https://doi.org/10.1038/s41592-024-02303-9.

[8] Gulati GS, Sikandar SS, Wesche DJ, et al. Single-cell transcriptional diversity is a hallmark of developmental potential. Science 2020;367:405–11. https://doi.org/10.1126/science.aax0249.

[9] **Ng MSF, Kwok I, Tan L**, et al. Deterministic reprogramming of neutrophils within tumors. Science 2024;383:eadf6493. https://doi.org/10.1126/science.adf6493.

[10] Holla P, Dizon B, Ambegaonkar AA, et al. Shared transcriptional profiles of atypical B cells suggest common drivers of expansion and function in malaria, HIV, and autoimmunity. Sci Adv 2021;7:eabg8384. https://doi.org/10.1126/sciadv.abg8384.

[11] King HW, Orban N, Riches JC, et al. Single-cell analysis of human B cell maturation predicts how antibody class switching shapes selection dynamics. Sci Immunol 2021;6:eabe6291. https://doi.org/10.1126/sciimmunol.abe6291.

[12] Victora GD, Dominguez-Sola D, Holmes AB, et al. Identification of human germinal center light and dark zone cells and their relationship to human B-cell lymphomas. Blood 2012;120:2240–8. https://doi.org/10.1182/blood-2012-03-415380.
