# Supplementary material for: Hyperinflammatory reprogramming marks the transition from MASH to HCC
Source: JHEP Rep. 2026 Oct 7;8(12):102055. doi: 10.1016/j.jhepr.2026.102055 (PMC13756514; doi:10.1016/j.jhepr.2026.102055)
Supplement: Supplementary file 4 [file mmc2.docx]

**JHEP Reports**

**CTAT methods**

Tables for a “Complete, Transparent, Accurate and Timely account” (CTAT) are now mandatory for all revised submissions. The aim is to enhance the reproducibility of methods.

- Only include the parts relevant to your study
- Refer to the CTAT in the main text as ‘Supplementary CTAT Table’
- Do not add subheadings
- Add as many rows as needed to include all information
- Only include one item per row

**If the CTAT form is not relevant to your study, please outline the reasons why:**

1.1 **Antibodies**

| **Name** | **Citation** | **Supplier** | **Cat no.** | **Clone no.** |
| --- | --- | --- | --- | --- |
| Ki67 |  | Abcam | ab15580 | - |
| Trem2 |  | Abcam | ab305103 | EPR26210-1 |
| Trem2 (WB) |  | Cell Signaling Technology | 61788 | E6T1P |
| F4/80 |  | Miltenyi Biotec | 130-116-499 | REA126 |
| CD31 |  | Miltenyi Biotec | 130-111-710 | REA793 |
| CD146 |  | Miltenyi Biotec | 130-118-407 | REA1064 |
| Albumin |  | Proteintech | 16475-1-AP | - |
| α-SMA |  | Miltenyi Biotec | 130-123-363 | REAL650 |
| Ly6G |  | BioLegend | 127606 | 1A8 |
| CD11b |  | BD Biosciences | 563402 | M1/70 |
| Siglec-F |  | BD Biosciences | 562757 | E50-2440 |
| CD101 |  | ThermoFisher Scientific | 12-1011-82 | Moushi101 |
| Alexa Fluor 647 donkey anti-rabbit |  | ThermoFisher Scientific | A-31573 | - |
| G-CSF |  | SouthernBiotech | 10128-01 | BVD11-37G10 |
| TotalSeq™-B0301 anti-mouse Hashtag 1 |  | BioLegend | 155831 | M1/42 |
| TotalSeq™-B0301 anti-mouse Hashtag 2 |  | BioLegend | 155833 | M1/42 |
| TotalSeq™-B0301 anti-mouse Hashtag 3 |  | BioLegend | 155835 | M1/42 |
| TotalSeq™-B0301 anti-mouse Hashtag 4 |  | BioLegend | 155837 | M1/42 |
| TotalSeq™-B0301 anti-mouse Hashtag 5 |  | BioLegend | 155839 | M1/42 |

1.2 **Cell lines**

| **Name** | **Citation** | **Supplier** | **Cat no.** | **Passage no.** | **Authentication test method** |
| --- | --- | --- | --- | --- | --- |
| RAW264.7 |  | ATCC | TIB-71 |  | Trem2 knockout validated by western blot and Sanger sequencing |

1.3 **Organisms**

| **Name** | **Citation** | **Supplier** | **Strain** | **Sex** | **Age** | **Overall n number** |
| --- | --- | --- | --- | --- | --- | --- |
| Mouse |  | Invivos | C57BL6/J | Male | 8 weeks at diet initiation | 148 |

1.4 **Sequence based reagents**

| **Name** | **Sequence** | **Supplier** |
| --- | --- | --- |
| Trem2 sgRNA #1 | GTCCCAAGCCCTCAACACCA | In-house |
| Trem2 sgRNA #2 | TATGACGCCTTGAAGCACTG | In-house |
| RNAscope Camp probe | Proprietary | ACDbio |

1.5 **Biological samples**

| **Description** | **Source** | **Identifier** |
| --- | --- | --- |
| Mouse liver tissues | This study | - |
| Mouse serum | This study | - |
| Human MASH liver transcriptomes | GEO | GSE164760 |

1.6 **Deposited data**

| **Name of repository** | **Identifier** | **Link** |
| --- | --- | --- |
| GEO | GSE308046 | <https://www.ncbi.nlm.nih.gov/geo/query/acc.cgi?acc=GSE308046> |
| GEO | GSE308029 | <https://www.ncbi.nlm.nih.gov/geo/query/acc.cgi?acc=GSE308029> |

1.7 **Software**

| **Software name** | **Manufacturer** | **Version** |
| --- | --- | --- |
| Cell Ranger | 10x Genomic | 7.1.0 |
| Seurat | Satija Lab | 5.0.0 |
| STAR | Dobin et al. | 2.7.11a |
| Samtools | HTSlib | 1.6 |
| HTSeq | Anders et al. | 2.0.3 |
| Cufflinks | Trapnell Lab | 2.2.1 |
| DESeq2 | Bioconductor | 1.50.2 |
| sva | Bioconductor | 3.44.0 |
| CellChat | Jin et al. | 2.1.0 |
| scVelo | Bergen et al. | 0.3.4 |
| Scanpy | Wolf et al. | 1.11.5 |
| CellRank | Theis Lab | 2 |
| Destiny | Bioconductor | 3.23 |
| CytoTRACE | Gulati et al. | 0.3.3 |
| GSVA | Bioconductor | 1.44.5 |
| ComplexHeatmap | Bioconductor | 2.26.1 |
| FlowJo | BD Biosciences | 10.9.0 |
| MACS iQ View | Miltenyi Biotec | 1.3.2 |
| ImageJ/Fiji | NIH | x86-64 |
| RStudio | Posit Software, PBC | 2026.1.0.392 |
| R | R Foundation for Statistical Computing | 4.5.2 |
| Python | Python Software Foundation | 3.11 |
| Spyder | The Spyder Project Contributors | 6.1.2 |

1.8 **Other (*e.g*. drugs, proteins, vectors etc.)**

| GAN diet | Research Diets | D09100310i |
| --- | --- | --- |
| LFD diet | Research Diets | D09100304i |
| G-CSF | Neupogen (Amgen) | - |
| PX458 CRISPR vector | Addgene | 48138 |
| Tumor Dissociation Kit | Miltenyi Biotec | 130-096-730 |
| Chromium Next GEM Single Cell 3' Kit v3.1 | 10x Genomics | PN-1000268 |
| Feature Barcode Kit | 10x Genomics | PN-1000262 |
| Olink Target 48 Mouse Cytokine Panel | Olink | - |
| RNAScope IntroPack 2.5 HD Reagent Kit | ACDbio | 322373 |
| MACS Tissue Storage Solution | Miltenyi Biotec | 130-100-008 |
| RNeasy Plus Mini Kit (50) | Qiagen | 74134 |
| Red Blood Cell Lysis Solution | Miltenyi Biotec | 130-094-183 |
| Percoll | Santa Cruz | sc-500790A |
| CountBright counting beads | ThermoFisher Scientific | C36950 |
| HiPerFect Transfection Reagent | Qiagen | 301704 |
| Dialyzed fetal bovine serum | ThermoFisher | 26400044 |
| FluorSave Reagent | Sigma Aldrich | 345789 |
| 1.1ml CAT-Gel microtubes | SARSTEDT | 41.1378.005 |
| RNAlater | ThermoFisher Scientific | AM7030 |
| ALT Activity Assay Kit | Abcam | ab105134 |
| Total Bile Acid Kit | Cell Biolabs | STA-631 |
| Mouse AFP ELISA Kit | R&D Systems | MAFP00 |
| Direct Red 80 | Sigma Aldrich | 365548 |
| Fast Green FCF | Sigma Aldrich | F7252 |
| Bloxall Blocking Solution | Vector Laboratories | SP-6000 |
| VECTASTAIN Elite ABC HRP Kits | Vector Laboratories | PK-6100 |
| ImmPACT DAB Peroxidase (HRP) Substrate kit | Vector Laboratories | SK-4105 |
| Permount | Fisher Scientific | SP15-500 |

1.9 **Please provide the details of the corresponding methods author for the manuscript:**

Name: Weiping Han

Address: 11 Biopolis Way #01-02, Helios, Singapore 138667

Telephone: 64788721

Email: Weiping_han@a-star.edu.sg

**2.0	Please confirm for randomised controlled trials all versions of the clinical protocol are included in the submission. These will be published online as supplementary information.**
